# The relationship between radiomics and pathomics in Glioblastoma patients: Preliminary results from a cross-scale association study

**DOI:** 10.3389/fonc.2022.1005805

**Published:** 2022-10-06

**Authors:** Valentina Brancato, Carlo Cavaliere, Nunzia Garbino, Francesco Isgrò, Marco Salvatore, Marco Aiello

**Affiliations:** ^1^ IRCCS Synlab SDN, Naples, Italy; ^2^ Department of Electrical Engineering and Information Technologies, University of Napoli Federico II, Napoli, Italy

**Keywords:** glioblastoma, radiomics, pathomics, correlation, digital pathology, WSI (whole slide image), MRI, radiopathomics

## Abstract

Glioblastoma multiforme (GBM) typically exhibits substantial intratumoral heterogeneity at both microscopic and radiological resolution scales. Diffusion Weighted Imaging (DWI) and dynamic contrast-enhanced (DCE) magnetic resonance imaging (MRI) are two functional MRI techniques that are commonly employed in clinic for the assessment of GBM tumor characteristics. This work presents initial results aiming at determining if radiomics features extracted from preoperative ADC maps and post-contrast T1 (T1C) images are associated with pathomic features arising from H&E digitized pathology images. 48 patients from the public available CPTAC-GBM database, for which both radiology and pathology images were available, were involved in the study. 91 radiomics features were extracted from ADC maps and post-contrast T1 images using PyRadiomics. 65 pathomic features were extracted from cell detection measurements from H&E images. Moreover, 91 features were extracted from cell density maps of H&E images at four different resolutions. Radiopathomic associations were evaluated by means of Spearman’s correlation (ρ) and factor analysis. p values were adjusted for multiple correlations by using a false discovery rate adjustment. Significant cross-scale associations were identified between pathomics and ADC, both considering features (n = 186, 0.45 < ρ < 0.74 in absolute value) and factors (n = 5, 0.48 < ρ < 0.54 in absolute value). Significant but fewer ρ values were found concerning the association between pathomics and radiomics features (n = 53, 0.5 < ρ < 0.65 in absolute value) and factors (n = 2, ρ = 0.63 and ρ = 0.53 in absolute value). The results of this study suggest that cross-scale associations may exist between digital pathology and ADC and T1C imaging. This can be useful not only to improve the knowledge concerning GBM intratumoral heterogeneity, but also to strengthen the role of radiomics approach and its validation in clinical practice as “virtual biopsy”, introducing new insights for omics integration toward a personalized medicine approach.

## 1 Introduction

Glioblastoma multiforme (GBM) is the most damaging tumor of the brain, characterized by an almost unavoidable propensity to relapse after rigorous treatment and carrying a fatal prognosis (1). Despite advancement in surgical and medical therapies, the overall prognosis of GBM patients remains poor, with a median survival of 10-14 months (2). One of the main reasons for the aggressive behavior and the poor outcomes of GBM is its intrinsic intra-tumor heterogeneity at both microscopic and radiological resolution scales, arising from the presence of clonal and subclonal differentiated tumor cell populations, glioma stem cells, and components of the tumor microenvironment, which affect multiple hallmark cellular functions in cancer. Recent studies have indicated that intratumor heterogeneity is partly responsible for the dismal outcome of GBM patients and this represents a significant challenge to the development of novel targeted therapies for GBM and evidence-based clinical decision-making (3, 4). In this context, numerous quantitative approaches at different imaging scales have been taken to comprehensively characterize this disease (3, 5, 6).

On a radiological point of view, it is well-known that conventional magnetic resonance imaging (MRI) provides basic anatomic and morphological information for diagnosing brain tumors and lacks the capability to illustrate tumor heterogeneity and identifies the hot spot for biopsy and surgical resection (7). Diffusion Weighted Imaging (DWI) and dynamic contrast-enhanced (DCE) MRI are two functional MRI techniques that are commonly employed in clinic for the assessment of GBM tumor characteristics (8).

T1-weighted imaging acquired following injection with a gadolinium contrast agent is used to identify regions where the active tumor has disrupted the blood-brain barrier, and contrast enhancement is used to define the extent of the primary tumor region (9). Hyperintense regions on fluid attenuated inversion recovery (FLAIR) images are thought to indicate a combination of tumor-related edema and infiltrative non-enhancing tumor. DWI can quantitatively and noninvasively reflect the random Brownian motion of water molecules within GBM tissues through quantitative apparent diffusion coefficient (ADC) maps. These maps identify areas of restricted diffusion that may indicate either hypercellular tumor or coagulative necrosis (10). Although several studies suggested that ADC values can play a valuable role in GBM diagnosis, staging, assessment of response to treatment, and prognosis (11), the biophysical mechanisms underlying changes in ADC are not always fully understood (12, 13).

Radiomics is a new frontier of medicine based on extracting numerical descriptors from radiologic images that are imperceptible by the human eye and are potentially able to describe the intratumoral heterogeneity (14). Moreover, concerning intratumoral heterogeneity, radiomics is often referred as a sort of «virtual biopsy», since it allows to enrich the traditional diagnostic radiologic workflow with more information not detectable by human eye and associated with processes not included in radiologic workflow (15). Current radiomics studies in the glioma field have shown promising results in demonstrating correlations between MRI features and GBM differential diagnosis (16), molecular characteristics (17, 18), and prognoses (11, 19, 20).

However, correlating these MRI features with underlying histopathology remains challenging (21, 22).

An interesting approach to relate radiomic results to tumor pathologic findings relies on quantitative analysis of digitized histopathology images (23). On a microscopic scale, the emerging and rapidly expanding field of pathomics aims to apply high-throughput image feature extraction techniques to interrogate the microscopic patterns in pathologic data, especially from hematoxylin-eosin–stained sections. Because of the close similarity of the approaches, the features from *in vivo* images may be compared with the features extracted from histopathological images, often benefiting from a clearer biological definition of the image patterns and hence a better understanding of the features (23, 24).

The founder hypothesis supporting the use of radiomics and pathomics in medical care is that data derived from images have a correlation with the underlying biological processes. More precisely, data derived from images would give additional information in relation to the underlying biological processes compared to the visual interpretation of the image as a picture, which is the traditional way of interpreting images (25).

Therefore, as MRI features and digital pathology offer complementary sources of information about the tumor *in vivo* and *in vitro*, a natural question is whether radiomics and pathomics features might be connected.

The promises arising from the integration between data at different imaging scales would surely be the improvement of diagnostics and molecular knowledge about GBM, for diagnostic, prognostic and therapeutic purposes, and this would have direct implications in clinical decision-making process (26). Radiomics and pathomics could fill the need to assess tumor heterogeneity, which strongly characterize GBM. Moreover, the radiopathomic integration could be beneficial for validating the radiomic approach in clinical practice as “virtual biopsy” (15, 27).

Previously, it has been shown that both radiomic and pathomic image-based signatures can independently predict outcomes of interest in GBM (5, 6, 21). Moreover, some studies on GBM and other cancer types support the hypothesis that combining radiomic and pathomic features will even further improve prognostication and enhance the understanding of the disease by means of predictive models (28–31). However, before making predictions it is important to understand if there are actually correlations between radiomic and pathomic characteristics.

To date, few studies have explored the associations between MRI images of GBM patients (which interrogate tumor at macroscopic scale) and histopathologic images (which depict tumor at microscopic scale).

Preliminary results reported an inverse correlation between ADC-based features and basic pathomic features such as tumor cellularity in GBM (32, 33). Moreover, there have been recent efforts to identify cross-scale associations between radiology and pathology scales by investigating on more complicated radiomics and pathomic features in different cancer types (of which brain tumors) (22, 23, 34). However, these findings were inconsistent mainly due to the small number of patients involved in the studies, and image-based tumor heterogeneity and radiomics features have not yet been exactly correlated with findings in histopathology.

In light of the above, this works presents initial results aiming at determining if radiomics features extracted from preoperative ADC maps and post-contrast T1 (T1C) images are associated with histological features (pathomic features) arising from H&E digitized pathology images of patients with GBM. In particular, we aimed at identifying pathomic features from digitized histopathology that potentially reflect tissue composition basis of radiomic descriptors from MRI, towards improving the understanding of GBM heterogeneity.

## 2 Methods

### 2.1 Patients

The study was conducted in accordance with the Declaration of Helsinki, and the study protocol was approved by the Ethics Committee of the Istituto Nazionale Tumori “Fondazione G. Pascale (protocol number 1/20). The subjects used for the study belong to the public database CPTAC-GBM (Clinical Proteomic Tumor Analysis Consortium-Glioblastoma Multiforme), accessed on October 2021 (35). Radiology images, clinical data, digital histopathology slides, and associated quantified features (cellularity, necrosis, tumor nuclei, age, tumor weight) of samples included were downloaded from The Cancer Imaging Archive (TCIA) database (36). The inclusion criteria were the following: availability of pretreatment T1C images and ADC maps, availability of corresponding digital pathology whole-slides images (WSI), WSI slides with at least 70% of tumor nuclei and at most 20% necrosis. Moreover, patients were excluded in case of i) insufficient quality of MRI to perform imaging analysis and/or obtain measurements, ii) insufficient quality of WSI that did not meet the requirements for diagnosis (e.g., tissue folds, torn tissue) and iii) images with a positive value of Clinical Trial Time Point ID (corresponding to the number of days from the date the patient was initially diagnosed pathologically with the disease to the date of the scan). Finally, a total of 48 subjects were included in the study. Refer to **Table 1** for clinical characteristics and outcomes of included patients.

**Table 1:** Clinical and pathologic characteristics of the included patients.

Clinical and pathologic characteristics	Value
Age [mean ± SD]	62.3 **±** 11.3
Sex [n (%)]	
Male	34 (70.8)
Female	14 (29.1)
BMI [mean ± SD]	62.3 **±** 11.3
Risk factors [n (%)]	
Alcohol	
≤ 2 drinks per day (men)**/**≤ 1 (women)	15 (31.2)
> 2 drinks per day (men)**/**> 1 (women)	2 (4.2)
Consumed in the past	2 (4.2)
Lifelong non-drinker	16 (33.3)
NR	13 (27)
Tobacco	
Smoker ≤ 15 years	2 (4.2)
Smoker > 15 years	6 (12.5)
Current smoker	8 (16.7)
Lifelong non-smoker	24 (50)
NR	8 (16.7)
Progression/Recurrence [n (%)]	
Y	19 (39.6)
N	16 (33.3)
NR	13 (27)
OS [n (%)]	
≤ 12 mo	22 (45.8)
> 12 mo	16 (33.3)
NR	10 (20.8)

BMI, Body Mass Index; Y, Yes; N, No; NR, not reported; OS, Overall Survival; mo, months.

### 2.2 MRI acquisition and processing

MR examinations with contrast injection were performed on 1.5 and 3 Tesla (T) equipment (34 patients=1.5T; 12 = 3T and 2=the magnetic field was not reported). The acquisition protocol included: axial FLAIR sequence (TE (echo time)=71-155 ms; TR (repetition time)=7752-12000 ms; slice thickness=4-5 mm; acquisition matrix=128x256-512x192); axial DWI MR sequence with ADC (apparent diffusion coefficient) map (TE=27-149 ms; TR=1000-9601 ms; slice thickness=2-5 mm; acquisition matrix=120x180-512x192); axial post-contrast 3D/2D T1-weighted MR sequence (10=had contrast 2D acquisition; 19=with 2D sequence and 20=both 2D/3D acquisition) (TE=17-130 ms; TR=35-2140 ms; slice thickness=0.8-5 mm; acquisition matrix=252x250-384x256).

The MRI scans were converted from DICOM format to NIfTI format using dcm2niix software (37). ADC maps were coregistered to T1C images by means of two-steps registration procedure both performed by using Elastix registration software (38). Considering the multimodal registration strategy proposed by Leibfarth et al. (39), a first step a rigid registration was performed to obtain a rough alignment of the fixed and moving images. A two-level multiresolution approach was applied using a Gaussian smoothing without downsampling. A localized version of mutual information was considered as similarity measure and consisted in evaluating mutual information on multiple subregions. Specifically, the localization is obtained by constraining the sampling procedure to a cubic subregion of the image, randomly chosen in every iteration step from the fixed image domain (39, 40). The standard gradient descent was applied for metric optimization (41). The resulting transformation matrix was used to initialize the following deformable registration step. In particular, a two-level multiresolution approach using 3D Gaussian smoothing without downsampling was used together with a bending energy penalty term calculated to regularize the transformation. Finally, the similarity metric consisted in a combination of localized mutual information and bending energy penalty and the adaptive stochastic gradient descent optimizer was adopted for its minimization (41). B-spline was used as interpolation method for the registration procedure. Volumes of interest (VOIs) were then manually delineated slice-by-slice by using ITK-SNAP (version 3.6.0, http://www.itksnap.org) on the T1C. VOIs consisted of T1C enhancing regions. Areas of intrinsic T1 hyperintensity representing hemorrhagic material were not included in T1C contour delineations. Necrotic/cystic regions and large vessels were excluded from all VOIs. The same 3D ROIs were applied to the registered ADC maps, and mean ADC values were extracted for each enhancing tumor VOI.

### 2.3 WSI acquisition and processing

H&E-stained formalin-fixed paraffin-embedded (FFPE) tissues from surgical resection of primary tumor were used for pathological diagnosis. The slides were digitalized to SVS format at 20x magnification (resolution = 0.494 μm/px) (42). All the WSI images were manually checked for artifacts, and the images free of all types of artifacts were chosen. WSIs of H&E staining slides without any preprocessing were imported in QuPath digital pathology software (43), and regions covering the largest possible tissue area with viable tissue with vivid histopathologic characteristics and free of artifacts were delineated by an expert microscopist.

### 2.4 Radiomic features extraction

Prior to radiomic features extraction, normalization was applied on T1C images intensities. Specifically, intensities were normalized by centering them at their respective mean value with the standard deviation of all gray values in the original image (44–46).

91 radiomics features were extracted from segmented VOIs on the enhanced region from T1C and ADC map by using the opensource Python package PyRadiomics (https://pyradiomics.readthedocs.io/en/Latest/). The extracted radiomics features were categorized into two groups: Firstorder features including 18 intensity statistics; 73 multi-dimensional texture features including 23 gray level co-occurrence matrix (GLCM), 16 gray level size zone matrix (GLSZM), 16 gray level run length matrix (GLRLM), 14 gray level dependence matrix (GLDM) and 5 neighboring gray tone difference matrix (NGTDM) features. The extracted radiomics features grouped by similarity in four categories are listed in the **Supplementary Table S1**. The computing algorithms can be found at www.radiomics.io and the image biomarker standardization initiative (IBSI) presented a document to standardize the nomenclature and definition of radiomic features (47).

### 2.5 Pathomic features extraction

#### 2.5.1 Detection measurements

WSIs of H&E staining slides were imported in QuPath digital pathology software to carrying out cell and nuclear segmentation. By applying the cell detection function from the analysis module, the nuclear segmentation was performed to recognize objects through watershed cell detection based on segmentation parameters (48, 49), including morphology features of cell, nuclear, and cytoplasm. The setup parameter was set as hematoxylin OD for detection image, with pixel size of 0.5 μm. For nucleus parameters, the background radius and sigma were set as 8 and 1.5 μm, respectively. For intensity parameters, the threshold was set as 0.1 and the max background intensity was set as 2. Other parameters were set as their respective default values. The quality of the automated cell detection was checked by an expert microscopist.

Detection measurements were calculated for all cells using QuPath’s add intensity features option (preferred pixel size: 0.5 μm, region: ROI, tile diameter: 25 μm, compute all features including Haralick features with 32 bins) and spatial analysis. Measurements included shape characteristics, Optical Density Sum (ODSum) Haralick texture features (50), and Delaunay triangulation measurements (51).

65 features were measured for each detection on the candidate slide (**Supplementary Table S2**) and exported into a tab delimited file using a QuPath script developed explicitly for this purpose.

Detection measurements were aggregated across the case-level tiles by the mean of the values. For patients with more than one slide, the value of each feature was averaged across WSIs.

Moreover, annotation measurements were also computed (WSI selected area, nuclei count, extracellular area, sum of cytoplasm, and extracellular area).

Nuclear segmentation and feature extraction process was performed by means of Groovy scripts implemented on QuPath script editor. Exported measurements were imported into R version 3.4.2 for statistical analysis.

#### 2.5.2 Cell density maps features

Cell density maps associated with ROIs placed on WSI were calculated at different resolutions (50μm, 100μm, 150μm, 200μm). Specifically, the cellular density was estimated in each tile of dimension associated with the resolution by using the segmented nuclei and assigning a gray level to each of these tiles based on the number of nuclei estimated in each tile. For each resolution, this resulted in a spatial map of the cellular density of the digitized histopathology. 91 features were extracted from cell density maps of digital pathology images using PyRadiomics [https://pyradiomics.readthedocs.io/en/latest/] (**Supplementary Table S2**). For patients with more than one slide, the value of each feature was averaged across WSIs.

### 2.6 Radiopathomic analysis

**Figure 1:**
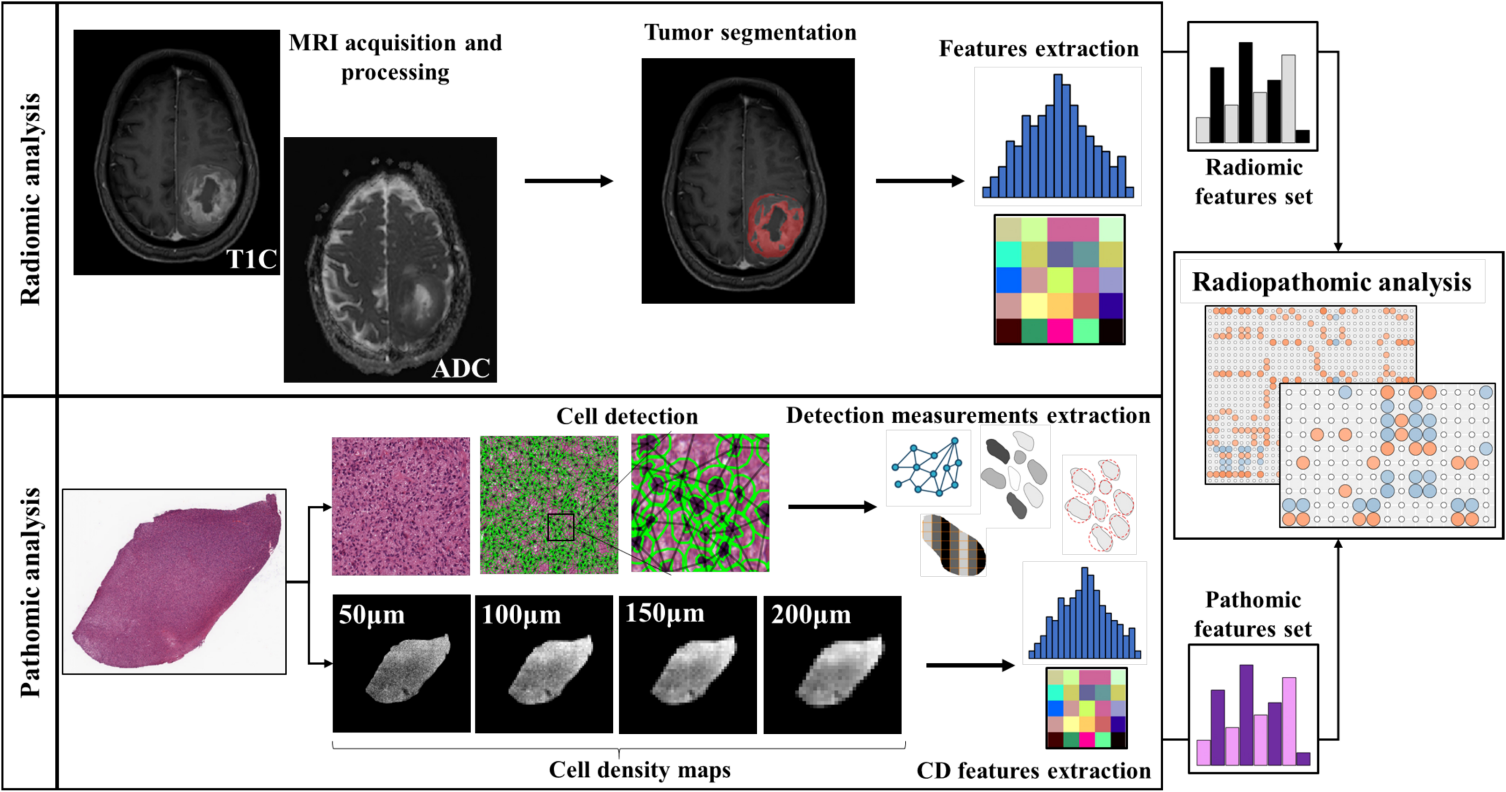
Workflow of the radiopathomic analysis implemented in the study. On the first row the radiomic analysis steps. On the second row the pathomic analysis steps.

An integrative study design was defined and reported as radiopathomic workflow in **Figure 1** to evaluate potential association between radiomic features and pathomic features for the included patients. After preliminary analyses, a deeper radiopathomic analysis including correlation analysis and factor analysis was performed. The details of each analysis are reported in the next three paragraphs. **Supplementary Analyses** were also performed to investigate radiopathomic associations including higher-order features obtained applying wavelet and local binary pattern (LBP) filters to the images (See **Supplementary Materials** - Section 4).

#### 2.6.1 Preliminary analysis

Preliminary analysis aiming at investigating the relationship between ADC mean and basic histopathologic features related to cellular density and commonly associated with ADC meaning (nuclei count, area of the extracellular space and the sum of extracellular space, and cytoplasm) were performed (32, 52). These features were normalized for the region considered in the WSI.

#### 2.6.2 Correlation analysis

Then, a deeper radiopathomic correlation analysis was conducted to explore more detailed associations between extracted pathomic and radiomic features detailed in the previous two paragraphs. Radiopathomic associations were performed considering two separate tasks for ADC and T1C images.

A preliminary features selection was performed separately for radiomic features from ADC, radiomic features from T1C and pathomic features. Specifically, a correlation filter based on the absolute values of pairwise Spearman’s correlation (ρ) coefficient was used to reduce feature redundancy. Threshold for ρ was set to 0.9. Briefly, if two features had ρ > 0.9, the function looks at the mean absolute correlation of each variable and the variable with the largest mean absolute correlation is removed. Spearman correlation analysis was also employed to determine the correlation of radiomics features with pathomic features. Specifically, we calculated ρ between ADC radiomic feature set selected after the correlation filter and the selected pathomic features surviving after the correlation filter step. The same analysis was performed considering T1C radiomic features. p values were adjusted for multiple correlations by using a false discovery rate (FDR) adjustment. An FDR-adjusted p-value (q-value) below 0.05 was considered statistically significant. Considering ρ in absolute value, the strength of correlation was described as very weak if ρ = 0.00-0.19, weak if ρ = 0.20-0.39, moderate if ρ = 0.40-0.59, strong if ρ = 0.60-0.79, and very strong if ρ = 0.80-1.00 (53).

Correlation analyses were complemented with Bayes Factors estimation. The Bayes Factor quantifies the evidence for or against the null hypothesis as the ratio of the likelihoods for the experimental and the null hypothesis (54). It can be expressed as the logarithm of the ratio (55, 56), where negative numbers indicate that the null hypothesis is likely to be true, positive that it is false. By convention, absolute log Bayes factors greater than 0.5 are considered substantial evidence for or against, and absolute log-factors greater than 1 strong evidence (57, 58).

The statistical analysis was performed using R version 4.0.2.

#### 2.6.3 Factor analysis

Factor analysis was performed to project the radiomic and pathomic features onto a lower-dimensional latent-feature space that retaining most of the information contained in the whole feature set (59). For each feature group (ADC, T1C and pathomic), the remaining correlation matrix after correlation filter was subjected to ridge-regularization and 5-fold cross-validation of the log-likelihood was used to determine the optimal value of the penalty-parameter. Feature normalization was performed using z-normalization to avoid the predominance of features with the largest scale in the analysis (60). The regularized feature-correlation matrix was used as input for projection by a maximum likelihood factor analysis procedure (factor analytic data compression step) (59). In particular, a latent lower dimensional orthogonal meta-feature space representing the projection of the shared information in a feature set was obtained. The number of factors, corresponding to the dimension of the latent space, was determined by Guttman bounds (61). The factor-solution was rotated to a simple orthogonal structure. After projection of the original variable-space onto the lower-dimensional factor-space, factor scores were obtained by regressing the latent features on the observed data by means of the obtained factor solution (59). Factor analysis was performed using the R FMradio (Factor Modelling for Radiomics Data) package (R version 4.0.2). The correlation between resulting factor scores associated with radiomics and pathomic factors were investigated and complemented with Bayes Factors estimation. An FDR-adjusted p-value (q-value) below 0.05 was considered statistically significant.

## 3 Results

### 3.1 Preliminary analysis

Preliminary analysis of ADC value and basic histopathological features revealed weak but significant correlations between ADC and nuclei count (ρ = -0.317, p = 0.0286) and extracellular space (ρ = 0.3029, p = 0.0368). A positive but not significant correlation was found between ADC and the sum of extracellular space and cytoplasm (ρ = 0.2560, p = 0.0792).

### 3.2 Correlation analysis

Concerning radiomic features, the correlation filter step reduced the feature set from 91 to 46 for ADC and from 91 to 53 for T1C. On the other hand, pathomic features were reduced from 429 to 232. Radiopathomic analysis between selected ADC radiomic features and pathomic features revealed 186 significant correlations (based on adjusted p-values after FDR correction), of which 31 negative correlations (-0.6909 <ρ<-0.4578, 22.9454 <BF< 2.587×10^5^) and 155 positive correlations (0.4556 <ρ< 0.7395, 21.6563 < BF <6.461×10^5^). Among features constituting the ADC-radiopathomic couples showing significant cross-scale associations, ADC radiomic features included 10 firstorder features and 23 texture features, while pathomic features included Mean Cytoplasm Eosin OD Min, 5 nuclear Haralick features and 22 cell-density map features (of which 6 from 50μm resolution, 6 from 100μm resolution, 6 from 150μm resolution and 4 from 200μm resolution).

Concerning ADC pipeline, most of the strong relationships (35/50) involved textural features from cell density maps, of which 10 correspond to associations with ADC firstorder features and the remaining 25 were with textural ADC features. The remaining strongest associations (15/50) involved intranuclear Haralick texture features (Haralick Angular Second Moment F0 and Information measure of correlation 2 F12) with ADC firstorder features (10 associations) and textural features (5 associations).

Radiopathomic analysis between selected T1C radiomic features and pathomic features revealed 53 significant correlations (based on adjusted p-values after FDR correction), of which 24 negative correlations (-0.6524<ρ< -0.5078, 22.9454 < BF < 2.587×10^5^) and 29 positive correlations (0.5064<ρ< 0.6472, 91.2984< BF <2.3688×10^4^). Among features constituting the T1C-radiopathomic couples showing significant cross-scale associations, T1C radiomic features included 4 firstorder features and 11 texture features, while pathomic features included 2 nuclear Haralick features and 9 cell-density map features (of which 1 from 50μm resolution, 2 from 100μm resolution, 3 from 150μm resolution and 3 from 200μm resolution).

Concerning T1C pipeline, similar finding were observed, with most of the strongest associations found between cell density map textural features and T1C firstorder (9/15) or textural features (3/15), and the remaining three associations involving the same two intranuclear Haralick texture features observed in the ADC results and two T1C textural features (glcm Joint Energy and ngtdm Coarseness). Similar findings, concerning both ADC and T1C radiopathomic tasks, were observed for significant moderate correlations (See **Supplementary Table S3**).

**Figure 2:**
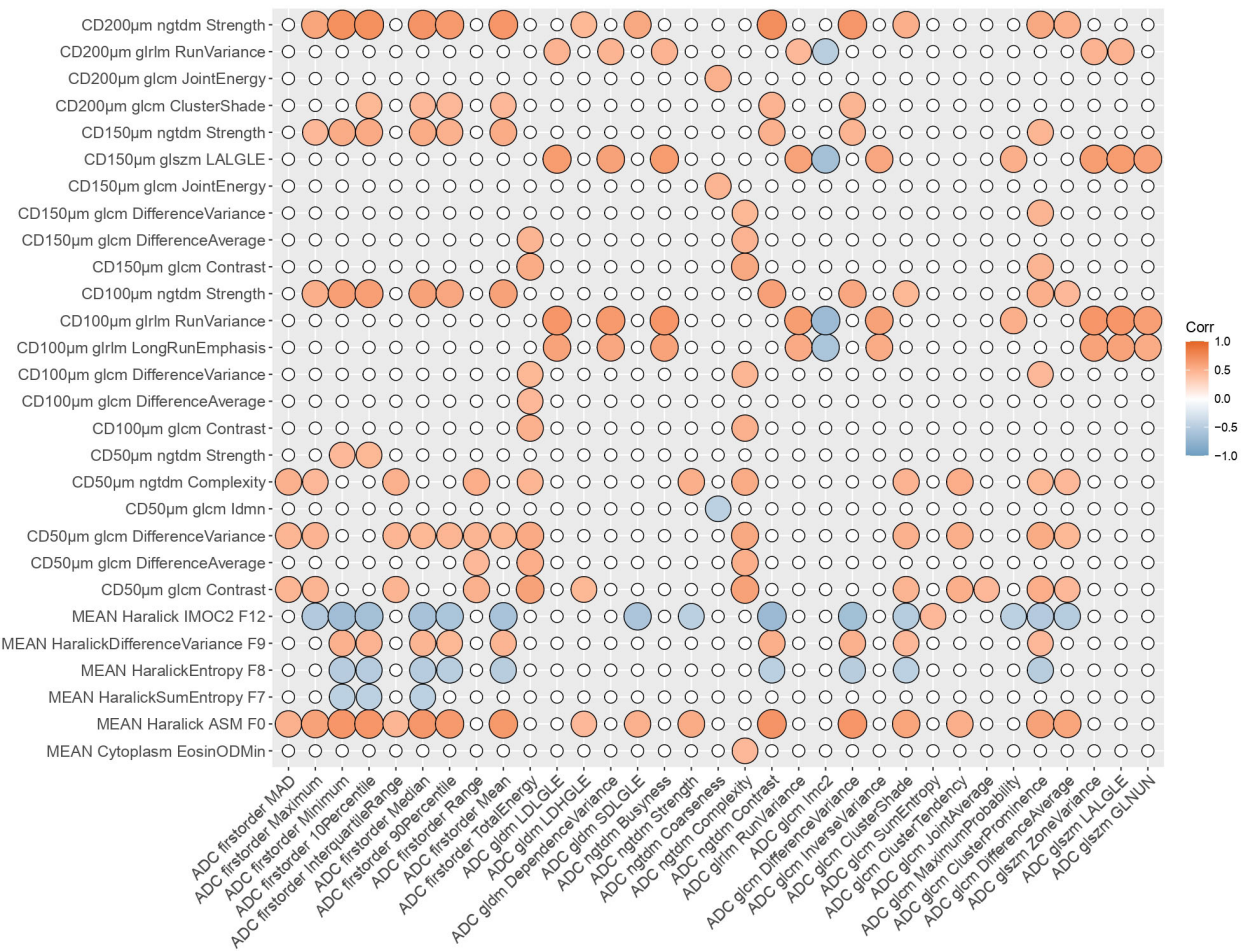
Radiopathomic analysis between ADC radiomic features and pathomic features. Correlation matrix filtered from nonsignificant correlations (rows and columns with non significant values were deleted, while nonsignificant values surviving were set to zero). CD, Cellular Density; ADC, Apparent Diffusion Coefficient; LALGLE, Large Area Low Gray Level Emphasis; IMOC, Information Measure Of Correlation; ASM, Angular Second Moment; LDLGLE, Large Dependence Low Gray Level Emphasis; LDHGLE, Large Dependence High Gray Level Emphasis; SDLGLE, Small Dependence Low Gray Level Emphasis; LALGLE, Large Area Low Gray Level Emphasis; GLNUN, Gray level non uniformity normalized; glcm, gray level co-occurrence matrix; gldm, Gray Level Dependence Matrix; glszm, Gray Level Size Zone Matrix; ngtdm, Neighbouring Gray Tone Difference Matrix; glrlm, Gray Level Run Length Matrix.

**Figure 3:**
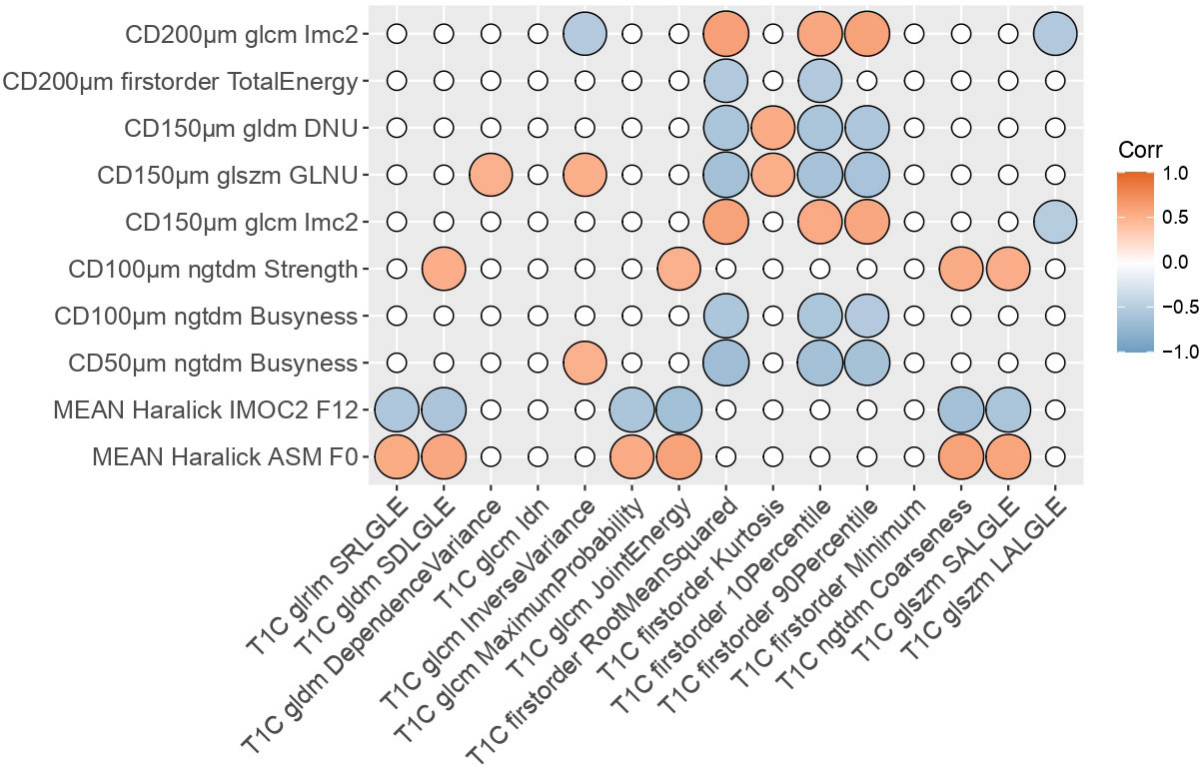
Radiopathomic analysis between T1C radiomic features and pathomic features. Correlation matrix filtered from nonsignificant correlations (rows and columns with non-significant values were deleted, while surviving nonsignificant values were set to zero). DNU, Dependence non uniformity; GLNU, gray-level non-uniformity; IMOC, Information Measure Of Correlation; ASM, Angular Second Moment; SRLGLE, Short Run Low Gray Level Emphasis; SDLGLE, Small Dependence Low Gray Level Emphasis; T1C, post-contrast T1; SALGLE, Small Area Low Gray Level Emphasis; LALGLE, Large Area Low Gray Level Emphasis; glcm, gray level co-occurrence matrix; gldm, Gray Level Dependence Matrix; glszm, Gray Level Size Zone Matrix; ngtdm, Neighbouring Gray Tone Difference Matrix; glrlm, Gray Level Run Length Matrix.

**Figures 2** and **3** show the resulting correlation heatmaps, displaying Spearman’s ρ between radiomic features (from ADC and T1C, respectively) and pathomic features.

Strongest radiopathomic associations (|ρ| ≥ 0.6) sorted by ρ strength are presented in **Tables 2** and **3**. Refer to **Supplementary Table S3** for the complete set of significant correlated radiopathomic features pairs.

**Table 2:** Summary of the highest-correlated (ρ>0.6) radiomic-pathomic features, with radiomic features extracted from ADC.

Radiomic feature name	Pathomic featµre name	ρ	FDR q-value	BF
ADC ngtdm Contrast	CD200µm ngtdm Strength	0.739	1.48×10^-5^	6.46×10^6^
ADC firstorder Minimum	CD200µm ngtdm Strength	0.729	1.48×10^-5^	3.15×10^6^
ADC firstorder 10Percentile	CD200µm ngtdm Strength	0.725	1.48×10^-5^	2.36×10^6^
ADC firstorder Median	CD200µm ngtdm Strength	0.698	4.12×10^-5^	4.06×10^5^
ADC ngtdm Contrast	MEAN Haralick ASM F0	0.697	4.12×10^-5^	3.74×10^5^
ADC firstorder Minimum	MEAN Haralick ASM F0	0.694	4.12×10^-5^	3.16×10^5^
ADC gldm LDLGLE	CD100µm glrlm RunVariance	0.693	4.12×10^-5^	3.00×10^5^
ADC glszm LALGLE	CD100µm glrlm RunVariance	0.693	4.12×10^-5^	2.93×10^5^
ADC firstorder Mean	CD200µm ngtdm Strength	0.692	4.12×10^-5^	2.75×10^5^
ADC ngtdm Contrast	MEAN Haralick IMOC2 F12	-0.691	4.12×10^-5^	2.59×10^5^
ADC ngtdm Busyness	CD100µm glrlm RunVariance	0.69	4.12×10^-5^	2.42×10^5^
ADC glszm ZoneVariance	CD100µm glrlm RunVariance	0.687	4.12×10^-5^	2.07×10^5^
ADC glcm DifferenceVariance	MEAN Haralick ASM F0	0.687	4.12×10^-5^	2.05×10^5^
ADC firstorder 10Percentile	MEAN Haralick ASM F0	0.687	4.12×10^-5^	2.01×10^5^
ADC glcm DifferenceVariance	CD200µm ngtdm Strength	0.679	6.01×10^-5^	1.31×10^5^
ADC firstorder Median	MEAN Haralick ASM F0	0.676	6.67×10^-5^	1.09×10^5^
ADC glcm Imc2	CD100µm glrlm RunVariance	-0.675	6.67×10^-5^	1.05×10^5^
ADC firstorder Mean	MEAN Haralick ASM F0	0.672	7.40×10^-5^	9.00×10^4^
ADC firstorder 90Percentile	CD200µm ngtdm Strength	0.66	1.44×10^-4^	4.52×10^4^
ADC firstorder 90Percentile	MEAN Haralick ASM F0	0.656	1.67×10^-4^	3.71×10^4^
ADC gldmDependenceVariance	CD100µm glrlm RunVariance	0.655	1.67×10^-4^	3.55×10^4^
ADC firstorder Minimum	MEAN Haralick IMOC2 F12	-0.654	1.67×10^-4^	3.27×10^4^
ADC glszm LALGLE	CD150µm glszm LALGLE	0.653	1.67×10^-4^	3.17×10^4^
ADC gldm LDLGLE	CD150µm glszm LALGLE	0.653	1.67×10^-4^	3.14×10^4^
ADC ngtdm Busyness	CD150µm glszm LALGLE	0.648	2.05×10^-4^	2.48×10^4^
ADC glszm ZoneVariance	CD150µm glszm LALGLE	0.644	2.44×10^-4^	2.03×10^4^
ADC glcm DifferenceVariance	MEAN Haralick IMOC2 F12	-0.641	2.72×10^-4^	1.76×10^4^
ADC glrlm RunVariance	CD100µm glrlm RunVariance	0.64	2.78×10^-4^	1.67×10^4^
ADC firstorder 10Percentile	CD100µm ngtdm Strength	0.634	3.58×10^-4^	1.27×10^4^
ADC glcm Imc2	CD150µm glszm LALGLE	-0.63	4.39×10^-4^	1.01×10^4^
ADC firstorder Minimum	CD100µm ngtdm Strength	0.628	4.46×10^-4^	9.50×10^3^
ADC firstorder 10Percentile	MEAN Haralick IMOC2 F12	-0.628	4.46×10^-4^	9.29×10^3^
ADC glszm GLNUN	CD100µm glrlm RunVariance	0.627	4.46×10^-4^	9.10×10^3^
ADC glszm GLNUN	CD150µm glszm LALGLE	0.622	5.71×10^-4^	6.98×10^3^
ADC ngtdm Contrast	CD100µm ngtdm Strength	0.621	5.87×10^-4^	6.62×10^3^
ADC glcm ClusterProminence	MEAN Haralick ASM F0	0.614	7.70×10^-4^	4.98×10^3^
ADC firstorder Median	CD100µm ngtdm Strength	0.613	7.74×10^-4^	4.83×10^3^
ADC ngtdm Complexity	CD50µm glcm Contrast	0.61	8.56×10^-4^	4.21×10^3^
ADC gldmDependenceVariance	CD150µm glszm LALGLE	0.61	8.56×10^-4^	4.11×10^3^
ADC firstorder TotalEnergy	CD50µm glcm Contrast	0.61	8.56×10^-4^	4.07×10^3^
ADC glcm InverseVariance	CD100µm glrlm RunVariance	0.609	8.64×10^-4^	3.94×10^3^
ADC firstorder Mean	CD100µm ngtdm Strength	0.607	9.13×10^-4^	3.66×10^3^
ADC firstorder Maximum	MEAN Haralick ASM F0	0.606	9.37×10^-4^	3.42×10^3^
ADC firstorder Median	MEAN Haralick IMOC2 F12	-0.606	9.37×10^-4^	3.41×10^3^
ADC gldm LDLGLE	CD100µm glrlm LongRunEmphasis	0.604	9.43×10^-4^	3.23×10^3^
ADC firstorder Mean	MEAN Haralick IMOC2 F12	-0.604	9.43×10^-4^	3.22×10^3^
ADC glszm LALGLE	CD100µm glrlm LongRunEmphasis	0.604	9.43×10^-4^	3.19×10^3^
ADC ngtdm Busyness	CD100µm glrlm LongRunEmphasis	0.602	9.95×10^-4^	2.97×10^3^
ADC glrlm RunVariance	CD150µm glszm LALGLE	0.601	1.02×10^-3^	2.84×10^3^
ADC glszm ZoneVariance	CD100µm glrlm LongRunEmphasis	0.6	1.04×10^-3^	2.73×10^3^

ADC, Apparent Diffusion Coefficient; CD, Cellular Density; FDR, False Discovery Rate; BF, Bayes Factor; LDLGLE, Large Dependence Low Gray Level Emphasis; LALGLE, Large Area Low Gray Level Emphasis; GLNUN, Gray level non uniformity normalized; LDLGLE, Large Dependence Low Gray Level Emphasis; ASM, Angular Second Moment; IMOC, Information Measure Of Correlation; glcm, gray level co-occurrence matrix; glszm, Gray Level Size Zone Matrix; ngtdm, Neighbouring Gray Tone Difference Matrix; glrlm, Gray Level Run Length Matrix.

**Table 3:** Summary of the highest-correlated (ρ>0.6) radiomic-pathomic features, with radiomic features extracted from T1C.

Radiomic feature name	Pathomic featµre name	ρ	FDR q-value	BF
T1C firstorder RootMeanSquared	CD50µm ngtdm Bµsyness	-0.652	2.48×10^-3^	3.09×10^4^
T1C glcm JointEnergy	CD200µm ngtdm Strength	0.647	2.48×10^-3^	2.37×10^4^
T1C ngtdm Coarseness	CD200µm ngtdm Strength	0.644	2.48×10^-3^	2.05×10^4^
T1C glcm JointEnergy	MEAN Haralick IMOC2 F12	-0.636	2.48×10^-3^	1.39×10^4^
T1C firstorder RootMeanSquared	CD150µm glszm GLNU	-0.632	2.48×10^-3^	1.12×10^4^
T1C firstorder 10Percentile	CD50µm ngtdm Bµsyness	-0.631	2.48×10^-3^	1.05×10^4^
T1C ngtdm Coarseness	MEAN Haralick IMOC2 F12	-0.629	2.48×10^-3^	9.88×10^3^
T1C firstorder RootMeanSquared	CD200µm glcm Imc2	0.621	3.15×10^-3^	6.73×10^3^
T1C firstorder 90Percentile	CD50µm ngtdm Bµsyness	-0.619	3.15×10^-3^	6.18×10^3^
T1C firstorder 10Percentile	CD150µm glszm GLNU	-0.61	4.26×10^-3^	4.20×10^3^
T1C ngtdm Coarseness	MEAN Haralick ASM F0	0.604	4.27×10^-3^	3.24×10^3^
T1C firstorder 90Percentile	CD150µm glszm GLNU	-0.603	4.27×10^-3^	3.09×10^3^
T1C firstorder 90Percentile	CD200µm glcm Imc2	0.602	4.27×10^-3^	2.98×10^3^
T1C firstorder RootMeanSquared	CD150µm glcm Imc2	0.602	4.27×10^-3^	2.91×10^3^
T1C gldm SDLGLE	CD200µm ngtdm Strength	0.601	4.27×10^-3^	2.86×10^3^

T1C, post-contrast T1; CD, Cellular Density; FDR, False Discovery Rate; BF, Bayes Factor; SDLGLE, Small Dependence Low Gray Level Emphasis; IMOC, Information Measure Of Correlation; GLNU, Grey Level Non Uniformity; ASM, Angular Second Moment; glcm, gray level co-occurrence matrix; glszm, Gray Level Size Zone Matrix; ngtdm, Neighbouring Gray Tone Difference Matrix; glrlm, Gray Level Run Length Matrix.

### 3.3 Factor analysis

The factor analytic data compression of the regularized correlation matrix resulted in 24, 32, and 27 latent factors, respectively for ADC, T1C and pathomic. These retained 76%, 89% and 81% of the covariation between the original 46 (for ADC), 53 (for T1C) and 232 (for pathomic) features. Loadings measuring the association between features and factors for ADC, DCE and pathomic feature analysis were reported in **Supplementary Tables S5–S7**, respectively.

Having established a compact representation of ADC, T1C and pathomic features in terms of factors, correlation analysis between factors scores revealed significant correlations based on adjusted p-values after FDR correction (**Supplementary Tables S8** and **S9**). To facilitate the reading of factor analysis results, the term “Factor” was abbreviated to “F”. Concerning ADC pipeline, 5 significant correlations were found, of which 3 negative correlations (ρ = -0.54, BF = 2.99×10^2^ between ADC F5 and pathomic F11; ρ = -0.53, BF = 2.55×10^2^ between ADC F4 and pathomic F11; ρ = -0.52, BF = 1.65×10^2^ between ADC F1 and pathomic F5) and 2 positive correlations (ρ = 0.51, BF = 1.24×10^2^ between ADC F1 and pathomic F17; ρ = 0.48, BF = 36.3 between ADC F2 and pathomic F14). Among factors constituting the ADC-radiopathomic couples showing significant cross-scale associations, ADC F1 consisted mainly of firstorder features (mean absolute deviation, maximum, minimum, 10^th^ and 90^th^ percentile, median and interquartile range) and textural glcm features (difference average and cluster shade, tendency and prominence); ADC F2 consisted mainly of glcm dependence variance and large dependence low gray level emphasis, Informational Measure of Correlation from glcm and three glszm features (zone variance, large area low gray level emphasis, gray level non-uniformity normalized); ADC F4 consisted mainly of textural features associated with entropy (gldm dependence entropy, glcm difference entropy and glszm zone entropy); ADC F5 consisted mainly of ngtdm complexity and firstorder total energy. Considering pathomic factors, F11 was mainly related to texture strength from ngtdm of cell density maps at different resolutions (100μm, 150μm, 200μm); F14 consisted mainly of morphological nuclear features (circularity and eccentricity); F17 is mainly related to dependence variance and non-uniformity normalized from gldm of 150μm cell density maps. Concerning T1C pipeline, 2 significant and negative correlations were found, in particular between T1C F2 and pathomic F11 (ρ = -0.63, BF = 9.14×10^3^) and between T1C F5 and pathomic F8 (ρ = -0.53, BF = 2.52×10^2^). Among factors constituting the T1C-radiopathomic couples showing significant cross-scale associations, T1C F2 is mainly related to short run emphasis, small dependence and small area low gray level from glrlm, gldm and glszm, respectively, and maximum probability and joint energy from glcm; T1C F5 is mainly composed of firstorder features (10^th^ percentile, root mean squared, minimum). Considering pathomic factors, F11 was described above since it also appeared in ADC-radiopathomic associations, while F8 consisted mainly of busyness from ngtdm of cell density maps at different resolutions (50μm, 100μm) and informational measure of correlation from glcm of cell density maps at 150μm and 200μm resolutions.

## 4 Discussion

The possibility to integrate data from different imaging scales in a common framework opens the improvement of diagnostics and molecular knowledge about GBM and its heterogeneity and provides an advantageous context to validate radiomic approach as a clinical “virtual biopsy” tool (15, 23). Multiparametric MRI and digital pathology images from biopsy samples are currently acquired as standard clinical practice for GBM and provide information that are essential to making correct diagnoses, appropriate patient management and treatment decisions. However, the isolation of radiology and pathology workflows, and consequently of the analysis of quantitative data arising from radiology (radiomics) and digital pathology (pathomics) makes it hard to harness the potential arising from the integration of quantitative data at different imaging scales (25, 62).

In this preliminary study, we evaluated cross-scale associations between radiomic and pathomic descriptors in patients with GBM, quantitatively correlating imaging features extracted by functional MRI images with histological features extracted from H&E digitized slices from surgical resections. In this study, we focused on commonly acquired functional MR sequences, including T1C and ADC from DWI, as they are both part of the routine examination for patients with GBM and are functional modalities, meaning they can provide functional information about GBM and are able to detect tumor volume and physiological changes beyond the lesions shown on conventional morphological MRI (8).

Firstly, we investigated the relationship between ADC mean value and basic histopathologic features related to cellular density, namely nuclei count, area of the extracellular space and the sum of the area of the latter with that of the cytoplasm, that commonly associated with ADC meaning (32, 52). Indeed, in the field of glial tumors, quantitative assessment of ADC with DWI has mainly been considered as imaging biomarker for its estimation of cellularity based on its inverse relation with water diffusivity in the extracellular compartment (the higher the tumor grade, the lower the mean tumor ADC values) (63). Our results supported this assumption, showing a significant negative correlation between ADC and nuclei count, and a positive correlation between ADC and extracellular space and the sum of extracellular space and cytoplasm. These findings were in line with results from the meta-analysis by Surov et al. (32), which found a strong inverse correlation between ADC and cellularity in glioma. An inverse correlation between ADC and cellularity in GBM was also found by Eidel et al. (33) who adopted an approach including trajectory analysis and automatic nuclei counting for the analysis. However, these results should be carefully interpreted mainly due to the extremely small populations investigated in the studies. Of note, we found that the value of correlation with ADC mean decreased when considering the sum of extracellular space and cytoplasm area than extracellular space area alone, and this could be associated with the bias introduced by water in cytoplasm (33).

Preliminary analyses were not performed for T1C since, differently from ADC map that corresponds to a quantitative measurement that is supposed to reflect water diffusivity and thus to be affected by tissue proprieties (e.g., cellularity, cell size, nuclear size, necrosis, extracellular space) directly detectable from nuclear-based features in H&E slides, the relationship between information provided from T1C and nuclear-based features from H&E is not trivial.

Further research is needed to investigate on the association between radiomics features from T1C and pathomic features directly associable to this kind of images, such as those associated to microvessel density and quantitative perfusion maps (64).

After preliminary analysis for ADC, a deeper radiopathomic analysis was conducted to explore more detailed radiopathomic associations. In particular, considering two separate tasks for ADC and T1C images, we first investigated correlations between radiomic and pathomic features. It should be highlighted that we opted for analyzing the extracted radiomic and pathomic features filtered with a correlation filter to make the analyses more controllable and manageable and eliminate redundancies between the features. Furthermore, although we have not built predictive models, it should be considered that the elimination of highly correlated features is part of the main remedies against overfitting. On the other hand, we used a high threshold (ρ = 0.90) to lose as little information as possible after features drop out. In addition, this value was recommended as threshold by Peeters et al. (59) for redundancy filtering to perform factor analysis. Significant cross-scale associations were identified between pathomics features and ADC radiomic features, with correlation strength ranging from 0.45 to 0.74 in absolute value. Significant but fewer and with a lower upper bound ρ values were also found concerning the association between pathomics and radiomics features from T1C, with correlation strength ranging from 0.5 to 0.65 in absolute value. These results were corroborated by very large values of BF (ranging from 21.7 to 6.46×10^5^ for ADC task and from 22.9 to 2.58×10^5^ for T1C task) that gave strong evidence that the observed data supported the alternative hypothesis.

The radiopathomic analysis was enforced by a factor analysis aiming at establishing a compact representation of radiomic and pathomic features in terms of factors that retained most of the information contained in the full data set, using an approach dealing with both the high-dimensionality as well as the collinearity burden of feature sets (59). The correlation between resulting radiomic and pathomic factors revealed five significant cross-scale associations between pathomics and ADC factors (with correlation strength ranging from 0.51 to 0.54 in absolute value) and only two significant ρ values were found concerning the association between pathomics and T1C radiomics features (ρ = -0.63 and ρ = -0.53). Also results of correlation analysis between factors were corroborated by very large values of BF (ranging from 36.3 to 9.14×10^3^) that gave strong evidence that the observed data supported the alternative hypothesis.

While it is difficult to demonstrate a causal relationship between radiomics and pathomic features, we can hypothesize about the underlying connections between them.

As highlighted, the number of significant radiopathomic associations was almost four times higher when considering ADC than T1C features and more than half higher when considering ADC than T1C factors. This could be related to the quantitative information provided from ADC maps that is supposed to be more influenced by cellularity-associated properties extracted from the H&E images with respect to T1C. However, it is interesting to highlight the significant relationships identified between features associated with cell density and T1C radiomic features since this could provide info on the complementarity of diffusion and perfusion that are usually considered independent characteristics associated with the aggressiveness of the lesions and are independently assessed (65). Moreover, these results are in line with those from a recent study by Bobholz et al. (66) who found that multiparametric MR imaging intensity values, of which ADC and T1C, were associated with tumor cellularity. However, it should be highlighted that tumor cellularity was quantified on postmortem data, and any other pathomic feature than cellularity was evaluated in their study.

Another interesting point to highlight was that most significant radiopathomics associations involved textural features extracted from cell density maps, both considering ADC and T1C tasks. Concerning ADC correlation analysis, almost 1/3 correspond to associations with first order features and the remaining 2/3 were with textural features. Concerning T1C correlation analysis, almost 4/5 correspond to associations with firstorder features and the remaining 1/3 were with textural features. A low number of significant associations involved two intranuclear Haralick texture features (Haralick Angular Second Moment F0 and Information measure of correlation 2 F12) with ADC firstorder features (10 associations) and textural features (5 associations) and two T1C textural features (glcm Joint Energy and ngtdm Coarseness). These results were in line with those obtained in the factor analysis, in which all but one pathomic factors associated with ADC and T1C radiomics were mainly composed by features from cell density maps. The remaining one was mainly associated with nuclear circularity and eccentricity, and this could support the hypothesis that a link exists between specific aspects of tissue heterogeneity and parameters from diffusion MRI (67).

The prevalence of correlations involving features and factors mainly associated with features from cell-density maps highlights that the cell-density map-based feature extraction technique gave rise to novel interesting potential markers that could reflect macroscopic properties from radiologic images. Moreover, this could be intuitively related to the intrinsic meaning of these features that, arising from cell-density maps, are associated with a higher scale level than cellular/intranuclear features and are supposed to be closer to the macro-scale of radiomic features. In addition, it should be also highlighted that, among these significant results involving features from cell density maps, most of them involved features at lower resolutions (100µm, 150µm and 200 µm per pixel), that are supposed to be closer to the macro-scale of radiomic features with respect to those from 50µm resolution maps. On the other side, any of delaunay triangulation features, that describe inter-nuclear characteristics, showed significant correlations with radiomics.

Given the promising results involving features and factors associated with original images, we considered it appropriate to perform **Supplementary Analyses** by extracting the first and second order features following application of wavelet and local binary pattern (LBP) filters to the images. **Supplementary Analysis** results followed the trend of results obtained in the main analyses, with more significant results involving ADC than T1C pipeline and most of correlations involving features and factors mainly associated with features from cell-density maps. Of note, several wavelet and LBP features appeared in significant results both in correlation and factor analysis. Of note, we opted for investigating these two higher-order feature groups due to the wavelet and LBP features ability to decipher textural information from different scales (68, 69) and their power for texture analysis and classification (70). Moreover, these features have also been used for analyzing histopathological images (71, 72).

To our knowledge, this is the first study aiming at investigating radiopathomic associations between radiomic features extracted from ADC maps and T1C images and handcrafted pathomic features arising both from cell segmentations and cell density maps at different resolutions.

Only a few works have similarly correlated radiology and histopathology information, and efforts have been rather limited to establishing qualitative clinical correlation. In the context of brain tumors, Rathore et al. (31) aimed to assess the power of radiomic and pathomic features, both in comparison and in combination, for the prediction of survival in GBM patients from TCIA. They found that performances of machine learning models based on the combination of radiomic and pathomic features lead to better results than those based on radiomics and pathomic features taken separately. However, they have conducted any analysis to assess relationships between radiomic and pathomic features. Moreover, radiomic features from ADC were not evaluated. Bobholz et al. (22) examined the localized relationship between MR-based radiomic features from T1, T1C, FLAIR and ADC and their corresponding histomics features in patients with brain tumors. They found several significant radiopathomic associations (ρ > 0.2) and suggested that radiomic features were able to capture underlying histopathology. However, a direct comparison with our results was not possible due to differences in extracted features, statistical analyses, and study setting (e.g., pathomic features extracted from autopsy samples, non-specificity on GBM patients).

In the context of other cancer types, Shao et al. (30) found that the combination of radiomics and pathomics features was helpful in terms of pretreatment prediction of pathological response in patients with rectal cancer. Interestingly, they also investigated on the correlation between extracted radiomic and pathomic features before the construction of the predictive signatures. Alvarez-Jimenez et al. (34) identified significant cross-scale associations between CT radiomic features and pathomic features that independently showed discriminative power for differentiating between non-small cell lung cancer subtypes. It is worth noting that, similarly to what we did in our study, they examined pathomic features extracted from cell density maps at different resolutions. Of note, Lu et al. (23) performed a brief review on fusion of pathomics, radiomics and genomics, also providing an overview of research works combining radiomic and pathomics in the specific field of cancer prognosis.

Despite some promising results emerging in our preliminary study, there are still many limitations to overcome. First, despite utilizing a large public database, our final cohort size was still limited. However, we ensured our analysis was conducted as rigorously as possible. We attempted to account for these limited numbers by appropriately adjusting the FDR threshold. Another important limitation affecting our work concerns the missing information on the exact localization and orientation of the surgical sample. This certainly constitute a bias related to the lack of MRI-histology correspondence, but at the same time it could be an advantage as regards the generalizability of the approach developed in a clinical context since the dataset investigated could be representative of real-world data acquired in clinical practice. However, further studies performed on radiomics and pathomics data arising from colocalized MRI-pathology images are needed, although care should be taken concerning issues that can introduce bias when studying correlations between imaging and histological data. In particular, differences in orientation between the MRI scan planes and the surgical sample can determine a significant mismatch, the tissue deformation occurring when the histological sample is placed outside its anatomical background can determine important and locally nonlinear alignment inconsistencies, and the different spatial resolution of the two methods (1–5 mm for MR and 3–5 μm, for histology) does not allow accurate co-localization (26). However, we attempted to account for the missing MRI-histology co-localization bias as best as possible by proceeding to accurately avoid necrotic/cystic zones and large vessels in both macroscopic VOIs on MRI and microscopic segmentations on WSI images, in order to allow for multiscale quantification providing complementary information contributing to the understanding of tumor characteristics (73). In addition, we could not investigate the association between pathomic features and features deriving from quantitative parameters associated with perfusion (e.g. Ktrans, Kep, Ve, CBV) due to the non-availability of DCE/DSC images for almost all included patients (74). Furthermore, as previously mentioned, the features associated with cell density and intranuclear ones are not directly associable (regarding the functional meaning) to those of T1C. It would have been interesting to conduct preliminary analyses on microvessel cell density (MVD) to evaluate the associations between more advanced features associated with MVD segmentations and radiomic features from T1C (64, 75).

Moreover, we have not carried out survival analyses since the information on treatments that the patients have carried out were missing.

Of note, we opted for evaluating a limited number of features in this study in order to be able to directly translate descriptors from one scale to the next as done in our pathomic-radiomic analysis experiments. An essential criterion for having examined these hand-crafted features is that they are easier to interpret with respect to features obtained from neural networks (76, 77). However, our framework may be used to study associations between an expanded suite of radiomic and pathomic measurements, as well as extended to characterizing other cancers.

Another critical limitation affecting our study concern the well-known lack of shared reference standards concerning data storage, the missing agreement on analysis procedures, and the feature reliability and reproducibility limitations affecting both radiomics and pathomics (78). In particular, the existing lack of standardization in terms of image acquisition, processes, segmentation methods, and radiomics/pathomics analysis tools, could lead to discrepancies in feature measurements that are not due to underlying biological variations (79, 80). For example, differences in scanners and image acquisition parameters between considered patients may have affected radiomics results (81). In particular, it should be highlighted that MR images were acquired at different magnetic field strengths, and this represented a confounding factor, mainly for T1 and T2 images (82). However, it is worth noting that the stability of ADC radiomics features was found to be unaffected by differences in magnetic field strength, matching the field-independent nature of ADC (83). However, we normalized T1C raw images to account for the varying intensity ranges of MRI data and improve the robustness of radiomics features, as indicated by the IBSI guidelines (47). Moreover, we proceeded to report in detail all steps of radiomic and pathomic workflow performed in our study since it is essential to develop this emerging field in terms of clinical translation and to improve the reproducibility of study outcomes (15, 84).

Although our findings require careful interpretation due to the limitations mentioned above, the implemented radiopathomic approach revealed interesting cross-scale relationships between radiology and pathology in patients with GBM and may represent a starting point for future research on GBM. Our results strengthen the role of radiomics approach and its validation in clinical practice as “virtual biopsy”, introducing new insights for omics integration toward a personalized medicine approach. Further prospective and retrospective studies involving larger groups of patients are essential to validate obtained results, perform in-depth analyses and extend this approach to other cancer types.

## Data availability statement

The original contributions presented in the study are included in the article/**Supplementary Material**. Further inquiries can be directed to the corresponding author.

## Ethics statement

The studies involving human participants were reviewed and approved by Ethics Committee of the Istituto Nazionale Tumori “Fondazione G. Pascale. Written informed consent for participation was not required for this study in accordance with the national legislation and the institutional requirements.

## Author contributions

VB, CC and MA conceptualized the problem and determined the study aim and strategies. VB wrote the article, carried out the features extraction and the statistical analysis, and prepared figures and tables. NG collected the data and assisted in the preparation of figures and tables. CC assisted in the data collection and performed analysis and interpretation of patient data. MA and FI helped shape the analysis and the manuscript draft. MS, MA, FI, CC reviewed and edited the manuscript draft. MA and MS supervised the project. All authors contributed to the article and approved the submitted version.

## Funding

This work was supported by “Ricerca Corrente” Grant from Italian Ministry of Health (IRCCS SYNLAB SDN).

## Acknowledgments

Data used in this publication were generated by the National Cancer Institute Clinical Proteomic Tumor Analysis Consortium (CPTAC).

The authors gratefully acknowledge the Computational and Quantitative Biology (CQB) Program of the University of Naples Federico II for the support. We thank Prof. Michele Ceccarelli (CQB Program Chair) and Prof. Giuseppe Aceto (CQB PhD co-tutor of VB) who provided insight and expertise that greatly assisted the research.

## Conflict of interest

The authors declare that the research was conducted in the absence of any commercial or financial relationships that could be construed as a potential conflict of interest.

## Publisher’s note

All claims expressed in this article are solely those of the authors and do not necessarily represent those of their affiliated organizations, or those of the publisher, the editors and the reviewers. Any product that may be evaluated in this article, or claim that may be made by its manufacturer, is not guaranteed or endorsed by the publisher.
