# Supplementary material for: The relationship between radiomics and pathomics in Glioblastoma patients: Preliminary results from a cross-scale association study
Source: Front Oncol. 2022 Oct 6;12:1005805. doi: 10.3389/fonc.2022.1005805 (PMC9582951; doi:10.3389/fonc.2022.1005805)
Supplement: Supplementary file 1 [file DataSheet_1.pdf]

The relationship between radiomics and pathomics in Glioblastoma patients: preliminary results from a cross-scale association study

Valentina Brancato[1], Carlo Cavaliere[1,*], Nunzia Garbino[1], Francesco Isgrò[2], Marco Salvatore[1], Marco Aiello[1]

[1]IRCCS Synlab SDN, via E.Gianturco 113, 80143, Naples, Italy.

[2] Department of Electrical Engineering and Information Technologies, University of Napoli Federico II, Napoli, Italy

Supplementary Material

1 Features extracted

Table S1. Extracted radiomics features. The extracted radiomic features from ADC and T1+C images: First-order features including 18 intensity statistics; 73 multi-dimensional texture features including 23 Gray Level Co-occurrence Matrix (GLCM), 16 Gray Level Size Zone Matrix (GLSZM), 16 Gray Level Run Length Matrix (GLRLM), 14 Gray Level Dependence Matrix (GLDM) and 5 Neighboring Gray Tone Difference Matrix (NGTDM) Features.

**Feature group**	**Feature name**
First order (n = 18)	firstorder InterquartileRange
	firstorder Median
	firstorder Uniformity
	firstorder Range
	firstorder Entropy
	firstorder Minimum
	firstorder Kurtosis
	firstorder RootMeanSquared
	firstorder 90Percentile
	firstorder Energy
	firstorder Skewness
	firstorder Mean
	firstorder Variance

**Feature group**	**Feature name**
	firstorder RobustMeanAbsoluteDeviation
	firstorder MeanAbsoluteDeviation
	firstorder Maximum
	firstorder 10Percentile
Second order texture (n = 73)	glrlm RunLengthNonUniformity
	glrlm GrayLevelNonUniformityNormalized
	glrlm LongRunLowGrayLevelEmphasis
	glrlm HighGrayLevelRunEmphasis
	glrlm RunVariance
	glrlm RunLengthNonUniformityNormalized
	glrlm ShortRunEmphasis
	glrlm LongRunEmphasis
	glrlm LongRunHighGrayLevelEmphasis
	glrlm GrayLevelNonUniformity
	glrlm ShortRunHighGrayLevelEmphasis
	glrlm ShortRunLowGrayLevelEmphasis
	glrlm RunPercentage
	glrlm GrayLevelVariance
	glrlm RunEntropy
	glrlm LowGrayLevelRunEmphasis
	ngtdm Coarseness
	ngtdm Complexity
	ngtdm Busyness
	ngtdm Strength
	ngtdm Contrast
	glcm JointEntropy
	glcm ClusterShade
	glcm ClusterProminence
	glcm Autocorrelation
	glcm SumSquares
	glcm SumEntropy

Feature group	Feature name
	glcm DifferenceVariance
	glcm Idmn
	glcm Imc2
	glcm Imc1
	glcm DifferenceAverage
	glcm JointAverage
	glcm InverseVariance
	glcm DifferenceEntropy
	glcm Idm
	glcm Idn
	glcm Correlation
	glcm MCC
	glcm ClusterTendency
	glcm Contrast
	glcm Id
	glcm MaximumProbability
	glcm JointEnergy
	gldm SmallDependenceHighGrayLevelEmphasis
	gldm LargeDependenceLowGrayLevelEmphasis
	gldm DependenceNonUniformity
	gldm LargeDependenceHighGrayLevelEmphasis
	gldm DependenceNonUniformityNormalized
	gldm LowGrayLevelEmphasis
	gldm LargeDependenceEmphasis
	gldm SmallDependenceEmphasis
	gldm DependenceEntropy
	gldm DependenceVariance
	gldm SmallDependenceLowGrayLevelEmphasis
	gldm GrayLevelNonUniformity
	gldm HighGrayLevelEmphasis
	gldm GrayLevelVariance
	glszm GrayLevelNonUniformityNormalized
	glszm SmallAreaHighGrayLevelEmphasis

Feature group	Feature name
	glszm GrayLevelVariance
	glszm LargeAreaEmphasis
	glszm SizeZoneNonUniformity
	glszm LargeAreaLowGrayLevelEmphasis
	glszm HighGrayLevelZoneEmphasis
	glszm ZonePercentage
	glszm ZoneVariance
	glszm GrayLevelNonUniformity
	glszm LargeAreaHighGrayLevelEmphasis
	glszm SizeZoneNonUniformityNormalized
	glszm SmallAreaEmphasis
	glszm ZoneEntropy
	glszm SmallAreaLowGrayLevelEmphasis
	glszm LowGrayLevelZoneEmphasis

Table S2. Extracted pathomic features. The extracted pathomic features from detection measurements (shape characteristics, intranuclear Haralick texture features, Delaunay triangulation) and cell-density maps features including 18 firstorder intensity statistics and 73 multi-dimensional texture features including 23 Gray Level Co-occurrence Matrix (GLCM), 16 Gray Level Size Zone Matrix (GLSZM), 16 Gray Level Run Length Matrix (GLRLM), 14 Gray Level Dependence Matrix (GLDM) and 5 Neighboring Gray Tone Difference Matrix (NGTDM) Features.

Feature group	Feature name
Detection measurements	MEAN Nucleus Area
	MEAN Nucleus Perimeter
	MEAN Nucleus Circularity
	MEAN Nucleus MaxCaliper
	MEAN Nucleus MinCaliper
	MEAN Nucleus Eccentricity
	MEAN Nucleus HematoxylinODMean
	MEAN Nucleus HematoxylinODSum
	MEAN Nucleus HematoxylinODStdDev
	MEAN Nucleus HematoxylinODMax

**Feature group**	**Feature name**
	MEAN Nucleus HematoxylinODMin
	MEAN Nucleus HematoxylinODRange
	MEAN Nucleus EosinODMean
	MEAN Nucleus EosinODSum
	MEAN Nucleus EosinODStdDev
	MEAN Nucleus EosinODMax
	MEAN Nucleus EosinODMin
	MEAN Nucleus EosinODRange
	MEAN Cell Area
	MEAN Cell Perimeter
	MEAN Cell Circularity
	MEAN Cell MaxCaliper
	MEAN Cell MinCaliper
	MEAN Cell Eccentricity
	MEAN Cell HematoxylinODMean
	MEAN Cell HematoxylinODStdDev
	MEAN Cell HematoxylinODMax
	MEAN Cell HematoxylinODMin
	MEAN Cell EosinODMean
	MEAN Cell EosinODStdDev
	MEAN Cell EosinODMax
	MEAN Cell EosinODMin
	MEAN Cytoplasm HematoxylinODMean
	MEAN Cytoplasm HematoxylinODStdDev
	MEAN Cytoplasm HematoxylinODMax
	MEAN Cytoplasm HematoxylinODMin
	MEAN Cytoplasm EosinODMean
	MEAN Cytoplasm EosinODStdDev
	MEAN Cytoplasm EosinODMax
	MEAN Cytoplasm EosinODMin
	MEAN Nucleus CellAreaRatio
	MEAN Delaunay MeanDistance
	MEAN Delaunay MedianDistance

**Feature group**	**Feature name**
	MEAN Delaunay MaxDistance
	MEAN Delaunay MinDistance
	MEAN Delaunay MeanTriangleArea
	MEAN Delaunay MaxTriangleArea
	MEAN ODSum Mean
	MEAN ODSum Std dev
	MEAN ODSum Min
	MEAN ODSum Max
	MEAN ODSum Median
	MEAN ODSum HaralickAngularSecondMoment F0
	MEAN ODSum HaralickContrast F1
	MEAN ODSum HaralickCorrelation F2
	MEAN ODSum HaralickSumOfSquares F3
	MEAN ODSum HaralickInverseDifferenceMomen
	MEAN ODSum HaralickSumAverage F5
	MEAN ODSum HaralickSumVariance F6
	MEAN ODSum HaralickSumEntropy F7
	MEAN ODSum HaralickEntropy F8
	MEAN ODSum HaralickDifferenceVariance F9
	MEAN ODSum HaralickDifferenceEntropy F10
	MEAN ODSum HaralickInformationMeasureOfCorrelation 1 F11
	MEAN ODSum HaralickInformationMeasureOfCorrelation 2 F12
Cell-density map-based (first-order)	firstorder InterquartileRange
	firstorder Median
	firstorder Uniformity
	firstorder Range
	firstorder Entropy
	firstorder Minimum
	firstorder Kurtosis
	firstorder RootMeanSquared

**Feature group**	**Feature name**
	firstorder 90Percentile
	firstorder Energy
	firstorder Skewness
	firstorder Mean
	firstorder Variance
	firstorder RobustMeanAbsoluteDeviation
	firstorder MeanAbsoluteDeviation
	firstorder Maximum
	firstorder 10Percentile
	glrlm RunLengthNonUniformity
	glrlm GrayLevelNonUniformityNormalized
	glrlm LongRunLowGrayLevelEmphasis
	glrlm HighGrayLevelRunEmphasis
	glrlm RunVariance
	glrlm RunLengthNonUniformityNormalized
	glrlm ShortRunEmphasis
	glrlm LongRunEmphasis
	glrlm LongRunHighGrayLevelEmphasis
	glrlm GrayLevelNonUniformity
	glrlm ShortRunHighGrayLevelEmphasis
	glrlm ShortRunLowGrayLevelEmphasis
	glrlm RunPercentage
	glrlm GrayLevelVariance
	glrlm RunEntropy
	glrlm LowGrayLevelRunEmphasis
	ngtdm Coarseness
	ngtdm Complexity
	ngtdm Busyness
	ngtdm Strength
	ngtdm Contrast
	glcm JointEntropy
	glcm ClusterShade
	glcm ClusterProminence

**Feature group**	**Feature name**
	glcm Autocorrelation
	glcm SumSquares
	glcm SumEntropy
	glcm DifferenceVariance
	glcm Idmn
	glcm Imc2
	glcm Imc1
	glcm DifferenceAverage
	glcm JointAverage
	glcm InverseVariance
	glcm DifferenceEntropy
	glcm Idm
	glcm Idn
	glcm Correlation
	glcm MCC
	glcm ClusterTendency
	glcm Contrast
	glcm Id
	glcm MaximumProbability
	glcm JointEnergy
	gldm SmallDependenceHighGrayLevelEmphasis
	gldm LargeDependenceLowGrayLevelEmphasis
	gldm DependenceNonUniformity
	gldm LargeDependenceHighGrayLevelEmphasis
	gldm DependenceNonUniformityNormalized
	gldm LowGrayLevelEmphasis
	gldm LargeDependenceEmphasis
	gldm SmallDependenceEmphasis
	gldm DependenceEntropy
	gldm DependenceVariance

Feature group	Feature name
	gldm SmallDependenceLowGrayLevelEmphasis
	gldm GrayLevelNonUniformity
	gldm HighGrayLevelEmphasis
	gldm GrayLevelVariance
	glszm GrayLevelNonUniformityNormalized
	glszm SmallAreaHighGrayLevelEmphasis
	glszm GrayLevelVariance
	glszm LargeAreaEmphasis
	glszm SizeZoneNonUniformity
	glszm LargeAreaLowGrayLevelEmphasis
	glszm HighGrayLevelZoneEmphasis
	glszm ZonePercentage
	glszm ZoneVariance
	glszm GrayLevelNonUniformity
	glszm LargeAreaHighGrayLevelEmphasis
	glszm SizeZoneNonUniformityNormalized
	glszm SmallAreaEmphasis
	glszm ZoneEntropy
	glszm SmallAreaLowGrayLevelEmphasis
	glszm LowGrayLevelZoneEmphasis

2 Summary of the significantly moderately correlated radiomic-pathomic features

Table S3 Summary of the significantly moderately correlated radiomic-pathomic features, with radiomic features extracted from ADC. Abbreviations: CD = Cellular Density; ADC = Apparent Diffusion Coefficient; LALGLE = Large Area Low Gray Level Emphasis; IMOC = Information Measure Of Correlation; ASM = Angular Second Moment; LDLGLE = Large Dependence Low Gray Level Emphasis; LDHGLE = Large Dependence High Gray Level Emphasis; SDLGLE = Small Dependence Low Gray Level Emphasis; LALGLE = Large Area Low Gray Level Emphasis; GLNUN = Gray level non uniformity normalized; glcm = gray level co-occurrence matrix; gldm = Gray Level Dependence Matrix; glszm = Gray Level Size Zone Matrix; ngtdm = Neighbouring Gray Tone Difference Matrix; glrlm = Gray Level Run Length Matrix.

Radiomic ADC feature name	Pathomic feature name	ρ	Q-value	BF
ADC glcm ClusterShade	MEAN Haralick ASM F0	0.589	$1.66{\times}10^{-3}$	$1.73{\times}10^{3}$
ADC glcm Imc2	CD100µm glrlm LongRunEmphasis	-0.589	$1.67{\times}10^{-3}$	$1.68{\times}10^{3}$
ADC firstorder Maximum	CD200µm ngtdm Strength	0.588	$1.71{\times}10^{-3}$	$1.62{\times}10^{3}$

Radiomic ADC feature name	Pathomic feature name	ρ	Q-value	BF
ADC firstorder 90Percentile	MEAN Haralick IMOC F12	-0.586	1.83×10^{-3}	1.49×10^{3}
ADC glcm DifferenceAverage	MEAN Haralick ASM F0	0.585	1.84×10^{-3}	1.46×10^{3}
ADC glcm DifferenceVariance	CD100µm ngtdm Strength	0.584	1.88×10^{-3}	1.40×10^{3}
ADC glcm InverseVariance	CD150µm glszm LALGLE	0.582	1.99×10^{-3}	1.31×10^{3}
ADC firstorder 90Percentile	CD100µm ngtdm Strength	0.579	2.26×10^{-3}	1.14×10^{3}
ADC gldm DependenceVariance	CD100µm glrlm LongRunEmphasis	0.578	2.34×10^{-3}	1.09×10^{3}
ADC gldm SDLGLE	MEAN Haralick IMOC F12	-0.577	2.42×10^{-3}	1.04×10^{3}
ADC glcm ClusterProminence	CD200µm ngtdm Strength	0.573	2.72×10^{-3}	9.15×10^{2}
ADC ngtdm Complexity	CD150µm glcm Contrast	0.573	2.76×10^{-3}	8.89×10^{2}
ADC ngtdm Complexity	CD50µm glcm DifferenceVariance	0.572	2.82×10^{-3}	8.56×10^{2}
ADC gldm SDLGLE	CD200µm ngtdm Strength	0.57	2.91×10^{-3}	8.19×10^{2}
ADC firstorder TotalEnergy	CD50µm glcm DifferenceVariance	0.564	3.67×10^{-3}	6.50×10^{2}
ADC glrlm RunVariance	CD100µm glrlm LongRunEmphasis	0.563	3.73×10^{-3}	6.30×10^{2}
ADC firstorder 10Percentile	CD150µm ngtdm Strength	0.558	4.55×10^{-3}	5.15×10^{2}
ADC glcm ClusterProminence	CD50µm glcm DifferenceVariance	0.546	7.04×10^{-3}	3.37×10^{2}
ADC ngtdm Strength	MEAN Haralick ASM F0	0.543	7.74×10^{-3}	3.01×10^{2}
ADC firstorder Median	CD150µm ngtdm Strength	0.543	7.74×10^{-3}	3.00×10^{2}
ADC firstorder Minimum	CD150µm ngtdm Strength	0.542	7.88×10^{-3}	2.92×10^{2}
ADC glszm GLNUN	CD100µm glrlm LongRunEmphasis	0.541	7.93×10^{-3}	2.83×10^{2}
ADC firstorder TotalEnergy	CD150µm glcm Contrast	0.541	7.93×10^{-3}	2.82×10^{2}
ADC glcm InverseVariance	CD100µm glrlm LongRunEmphasis	0.539	8.34×10^{-3}	2.66×10^{2}
ADC ngtdm Complexity	CD50µm ngtdm Complexity	0.539	8.45×10^{-3}	2.59×10^{2}
ADC firstorder Mean	CD150µm ngtdm Strength	0.535	9.40×10^{-3}	2.32×10^{2}
ADC glcm ClusterTendency	CD50µm ngtdm Complexity	0.531	1.07×10^{-2}	2.02×10^{2}
ADC glcm ClusterProminence	CD50µm glcm Contrast	0.531	1.07×10^{-2}	2.00×10^{2}
ADC firstorder Range	CD50µm ngtdm Complexity	0.53	1.09×10^{-2}	1.95×10^{2}
ADC firstorder TotalEnergy	CD50µm glcm DifferenceAverage	0.53	1.09×10^{-2}	1.92×10^{2}

**Radiomic ADC feature name**	**Pathomic feature name**	**ρ**	**Q-value**	**BF**
ADC firstorder Maximum	MEAN Haralick IMOC2 F12	-0.525	1.27×10^{-2}	1.65×10^{2}
ADC glcm DifferenceAverage	CD200μm ngtdm Strength	0.524	1.28×10^{-2}	1.61×10^{2}
ADC glcm ClusterTendency	CD50μm glcm Contrast	0.524	1.30×10^{-2}	1.57×10^{2}
ADC glcm ClusterTendency	CD50μm glcm DifferenceVariance	0.523	1.32×10^{-2}	1.53×10^{2}
ADC glcm ClusterProminence	MEAN Haralick IMOC2 F12	-0.522	1.35×10^{-2}	1.48×10^{2}
ADC glcm ClusterTendency	MEAN Haralick ASM F0	0.521	1.37×10^{-2}	1.45×10^{2}
ADC glcm ClusterShade	CD200μm ngtdm Strength	0.519	1.44×10^{-2}	1.37×10^{2}
ADC ngtdm Contrast	MEAN HaralickDifferenceVariance F9	0.519	1.46×10^{-2}	1.34×10^{2}
ADC glcm ClusterProminence	CD100μm ngtdm Strength	0.518	1.46×10^{-2}	1.32×10^{2}
ADC ngtdm Strength	CD50μm ngtdm Complexity	0.518	1.48×10^{-2}	1.29×10^{2}
ADC ngtdm Complexity	CD50μm glcm DifferenceAverage	0.517	1.49×10^{-2}	1.28×10^{2}
ADC firstorder Maximum	CD100μm ngtdm Strength	0.516	1.52×10^{-2}	1.23×10^{2}
ADC glcm MaximumProbability	CD150μm glszm LALGLE	0.516	1.52×10^{-2}	1.22×10^{2}
ADC glcm MaximumProbability	CD100μm glrlm RunVariance	0.515	1.53×10^{-2}	1.20×10^{2}
ADC firstorder MeanAbsoluteDeviation	CD50μm ngtdm Complexity	0.515	1.53×10^{-2}	1.19×10^{2}
ADC firstorder InterquartileRange	CD50μm ngtdm Complexity	0.515	1.53×10^{-2}	1.18×10^{2}
ADC glcm ClusterShade	MEAN Haralick IMOC2 F12	-0.514	1.57×10^{-2}	1.14×10^{2}
ADC ngtdm Coarseness	CD200μm glcm JointEnergy	0.512	1.62×10^{-2}	1.10×10^{2}
ADC firstorder Range	CD50μm glcm Contrast	0.511	1.66×10^{-2}	1.07×10^{2}
ADC ngtdm Complexity	CD100μm glcm Contrast	0.51	1.70×10^{-2}	1.03×10^{2}
ADC firstorder TotalEnergy	CD100μm glcm Contrast	0.51	1.70×10^{-2}	1.02×10^{2}
ADC firstorder Minimum	MEAN HaralickDifferenceVariance F9	0.51	1.71×10^{-2}	1.01×10^{2}
ADC glcm DifferenceVariance	MEAN HaralickDifferenceVariance F9	0.509	1.73×10^{-2}	9.89×10^{1}
ADC glcm DifferenceAverage	MEAN Haralick IMOC2 F12	-0.508	1.73×10^{-2}	9.72×10^{1}
ADC gldm SDLGLE	MEAN Haralick ASM F0	0.508	1.73×10^{-2}	9.71×10^{1}
ADC firstorder MeanAbsoluteDeviation	MEAN Haralick ASM F0	0.508	1.75×10^{-2}	9.50×10^{1}
ADC glcm ClusterShade	CD50μm glcm DifferenceVariance	0.507	1.76×10^{-2}	9.30×10^{1}
ADC firstorder 90Percentile	CD150μm ngtdm Strength	0.507	1.76×10^{-2}	9.28×10^{1}
ADC ngtdm Contrast	CD150μm ngtdm Strength	0.507	1.77×10^{-2}	9.17×10^{1}

**Radiomic ADC feature name**	**Pathomic feature name**	**ρ**	**Q-value**	**BF**
ADC ngtdm Complexity	CD150µm glcm DifferenceAverage	0.502	2.02×10^{-2}	8.05×10^{1}
ADC firstorder Maximum	CD50µm glcm DifferenceVariance	0.501	2.06×10^{-2}	7.84×10^{1}
ADC glszm ZoneVariance	CD200µm glrlm RunVariance	0.5	2.13×10^{-2}	7.54×10^{1}
ADC firstorder 10Percentile	MEAN HaralickDifferenceVariance F9	0.5	2.13×10^{-2}	7.47×10^{1}
ADC ngtdm Contrast	CD200µm glcm ClusterShade	0.498	2.23×10^{-2}	7.09×10^{1}
ADC gldm LDLGLE	CD200µm glrlm RunVariance	0.497	2.25×10^{-2}	6.94×10^{1}
ADC firstorder Minimum	MEAN HaralickEntropy F8	-0.497	2.25×10^{-2}	6.90×10^{1}
ADC firstorder 10Percentile	MEAN HaralickEntropy F8	-0.497	2.25×10^{-2}	6.84×10^{1}
ADC glszm LALGLE	CD200µm glrlm RunVariance	0.497	2.25×10^{-2}	6.81×10^{1}
ADC firstorder Median	MEAN HaralickEntropy F8	-0.497	2.25×10^{-2}	6.76×10^{1}
ADC ngtdm Busyness	CD200µm glrlm RunVariance	0.496	2.29×10^{-2}	6.59×10^{1}
ADC glcm ClusterShade	CD50µm ngtdm Complexity	0.495	2.31×10^{-2}	6.50×10^{1}
ADC glcm ClusterShade	CD50µm glcm Contrast	0.494	2.38×10^{-2}	6.28×10^{1}
ADC glcm DifferenceVariance	CD150µm ngtdm Strength	0.494	2.38×10^{-2}	6.22×10^{1}
ADC glcm ClusterProminence	CD150µm glcm DifferenceVariance	0.492	2.49×10^{-2}	5.92×10^{1}
ADC firstorder Mean	MEAN HaralickEntropy F8	-0.492	2.50×10^{-2}	5.85×10^{1}
ADC glcm DifferenceVariance	MEAN HaralickEntropy F8	-0.491	2.55×10^{-2}	5.71×10^{1}
ADC glcm ClusterProminence	CD150µm glcm Contrast	0.49	2.56×10^{-2}	5.64×10^{1}
ADC firstorder Median	MEAN HaralickDifferenceVariance F9	0.49	2.57×10^{-2}	5.59×10^{1}
ADC firstorder TotalEnergy	CD150µm glcm DifferenceAverage	0.489	2.64×10^{-2}	5.40×10^{1}
ADC ngtdm Coarseness	CD150µm glcm JointEnergy	0.488	2.67×10^{-2}	5.31×10^{1}
ADC firstorder Mean	MEAN HaralickDifferenceVariance F9	0.488	2.69×10^{-2}	5.25×10^{1}
ADC firstorder InterquartileRange	CD50µm glcm Contrast	0.487	2.71×10^{-2}	5.12×10^{1}
ADC gldm DependenceVariance	CD200µm glrlm RunVariance	0.487	2.71×10^{-2}	5.10×10^{1}
ADC firstorder Maximum	CD50µm glcm Contrast	0.487	2.71×10^{-2}	5.10×10^{1}
ADC glcm ClusterProminence	MEAN HaralickEntropy F8	-0.486	2.73×10^{-2}	5.03×10^{1}
ADC glcm DifferenceVariance	CD200µm glcm ClusterShade	0.486	2.76×10^{-2}	4.95×10^{1}

**Radiomic ADC feature name**	**Pathomic feature name**	**ρ**	**Q-value**	**BF**
ADC firstorder Range	CD50µm glcm DifferenceVariance	0.485	2.80×10^{-2}	4.84×10^{1}
ADC firstorder InterquartileRange	CD50µm glcm DifferenceVariance	0.484	2.83×10^{-2}	4.76×10^{1}
ADC ngtdm Complexity	CD100µm glcm DifferenceVariance	0.484	2.85×10^{-2}	4.70×10^{1}
ADC firstorder TotalEnergy	CD50µm ngtdm Complexity	0.482	3.03×10^{-2}	4.42×10^{1}
ADC glcm ClusterProminence	CD150µm ngtdm Strength	0.481	3.08×10^{-2}	4.29×10^{1}
ADC glcm Imc2	CD200µm glrlm RunVariance	-0.481	3.08×10^{-2}	4.29×10^{1}
ADC firstorder InterquartileRange	MEAN Haralick ASM F0	0.48	3.11×10^{-2}	4.22×10^{1}
ADC firstorder MeanAbsoluteDeviation	CD50µm glcm DifferenceVariance	0.48	3.12×10^{-2}	4.19×10^{1}
ADC firstorder MeanAbsoluteDeviation	CD50µm glcm Contrast	0.479	3.16×10^{-2}	4.11×10^{1}
ADC firstorder 90Percentile	MEAN HaralickEntropy F8	-0.478	3.25×10^{-2}	3.98×10^{1}
ADC firstorder 90Percentile	CD50µm glcm DifferenceVariance	0.475	3.54×10^{-2}	3.66×10^{1}
ADC firstorder 90Percentile	MEAN HaralickDifferenceVariance F9	0.475	3.55×10^{-2}	3.62×10^{1}
ADC firstorder Median	CD50µm glcm DifferenceVariance	0.474	3.60×10^{-2}	3.56×10^{1}
ADC firstorder TotalEnergy	CD100µm glcm DifferenceVariance	0.474	3.62×10^{-2}	3.51×10^{1}
ADC gldm LDHGLE	CD50µm glcm Contrast	0.473	3.65×10^{-2}	3.47×10^{1}
ADC firstorder Mean	CD50µm glcm DifferenceVariance	0.472	3.81×10^{-2}	3.31×10^{1}
ADC firstorder 10Percentile	CD200µm glcm ClusterShade	0.47	3.94×10^{-2}	3.19×10^{1}
ADC ngtdm Contrast	MEAN HaralickEntropy F8	-0.47	3.97×10^{-2}	3.15×10^{1}
ADC ngtdm Complexity	CD150µm glcm DifferenceVariance	0.468	4.09×10^{-2}	3.05×10^{1}
ADC glcm DifferenceAverage	CD50µm glcm DifferenceVariance	0.468	4.12×10^{-2}	3.01×10^{1}
ADC ngtdm Strength	MEAN Haralick IMOC2 F12	-0.467	4.15×10^{-2}	2.95×10^{1}
ADC glcm ClusterProminence	CD50µm ngtdm Complexity	0.467	4.15×10^{-2}	2.95×10^{1}
ADC firstorder 10Percentile	CD50µm ngtdm Strength	0.467	4.15×10^{-2}	2.93×10^{1}
ADC glcm ClusterShade	CD100µm ngtdm Strength	0.467	4.15×10^{-2}	2.92×10^{1}
ADC gldm LDHGLE	CD200µm ngtdm Strength	0.466	4.24×10^{-2}	2.85×10^{1}
ADC firstorder Mean	CD200µm glcm ClusterShade	0.465	4.35×10^{-2}	2.75×10^{1}
ADC glcm DifferenceAverage	CD50µm ngtdm Complexity	0.465	4.35×10^{-2}	2.75×10^{1}
ADC glcm ClusterProminence	CD100µm glcm DifferenceVariance	0.464	4.35×10^{-2}	2.73×10^{1}
ADC firstorder Range	CD50µm glcm DifferenceAverage	0.463	4.44×10^{-2}	2.65×10^{1}

Radiomic ADC feature name	Pathomic feature name	ρ	Q-value	BF
ADC glrlm RunVariance	CD200µm glrlm RunVariance	0.463	4.44×10^{-2}	2.65×10^{1}
ADC gldm LDHGLE	MEAN Haralick ASM F0	0.463	4.44×10^{-2}	2.63×10^{1}
ADC firstorder 90Percentile	CD200µm glcm ClusterShade	0.463	4.44×10^{-2}	2.62×10^{1}
ADC firstorder Median	CD200µm glcm ClusterShade	0.463	4.45×10^{-2}	2.60×10^{1}
ADC firstorder TotalEnergy	CD100µm glcm DifferenceAverage	0.462	4.56×10^{-2}	2.53×10^{1}
ADC glcm DifferenceAverage	CD100µm ngtdm Strength	0.461	4.67×10^{-2}	2.46×10^{1}
ADC ngtdm Coarseness	CD50µm glcm Idmn	-0.459	4.78×10^{-2}	2.39×10^{1}
ADC glcm JointAverage	CD50µm glcm Contrast	0.459	4.78×10^{-2}	2.36×10^{1}
ADC ngtdm Complexity	MEAN Cytoplasm EosinODMin	0.459	4.78×10^{-2}	2.36×10^{1}
ADC firstorder 10Percentile	MEAN HaralickSumEntropy F7	-0.458	4.78×10^{-2}	2.33×10^{1}
ADC glcm ClusterShade	MEAN HaralickEntropy F8	-0.458	4.78×10^{-2}	2.33×10^{1}
ADC glcm MaximumProbability	MEAN Haralick IMOC2 F12	-0.458	4.78×10^{-2}	2.33×10^{1}
ADC firstorder Median	MEAN HaralickSumEntropy F7	-0.458	4.78×10^{-2}	2.32×10^{1}
ADC glcm SumEntropy	MEAN Haralick IMOC2 F12	0.458	4.78×10^{-2}	2.31×10^{1}
ADC firstorder Minimum	MEAN HaralickSumEntropy F7	-0.458	4.79×10^{-2}	2.29×10^{1}
ADC firstorder Maximum	CD150µm ngtdm Strength	0.457	4.81×10^{-2}	2.27×10^{1}
ADC firstorder Minimum	CD50µm ngtdm Strength	0.457	4.81×10^{-2}	2.27×10^{1}
ADC glcm DifferenceAverage	CD50µm glcm Contrast	0.457	4.85×10^{-2}	2.24×10^{1}
ADC glcm ClusterProminence	MEAN HaralickDifferenceVariance F9	0.456	4.94×10^{-2}	2.17×10^{1}
ADC firstorder Maximum	CD50µm ngtdm Complexity	0.456	4.94×10^{-2}	2.17×10^{1}
ADC glcm ClusterShade	MEAN HaralickDifferenceVariance F9	0.456	4.94×10^{-2}	2.17×10^{1}

Table S4 Summary of the significantly moderately correlated radiomic-pathomic features, with radiomic features extracted from T1C. Abbreviations: DNU = Dependence non uniformity; GLNU = gray-level non-uniformity; IMOC = Information Measure Of Correlation; ASM = Angular Second Moment; SRLGLE = Short Run Low Gray Level Emphasis; SDLGLE = Small Dependence Low Gray Level Emphasis; T1C = post-contrast T1; SALGLE = Small Area Low Gray Level Emphasis; LALGLE = Large Area Low Gray Level Emphasis; glcm = gray level co-occurrence matrix; gldm = Gray Level Dependence Matrix; glszm = Gray Level Size Zone Matrix; ngtdm = Neighbouring Gray Tone Difference Matrix; glrlm = Gray Level Run Length Matrix.

Radiomic T1C feature name	Pathomic feature name	ρ	FDR q-value	BF
T1C glszm SALGLE	CD200μm ngtdm Strength	0.599	4.44×10^{-3}	2.59×10^{3}
T1C glcm JointEnergy	MEAN Haralick ASM F0	0.597	4.66×10^{-3}	2.33×10^{3}
T1C firstorder RootMeanSquared	CD150μm gldm DNU	-0.594	4.91×10^{-3}	2.11×10^{3}
T1C glszm SALGLE	MEAN Haralick IMOC2 F12	-0.591	5.27×10^{-3}	1.86×10^{3}
T1C glcm MaximumProbability	MEAN Haralick IMOC2 F12	-0.59	5.27×10^{-3}	1.78×10^{3}
T1C glszm SALGLE	MEAN Haralick ASM F0	0.586	5.63×10^{-3}	1.54×10^{3}
T1C firstorder 10Percentile	CD150μm gldm DNU	-0.586	5.63×10^{-3}	1.53×10^{3}
T1C firstorder 90Percentile	CD150μm glcm Imc2	0.578	7.49×10^{-3}	1.11×10^{3}
T1C firstorder 10Percentile	CD200μm glcm Imc2	0.577	7.49×10^{-3}	1.05×10^{3}
T1C gldm SDLGLE	MEAN Haralick IMOC2 F12	-0.576	7.49×10^{-3}	1.03×10^{3}
T1C gldm SDLGLE	MEAN Haralick ASM F0	0.576	7.49×10^{-3}	9.98×10^{2}
T1C glcm MaximumProbability	CD200μm ngtdm Strength	0.572	8.45×10^{-3}	8.59×10^{2}
T1C glrlm SRLGLE	CD200μm ngtdm Strength	0.57	8.52×10^{-3}	8.15×10^{2}
T1C firstorder 10Percentile	CD100μm ngtdm Busyness	-0.57	8.52×10^{-3}	7.97×10^{2}
T1C firstorder RootMeanSquared	CD100μm ngtdm Busyness	-0.569	8.56×10^{-3}	7.69×10^{2}
T1C firstorder 10Percentile	CD150μm glcm Imc2	0.562	1.01×10^{-2}	6.00×10^{2}
T1C firstorder 90Percentile	CD150μm gldm DNU	-0.562	1.01×10^{-2}	5.96×10^{2}
T1C glrlm SRLGLE	MEAN Haralick IMOC2 F12	-0.561	1.01×10^{-2}	5.78×10^{2}
T1C firstorder 10Percentile	CD200μm ngtdm Strength	0.561	1.01×10^{-2}	5.78×10^{2}
T1C firstorder Minimum	CD200μm ngtdm Strength	0.561	1.01×10^{-2}	5.70×10^{2}
T1C glrlm SRLGLE	MEAN Haralick ASM F0	0.554	1.26×10^{-2}	4.49×10^{2}
T1C glcm MaximumProbability	MEAN Haralick ASM F0	0.545	1.76×10^{-2}	3.20×10^{2}
T1C firstorder Kurtosis	CD150μm gldm DNU	0.539	2.09×10^{-2}	2.66×10^{2}
T1C ngtdm Coarseness	CD100μm ngtdm Strength	0.537	2.21×10^{-2}	2.42×10^{2}
T1C glcm Idn	CD200μm ngtdm Strength	-0.536	2.21×10^{-2}	2.40×10^{2}
T1C firstorder RootMeanSquared	CD200μm firstorder TotalEnergy	-0.533	2.40×10^{-2}	2.14×10^{2}
T1C firstorder 10Percentile	CD200μm firstorder TotalEnergy	-0.533	2.40×10^{-2}	2.13×10^{2}
T1C glszm SALGLE	CD100μm ngtdm Strength	0.531	2.50×10^{-2}	2.00×10^{2}
T1C gldm SDLGLE	CD100μm ngtdm Strength	0.529	2.62×10^{-2}	1.87×10^{2}

Radiomic T1C feature name	Pathomic feature name	ρ	FDR q-value	BF
T1C glszm LALGLE	CD200µm glcm Imc2	-0.525	2.91×10^{-2}	1.67×10^{2}
T1C firstorder 90Percentile	CD100µm ngtdm Busyness	-0.523	3.09×10^{-2}	1.54×10^{2}
T1C glcm InverseVariance	CD150µm glszm GLNU	0.522	3.10×10^{-2}	1.48×10^{2}
T1C glcm InverseVariance	CD200µm glcm Imc2	-0.522	3.10×10^{-2}	1.48×10^{2}
T1C firstorder Kurtosis	CD150µm glszm GLNU	0.516	3.64×10^{-2}	1.25×10^{2}
T1C glcm JointEnergy	CD100µm ngtdm Strength	0.514	3.87×10^{-2}	1.16×10^{2}
T1C glcm InverseVariance	CD50µm ngtdm Busyness	0.51	4.32×10^{-2}	1.03×10^{2}
T1C glszm LALGLE	CD150µm glcm Imc2	-0.508	4.59×10^{-2}	9.51×10^{1}
T1C gldm DependenceVariance	CD150µm glszm GLNU	0.506	4.70×10^{-2}	9.13×10^{1}

3 Factor analysis

Table S5 ADC factor loadings for the five ADC factors. A darker grey in the column cell correspond to a higher loadings measuring the association between features and factors. Abbreviations: ADC = Apparent Diffusion Coefficient; LALGLE = Large Area Low Gray Level Emphasis; LDLGLE = Large Dependence Low Gray Level Emphasis; LDHGLE = Large Dependence High Gray Level Emphasis; SDLGLE = Small Dependence Low Gray Level Emphasis; LALGLE = Large Area Low Gray Level Emphasis; GLNUN = Gray level non uniformity normalized; SZNU = Size Zone Non-Uniformity; glcm = gray level co-occurrence matrix; gldm = Gray Level Dependence Matrix; glszm = Gray Level Size Zone Matrix; ngtdm = Neighbouring Gray Tone Difference Matrix; glrlm = Gray Level Run Length Matrix; F = Factor.

Radiomic ADC feature	*ADC_F1*	*ADC_F2*	*ADC_F3*	*ADC_F4*	*ADC_F5*
ADC firstorder MeanAbsoluteDeviation	0.857	-0.106	-0.114	0.241	-0.016
ADC firstorder Maximum	0.902	-0.088	-0.029	-0.004	0.002
ADC firstorder Minimum	0.814	-0.03	-0.137	-0.309	-0.068
ADC firstorder Kurtosis	-0.027	-0.006	0.48	-0.09	0.057
ADC firstorder 10Percentile	0.86	-0.057	-0.065	-0.251	-0.051
ADC firstorder InterquartileRange	0.836	-0.112	-0.144	0.286	0.013
ADC firstorder Median	0.876	-0.068	-0.076	-0.184	-0.027
ADC firstorder 90Percentile	0.892	-0.076	-0.085	-0.102	-0.046
ADC firstorder Skewness	0.121	0.09	0.243	0.141	-0.116

Radiomic ADC feature	*ADC_F1*	*ADC_F2*	*ADC_F3*	*ADC_F4*	*ADC_F5*
ADC firstorder Range	0.82	-0.13	0.083	0.307	0.073
ADC firstorder Mean	0.881	-0.069	-0.076	-0.17	-0.035
ADC firstorder TotalEnergy	0.554	-0.042	0.194	0.331	0.491
ADC gldm LDLGLE	-0.002	0.898	-0.099	-0.012	-0.034
ADC gldm DependenceEntropy	-0.008	-0.217	0.49	0.721	0.068
ADC gldm GLNU	-0.148	0.637	0.559	-0.135	-0.024
ADC gldm LDHGLE	0.814	-0.066	0.089	0.131	0.103
ADC gldm DependenceVariance	-0.16	0.867	0.11	-0.079	-0.006
ADC gldm SDLGLE	0.501	0.202	-0.497	-0.424	-0.16
ADC ngtdm Busyness	-0.043	0.899	-0.054	-0.033	-0.027
ADC ngtdm Strength	0.845	-0.068	-0.184	0.161	0.015
ADC ngtdm Coarseness	-0.263	-0.027	-0.677	-0.292	0.157
ADC ngtdm Complexity	0.588	-0.022	-0.034	0.393	0.559
ADC ngtdm Contrast	0.777	-0.007	-0.087	-0.369	-0.273
ADC glrlm RunLengthNonUniformity	-0.038	-0.025	0.835	0.178	-0.058
ADC glrlm RunVariance	-0.206	0.853	0.107	-0.135	0.025
ADC glcm Imc2	0.041	-0.901	-0.021	0.028	0.017
ADC glcm DifferenceVariance	0.873	-0.019	-0.109	-0.184	-0.113
ADC glcm InverseVariance	-0.324	0.783	0.031	-0.269	0.083
ADC glcm Imc1	-0.382	0.346	0.678	-0.236	0.03
ADC glcm JointEntropy	-0.198	-0.2	0.774	0.355	0.029
ADC glcm ClusterShade	0.874	-0.014	-0.074	0.039	0.138
ADC glcm Correlation	-0.402	0.056	0.327	0.297	0.089
ADC glcm DifferenceEntropy	0.136	-0.451	0.198	0.729	0.068
ADC glcm SumEntropy	-0.084	-0.338	0.491	0.65	0.066
ADC glcm ClusterTendency	0.859	-0.038	-0.104	0.192	0.165
ADC glcm JointAverage	0.71	-0.157	0.112	0.326	0.088
ADC glcm MaximumProbability	0.25	0.629	-0.453	-0.344	-0.081
ADC glcm ClusterProminence	0.878	-0.009	-0.089	-0.03	0.2
ADC glcm Idn	-0.241	-0.016	0.757	0.106	0.078
ADC glcm DifferenceAverage	0.882	-0.092	-0.145	0.095	-0.049
ADC glcm Idmn	-0.387	-0.01	0.71	0.128	0.161
ADC glszm ZoneVariance	-0.061	0.905	-0.006	-0.014	-0.019

Radiomic ADC feature	*ADC_F1*	*ADC_F2*	*ADC_F3*	*ADC_F4*	*ADC_F5*
ADC glszm ZoneEntropy	0.038	-0.302	0.413	0.74	0.067
ADC glszm LALGLE	-0.006	0.899	-0.092	-0.005	-0.033
ADC glszm GLNUN	-0.046	0.806	-0.161	-0.364	0.027
ADC glszm SZNU	0.015	-0.116	0.774	0.294	-0.048

Table S6 T1C factor loadings for the 8 T1C factors. A darker grey in the column cell correspond to a higher loading measuring the association between features and factors. Abbreviations: T1C = post-contrast T1; F = Factor; glcm = gray level co-occurrence matrix; gldm = Gray Level Dependence Matrix; glszm = Gray Level Size Zone Matrix; ngtdm = Neighbouring Gray Tone Difference Matrix; glrlm = Gray Level Run Length Matrix.

Radiomic T1C feature	*T1C_F1*	*T1C_F2*	*T1C_F3*	*T1C_F4*	*T1C_F5*	*T1C_F6*	*T1C_F7*	*T1C_F8*
T1C glrlm RunPercentage	0.432	-0.104	-0.241	-0.747	0.158	-0.166	-0.251	-0.069
T1C glrlm GrayLevelNonUniformity	-0.25	0.226	0.746	0.481	-0.093	0.071	-0.06	0.056
T1C glrlm RunLengthNonUniformity	0.044	0.29	0.897	0.204	-0.045	0.004	-0.004	0.053
T1C glrlm RunEntropy	0.776	0.522	0.079	-0.166	0.015	-0.148	-0.01	-0.009
T1C glrlm ShortRunLowGrayLevelEmphasis	-0.171	-0.856	-0.291	0.026	0.061	-0.148	0.11	-0.184
T1C gldm SmallDependenceLowGrayLevelEmphasis	-0.102	-0.87	-0.287	-0.07	0.073	-0.17	0.028	-0.13
T1C gldm DependenceEntropy	0.327	0.704	0.416	0.275	-0.094	0.002	0.262	0.041
T1C gldm SmallDependenceHighGrayLevelEmphasis	0.825	0.19	0.157	-0.239	0.145	0.073	-0.085	0.264
T1C gldm DependenceNonUniformity	0.278	0.289	0.875	-0.088	-0.004	0.044	-0.045	0.011
T1C gldm LargeDependenceLowGrayLevelEmphasis	-0.322	-0.356	-0.138	0.673	-0.134	0.045	0.269	-0.268
T1C gldm LargeDependenceHighGrayLevelEmphasis	0.285	0.354	0.544	0.185	0.028	-0.087	0.156	0.588
T1C gldm DependenceVariance	-0.385	0.087	0.183	0.781	-0.154	0.208	0.215	0.055
T1C glcm SumSquares	0.936	0.156	-0.09	-0.118	0.049	-0.045	0.046	-0.009
T1C glcm JointAverage	0.675	0.336	0.296	-0.254	0.122	-0.067	-0.079	0.43
T1C glcm Idn	-0.044	0.569	0.454	0.248	-0.203	0.434	0.206	0.149
T1C glcm Imc2	0.402	-0.166	-0.673	-0.376	0.077	-0.044	-0.027	-0.071
T1C glcm ClusterShade	0.551	0.03	0.033	-0.056	-0.031	0.497	-0.04	-0.044
T1C glcm Correlation	0.081	0.495	0.173	0.336	-0.202	-0.005	0.456	0.021
T1C glcm Imc1	-0.251	0.479	0.684	0.213	-0.055	0.113	0.257	0.061
T1C glcm ClusterTendency	0.917	0.172	-0.053	-0.078	0.034	-0.061	0.152	0

Radiomic T1C feature	*T1C_F1*	*T1C_F2*	*T1C_F3*	*T1C_F4*	*T1C_F5*	*T1C_F6*	*T1C_F7*	*T1C_F8*
T1C glcm InverseVariance	-0.599	-0.04	0.147	0.57	-0.182	0.275	0.32	0.041
T1C glcm SumEntropy	0.649	0.655	0.191	-0.122	-0.018	-0.103	0.13	-0.033
T1C glcm ClusterProminence	0.902	0.063	0.022	0.009	0.066	0.155	0.163	0.039
T1C glcm MaximumProbability	-0.299	-0.858	-0.253	0.136	0.025	0.047	-0.022	0.067
T1C glcm DifferenceVariance	0.87	0.114	-0.118	-0.17	0.035	0.083	-0.166	-0.02
T1C glcm JointEnergy	-0.207	-0.912	-0.181	0.06	0.076	0.005	-0.025	0.055
T1C glcm DifferenceEntropy	0.745	0.37	-0.035	-0.391	0.105	-0.147	-0.193	-0.039
T1C glcm JointEntropy	0.385	0.657	0.542	-0.058	0.001	-0.045	0.179	0.024
T1C firstorder RootMeanSquared	0.428	-0.048	0.021	-0.282	0.797	-0.19	-0.045	0.091
T1C firstorder Kurtosis	-0.128	0.091	0.148	0.131	-0.161	0.793	0.013	0.131
T1C firstorder Maximum	0.757	0.163	0.143	-0.282	0.419	0.169	-0.08	-0.017
T1C firstorder 10Percentile	0.111	-0.151	0.081	-0.234	0.902	-0.105	-0.065	0.11
T1C firstorder Skewness	0.186	0.16	-0.098	0.12	-0.231	0.689	0.015	-0.212
T1C firstorder Range	0.784	0.331	0.247	-0.224	0.01	0.257	-0.087	0.106
T1C firstorder InterquartileRange	0.84	0.235	-0.163	-0.201	0.022	-0.289	0.059	-0.018
T1C firstorder 90Percentile	0.61	0.024	-0.018	-0.288	0.663	-0.205	-0.044	0.056
T1C firstorder Minimum	-0.117	-0.354	-0.223	-0.093	0.799	-0.188	0.022	-0.253
T1C firstorder TotalEnergy	0.169	0.299	0.855	0.101	0.201	-0.03	0.069	0.11
T1C ngtdm Complexity	0.879	0.137	0.142	-0.122	0.07	0.212	-0.03	0.044
T1C ngtdm Strength	0.551	-0.223	-0.598	-0.167	-0.055	0.014	-0.171	0.04
T1C ngtdm Coarseness	-0.095	-0.899	-0.282	-0.06	0.075	-0.115	0.002	0.046
T1C ngtdm Busyness	-0.445	0.195	0.539	0.535	-0.088	-0.012	0.019	-0.231
T1C ngtdm Contrast	0.555	-0.193	-0.511	-0.228	0.092	-0.314	-0.262	-0.069
T1C glszm ZoneVariance	-0.173	0.057	0.141	0.889	-0.153	0.017	-0.17	0.007
T1C glszm SizeZoneNonUniformity	0.323	0.28	0.845	-0.175	0.007	0.049	-0.045	0.002
T1C glszm SmallAreaLowGrayLevelEmphasis	-0.134	-0.879	-0.258	-0.022	0.05	-0.152	0.071	-0.116
T1C glszm ZoneEntropy	0.407	0.715	0.397	0.218	-0.088	-0.01	0.176	0.053
T1C glszm LargeAreaLowGrayLevelEmphasis	-0.252	-0.181	-0.008	0.852	-0.172	0.046	-0.003	-0.122
T1C glszm GrayLevelNonUniformityNormalized	-0.691	-0.566	-0.008	0.237	-0.009	0.214	0.133	0.022
T1C glszm LargeAreaHighGrayLevelEmphasis	0.012	0.215	0.397	0.759	-0.065	-0.063	-0.104	0.3
T1C glszm GrayLevelVariance	0.939	0.178	-0.052	-0.11	0.065	-0.016	0.021	0.032
T1C glszm GrayLevelNonUniformity	-0.149	0.276	0.899	0.1	-0.066	0.066	-0.024	0.031
T1C glszm SizeZoneNonUniformityNormalized	0.507	-0.177	-0.325	-0.552	0.17	-0.095	-0.404	-0.116

Table S7 Factor loadings for the 19 pathomic factors. A darker grey in the column cell correspond to a higher loadings measuring the association between features and factors. Abbreviations: P = Pathomic; F = Factor; CD = Cellular Density; OD = Optical Density; glcm = gray level co-occurrence matrix; gldm = Gray Level Dependence Matrix; glszm = Gray Level Size Zone Matrix; ngtdm = Neighbouring Gray Tone Difference Matrix; glrlm = Gray Level Run Length Matrix.

Pathomic feature name	*P_F1*	*P_F2*	*P_F3*	*P_F4*	*P_F5*	*P_F6*	*P_F7*	*P_F8*	*P_F9*	*P_F10*	*P_F11*	*P_F12*	*P_F13*	*P_F14*	*P_F15*	*P_F16*	*P_F17*	*P_F18*	*P_F19*
MEAN Nucleus Area	0.205	-0.136	0.007	-0.001	-0.036	0.13	0.013	0.096	-0.846	-0.077	-0.071	0.029	-0.024	-0.027	0.027	0.039	0.003	-0.012	-0.011
MEAN Nucleus Perimeter	0.266	-0.053	0.052	0.055	0.02	0.118	-0.04	0.064	-0.814	-0.116	-0.044	-0.004	-0.008	0.196	0.004	0.058	0.017	0.005	0.005
MEAN Nucleus Circularity	-0.246	-0.258	-0.096	-0.112	-0.197	-0.032	0.029	0.063	0.226	0.173	-0.105	0.136	-0.006	-0.684	0.06	-0.039	-0.044	-0.009	-0.015
MEAN Nucleus MaxCaliper	0.312	-0.035	0.108	0.02	0.07	0.085	-0.051	0.057	-0.758	-0.143	-0.08	-0.005	-0.01	0.276	0.004	0.097	-0.015	0.02	0.005
MEAN Nucleus MinCaliper	0.093	-0.159	-0.092	0.058	-0.098	0.139	-0.005	0.105	-0.822	-0.033	-0.001	0.048	-0.005	-0.171	0.034	-0.042	0.054	-0.016	-0.019
MEAN Nucleus Eccentricity	0.353	0.274	0.149	0.044	0.289	-0.047	-0.013	-0.053	-0.06	-0.334	-0.091	-0.093	-0.041	0.555	-0.088	0.092	-0.114	-0.016	-0.012
MEAN Nucleus HematoxylinODMean	0.274	0.07	0.131	-0.007	0.419	0.003	0.738	0.024	0.084	-0.033	0.004	-0.049	-0.013	-0.065	0.014	0.017	-0.002	-0.035	-0.009
MEAN Nucleus HematoxylinODSum	0.328	-0.042	0.125	-0.024	0.393	0.102	0.674	0.072	-0.249	-0.023	0.001	-0.054	-0.039	-0.057	0.009	0.019	0.002	0.001	0.004
MEAN Nucleus HematoxylinODStdDev	0.15	0.011	0.069	-0.056	0.5	-0.039	0.646	0.017	0.229	-0.108	-0.031	-0.063	0.013	0.124	-0.015	0.01	0.017	-0.033	-0.098
MEAN Nucleus HematoxylinODMin	0.337	0.117	0.17	0.017	0.266	0.031	0.717	0.021	-0.023	0.042	0.025	-0.022	-0.005	-0.194	0.051	0.035	-0.058	-0.051	0.094
MEAN Nucleus HematoxylinODRange	0.18	-0.016	0.049	-0.003	0.503	-0.036	0.645	0.051	0.169	-0.113	-0.024	-0.075	0.017	0.136	-0.034	0.003	0.047	0.023	-0.112
MEAN Nucleus EosinODMean	0.015	0.104	-0.01	0.001	0.281	0.836	-0.116	0.07	0.012	-0.025	0.001	0.052	0.018	-0.043	0.014	-0.017	0.031	0.01	0.07
MEAN Nucleus EosinODSum	0.102	-0.025	-0.011	0.011	0.272	0.771	-0.083	0.093	-0.32	-0.032	-0.01	0.026	-0.009	-0.052	0.002	-0.026	0.035	-0.016	0.043
MEAN Nucleus EosinODStdDev	-0.182	0.071	-0.087	-0.11	0.728	0.108	0.09	0.051	0.354	0.091	0.046	0.021	-0.072	0.059	0.098	-0.013	0.142	-0.037	-0.067
MEAN Nucleus EosinODMin	0.079	0.084	0.039	0.041	-0.036	0.866	-0.155	0.05	-0.092	-0.044	-0.038	0.048	0.052	-0.049	-0.034	-0.015	-0.034	0.005	0.093
MEAN Nucleus EosinODRange	-0.103	0.072	-0.095	-0.051	0.762	0.184	0.163	0.074	0.257	0.037	0.063	0.011	-0.08	0.091	0.085	-0.01	0.16	0.025	-0.095
MEAN Cell Perimeter	-0.319	-0.378	-0.079	-0.684	0.001	-0.034	-0.058	0.011	-0.299	-0.017	-0.062	-0.045	-0.063	0.039	-0.013	0.051	0.032	-0.012	-0.03
MEAN Cell Circularity	-0.489	-0.351	-0.101	-0.507	-0.084	-0.125	-0.004	-0.047	0.292	0.214	0.007	-0.069	-0.002	-0.181	-0.016	-0.03	0.033	0.019	-0.001
MEAN Cell MaxCaliper	-0.229	-0.34	-0.051	-0.669	0.022	-0.01	-0.076	0.026	-0.401	-0.09	-0.092	-0.03	-0.072	0.098	-0.009	0.08	0.019	-0.007	-0.033
MEAN Cell Eccentricity	0.48	0.366	0.086	0.355	0.12	0.108	-0.028	0.037	-0.288	-0.389	-0.07	0.062	-0.02	0.216	0.013	0.045	-0.06	0.012	0.003
MEAN Cell HematoxylinODMean	0.42	0.169	0.202	0.201	0.195	0.021	0.663	0.049	-0.099	0.04	0.059	-0.019	0.043	-0.078	-0.014	0.018	-0.02	0.052	0.109
MEAN Cell HematoxylinODStdDev	0.268	0.058	0.106	0.075	0.503	0.102	0.662	0.032	0.082	-0.064	-0.023	-0.018	-0.025	0.017	0.018	0.004	0.007	-0.04	-0.095
MEAN Cell HematoxylinODMax	0.244	0.034	0.092	0.002	0.457	-0.017	0.721	0.043	0.119	-0.068	-0.007	-0.062	0.008	0.038	-0.008	0.013	0.016	-0.002	-0.053

Pathomic feature name	***P_F1***	***P_F2***	***P_F3***	***P_F4***	***P_F5***	***P_F6***	***P_F7***	***P_F8***	***P_F9***	***P_F10***	***P_F11***	***P_F12***	***P_F13***	***P_F14***	***P_F15***	***P_F16***	***P_F17***	***P_F18***	***P_F19***
MEAN Cell EosinODMean	0.147	0.113	0.05	0.035	0.113	0.878	0.023	0.055	-0.046	-0.045	0.003	0.044	0.003	0.015	0.039	0.01	-0.009	-0.001	-0.008
MEAN Cell EosinODStdDev	-0.172	0.15	-0.13	0.005	0.679	0.381	-0.151	-0.005	0.103	0.053	0.157	0.026	-0.057	0.231	0.083	0.016	0.047	-0.034	-0.033
MEAN Cell EosinODMax	0.003	0.097	-0.021	0.009	0.394	0.793	0.016	0.052	0.08	-0.003	0.038	0.036	-0.029	0.098	0.047	0.002	0.062	0.006	-0.072
MEAN Cell EosinODMin	0.213	0.085	0.11	0.03	-0.148	0.83	0.007	0.099	-0.068	-0.008	-0.077	0.054	0.076	-0.084	-0.067	-0.028	-0.022	-0.028	0.012
MEAN Cytoplasm HematoxylinODMean	0.393	0.202	0.2	0.156	0.027	-0.032	0.613	0.037	-0.118	0.078	0.11	-0.049	0.069	-0.076	-0.02	0.021	-0.022	0.084	0.186
MEAN Cytoplasm HematoxylinODStdDev	0.275	0.145	0.104	0.274	0.395	0.361	0.514	0.019	-0.035	-0.006	0.059	-0.01	0.034	0.236	0.029	-0.027	-0.029	0.04	-0.071
MEAN Cytoplasm HematoxylinODMax	0.292	0.125	0.13	0.127	0.397	0.052	0.714	0.032	-0.004	-0.032	0.047	-0.052	0.032	0.086	-0.06	-0.014	0.017	0.036	-0.01
MEAN Cytoplasm HematoxylinODMin	0.144	0.097	0.144	-0.067	-0.161	-0.642	0.31	-0.024	-0.064	0.056	-0.029	-0.037	0.062	-0.311	-0.117	0.012	-0.016	-0.023	0.239
MEAN Cytoplasm EosinODMean	0.154	0.105	0.07	0.017	0.069	0.879	0.061	0.051	-0.04	-0.046	0.004	0.043	0.003	0.032	0.045	0.018	-0.022	-0.001	-0.026
MEAN Cytoplasm EosinODStdDev	-0.073	0.191	-0.096	-0.003	0.536	0.522	-0.029	-0.026	0.022	0.028	0.193	0.021	-0.046	0.324	0.119	0.057	-0.043	-0.025	-0.035
MEAN Cytoplasm EosinODMax	0.05	0.121	0.006	0.002	0.302	0.827	0.033	0.039	0.029	-0.01	0.054	0.04	-0.027	0.13	0.067	0.027	0.011	-0.005	-0.061
MEAN Cytoplasm EosinODMin	0.228	0.092	0.131	0.018	-0.122	0.821	0.078	0.104	-0.047	-0.016	-0.092	0.047	0.067	-0.081	-0.055	-0.029	-0.016	-0.039	-0.016
MEAN Nucleus CellAreaRatio	0.425	0.18	0.095	0.608	-0.039	0.111	-0.01	0.064	-0.451	-0.054	-0.004	0.062	0.056	-0.036	0.015	-0.016	-0.028	0.03	0.015
MEAN Delaunay MinDistance	-0.363	-0.442	0.056	-0.345	-0.15	-0.103	-0.054	-0.011	0.125	0.555	0.012	-0.058	0.029	-0.064	-0.072	0.036	0.019	-0.003	-0.028
MEAN Delaunay MeanTriangleArea	-0.321	-0.396	0.093	-0.236	-0.172	-0.097	-0.065	-0.011	0.176	0.632	0.014	-0.054	0.032	-0.065	-0.101	0.044	0.015	-0.028	-0.021
MEAN Delaunay MaxTriangleArea	-0.306	-0.388	0.117	-0.247	-0.172	-0.097	-0.054	0.001	0.167	0.633	0.011	-0.074	0.03	-0.06	-0.102	0.056	0.025	-0.023	-0.017
MEAN DM Std dev	0.036	0.054	-0.059	0.086	0.841	0.103	0.267	0.03	-0.056	0.006	0.014	-0.058	-0.016	0.013	-0.016	-0.048	0.028	-0.054	0.061
MEAN DM Min	0.297	0.128	0.164	0.087	-0.104	0.787	0.195	0.077	-0.046	-0.027	-0.003	0.051	0.02	-0.086	0.018	0.021	0	0.042	0.024
MEAN DM Max	0.2	0.09	0.076	0.049	0.447	0.67	0.329	0.078	-0.047	-0.024	0.005	-0.004	-0.002	-0.069	0.022	-0.007	0.031	0.021	0.047
MEAN DM HaralickAngularSecondMoment F0	0.063	0.07	0.105	0.147	-0.686	-0.092	-0.061	-0.014	0.268	0.262	0.302	-0.06	-0.066	-0.003	0.026	-0.007	0.213	-0.07	0.031
MEAN DM HaralickContrast F1	0.042	0.121	-0.039	0.147	0.78	-0.006	0.329	0.049	0.078	0.056	0.004	-0.026	-0.07	-0.012	0.036	-0.049	0.121	0.02	-0.002
MEAN DM HaralickCorrelation F2	-0.243	-0.231	-0.16	-0.521	0.262	-0.003	-0.02	0.014	-0.466	-0.086	-0.077	-0.015	-0.02	-0.014	-0.091	-0.02	-0.139	-0.209	0.03
MEAN DM HaralickSumOfSquares F3	0.006	0.068	-0.071	0.018	0.804	0.029	0.323	0.046	-0.029	0.045	0.043	-0.065	-0.08	-0.004	0.003	-0.06	0.087	-0.045	0.031
MEAN DM HaralickInverseDifferenceMomen	-0.147	-0.119	0.008	-0.297	-0.774	-0.181	-0.182	-0.005	0.042	0.085	0.091	-0.01	-0.089	-0.012	0.015	0.019	0.088	-0.016	-0.059
MEAN DM HaralickSumAverage F5	0.284	0.148	0.119	0.107	0.159	0.768	0.263	0.064	-0.091	-0.028	0.016	0.028	0.025	-0.034	0.03	0.015	-0.016	0.021	0.04
MEAN DM HaralickSumEntropy F7	-0.125	-0.192	-0.122	-0.324	0.679	0.137	0.133	0.021	-0.28	-0.178	-0.133	-0.045	-0.006	-0.06	0.082	-0.002	-0.111	-0.03	0.032
MEAN DM HaralickEntropy F8	-0.16	-0.177	-0.122	-0.315	0.689	0.135	0.114	0.015	-0.261	-0.213	-0.148	-0.029	0.028	-0.004	0.015	0.005	-0.11	0.028	0.014
MEAN DM HaralickDifferenceVariance F9	0.09	0.158	0.052	0.051	-0.837	-0.106	-0.11	-0.023	-0.023	0.043	0.163	0.012	-0.054	0.096	0.043	0.017	0.075	-0.021	-0.021
MEAN DM HaralickInformationMeasureOfCo	-0.241	-0.212	-0.018	-0.44	-0.662	-0.151	-0.176	0	0.052	0.036	0.044	-0.006	-0.092	-0.036	0.041	0.03	0.08	0.034	-0.084
MEAN DM HaralickInformationMeasureOf 1	0.094	0.045	-0.028	0.22	0.737	0.119	0.068	-0.002	-0.178	-0.134	-0.265	0.041	0.117	-0.014	-0.072	-0.022	-0.211	0.034	0.017
CD50um firstorder 10Percentile	0.306	0.195	-0.367	0.744	-0.014	0.017	0.029	0.053	0.009	-0.027	-0.011	0.004	0.034	0.004	-0.003	0.013	0.007	0.037	0.038
CD50um firstorder 90Percentile	0.481	0.441	0.057	0.594	-0.033	0.075	0.064	0.012	0.041	-0.072	0.129	0.098	0.038	0.038	0.047	-0.027	0.01	0.038	0.027
CD50um firstorder Entropy	0.606	0.535	-0.013	0.156	0.005	0.113	0.065	-0.013	-0.028	-0.239	0.158	0.19	0.004	0.085	0.07	-0.041	0.016	0.052	0.024

Pathomic feature name	*P_F1*	*P_F2*	*P_F3*	*P_F4*	*P_F5*	*P_F6*	*P_F7*	*P_F8*	*P_F9*	*P_F10*	*P_F11*	*P_F12*	*P_F13*	*P_F14*	*P_F15*	*P_F16*	*P_F17*	*P_F18*	*P_F19*
CD50um firstorder InterquartileRange	0.498	0.562	0.163	0.084	-0.053	0.112	-0.002	-0.074	0.081	-0.068	0.198	0.302	0.014	0.052	0.045	-0.021	0.025	0.101	0.018
CD50um firstorder Kurtosis	-0.202	-0.326	0.015	-0.351	-0.165	-0.162	0.053	-0.017	0.17	0.359	-0.08	-0.5	0.109	0.041	-0.216	0	-0.042	0.039	0.015
CD50um firstorder Maximum	0.681	0.327	0.015	0.384	-0.038	0.059	0.073	0.125	0.001	-0.09	0.171	0.02	0.044	0.01	0.037	0.095	-0.017	0.111	0.042
CD50um firstorder Minimum	-0.065	-0.003	-0.128	0.743	0.046	-0.026	-0.027	-0.22	-0.172	0.009	0.081	-0.1	-0.191	0.013	0.057	0.068	0.049	0.18	-0.111
CD50um firstorder Range	0.726	0.338	0.064	0.111	-0.057	0.071	0.086	0.213	0.067	-0.096	0.144	0.06	0.119	0.006	0.017	0.07	-0.036	0.043	0.086
CD50um firstorder RMAD	0.387	0.471	0.576	0.07	0.002	0.102	0.051	-0.052	0.06	-0.106	0.207	0.19	0.015	0.023	0.054	-0.051	0.001	0.028	-0.009
CD50um firstorder Skewness	-0.259	-0.235	-0.301	-0.572	-0.153	-0.087	-0.09	-0.139	0.056	0.171	0.257	-0.234	-0.113	0.027	-0.077	0.042	0.015	0.204	-0.005
CD50um firstorder Uniformity	-0.494	-0.488	0.093	-0.156	-0.099	-0.111	-0.065	0.008	0.145	0.47	-0.058	-0.157	0.041	-0.078	-0.091	0.04	-0.004	-0.042	-0.008
CD50um glcm JointAverage	0.558	0.432	0.076	0.404	-0.03	0.081	0.105	0.184	0.129	-0.069	-0.025	0.145	0.175	0.007	-0.008	-0.061	-0.049	-0.088	0.087
CD50um glcm ClusterProminence	0.154	0.258	0.815	-0.016	-0.065	0.086	0.07	-0.028	0.099	-0.06	0.184	-0.037	0.02	0.011	0.056	-0.04	0.027	-0.037	0.042
CD50um glcm ClusterShade	-0.001	-0.069	-0.787	-0.169	-0.179	-0.036	-0.026	-0.041	0.082	0.021	0.287	-0.022	-0.088	0.034	0.074	0.045	0.136	0.088	0.054
CD50um glcm ClusterTendency	0.338	0.381	0.631	-0.021	-0.088	0.09	0.068	-0.051	0.134	-0.043	0.316	0.096	0.028	0.037	0.084	-0.056	0.034	0.031	0.056
CD50um glcm Contrast	0.205	0.771	0.029	0.223	-0.016	0.212	-0.002	0.01	0.078	-0.047	0.013	-0.062	0.041	0.039	-0.04	0.078	0.128	-0.135	0.107
CD50um glcm Correlation	0.603	0.156	0.342	-0.187	-0.137	-0.044	0.115	-0.076	0.086	0.089	0.341	0.222	-0.01	0.091	-0.009	-0.143	-0.081	0.137	-0.037
CD50um glcm DifferenceAverage	0.247	0.744	-0.113	0.261	0.022	0.197	-0.015	0.027	-0.014	-0.167	0.016	-0.033	0.011	0.068	-0.016	0.074	0.109	-0.138	0.058
CD50um glcm DifferenceVariance	0.205	0.748	0.235	0.175	-0.03	0.198	0.017	-0.014	0.127	-0.049	0.037	-0.089	0.042	0.05	-0.039	0.059	0.128	-0.096	0.135
CD50um glcm JointEnergy	-0.457	-0.338	0.521	-0.103	-0.072	-0.071	-0.036	-0.001	0.124	0.415	-0.04	-0.094	0.047	-0.097	-0.044	0.001	-0.047	-0.025	-0.016
CD50um glcm JointEntropy	0.715	0.415	-0.1	0.179	0.008	0.131	0.054	0.015	-0.02	-0.197	0.102	0.133	0.016	0.083	0.046	0.001	0.064	0.033	0.032
CD50um glcm Imc2	-0.119	0.55	0.214	-0.217	-0.182	-0.012	0.075	-0.192	0.146	-0.004	0.317	0.295	0.044	0.115	0.035	-0.188	-0.135	0.205	-0.046
CD50um glcm Idm	-0.284	-0.538	0.416	-0.242	-0.044	-0.127	0.006	-0.039	0.153	0.386	0.012	-0.014	0.038	-0.066	-0.041	-0.046	-0.08	0.098	0.039
CD50um glcm Idmn	0.808	-0.127	0.061	-0.108	-0.116	-0.118	0.096	0.119	0.061	0.092	0.081	-0.015	-0.005	-0.028	-0.157	0.034	-0.108	-0.02	-0.009
CD50um glcm Idn	0.765	-0.167	0.168	-0.121	-0.111	-0.103	0.109	0.154	0.103	0.099	0.117	0.014	0.052	-0.036	-0.096	0.029	-0.117	0.066	0.039
CD50um glcm InverseVariance	-0.286	-0.649	-0.085	-0.299	-0.013	-0.198	0.047	-0.059	0.151	0.271	-0.031	0.014	0.062	-0.07	-0.015	-0.052	-0.016	0.181	0.013
CD50um glcm MaximumProbability	-0.262	-0.15	0.759	-0.073	-0.09	-0.044	-0.019	-0.021	0.078	0.321	-0.005	-0.098	0.02	-0.069	-0.086	-0.02	-0.063	-0.066	-0.038
CD50um glcm SumEntropy	0.658	0.488	0.007	0.056	-0.055	0.075	0.057	-0.027	0.03	-0.118	0.244	0.217	0.004	0.114	0.03	-0.058	0.003	0.069	0.017
CD50um glcm SumSquares	0.341	0.455	0.591	0.009	-0.084	0.112	0.063	-0.047	0.135	-0.046	0.296	0.081	0.031	0.039	0.073	-0.042	0.048	0.011	0.067
CD50um glrlm RLNUN	0.182	0.654	-0.258	0.197	-0.031	0.107	0.02	-0.031	-0.078	-0.289	-0.025	-0.075	-0.068	0.035	-0.006	0.031	0.158	-0.076	-0.175
CD50um glrlm RunPercentage	0.072	0.36	-0.775	0.097	-0.023	0.027	-0.023	-0.008	-0.051	-0.196	0.016	-0.003	-0.008	0.041	0.016	0.04	0.11	-0.024	-0.07
CD50um glrlm RunVariance	0.022	0.044	0.902	0.02	0.037	0.045	0.037	-0.009	-0.008	-0.027	-0.035	-0.048	-0.021	-0.029	-0.023	-0.035	-0.057	-0.035	-0.027
CD50um glszm GLNUN	-0.501	-0.491	0.052	-0.156	-0.097	-0.111	-0.066	0.009	0.145	0.47	-0.06	-0.148	0.04	-0.078	-0.083	0.044	-0.006	-0.039	-0.005

Pathomic feature name	*P_F1*	*P_F2*	*P_F3*	*P_F4*	*P_F5*	*P_F6*	*P_F7*	*P_F8*	*P_F9*	*P_F10*	*P_F11*	*P_F12*	*P_F13*	*P_F14*	*P_F15*	*P_F16*	*P_F17*	*P_F18*	*P_F19*
CD50um glszm SALGLE	-0.599	-0.384	0.068	-0.167	-0.102	-0.03	-0.069	-0.038	0.122	0.471	0.006	-0.016	0.074	-0.054	0.064	0.05	0.074	0.113	0.044
CD50um gldm DependenceEntropy	0.701	0.386	0.022	0.135	0.012	0.104	0.078	0.002	-0.007	-0.185	0.169	0.231	0.014	0.08	0.092	-0.044	-0.004	0.093	0.075
CD50um gldm DependenceVariance	-0.01	-0.225	0.858	-0.052	0.02	0.003	0.038	0.00E+00	0.033	0.13	-0.028	-0.023	-0.022	-0.033	-0.02	-0.041	-0.077	0.007	0.022
CD50um gldm LDLGLE	-0.073	0.029	0.902	0.003	0.02	0.042	0.02	-0.01	0	0.036	-0.026	-0.05	-0.003	-0.037	-0.015	-0.027	-0.051	-0.032	-0.03
CD50um gldm LowGrayLevelEmphasis	-0.374	-0.171	0.751	-0.087	-0.038	0.017	-0.022	-0.027	0.058	0.277	-0.013	-0.046	0.038	-0.057	0.021	0.003	-0.007	0.031	-0.003
CD50um ngtdm Busyness	0.284	-0.337	-0.053	-0.008	0.032	0.144	0.038	0.751	-0.13	0.043	-0.045	0.012	0.027	-0.014	0.005	0.02	0.035	0.015	0.007
CD50um ngtdm Complexity	0.607	0.437	0.065	0.152	-0.054	0.195	0.112	0.258	0.124	0.021	0.074	0.021	0.178	-0.003	0.046	0.036	0.035	0.032	0.137
CD50um ngtdm Contrast	-0.262	0.642	0.413	0.113	0.024	0.13	-0.023	-0.177	0.019	-0.144	0.165	0.156	-0.066	0.05	0.113	-0.049	0.062	0.001	0.011
CD50um ngtdm Strength	-0.374	0.602	-0.028	-0.208	-0.127	-0.081	-0.017	-0.131	0.14	0.022	0.381	-0.071	-0.074	0.112	-0.138	-0.043	-0.061	-0.122	0.033
CD100um firstorder 10Percentile	0.373	0.071	-0.362	0.741	0.023	0.013	0.021	0.064	-0.005	-0.045	0.001	-0.003	0.024	-0.009	0.021	0.008	-0.038	0.01	0.009
CD100um firstorder Entropy	0.672	0.458	0.036	0.184	0.042	0.102	0.093	-0.023	-0.022	-0.136	0.102	0.23	0.033	0.081	0.082	-0.049	-0.013	0.115	-0.002
CD100um firstorder InterquartileRange	0.366	0.571	0.149	0.034	-0.041	0.04	0.013	-0.084	0.072	0.001	0.267	0.43	0.08	-0.03	0.049	-0.073	-0.063	0.144	0.015
CD100um firstorder Kurtosis	0.005	-0.329	0.022	-0.148	-0.024	-0.143	0.115	0.012	0.089	0.221	0.003	-0.736	0.06	0.055	-0.1	-0.063	0.045	0.074	-0.071
CD100um firstorder Maximum	0.652	0.31	0.053	0.424	-0.046	0.088	0.166	0.099	-0.009	-0.063	0.161	-0.064	0.001	0.072	0.055	-0.037	-0.028	0.138	0.003
CD100um firstorder Minimum	-0.039	-0.018	-0.261	0.618	-0.017	-0.091	0.104	-0.149	-0.017	-0.2	0.072	-0.015	-0.467	-0.011	-0.088	0.087	-0.019	0.044	-0.047
CD100um firstorder Range	0.691	0.328	0.181	0.136	-0.039	0.135	0.12	0.174	-0.001	0.033	0.131	-0.059	0.231	0.081	0.101	-0.081	-0.018	0.119	0.026
CD100um firstorder RMAD	0.286	0.504	0.582	0.034	-0.047	0.062	0.055	-0.058	0.015	-0.027	0.191	0.29	0.023	-0.023	0.049	-0.065	-0.018	0.086	-0.005
CD100um firstorder Skewness	-0.134	-0.145	-0.252	-0.65	-0.148	-0.105	-0.006	-0.118	0.087	0.177	0.282	-0.231	-0.166	0.003	-0.09	-0.042	-0.003	0.159	-0.028
CD100um firstorder Uniformity	-0.578	-0.457	0.079	-0.154	-0.114	-0.095	-0.079	0.024	0.116	0.388	-0.031	-0.194	0.023	-0.099	-0.072	0.041	0.028	-0.072	0.005
CD100um glcm ClusterProminence	0.124	0.207	0.834	-0.01	-0.075	0.078	0.064	-0.023	0.076	-0.075	0.196	-0.042	-0.01	0.005	0.057	-0.039	0.038	-0.036	0.024
CD100um glcm ClusterShade	0.008	-0.035	-0.783	-0.149	-0.189	-0.023	-0.01	-0.03	0.115	0.034	0.302	-0.008	-0.068	0.023	0.088	0.033	0.151	0.057	0.073
CD100um glcm ClusterTendency	0.308	0.365	0.65	-0.02	-0.099	0.08	0.07	-0.046	0.106	-0.017	0.329	0.137	0.021	0.02	0.083	-0.06	0.027	0.05	0.034
CD100um glcm Contrast	0.048	0.818	0.243	0.094	0.062	0.084	0.065	-0.06	0.161	0.114	0.017	-0.013	0.05	0.038	-0.017	0.014	0.074	-0.024	0.111
CD100um glcm Correlation	0.63	-0.146	0.236	-0.117	-0.212	-0.04	0.042	0.028	-0.056	0.179	0.343	0.247	-0.058	0.03	-0.082	-0.108	-0.082	0.009	-0.111
CD100um glcm DifferenceAverage	0.03	0.866	0.081	0.116	0.104	0.074	0.047	-0.057	0.091	-0.014	-0.007	0.036	0.051	0.076	0.06	0.018	0.029	-0.011	0.084
CD100um glcm DifferenceEntropy	0.328	0.786	0.076	0.128	0.1	0.078	0.059	-0.077	0.009	-0.171	-0.031	-0.012	0.028	0.124	0.019	0.009	0.035	0.012	0.024
CD100um glcm DifferenceVariance	0.098	0.744	0.392	0.096	0.02	0.092	0.079	-0.076	0.172	0.102	0.064	-0.058	0.049	0.03	-0.048	-0.003	0.104	-0.002	0.101
CD100um glcm JointEnergy	-0.752	-0.183	0.333	-0.103	-0.096	-0.067	-0.059	0.02	0.06	0.267	0.032	0.002	0.088	-0.038	0.018	0.017	0.025	0.05	0.039
CD100um glcm JointEntropy	0.852	0.021	0.01	0.167	0.061	0.098	0.061	0.078	-0.061	-0.117	-0.049	0.111	0.074	0.033	0.101	0.012	0.01	0.07	0.016
CD100um glcm Imc1	0.537	-0.58	-0.043	0.081	0.097	0.02	-0.007	0.153	-0.102	-0.051	-0.278	-0.19	0.043	-0.06	0.025	0.1	0.035	-0.085	0.022
CD100um glcm Imc2	-0.271	0.48	0.109	-0.071	-0.2	0.023	0.04	-0.419	0.165	0.008	0.179	0.285	0.045	0.141	-0.011	-0.151	-0.077	0.225	-0.085
CD100um glcm Idm	-0.041	-0.625	0.28	-0.166	-0.173	-0.123	-0.008	0.016	0.079	0.331	0.002	-0.101	-0.031	-0.032	-0.377	-0.004	0.09	-0.002	-0.069
CD100um glcm Idmn	0.776	-0.242	0.013	-0.019	-0.113	-0.011	0.055	0.051	-0.099	0.189	0.085	-0.161	-0.007	-0.004	-0.167	-0.028	-0.024	-0.097	-0.155

Pathomic feature name	*P_F1*	*P_F2*	*P_F3*	*P_F4*	*P_F5*	*P_F6*	*P_F7*	*P_F8*	*P_F9*	*P_F10*	*P_F11*	*P_F12*	*P_F13*	*P_F14*	*P_F15*	*P_F16*	*P_F17*	*P_F18*	*P_F19*
CD100um glcm Idn	0.763	-0.274	0.089	0.004	-0.127	0.011	0.073	0.103	-0.077	0.191	0.128	-0.156	0.052	0.003	-0.135	-0.056	0.003	-0.005	-0.112
CD100um glcm InverseVariance	-0.182	-0.714	0.013	-0.076	-0.126	-0.063	-0.026	0.055	0.051	0.415	0.036	-0.113	0.012	-0.16	-0.01	-0.05	0.036	-0.02	-0.114
CD100um glcm MaximumProbability	-0.418	-0.046	0.736	-0.135	-0.152	-0.05	-0.004	-0.028	-0.055	0.135	-0.006	-0.004	0.033	0.079	-0.056	0.065	0.135	0.00E+00	0.009
CD100um glcm SumEntropy	0.758	0.317	0.08	0.116	-0.057	0.095	0.068	-0.006	-0.006	-0.071	0.172	0.251	0.06	0.067	0.026	-0.06	-0.037	0.079	-0.028
CD100um glcm SumSquares	0.284	0.457	0.622	-0.003	-0.079	0.085	0.073	-0.051	0.12	0.003	0.298	0.121	0.027	0.024	0.072	-0.052	0.036	0.041	0.049
CD100um glrlm LongRunEmphasis	0.119	-0.184	0.668	-0.179	-0.13	-0.087	0.074	-0.004	-0.154	0.036	-0.114	0.029	-0.019	0.226	-0.287	0.156	0.2	0.032	0.061
CD100um glrlm RLNUN	-0.235	0.495	-0.154	0.141	0.074	0.176	-0.014	-0.012	-0.072	-0.142	0.035	0.072	0.078	-0.044	0.619	0.01	0.002	0.01	-0.006
CD100um glrlm RunVariance	0.089	-0.074	0.675	-0.172	-0.13	-0.057	0.084	-0.009	-0.211	0.005	-0.131	0.062	-0.009	0.249	-0.154	0.192	0.223	0.036	0.07
CD100um glszm SALGLE	-0.8	-0.127	0.02	-0.081	0.018	-0.081	-0.02	-0.019	0.144	-0.061	0.098	0.035	0.12	0.045	-0.075	-0.011	0.014	0.242	0.061
CD100um glszm ZoneEntropy	0.766	0.315	0.114	0.161	0.031	0.07	0.099	0.004	-0.024	-0.119	0.093	0.197	0.031	0.088	-0.044	-0.049	0	0.109	0.006
CD100um gldm LDHGLE	0.491	0.258	0.262	0.385	0.015	0.103	0.018	0.15	0.058	0.085	-0.056	0.082	0.445	0.04	0.068	-0.108	-0.022	0.012	0.051
CD100um gldm LDLGLE	-0.112	0.033	0.845	-0.097	-0.042	-0.013	0.065	-0.019	-0.121	-0.038	-0.093	0.037	0.008	0.138	-0.074	0.105	0.096	0.055	0.031
CD100um gldm SmallDependenceEmphasis	-0.224	0.445	-0.376	0.155	0.095	0.154	-0.033	-0.009	-0.03	-0.11	0.044	0.066	0.064	-0.091	0.582	-0.02	-0.062	0.003	-0.02
CD100um gldm SDHGLE	0.46	0.342	0.273	0.36	0.016	0.114	0.01	0.131	0.063	0.106	-0.068	0.069	0.444	0.026	0.087	-0.093	0.01	0.015	0.037
CD100um gldm SDLGLE	-0.801	-0.111	0.118	-0.078	0.025	-0.068	-0.01	-0.016	0.121	-0.079	0.086	0.044	0.12	0.048	-0.041	-0.002	0.019	0.245	0.076
CD100um ngtdm Busyness	0.144	-0.488	-0.08	-0.169	0.049	0.099	-0.004	0.662	-0.082	0.068	-0.085	-0.065	-0.098	-0.075	-0.054	0.064	0.072	-0.017	0.078
CD100um ngtdm Coarseness	-0.807	0.262	-0.068	-0.054	-0.083	-0.073	-0.061	-0.091	-0.02	-0.01	0.14	0.138	0.01	0.002	0.022	-0.082	-0.128	0.029	-0.114
CD100um ngtdm Complexity	0.498	0.445	0.388	0.144	-0.015	0.136	0.102	0.229	0.13	0.085	0.129	0.025	0.197	0.074	0.141	-0.044	0.033	0.072	0.079
CD100um ngtdm Contrast	-0.443	0.701	0.165	-0.003	0.03	-0.003	-0.006	-0.103	0.096	-0.039	0.182	0.157	-0.024	0.032	0.005	-0.032	-0.003	0.008	0.081
CD100um ngtdm Strength	-0.224	0.575	0.113	-0.131	-0.226	-0.03	0.033	-0.166	0.104	0.051	0.504	-0.058	-0.115	0.07	0.005	-0.085	0.035	-0.093	-0.045
CD150um firstorder 90Percentile	0.519	0.337	0.081	0.621	-0.01	0.055	0.07	0.022	0.033	-0.057	0.111	0.15	0.049	0.02	0.038	-0.033	-0.011	0.078	0.011
CD150um firstorder InterquartileRange	0.351	0.558	0.157	0.064	-0.107	0.063	0.03	-0.085	0.098	0.02	0.197	0.456	0.132	-0.138	-0.013	-0.067	-0.014	0.106	0.014
CD150um firstorder Kurtosis	0.08	-0.197	-0.017	-0.14	0.02	-0.141	0.156	0.018	0.041	0.018	-0.043	-0.739	-0.031	-0.009	0.1	-0.029	-0.016	0.051	-0.06
CD150um firstorder Maximum	0.671	0.315	0.061	0.409	-0.043	0.054	0.152	0.108	0.02	-0.055	0.133	-0.02	0.00E+00	0.012	0.122	-0.042	-0.048	0.127	0.048
CD150um firstorder MeanAbsoluteDeviation	0.384	0.565	0.396	0.1	-0.103	0.075	0.07	-0.071	0.082	-0.003	0.25	0.275	0.09	-0.044	0.002	-0.066	0.01	0.088	0.012
CD150um firstorder Minimum	0.065	0.024	-0.314	0.629	0.061	-0.088	-0.083	-0.121	-0.008	-0.125	0.107	0.099	-0.457	0.062	0.004	-0.074	0.053	-0.02	0.04
CD150um firstorder Range	0.679	0.322	0.205	0.149	-0.072	0.097	0.198	0.168	0.025	-0.002	0.092	-0.066	0.208	-0.015	0.126	-0.011	-0.074	0.142	0.032
CD150um firstorder Skewness	-0.084	-0.056	-0.214	-0.693	-0.135	-0.102	0.018	-0.093	0.109	0.172	0.255	-0.21	-0.172	-0.024	0.012	-0.088	-0.049	0.195	0.01
CD150um firstorder Uniformity	-0.796	-0.275	0.053	-0.167	-0.019	-0.1	-0.073	-0.003	0.114	0.169	-0.033	-0.139	0.009	-0.002	-0.028	0.022	0.007	0.02	0.053
CD150um glcm JointAverage	0.52	0.275	0.217	0.476	-0.024	0.12	0.144	0.144	0.011	0.001	-0.069	0.029	0.358	-0.025	0.04	0.027	-0.067	0.048	-0.034

Pathomic feature name	***P_F1***	***P_F2***	***P_F3***	***P_F4***	***P_F5***	***P_F6***	***P_F7***	***P_F8***	***P_F9***	***P_F10***	***P_F11***	***P_F12***	***P_F13***	***P_F14***	***P_F15***	***P_F16***	***P_F17***	***P_F18***	***P_F19***
CD150um glcm ClusterProminence	0.129	0.208	0.832	0.004	-0.088	0.084	0.073	-0.025	0.08	-0.08	0.178	-0.033	0.008	-0.026	0.048	-0.032	0.051	-0.034	0.019
CD150um glcm ClusterShade	0.011	-0.045	-0.778	-0.149	-0.173	-0.027	-0.022	-0.019	0.115	0.041	0.313	0.009	-0.077	0.037	0.106	0.033	0.146	0.053	0.077
CD150um glcm ClusterTendency	0.322	0.362	0.636	0.004	-0.122	0.079	0.085	-0.044	0.113	-0.023	0.316	0.162	0.063	-0.033	0.048	-0.056	0.037	0.054	0.034
CD150um glcm Contrast	0.052	0.772	0.336	0.122	-0.056	0.129	0.059	-0.099	0.121	0.119	-0.046	-0.028	0.077	-0.107	0.006	-0.003	0.072	-0.017	0.053
CD150um glcm Correlation	0.642	-0.186	0.168	-0.106	-0.175	-0.135	0.077	0.087	-0.03	0.133	0.319	0.226	-0.028	0.064	-0.218	-0.092	-0.008	-0.039	-0.09
CD150um glcm DifferenceAverage	0.008	0.851	0.177	0.099	-0.019	0.111	0.02	-0.098	0.071	0.07	-0.058	0.041	0.07	-0.061	0.063	0.016	-0.029	-0.019	0.019
CD150um glcm DifferenceEntropy	0.662	0.485	0.186	0.146	0.064	0.135	0.047	-0.105	0.071	-0.06	-0.11	-0.025	0.085	0.019	0.064	0.059	0.049	0.006	0.047
CD150um glcm DifferenceVariance	0.132	0.653	0.487	0.165	-0.08	0.121	0.073	-0.125	0.125	0.065	0.034	-0.082	0.063	-0.088	0.014	-0.03	0.148	0.017	0.048
CD150um glcm JointEnergy	-0.846	0.125	0.073	-0.026	-0.002	-0.013	-0.061	0.013	-0.01	-0.092	0.079	0.136	0.099	0.01	0.144	-0.033	-0.029	0.13	0.068
CD150um glcm Imc1	0.566	-0.533	0.006	0.053	0.1	0	0.028	0.351	-0.133	0.019	-0.149	-0.147	0.023	-0.027	-0.002	0.082	0.055	-0.059	0.084
CD150um glcm Imc2	-0.199	0.271	0.073	-0.039	-0.152	-0.074	-0.015	-0.741	0.077	0.003	0.05	0.164	0.042	0.081	0.026	-0.073	-0.059	0.132	-0.038
CD150um glcm Idm	0.132	-0.704	0.227	-0.064	-0.092	0.025	-0.035	0.022	0.009	0.171	0.152	-0.109	-0.039	-0.017	-0.159	-0.055	0.347	-0.003	-0.052
CD150um glcm Idmn	0.784	-0.278	-0.005	-0.021	-0.024	-0.051	0.127	0.084	-0.033	0.099	0.079	-0.178	-0.016	0.02	-0.145	0.022	-0.003	-0.038	-0.019
CD150um glcm Id	0.111	-0.772	0.163	-0.064	-0.083	-0.014	-0.011	0.041	-0.008	0.128	0.13	-0.114	-0.047	-0.016	-0.149	-0.055	0.291	0.004	-0.042
CD150um glcm Idn	0.76	-0.337	0.036	0.025	-0.034	-0.027	0.157	0.129	-0.045	0.039	0.118	-0.178	0.02	0.01	-0.079	-0.012	0.033	0.04	-0.004
CD150um glcm InverseVariance	-0.06	-0.756	-0.052	-0.068	0.024	0.026	-0.069	0.04	0.137	0.185	0.065	-0.066	-0.029	0.02	-0.16	-0.051	0.226	0.017	-0.055
CD150um glcm MaximumProbability	-0.48	0.151	0.677	-0.154	-0.076	0.009	-0.034	-0.081	-0.077	-0.027	-0.025	0.059	-0.013	0.134	-0.052	0.066	0.116	0.068	0.047
CD150um glcm SumEntropy	0.879	-0.059	0.102	0.111	-0.015	0.05	0.066	0.057	-0.002	-0.007	0.014	0.101	0.073	0.012	-0.031	0.001	0.042	0.048	0.011
CD150um glcm SumSquares	0.287	0.465	0.613	0.028	-0.116	0.093	0.085	-0.057	0.121	0.004	0.262	0.133	0.07	-0.049	0.042	-0.049	0.047	0.043	0.039
CD150um glrlm ShortRunEmphasis	-0.411	0.484	-0.292	-0.032	0.144	-0.06	0.019	-0.033	0.077	-0.072	-0.266	0.129	0.033	0.111	0.087	0.094	-0.341	0.041	0.002
CD150um glszm GLNU	0.292	-0.202	-0.047	0.053	0.009	0.157	0.062	0.797	-0.059	-0.019	-0.021	-0.004	0.065	0.041	0.002	-0.016	-0.039	0.034	-0.03
CD150um glszm GLNUN	-0.799	-0.274	-0.027	-0.165	-0.012	-0.102	-0.078	0	0.121	0.158	-0.024	-0.136	0.015	-0.005	-0.029	0.016	-0.003	0.028	0.055
CD150um glszm LALGLE	-0.138	0.042	0.699	-0.172	-0.125	-0.071	0.076	-0.02	-0.215	0.025	-0.108	0.113	-0.004	0.229	-0.096	0.187	0.194	0.04	0.028
CD150um glszm LGLZE	-0.723	0.061	0.182	-0.123	-0.12	-0.171	-0.027	-0.023	0.078	0.09	0.158	0.092	0.005	0.043	-0.269	-0.049	0.069	0.043	-0.132
CD150um glszm SAHGLE	0.431	0.267	0.379	0.414	-0.038	0.122	0.144	0.137	0.061	0.039	-0.078	0.013	0.405	-0.021	0.028	0.009	-0.062	0.052	-0.003
CD150um glszm SALGLE	-0.737	0.059	0.03	-0.118	-0.119	-0.176	-0.038	-0.02	0.089	0.084	0.17	0.096	0.006	0.039	-0.266	-0.058	0.063	0.05	-0.131
CD150um gldm DependenceEntropy	0.831	0.06	0.09	0.217	-0.021	0.123	0.07	0.076	-0.077	-0.033	0.057	0.138	0.086	-0.043	0.071	-0.034	0.05	0.052	-0.001
CD150um gldm DependenceNonUniformity	0.317	-0.147	-0.038	0.065	-0.018	0.169	0.062	0.772	-0.036	-0.024	-0.008	0.055	0.107	0.065	0.025	-0.035	-0.065	0.081	-0.051
CD150um gldm DNUN	-0.416	0.462	-0.378	-0.012	0.134	-0.045	-0.002	-0.031	0.13	-0.056	-0.21	0.118	0.021	0.061	0.094	0.056	-0.35	0.029	0.002
CD150um gldm DependenceVariance	0.333	-0.313	0.612	-0.053	-0.114	0.029	0.04	0.016	-0.217	0.024	0.079	-0.077	-0.005	0.041	-0.079	0.025	0.318	-0.001	0.027
CD150um ngtdm Busyness	-0.236	-0.501	0.004	-0.283	0.009	0.015	-0.095	0.446	0.091	0.37	-0.071	-0.044	-0.051	-0.118	-0.025	0.043	0.079	-0.004	0.085
CD150um ngtdm Coarseness	-0.816	0.128	-0.076	-0.067	-0.082	-0.075	-0.04	-0.167	0.066	0.025	0.164	0.097	0.039	0.025	-0.032	-0.073	-0.097	0.041	-0.103
CD150um ngtdm Complexity	0.473	0.383	0.489	0.22	-0.049	0.15	0.08	0.141	0.091	0.063	0.004	0.068	0.254	-0.044	0.091	-0.025	0.008	0.12	0.041

Pathomic feature name	***P_F1***	***P_F2***	***P_F3***	***P_F4***	***P_F5***	***P_F6***	***P_F7***	***P_F8***	***P_F9***	***P_F10***	***P_F11***	***P_F12***	***P_F13***	***P_F14***	***P_F15***	***P_F16***	***P_F17***	***P_F18***	***P_F19***
CD150um ngtdm Contrast	-0.43	0.708	0.096	-0.059	-0.018	-0.061	-0.037	-0.016	0.027	0.06	0.229	0.111	-0.094	0.039	-0.019	-0.036	-0.067	-0.098	0.028
CD150um ngtdm Strength	-0.125	0.613	0.166	-0.029	-0.214	-0.036	0.094	-0.264	0.106	-0.004	0.462	0.009	-0.051	0.017	-0.093	-0.091	-0.004	-0.03	-0.057
CD200um firstorder 10Percentile	0.419	0.037	-0.323	0.697	0.021	0.032	0.039	0.126	-0.009	-0.048	-0.02	0.044	0.042	0.045	-0.005	-0.153	-0.004	-0.03	0.077
CD200um firstorder InterquartileRange	0.343	0.536	0.135	0.137	-0.041	0.078	0.033	-0.118	0.091	0.024	0.108	0.502	0.072	-0.147	0.05	-0.052	-0.013	0.156	-0.073
CD200um firstorder Kurtosis	0.172	-0.207	0.02	-0.122	0.087	-0.1	0.072	0.133	0.011	-0.027	0.125	-0.744	0.006	0.008	-0.018	0.039	0.008	-0.023	0.102
CD200um firstorder Maximum	0.649	0.263	0.087	0.464	-0.047	0.062	0.159	0.101	0.008	-0.084	0.163	0.037	0.003	0.038	0.087	-0.054	-0.048	0.145	0.049
CD200um firstorder MAD	0.38	0.538	0.379	0.191	-0.057	0.061	0.066	-0.093	0.082	-0.025	0.254	0.292	0.045	-0.065	0.085	0.063	-0.008	0.148	-0.041
CD200um firstorder Median	0.457	0.19	0.076	0.736	0.015	0.048	0.081	0.072	-0.027	-0.091	-0.088	0.048	0.056	0.008	0.009	-0.032	-0.03	-0.006	-0.028
CD200um firstorder Minimum	0.113	0.13	-0.317	0.599	-0.049	-0.021	0.03	-0.194	-0.004	-0.016	0.086	0.028	-0.299	0.16	-0.017	-0.245	-0.058	0.068	0.079
CD200um firstorder Range	0.68	0.233	0.264	0.213	-0.027	0.081	0.164	0.216	0.011	-0.089	0.142	0.028	0.166	-0.043	0.107	0.07	-0.021	0.125	0.01
CD200um firstorder RMAD	0.367	0.519	0.26	0.173	-0.038	0.049	0.049	-0.134	0.069	-0.005	0.174	0.396	0.057	-0.143	0.087	0.144	-0.061	0.172	-0.103
CD200um firstorder Skewness	-0.16	0.007	-0.178	-0.645	-0.121	-0.066	0.036	-0.129	0.128	0.169	0.359	-0.074	-0.072	0.056	-0.004	-0.187	-0.039	0.246	0.122
CD200um firstorder TotalEnergy	0.32	-0.123	-0.018	0.239	-0.046	0.14	0.052	0.73	0.016	-0.021	0.001	0.052	0.114	0.092	0.011	-0.068	-0.095	0.107	-0.049
CD200um firstorder Uniformity	-0.865	-0.073	0.012	-0.106	-0.034	-0.074	-0.061	0.017	0.033	0.146	0.062	-0.031	0.053	-0.053	0.104	-0.006	-0.063	-0.002	0.044
CD200um firstorder Variance	0.331	0.444	0.576	0.171	-0.066	0.051	0.087	-0.077	0.111	-0.021	0.267	0.135	0.038	-0.064	0.078	0.127	0	0.114	-0.023
CD200um glcm JointAverage	0.528	0.178	0.239	0.481	0.042	0.075	0.08	0.219	-0.008	-0.078	-0.07	0.083	0.286	-0.105	0.029	0.11	0.018	-0.028	-0.077
CD200um glcm ClusterProminence	0.142	0.165	0.778	0.038	-0.127	0.063	0.088	-0.02	0.083	-0.124	0.259	-0.071	-0.01	-0.06	0.085	0.167	0.033	-0.025	-0.022
CD200um glcm ClusterShade	-0.002	0.005	-0.696	-0.181	-0.207	0.011	-0.004	-0.031	0.146	0.057	0.32	0.031	-0.076	0.063	0.081	-0.177	0.207	0.034	0.118
CD200um glcm ClusterTendency	0.336	0.291	0.614	0.072	-0.134	0.051	0.088	-0.056	0.101	-0.085	0.362	0.144	0.047	-0.079	0.097	0.153	0.001	0.081	-0.023
CD200um glcm Contrast	0.065	0.749	0.318	0.166	0.131	0.06	0.086	-0.096	0.13	0.172	-0.116	0.033	-0.044	-0.047	-0.058	0.04	0.021	0.096	-0.023
CD200um glcm Correlation	0.399	-0.46	0.201	-0.087	-0.264	-0.117	-0.022	0.134	-0.039	-0.005	0.395	0.296	0.114	-0.053	0.089	0.009	-0.002	-0.046	0.076
CD200um glcm DifferenceAverage	0.06	0.829	0.188	0.149	0.086	0.062	0.049	-0.118	0.039	0.098	-0.1	0.067	-0.025	-0.042	-0.054	0.01	0.006	0.075	-0.111
CD200um glcm DifferenceEntropy	0.806	0.147	0.174	0.126	0.133	0.118	0.053	-0.083	0.044	-0.057	-0.113	-0.058	0.05	0.02	0.003	0.085	0.078	0.072	-0.015
CD200um glcm DifferenceVariance	0.138	0.647	0.426	0.202	0.18	0.048	0.075	-0.106	0.164	0.149	-0.056	0.011	-0.056	0.019	-0.019	0.067	-0.019	0.125	0.016
CD200um glcm JointEnergy	-0.832	0.17	-0.008	-0.007	-0.043	-0.025	-0.069	0.025	-0.059	-0.087	0.085	0.138	0.095	-0.032	0.152	-0.027	-0.073	0.108	0.014
CD200um glcm JointEntropy	0.762	-0.305	0.053	0.133	0.062	0.074	0.055	0.306	-0.105	-0.013	-0.055	0.032	0.076	-0.007	0.058	0.032	0.037	0.008	0.067
CD200um glcm Imc2	-0.163	0.228	0.07	-0.014	-0.126	-0.104	-0.057	-0.801	0.046	0.007	0.032	0.137	0.003	0.06	0.006	-0.029	-0.035	0.083	-0.005
CD200um glcm Idm	-0.048	-0.746	0.163	-0.067	-0.085	-0.001	-0.025	0.097	0.189	0.291	0.01	-0.074	-0.026	0.032	0.002	0.186	-0.019	-0.041	0.198
CD200um glcm Idmn	0.558	-0.606	0.04	-0.011	-0.076	-0.032	0.092	0.146	-0.004	-0.092	0.129	-0.121	0.135	-0.033	0.074	0.04	0.049	-0.001	0.175
CD200um glcm Id	-0.049	-0.79	0.106	-0.082	-0.071	-0.002	-0.023	0.111	0.167	0.199	0.025	-0.091	-0.009	0.032	-0.001	0.154	-0.004	-0.043	0.204

Pathomic feature name	***P_F1***	***P_F2***	***P_F3***	***P_F4***	***P_F5***	***P_F6***	***P_F7***	***P_F8***	***P_F9***	***P_F10***	***P_F11***	***P_F12***	***P_F13***	***P_F14***	***P_F15***	***P_F16***	***P_F17***	***P_F18***	***P_F19***
CD200um glcm Idn	0.562	-0.578	0.057	0.031	-0.026	-0.001	0.11	0.197	0.046	-0.081	0.151	-0.107	0.107	-0.002	0.095	0.071	0.025	0.028	0.212
CD200um glcm InverseVariance	-0.204	-0.651	-0.014	-0.066	0.031	-0.107	0.013	0.083	0.248	0.359	0.071	-0.059	0.018	0.015	0.146	-0.023	-0.005	0.02	0.269
CD200um glcm MaximumProbability	-0.695	0.131	0.334	-0.105	-0.102	0.026	-0.063	-0.043	0.009	-0.044	-0.054	0.051	0.057	0.028	0.016	0.338	-0.097	0.051	0.046
CD200um glcm SumEntropy	0.844	-0.233	0.101	0.13	0.018	0.066	0.039	0.095	-0.023	-0.005	-0.048	0.074	0.067	-0.01	0.049	0.046	0.017	0.04	0.045
CD200um glcm SumSquares	0.302	0.437	0.603	0.103	-0.081	0.059	0.097	-0.072	0.119	-0.028	0.278	0.131	0.028	-0.08	0.067	0.14	0.007	0.094	-0.025
CD200um glrlm RunVariance	0.119	-0.261	0.299	-0.038	-0.162	-0.013	0.056	0.08	-0.136	0.04	-0.091	-0.003	-0.021	0.074	-0.025	0.724	-0.032	-0.013	0.005
CD200um glszm GLNUN	-0.868	-0.058	-0.026	-0.102	-0.023	-0.066	-0.061	0.02	0.03	0.132	0.066	-0.023	0.054	-0.05	0.111	-0.033	-0.06	0.001	0.045
CD200um glszm LGLZE	-0.783	0.013	0.156	-0.071	-0.036	-0.086	-0.065	-0.018	0.132	0.29	0.124	0.089	-0.017	-0.034	0.176	-0.013	0.018	0.068	0.021
CD200um glszm SmallAreaEmphasis	-0.267	0.642	-0.277	-0.025	0.133	-0.133	0.052	-0.133	-0.009	-0.147	0.029	0.05	0.081	0.002	0.134	-0.19	0.015	0.124	-0.061
CD200um glszm SALGLE	-0.796	0.011	0.055	-0.067	-0.014	-0.088	-0.076	-0.016	0.134	0.287	0.112	0.101	-0.013	-0.038	0.175	-0.048	0.006	0.078	0.021
CD200um glszm ZoneEntropy	0.851	-0.102	0.118	0.187	0.014	0.09	0.048	0.08	-0.026	-0.052	-0.033	0.103	0.047	0	0.017	0.037	0.05	0.089	0.023
CD200um gldm LDHGLE	0.469	0.101	0.389	0.425	0.041	0.085	0.069	0.258	0.021	-0.062	-0.036	0.063	0.312	-0.108	0.034	0.117	-0.006	-0.018	-0.07
CD200um gldm LDLGLE	-0.221	0.015	0.625	-0.056	-0.143	-0.066	0.082	-0.014	-0.089	0.074	0.044	0.004	-0.009	0.057	0.091	0.522	0.056	0.024	-0.014
CD200um gldm LowGrayLevelEmphasis	-0.726	0.032	0.336	-0.075	-0.071	-0.091	-0.024	-0.018	0.076	0.245	0.124	0.076	-0.018	-0.009	0.184	0.155	0.036	0.068	0.015
CD200um gldm SmallDependenceEmphasis	-0.256	0.608	-0.321	-0.009	0.152	-0.116	0.036	-0.126	0.015	-0.128	0.052	0.045	0.077	-0.017	0.123	-0.311	0.028	0.111	-0.056
CD200um ngtdm Busyness	-0.495	-0.294	-0.013	-0.33	0.051	0	-0.043	0.337	0.015	0.436	0.048	-0.047	0.003	-0.08	0.042	-0.034	0.01	0.107	0.076
CD200um ngtdm Coarseness	-0.78	-0.08	-0.043	-0.134	-0.19	-0.084	-0.093	-0.194	0.204	0.038	0.077	0.053	0.065	0.067	-0.104	-0.041	-0.024	0.049	-0.027
CD200um ngtdm Complexity	0.487	0.289	0.538	0.28	0.05	0.089	0.052	0.141	0.06	0.045	-0.028	0.106	0.161	-0.041	0.098	0.029	-0.014	0.201	-0.033
CD200um ngtdm Contrast	-0.475	0.646	-0.009	-0.015	0.011	-0.098	-0.039	0.038	-0.124	0.09	0.229	0.141	-0.064	0.002	-0.006	-0.05	-0.141	-0.112	-0.062
CD200um ngtdm Strength	-0.072	0.399	0.209	0.021	-0.305	-0.018	0.088	-0.305	0.2	-0.121	0.56	0.016	-0.086	-0.009	0.046	0.031	0.12	0.012	0.017

Table S8 Summary of the significantly correlated radiomic-pathomic factors, with radiomic factors associated with ADC. Abbreviations: ADC = Apparent Diffucion Coefficient; F = Factor; FDR = False Discovery Rate; BF = Bayes Factor.

**Radiomic ADC factor name**	**Pathomic factor name**	**ρ**	**FDR q-value**	**BF**
ADC_F5	P_F11	-0.543	6.55×10^{-3}	3.00×10^{2}
ADC_F4	P_F11	-0.538	6.55×10^{-3}	2.55×10^{2}
ADC_F1	P_F5	-0.525	6.55×10^{-3}	1.65×10^{2}
ADC_F1	P_F17	0.516	6.55×10^{-3}	1.24×10^{2}
ADC_F2	P_F14	0.475	1.4×10^{-2}	3.63×10^{1}

Table S9 Summary of the significantly correlated radiomic-pathomic factors, with radiomic factors associated with T1C. Abbreviations: T1C = post-contrast T1; F = Factor; FDR = False Discovery Rate; BF = Bayes Factor.

Radiomic T1C factor name	Pathomic factor name	ρ	FDR q-value	BF
T1C_F2	P_F11	-0.628	2.70×10^{-4}	9.14×10^{3}
T1C_F5	P_F8	-0.537	6.21×10^{-3}	2.45×10^{2}

4 Radiopathomic analysis with higher-order features

4.1 Materials and methods

Further higher order features were explored by extracting the first and second order features following application of wavelet and local binary pattern (LBP) filters to the images. The mathematical definition of each of the radiomic features can be found within the PyRadiomics documentation (https://pyradiomics.readthedocs.io/en/latest/features.html). The extracted higher-order radiomic features (from ADC and T1C) included (1) 728 wavelet features in frequency channels LHL, LLH, HHH, HLH, HLL,HHL, LHH and LLL, where L and H are low- and high-pass filters, respectively; (2) 353 Local Binary Pattern (2D and 3D). The extracted higher-order pathomic features included (1) 364 2D wavelet features in frequency channels LL, LH, HL and HH; (2) 84 Local Binary Pattern (2D). A first step of feature selection was performed separately for wavelet features and LBP features by means of a correlation filter based on the absolute values of pairwise Spearman's correlation (ρ) coefficient to reduce feature redundancy. Threshold for ρ was set to 0.9. Then, to check for redundancies among features belonging to the different groups, the same correlation filter was applied to the whole feature set passed the first step of feature selection. The second step was performed also considering original features that passed the feature selection step described in paragraph 2.6.1 in the main text. Correlation analysis and factor analysis were performed according to 2.6.1 and 2.6.2 paragraphs in the main text.

4.2 Results

4.2.1 Correlation analysis

Concerning ADC radiomic features, the first correlation filter step reduced the feature set from 91, 728 and 353 to 46, 182 and 143, respectively for original, wavelet and LBP. The second step of correlation filter among features belonging to different groups reduced the ADC radiomic feature set to 340. Concerning T1C radiomic features, the first correlation filter step reduced the feature set from 91, 728 and 353 to 53, 202 and 135, respectively for original, wavelet and LBP. The second step of correlation filter among features belonging to different groups reduced the T1C radiomic feature set to 362. On the other hand, pathomic features were reduced from 429, 1456 and 336 to 232, 362 and 115, respectively for original, wavelet and LBP. The second step of correlation filter among features belonging to different groups reduced the pathomic feature set to 352. Radiopathomic analysis between selected ADC radiomic features and pathomic features revealed 671 significant correlations (based on adjusted p-values after FDR correction), of which 154 negative correlations ($-0.753 < \rho < -0.501$, $77.5 < BF < 1.74\times10^{7}$) and 517 positive correlations ($0.501 < \rho < 0.785$, $21.6563 < BF < 6.461\times10^{5}$). Among features constituting the ADC-radiopathomic couples showing significant cross-scale associations, ADC radiomic features included 6 firstorder

features, 9 texture features, 119 wavelet and 34 LBP features, while pathomic features included 11 features from detection measurements and 43 cell-density map features, of which 4 from 50μm resolution (2 texture, 2 wavelet), 10 from 100μm resolution (7 lbp and 3 texture), 11 from 150μm resolution (3 texture, 2 wavelet and 2 lbp) and 18 from 200μm resolution (1 firstorder, 1 texture, 13 wavelet, 3 lbp).
Concerning ADC pipeline, most of the strong relationships (250/350) involved features from cell density maps, of which 211 correspond to associations with ADC wavelet features, 8 with ADC LBP, 5 texture and 4 firstorder ADC features. The remaining strongest associations (100/350) involved intranuclear Haralick texture features (Haralick Angular Second Moment F0 and Information measure of correlation 2 F12) with ADC wavelet features (93 associations), firstorder and lbp features (3 and 1 associations, respectively).
Radiopathomic analysis between selected T1C radiomic features and pathomic features revealed 127 significant correlations (based on adjusted p-values after FDR correction), of which 54 negative correlations ($-0.762<\rho< -0.55$, $384.99 < BF < 3.61\times10^7$) and 73 positive correlations ($0.553<\rho< 0.778$, $422.44< BF <3.79\times10^8$). Among features constituting the T1C-radiopathomic couples showing significant cross-scale associations, T1C radiomic features included 3 firstorder features, 2 texture features, 11 LBP and 39 wavelet features, while pathomic features included 3 features from detection measurements and 25 cell-density map features, of which 9 from 50μm resolution (2 texture, 7 wavelet), 4 from 100μm resolution (2 texture, 2 LBP), 3 from 150μm resolution (2 texture, 1 wavelet) and 9 from 200μm resolution (1 firstorder, 2 texture, 5 wavelet, 1 LBP).
Concerning T1C pipeline, similar findings were observed, with most of the strongest associations found between cell density map features and T1C wavelet (33/57), firstorder (7/57), and LBP (3/57). The remaining three associations involving the same two intranuclear Haralick texture features observed in the ADC results and two T1C wavelet features. Similar findings, concerning both ADC and T1C radiopathomic tasks, were observed for significant moderate correlations.
Figure S1 and S2 show the resulting correlation heatmaps for the strongest radiopathomic associations ($|\rho| \geq 0.6$), displaying Spearman's ρ between radiomic features (from ADC and T1C, respectively) and pathomic features.
The entire set of significant radiopathomic associations sorted by ρ strength are presented in Table S10 and S11.

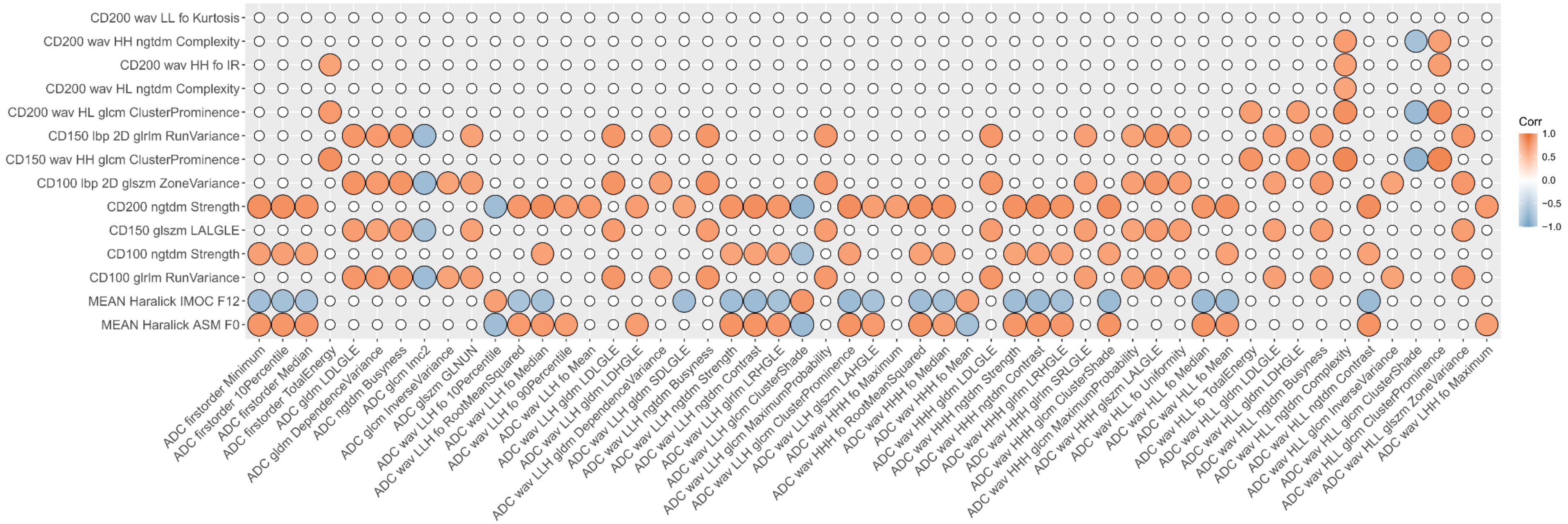

CD200 wav LL fo Kurtosis
CD200 wav HH ngtdm Complexity
CD200 wav HH fo IR
CD200 wav HL ngtdm Complexity
CD200 wav HL glcm ClusterProminence
CD150 lbp 2D glrlm RunVariance
CD150 wav HH glcm ClusterProminence
CD100 lbp 2D glszm ZoneVariance
CD200 ngtdm Strength
CD150 glszm LALGLE
CD100 ngtdm Strength
CD100 glrlm RunVariance
MEAN Haralick IMOC F12
MEAN Haralick ASM F0
Corr
1.0
0.5
0.0
−0.5
−1.0

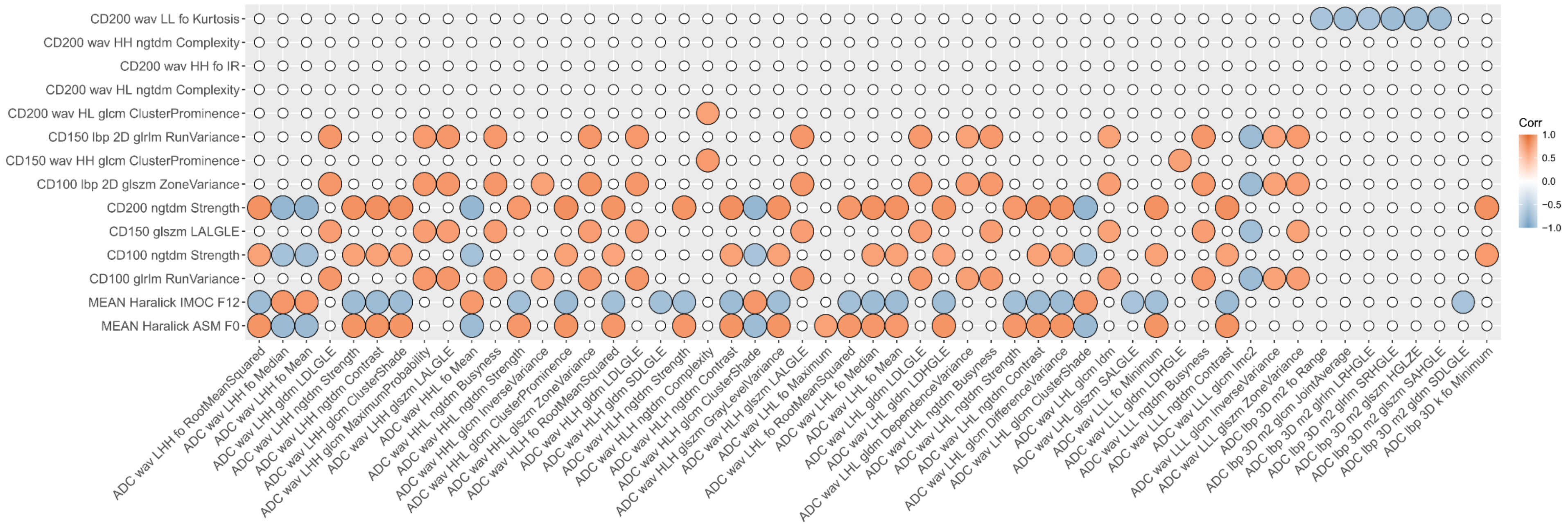

CD200 wav LL fo Kurtosis
CD200 wav HH ngtdm Complexity
CD200 wav HH fo IR
CD200 wav HL ngtdm Complexity
CD200 wav HL glcm ClusterProminence
CD150 lbp 2D glrlm RunVariance
CD150 wav HH glcm ClusterProminence
CD100 lbp 2D glszm ZoneVariance
CD200 ngtdm Strength
CD150 glszm LALGLE
CD100 ngtdm Strength
CD100 glrlm RunVariance
MEAN Haralick IMOC F12
MEAN Haralick ASM F0
Corr
1.0
0.5
0.0
−0.5
−1.0

Figure S1 Radiopathomic analysis between ADC radiomic features and pathomic features. Correlation matrix filtered from nonsignificant correlations and moderate significant correlations (rows and columns with nonsignificant and moderate significant values were deleted, while nonsignificant values surviving were set to zero). For an easier interpretation, the correlation matrix was splitted in two submatrices. Abbreviations: ADC = Apparent Diffusion Coefficient; LALGLE = Large Area Low Gray Level Emphasis; LDLGLE = Large Dependence Low Gray Level Emphasis; LDHGLE = Large Dependence High Gray Level Emphasis; SDLGLE = Small Dependence Low Gray Level Emphasis; LALGLE = Large Area Low Gray Level Emphasis; GLNUN = Gray level non uniformity normalized; SZNU = Size Zone Non-Uniformity; glcm = gray level co-occurrence matrix; gldm = Gray Level Dependence Matrix; glszm = Gray Level Size Zone Matrix; ngtdm = Neighbouring Gray Tone Difference Matrix; glrlm = Gray Level Run Length Matrix; wav = wavelet; lbp = local binary pattern; L = Low-pass filter; H = High-pass filter.

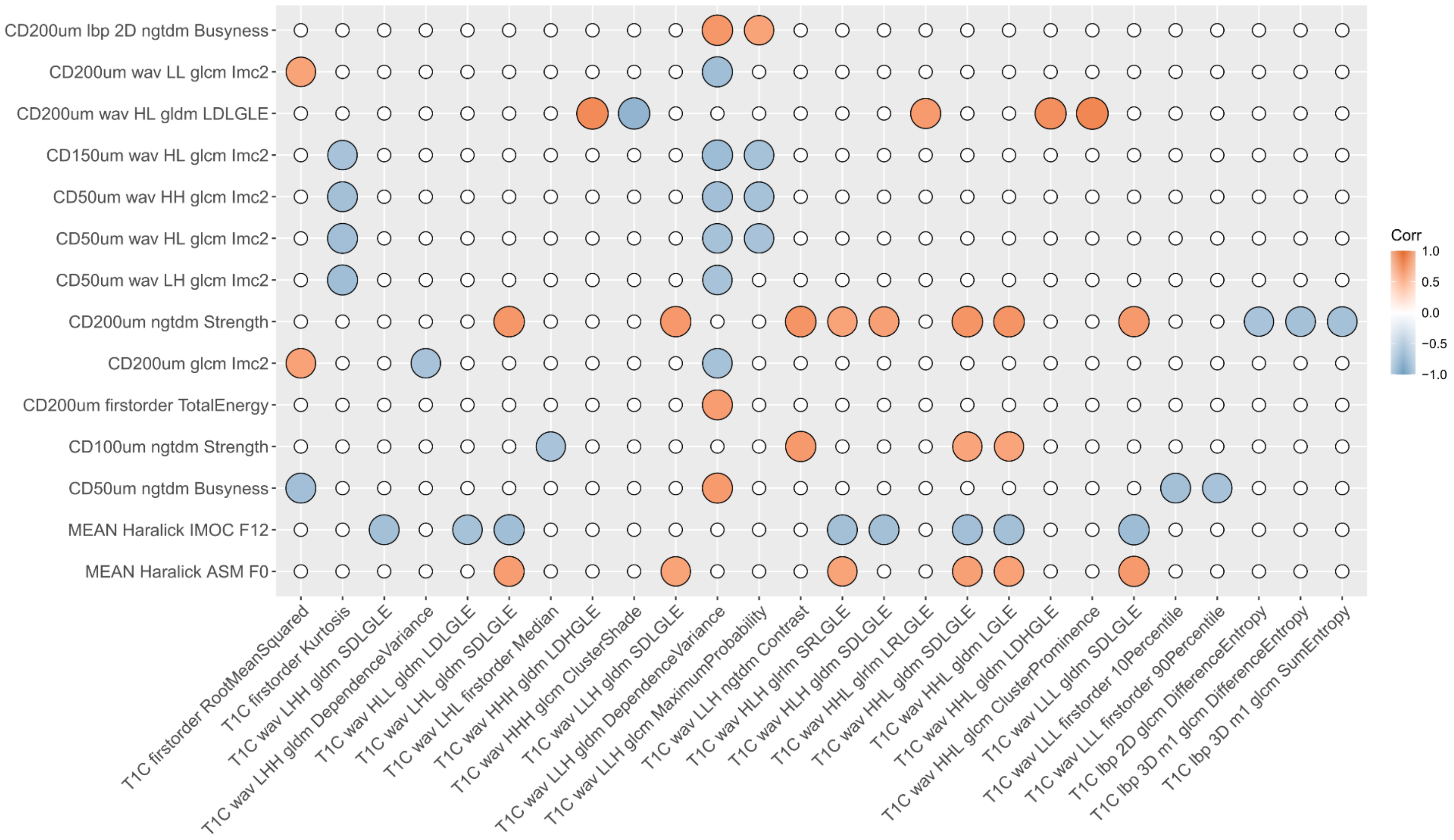

Figure S2 Radiopathomic analysis between T1C radiomic features and pathomic features. Correlation matrix filtered from nonsignificant correlations and moderate significant correlations (rows and columns with nonsignificant and moderate significant values were deleted, while nonsignificant values surviving were set to zero). Abbreviations: T1C = post-contrast T1; LDLGLE = Large Dependence Low Gray Level Emphasis; LDHGLE = Large Dependence High Gray Level Emphasis; SDLGLE = Small Dependence Low Gray Level Emphasis; LALGLE = Large Area Low Gray Level Emphasis; GLNUN = Gray level non uniformity normalized; ASM = Angular Second Moment; IMOC = Information Measure of Correlation; glcm = gray level co-occurrence matrix; gldm = Gray Level Dependence Matrix; glszm = Gray Level Size Zone Matrix; ngtdm = Neighbouring Gray Tone Difference Matrix; glrlm = Gray Level Run Length Matrix; wav = wavelet; lbp = local binary pattern; L = Low-pass filter; H = High-pass filter.

Table S9 Summary of the significantly correlated radiomic-pathomic features, with radiomic features extracted from ADC. Abbreviations: ADC = Apparent Diffusion Coefficient; BF = Bayes Factor; LALGLE = Large Area Low Gray Level Emphasis; LDLGLE = Large Dependence Low Gray Level Emphasis; LDHGLE = Large Dependence High Gray Level Emphasis; SDLGLE = Small Dependence Low Gray Level Emphasis; LALGLE = Large Area Low Gray Level Emphasis; GLNUN = Gray level non uniformity normalized; SZNU = Size Zone Non-Uniformity; ASM = Angular Second Moment; IMOC = Information Measure of Correlation; glcm = gray level co-occurrence matrix; gldm = Gray Level Dependence Matrix; glszm = Gray Level Size Zone Matrix; ngtdm = Neighbouring Gray Tone Difference Matrix; glrlm = Gray Level Run Length Matrix; wav = wavelet; lbp = local binary pattern; L = Low-pass filter; H = High-pass filter.

**Radiomic feature name**	**Pathomic feature name**	**ρ**	**Q-value**	**BF**
ADC wav HLL glcm ClusterProminence	CD150um wav HH glcm ClusterProminence	3.01×10^{-6}	0.785	2.77×10^{8}
ADC wav HLL ngtdm Complexity	CD150um wav HH glcm ClusterProminence	3.01×10^{-6}	0.782	2.14×10^{8}
ADC wav LHH firstorder Mean	CD200um ngtdm Strength	1.31×10^{-5}	-0.753	1.74×10^{7}
ADC wav HLL glcm ClusterShade	CD150um wav HH glcm ClusterProminence	1.31×10^{-5}	-0.747	1.15×10^{7}
ADC wav LHH firstorder Median	CD200um ngtdm Strength	1.31×10^{-5}	-0.742	7.89×10^{6}
ADC wav HLL glcm ClusterProminence	CD200um wav HL glcm ClusterProminence	1.31×10^{-5}	0.742	7.58×10^{6}
ADC wav LLH glcm ClusterProminence	CD200um ngtdm Strength	1.31×10^{-5}	0.741	7.17×10^{6}
ADC wav LHL glcm ClusterShade	CD200um ngtdm Strength	1.31×10^{-5}	-0.74	6.87×10^{6}
ADC wav LHH ngtdm Contrast	CD200um ngtdm Strength	1.31×10^{-5}	0.74	6.62×10^{6}
ADC wav LHL ngtdm Contrast	CD200um ngtdm Strength	1.31×10^{-5}	0.74	6.62×10^{6}
ADC wav LLH ngtdm Contrast	CD200um ngtdm Strength	1.31×10^{-5}	0.74	6.60×10^{6}
ADC wav HHH ngtdm Contrast	CD200um ngtdm Strength	1.31×10^{-5}	0.74	6.58×10^{6}
ADC wav HLH ngtdm Contrast	CD200um ngtdm Strength	1.31×10^{-5}	0.74	6.54×10^{6}
ADC wav LLL ngtdm Contrast	CD200um ngtdm Strength	1.31×10^{-5}	0.74	6.49×10^{6}
ADC wav LHH glcm ClusterShade	CD200um ngtdm Strength	1.31×10^{-5}	0.74	6.49×10^{6}

**Radiomic feature name**	**Pathomic feature name**	**ρ**	**Q-value**	**BF**
ADC wav HHH glcm ClusterShade	CD200um ngtdm Strength	1.31×10^{-5}	0.739	6.32×10^{6}
ADC wav HHL glcm ClusterProminence	CD200um ngtdm Strength	1.31×10^{-5}	0.739	6.24×10^{6}
ADC wav HLL ngtdm Contrast	CD200um ngtdm Strength	1.31×10^{-5}	0.739	6.20×10^{6}
ADC wav LLH glcm ClusterShade	CD200um ngtdm Strength	1.31×10^{-5}	-0.738	5.88×10^{6}
ADC wav LLH firstorder Median	CD200um ngtdm Strength	1.32×10^{-5}	0.737	5.52×10^{6}
ADC wav HHL firstorder Mean	CD200um ngtdm Strength	1.55×10^{-5}	-0.734	4.40×10^{6}
ADC wav LHL glcm DifferenceVariance	CD200um ngtdm Strength	1.55×10^{-5}	0.734	4.33×10^{6}
ADC lbp 3D k firstorder Minimum	CD200um ngtdm Strength	1.68×10^{-5}	0.732	3.83×10^{6}
ADC wav LHL firstorder Median	CD200um ngtdm Strength	1.72×10^{-5}	0.73	3.36×10^{6}
ADC wav LLL firstorder Minimum	CD200um ngtdm Strength	1.72×10^{-5}	0.73	3.34×10^{6}
ADC wav LHH firstorder RootMeanSquared	CD200um ngtdm Strength	1.72×10^{-5}	0.73	3.17×10^{6}
ADC firstorder Minimum	CD200um ngtdm Strength	1.72×10^{-5}	0.729	3.15×10^{6}
ADC wav HLL ngtdm Complexity	CD200um wav HL glcm ClusterProminence	1.72×10^{-5}	0.729	3.11×10^{6}
ADC firstorder TotalEnergy	CD150um wav HH glcm ClusterProminence	1.78×10^{-5}	0.728	2.87×10^{6}
ADC wav LHL firstorder Mean	CD200um ngtdm Strength	1.78×10^{-5}	0.728	2.76×10^{6}
ADC wav HLL firstorder Mean	CD200um ngtdm Strength	1.78×10^{-5}	0.727	2.73×10^{6}
ADC wav HHH firstorder RootMeanSquared	CD200um ngtdm Strength	1.85×10^{-5}	0.726	2.52×10^{6}
ADC wav HLH glcm ClusterShade	CD200um ngtdm Strength	1.85×10^{-5}	-0.726	2.47×10^{6}
ADC firstorder 10Percentile	CD200um ngtdm Strength	1.86×10^{-5}	0.725	2.36×10^{6}
ADC wav LHH ngtdm Strength	CD200um ngtdm Strength	1.86×10^{-5}	0.725	2.32×10^{6}
ADC wav HHH firstorder Median	CD200um ngtdm Strength	2.07×10^{-5}	0.723	2.04×10^{6}
ADC wav HLH glszm GrayLevelVariance	CD200um ngtdm Strength	2.18×10^{-5}	0.722	1.85×10^{6}
ADC wav HHH ngtdm Strength	CD200um ngtdm Strength	2.18×10^{-5}	0.722	1.84×10^{6}
ADC wav LLH ngtdm Strength	CD200um ngtdm Strength	4.93×10^{-5}	0.709	8.19×10^{5}
ADC wav LHL gldm LDHGLE	CD200um ngtdm Strength	5.15×10^{-5}	0.708	7.62×10^{5}
ADC wav HLL firstorder TotalEnergy	CD150um wav HH glcm ClusterProminence	5.15×10^{-5}	0.708	7.49×10^{5}
ADC wav HLH ngtdm Strength	CD200um ngtdm Strength	5.28×10^{-5}	0.707	7.15×10^{5}
ADC wav LHH firstorder Mean	MEAN ODSum Haralick ASM F0	6.09×10^{-5}	-0.704	5.80×10^{5}
ADC wav LLH glcm ClusterProminence	MEAN ODSum Haralick ASM F0	6.09×10^{-5}	0.704	5.71×10^{5}
ADC wav LHL glcm DifferenceVariance	MEAN ODSum Haralick ASM F0	6.09×10^{-5}	0.703	5.27×10^{5}
ADC wav HLL gldm LDHGLE	CD150um wav HH glcm ClusterProminence	6.09×10^{-5}	0.701	4.93×10^{5}

**Radiomic feature name**	**Pathomic feature name**	**ρ**	**Q-value**	**BF**
ADC wav HHH glrlm LRHGLE	CD200um ngtdm Strength	6.09×10^{-5}	0.701	4.81×10^{5}
ADC wav HHL glcm ClusterProminence	MEAN ODSum Haralick ASM F0	6.09×10^{-5}	0.701	4.66×10^{5}
ADC wav HLL glcm ClusterShade	CD200um wav HL glcm ClusterProminence	6.09×10^{-5}	-0.7	4.63×10^{5}
ADC wav LHH firstorder RootMeanSquared	MEAN ODSum Haralick ASM F0	6.09×10^{-5}	0.7	4.45×10^{5}
ADC wav LHH firstorder Median	MEAN ODSum Haralick ASM F0	6.09×10^{-5}	-0.699	4.35×10^{5}
ADC wav HLH glszm GrayLevelVariance	MEAN ODSum Haralick ASM F0	6.09×10^{-5}	0.699	4.23×10^{5}
ADC firstorder Median	CD200um ngtdm Strength	6.09×10^{-5}	0.698	4.06×10^{5}
ADC wav LHL glcm ClusterShade	MEAN ODSum Haralick ASM F0	6.09×10^{-5}	-0.698	4.06×10^{5}
ADC wav LHL gldm LDHGLE	MEAN ODSum Haralick ASM F0	6.09×10^{-5}	0.698	4.00×10^{5}
ADC wav HLL ngtdm Contrast	MEAN ODSum Haralick ASM F0	6.09×10^{-5}	0.697	3.83×10^{5}
ADC wav LLL firstorder Minimum	MEAN ODSum Haralick ASM F0	6.09×10^{-5}	0.697	3.78×10^{5}
ADC wav HLH ngtdm Contrast	MEAN ODSum Haralick ASM F0	6.09×10^{-5}	0.697	3.76×10^{5}
ADC wav LLL ngtdm Contrast	MEAN ODSum Haralick ASM F0	6.09×10^{-5}	0.697	3.75×10^{5}
ADC wav LLH ngtdm Contrast	MEAN ODSum Haralick ASM F0	6.09×10^{-5}	0.697	3.72×10^{5}
ADC wav HHH firstorder RootMeanSquared	MEAN ODSum Haralick ASM F0	6.09×10^{-5}	0.697	3.72×10^{5}
ADC wav HHH ngtdm Contrast	MEAN ODSum Haralick ASM F0	6.09×10^{-5}	0.697	3.71×10^{5}
ADC wav LHL ngtdm Contrast	MEAN ODSum Haralick ASM F0	6.09×10^{-5}	0.697	3.70×10^{5}
ADC wav LHH ngtdm Contrast	MEAN ODSum Haralick ASM F0	6.09×10^{-5}	0.697	3.70×10^{5}
ADC gldm LDLGLE	CD100um lbp 2D glszm ZoneVariance	6.09×10^{-5}	0.697	3.67×10^{5}
ADC wav LHH ngtdm Strength	MEAN ODSum Haralick ASM F0	6.09×10^{-5}	0.697	3.67×10^{5}
ADC wav LLH ngtdm Strength	MEAN ODSum Haralick ASM F0	6.09×10^{-5}	0.697	3.64×10^{5}
ADC wav LLH ngtdm Busyness	CD100um lbp 2D glszm ZoneVariance	6.09×10^{-5}	0.696	3.61×10^{5}
ADC wav LHH glcm ClusterShade	MEAN ODSum Haralick ASM F0	6.09×10^{-5}	0.696	3.58×10^{5}
ADC wav HLL firstorder Median	MEAN ODSum Haralick ASM F0	6.09×10^{-5}	0.696	3.58×10^{5}
ADC wav HLH firstorder RootMeanSquared	CD200um ngtdm Strength	6.09×10^{-5}	0.696	3.50×10^{5}
ADC wav HHH glcm ClusterShade	MEAN ODSum Haralick ASM F0	6.09×10^{-5}	0.696	3.43×10^{5}
ADC wav LLH glcm ClusterShade	MEAN ODSum Haralick ASM F0	6.09×10^{-5}	-0.695	3.40×10^{5}
ADC wav LLH gldm LDLGLE	CD100um lbp 2D glszm ZoneVariance	6.09×10^{-5}	0.695	3.40×10^{5}
ADC ngtdm Busyness	CD100um lbp 2D glszm ZoneVariance	6.09×10^{-5}	0.695	3.39×10^{5}

**Radiomic feature name**	**Pathomic feature name**	**ρ**	**Q-value**	**BF**
ADC wav HHL glszm ZoneVariance	CD100um lbp 2D glszm ZoneVariance	6.09×10^{-5}	0.695	3.37×10^{5}
ADC wav LHH glszm LALGLE	CD100um lbp 2D glszm ZoneVariance	6.09×10^{-5}	0.695	3.29×10^{5}
ADC wav HHH glszm LALGLE	CD100um lbp 2D glszm ZoneVariance	6.09×10^{-5}	0.695	3.27×10^{5}
ADC wav HLH glszm LALGLE	CD100um lbp 2D glszm ZoneVariance	6.09×10^{-5}	0.695	3.26×10^{5}
ADC wav HLL firstorder Median	CD200um ngtdm Strength	6.09×10^{-5}	0.695	3.23×10^{5}
ADC firstorder Minimum	MEAN ODSum Haralick ASM F0	6.09×10^{-5}	0.694	3.16×10^{5}
ADC wav LHH gldm LDLGLE	CD100um lbp 2D glszm ZoneVariance	6.09×10^{-5}	0.694	3.03×10^{5}
ADC wav LLL glcm Imc2	CD100um lbp 2D glszm ZoneVariance	6.09×10^{-5}	-0.693	3.00×10^{5}
ADC gldm LDLGLE	CD100um glrlm RunVariance	6.09×10^{-5}	0.693	3.00×10^{5}
ADC wav HHH gldm LDLGLE	CD100um lbp 2D glszm ZoneVariance	6.09×10^{-5}	0.692	2.84×10^{5}
ADC wav HHL ngtdm Busyness	CD100um lbp 2D glszm ZoneVariance	6.09×10^{-5}	0.692	2.78×10^{5}
ADC wav LLH ngtdm Busyness	CD100um glrlm RunVariance	6.09×10^{-5}	0.692	2.77×10^{5}
ADC wav HHH ngtdm Strength	MEAN ODSum Haralick ASM F0	6.09×10^{-5}	0.692	2.72×10^{5}
ADC wav HHL glszm ZoneVariance	CD100um glrlm RunVariance	6.09×10^{-5}	0.692	2.71×10^{5}
ADC wav LLH gldm LDLGLE	CD100um glrlm RunVariance	6.09×10^{-5}	0.692	2.69×10^{5}
ADC wav HHH glcm ClusterShade	MEAN ODSum Haralick IMOC2 F12	6.09×10^{-5}	-0.691	2.65×10^{5}
ADC gldm LDLGLE	CD150um lbp 2D glrlm RunVariance	6.09×10^{-5}	0.691	2.64×10^{5}
ADC wav LLH glrlm LRHGLE	CD200um ngtdm Strength	6.09×10^{-5}	0.691	2.63×10^{5}
ADC wav LHH glcm ClusterShade	MEAN ODSum Haralick IMOC2 F12	6.09×10^{-5}	-0.691	2.62×10^{5}
ADC wav HLH gldm LDLGLE	CD100um lbp 2D glszm ZoneVariance	6.09×10^{-5}	0.691	2.61×10^{5}
ADC wav LHL ngtdm Contrast	MEAN ODSum Haralick IMOC2 F12	6.09×10^{-5}	-0.691	2.60×10^{5}
ADC wav LHH ngtdm Contrast	MEAN ODSum Haralick IMOC2 F12	6.09×10^{-5}	-0.691	2.60×10^{5}
ADC wav HLL ngtdm Contrast	MEAN ODSum Haralick IMOC2 F12	6.09×10^{-5}	-0.691	2.60×10^{5}
ADC wav LHH glszm LALGLE	CD100um glrlm RunVariance	6.09×10^{-5}	0.691	2.59×10^{5}
ADC wav LLH ngtdm Contrast	MEAN ODSum Haralick IMOC2 F12	6.09×10^{-5}	-0.691	2.59×10^{5}
ADC wav LLL ngtdm Contrast	MEAN ODSum Haralick IMOC2 F12	6.09×10^{-5}	-0.691	2.59×10^{5}
ADC wav HHH ngtdm Contrast	MEAN ODSum Haralick IMOC2 F12	6.09×10^{-5}	-0.691	2.59×10^{5}
ADC wav HHH glszm LALGLE	CD100um glrlm RunVariance	6.09×10^{-5}	0.691	2.58×10^{5}
ADC wav LLH glcm ClusterShade	MEAN ODSum Haralick IMOC2 F12	6.09×10^{-5}	0.691	2.58×10^{5}
ADC wav HLH ngtdm Contrast	MEAN ODSum Haralick IMOC2 F12	6.09×10^{-5}	-0.691	2.57×10^{5}
ADC wav HLH glszm LALGLE	CD100um glrlm RunVariance	6.09×10^{-5}	0.691	2.56×10^{5}

**Radiomic feature name**	**Pathomic feature name**	**ρ**	**Q-value**	**BF**
ADC wav LHL glcm ClusterShade	MEAN ODSum Haralick IMOC2 F12	6.11×10^{-5}	0.69	2.52×10^{5}
ADC wav HLH glcm ClusterShade	MEAN ODSum Haralick IMOC2 F12	6.11×10^{-5}	0.69	2.48×10^{5}
ADC wav LHL ngtdm Strength	MEAN ODSum Haralick ASM F0	6.11×10^{-5}	0.69	2.45×10^{5}
ADC wav LLL ngtdm Busyness	CD150um lbp 2D glrlm RunVariance	6.11×10^{-5}	0.69	2.43×10^{5}
ADC ngtdm Busyness	CD100um glrlm RunVariance	6.11×10^{-5}	0.69	2.42×10^{5}
ADC wav LHH gldm LDLGLE	CD100um glrlm RunVariance	6.11×10^{-5}	0.69	2.41×10^{5}
ADC ngtdm Busyness	CD150um lbp 2D glrlm RunVariance	6.11×10^{-5}	0.69	2.39×10^{5}
ADC wav HHL glszm ZoneVariance	CD150um lbp 2D glrlm RunVariance	6.11×10^{-5}	0.689	2.38×10^{5}
ADC wav LLL glcm Imc2	CD100um glrlm RunVariance	6.18×10^{-5}	-0.689	2.34×10^{5}
ADC wav HHL firstorder Mean	MEAN ODSum Haralick ASM F0	6.25×10^{-5}	-0.689	2.27×10^{5}
ADC wav HHL glcm ClusterProminence	MEAN ODSum Haralick IMOC2 F12	6.25×10^{-5}	-0.689	2.26×10^{5}
ADC wav HLH ngtdm Strength	MEAN ODSum Haralick ASM F0	6.25×10^{-5}	0.688	2.25×10^{5}
ADC wav HLH firstorder RootMeanSquared	MEAN ODSum Haralick ASM F0	6.25×10^{-5}	0.688	2.21×10^{5}
ADC wav LHH glszm LALGLE	CD150um lbp 2D glrlm RunVariance	6.25×10^{-5}	0.688	2.19×10^{5}
ADC wav LHH firstorder Median	MEAN ODSum Haralick IMOC2 F12	6.25×10^{-5}	0.688	2.19×10^{5}
ADC wav HHH glszm LALGLE	CD150um lbp 2D glrlm RunVariance	6.25×10^{-5}	0.688	2.18×10^{5}
ADC wav HLH glszm LALGLE	CD150um lbp 2D glrlm RunVariance	6.25×10^{-5}	0.688	2.17×10^{5}
ADC wav HHH gldm LDLGLE	CD100um glrlm RunVariance	6.28×10^{-5}	0.688	2.13×10^{5}
ADC wav LHL firstorder RootMeanSquared	MEAN ODSum Haralick ASM F0	6.28×10^{-5}	0.688	2.12×10^{5}
ADC wav LHL firstorder RootMeanSquared	CD200um ngtdm Strength	6.28×10^{-5}	0.687	2.10×10^{5}
ADC wav HHL ngtdm Busyness	CD100um glrlm RunVariance	6.28×10^{-5}	0.687	2.09×10^{5}
ADC wav LLL glcm Imc2	CD150um lbp 2D glrlm RunVariance	6.36×10^{-5}	-0.687	2.05×10^{5}
ADC wav HLH gldm LDLGLE	CD100um glrlm RunVariance	6.41×10^{-5}	0.687	2.01×10^{5}
ADC firstorder 10Percentile	MEAN ODSum Haralick ASM F0	6.41×10^{-5}	0.687	2.01×10^{5}
ADC wav HHL ngtdm Strength	MEAN ODSum Haralick ASM F0	6.47×10^{-5}	0.686	1.97×10^{5}
ADC wav LLH gldm LDLGLE	CD150um lbp 2D glrlm RunVariance	6.49×10^{-5}	0.686	1.95×10^{5}
ADC wav LHL firstorder Median	MEAN ODSum Haralick ASM F0	6.49×10^{-5}	0.686	1.94×10^{5}
ADC wav LLH ngtdm Busyness	CD150um lbp 2D glrlm RunVariance	6.56×10^{-5}	0.686	1.91×10^{5}
ADC wav LHH gldm LDLGLE	CD150um lbp 2D glrlm RunVariance	6.57×10^{-5}	0.686	1.89×10^{5}

**Radiomic feature name**	**Pathomic feature name**	**ρ**	**Q-value**	**BF**
ADC wav LHL glcm DifferenceVariance	MEAN ODSum Haralick IMOC2 F12	6.60×10^{-5}	-0.685	1.87×10^{5}
ADC wav LLH glcm ClusterProminence	MEAN ODSum Haralick IMOC2 F12	6.63×10^{-5}	-0.685	1.85×10^{5}
ADC wav LLL ngtdm Busyness	CD100um lbp 2D glszm ZoneVariance	7.01×10^{-5}	0.684	1.74×10^{5}
ADC wav HLL firstorder Median	MEAN ODSum Haralick IMOC2 F12	7.11×10^{-5}	-0.684	1.70×10^{5}
ADC wav HHL ngtdm Busyness	CD150um lbp 2D glrlm RunVariance	7.11×10^{-5}	0.684	1.69×10^{5}
ADC wav LHL firstorder Median	MEAN ODSum Haralick IMOC2 F12	7.38×10^{-5}	-0.683	1.61×10^{5}
ADC wav HLL glszm ZoneVariance	CD100um lbp 2D glszm ZoneVariance	7.38×10^{-5}	0.683	1.60×10^{5}
ADC wav LHL ngtdm Busyness	CD150um lbp 2D glrlm RunVariance	7.38×10^{-5}	0.683	1.59×10^{5}
ADC wav LHH firstorder Mean	MEAN ODSum Haralick IMOC2 F12	7.38×10^{-5}	0.683	1.58×10^{5}
ADC wav LLH firstorder RootMeanSquared	CD200um ngtdm Strength	7.38×10^{-5}	0.682	1.56×10^{5}
ADC wav LHL firstorder Mean	MEAN ODSum Haralick ASM F0	7.38×10^{-5}	0.682	1.56×10^{5}
ADC wav HLL ngtdm Busyness	CD100um lbp 2D glszm ZoneVariance	7.38×10^{-5}	0.682	1.55×10^{5}
ADC wav HLL glszm ZoneVariance	CD150um lbp 2D glrlm RunVariance	7.38×10^{-5}	0.682	1.55×10^{5}
ADC wav LLH glrlm LRHGLE	MEAN ODSum Haralick ASM F0	7.47×10^{-5}	0.682	1.52×10^{5}
ADC wav HLH gldm LDLGLE	CD150um lbp 2D glrlm RunVariance	7.50×10^{-5}	0.682	1.50×10^{5}
ADC wav HLL glszm ZoneVariance	CD100um glrlm RunVariance	7.55×10^{-5}	0.681	1.48×10^{5}
ADC wav HHH gldm LDLGLE	CD150um lbp 2D glrlm RunVariance	7.55×10^{-5}	0.681	1.48×10^{5}
ADC wav LHL ngtdm Strength	CD200um ngtdm Strength	7.60×10^{-5}	0.681	1.46×10^{5}
ADC wav HLL firstorder Mean	MEAN ODSum Haralick ASM F0	7.62×10^{-5}	0.681	1.45×10^{5}
ADC wav LLL ngtdm Busyness	CD100um glrlm RunVariance	7.65×10^{-5}	0.681	1.43×10^{5}
ADC wav LHL gldm LDLGLE	CD100um lbp 2D glszm ZoneVariance	7.80×10^{-5}	0.68	1.40×10^{5}
ADC wav LHH firstorder RootMeanSquared	MEAN ODSum Haralick IMOC2 F12	7.99×10^{-5}	-0.68	1.36×10^{5}
ADC glcm Imc2	CD100um lbp 2D glszm ZoneVariance	8.02×10^{-5}	-0.68	1.34×10^{5}
ADC lbp 3D m2 glrlm SRHGLE	CD200um wav LL firstorder Kurtosis	8.04×10^{-5}	-0.679	1.33×10^{5}
ADC wav LLH glcm MaximumProbability	CD100um lbp 2D glszm ZoneVariance	8.10×10^{-5}	0.679	1.31×10^{5}
ADC wav HHH glrlm LRHGLE	MEAN ODSum Haralick ASM F0	8.38×10^{-5}	0.679	1.26×10^{5}
ADC wav LHH glcm MaximumProbability	CD100um lbp 2D glszm ZoneVariance	8.38×10^{-5}	0.678	1.26×10^{5}
ADC wav LHL firstorder Mean	MEAN ODSum Haralick IMOC2 F12	8.58×10^{-5}	-0.678	1.22×10^{5}
ADC wav HLH glcm ClusterShade	MEAN ODSum Haralick ASM F0	8.70×10^{-5}	-0.678	1.20×10^{5}
ADC wav LHL ngtdm Busyness	CD100um lbp 2D glszm ZoneVariance	8.95×10^{-5}	0.677	1.16×10^{5}
ADC wav HHL ngtdm Strength	CD200um ngtdm Strength	9.36×10^{-5}	0.676	1.10×10^{5}

**Radiomic feature name**	**Pathomic feature name**	**ρ**	**Q-value**	**BF**
ADC firstorder Median	MEAN ODSum Haralick ASM F0	9.44×10^{-5}	0.676	1.09×10^{5}
ADC wav LHH glcm MaximumProbability	CD100um glrlm RunVariance	9.68×10^{-5}	0.675	1.05×10^{5}
ADC glcm Imc2	CD100um glrlm RunVariance	9.68×10^{-5}	-0.675	1.05×10^{5}
ADC wav LHH ngtdm Strength	MEAN ODSum Haralick IMOC2 F12	9.69×10^{-5}	-0.675	1.04×10^{5}
ADC wav LHL gldm LDLGLE	CD100um glrlm RunVariance	9.90×10^{-5}	0.675	1.02×10^{5}
ADC lbp 3D m2 glszm SAHGLE	CD200um wav LL firstorder Kurtosis	1.01×10^{-4}	-0.674	9.93×10^{4}
ADC wav HHH ngtdm Strength	MEAN ODSum Haralick IMOC2 F12	1.01×10^{-4}	-0.674	9.89×10^{4}
ADC wav HLL ngtdm Busyness	CD100um glrlm RunVariance	1.02×10^{-4}	0.674	9.73×10^{4}
ADC wav LLH glcm MaximumProbability	CD100um glrlm RunVariance	1.02×10^{-4}	0.674	9.71×10^{4}
ADC wav LLH firstorder 10Percentile	CD200um ngtdm Strength	1.02×10^{-4}	-0.674	9.64×10^{4}
ADC wav LHL ngtdm Busyness	CD100um glrlm RunVariance	1.03×10^{-4}	0.673	9.44×10^{4}
ADC wav HHH firstorder RootMeanSquared	MEAN ODSum Haralick IMOC2 F12	1.04×10^{-4}	-0.673	9.37×10^{4}
ADC glcm Imc2	CD150um lbp 2D glrlm RunVariance	1.04×10^{-4}	-0.673	9.28×10^{4}
ADC wav HLL ngtdm Busyness	CD150um lbp 2D glrlm RunVariance	1.04×10^{-4}	0.673	9.25×10^{4}
ADC wav LLH glcm MaximumProbability	CD150um lbp 2D glrlm RunVariance	1.11×10^{-4}	0.672	8.65×10^{4}
ADC lbp 3D m2 glszm HGLZE	CD200um wav LL firstorder Kurtosis	1.24×10^{-4}	-0.67	7.71×10^{4}
ADC wav LHL gldm LDLGLE	CD150um lbp 2D glrlm RunVariance	1.25×10^{-4}	0.67	7.64×10^{4}
ADC wav HLL firstorder Mean	MEAN ODSum Haralick IMOC2 F12	1.29×10^{-4}	-0.669	7.35×10^{4}
ADC wav LLH firstorder 10Percentile	MEAN ODSum Haralick ASM F0	1.38×10^{-4}	-0.668	6.87×10^{4}
ADC wav HHL firstorder Mean	MEAN ODSum Haralick IMOC2 F12	1.49×10^{-4}	0.666	6.34×10^{4}
ADC wav LLH firstorder Median	MEAN ODSum Haralick ASM F0	1.63×10^{-4}	0.664	5.78×10^{4}
ADC wav LLH firstorder RootMeanSquared	MEAN ODSum Haralick ASM F0	1.69×10^{-4}	0.664	5.56×10^{4}
ADC wav HLH glszm GrayLevelVariance	MEAN ODSum Haralick IMOC2 F12	1.78×10^{-4}	-0.663	5.28×10^{4}
ADC wav LLL glszm ZoneVariance	CD150um lbp 2D glrlm RunVariance	1.78×10^{-4}	0.663	5.25×10^{4}
ADC wav LLH firstorder Mean	CD200um ngtdm Strength	1.86×10^{-4}	0.662	5.00×10^{4}
ADC wav LLH glszm LAHGLE	MEAN ODSum Haralick ASM F0	1.92×10^{-4}	0.661	4.82×10^{4}
ADC gldm DependenceVariance	CD150um lbp 2D glrlm RunVariance	1.92×10^{-4}	0.661	4.81×10^{4}
ADC wav HLH ngtdm Complexity	CD150um wav HH glcm ClusterProminence	1.93×10^{-4}	0.661	4.77×10^{4}
ADC firstorder TotalEnergy	CD200um wav HL glcm ClusterProminence	1.93×10^{-4}	0.661	4.74×10^{4}

**Radiomic feature name**	**Pathomic feature name**	**ρ**	**Q-value**	**BF**
ADC wav LHH glcm MaximumProbability	CD150um lbp 2D glrlm RunVariance	1.94×10^{-4}	0.66	4.68×10^{4}
ADC wav LLH ngtdm Strength	MEAN ODSum Haralick IMOC2 F12	2.06×10^{-4}	-0.659	4.40×10^{4}
ADC wav LHL gldm LDHGLE	MEAN ODSum Haralick IMOC2 F12	2.12×10^{-4}	-0.659	4.26×10^{4}
ADC wav LHH firstorder Maximum	CD200um ngtdm Strength	2.27×10^{-4}	0.657	3.98×10^{4}
ADC lbp 3D k firstorder Minimum	CD100um ngtdm Strength	2.50×10^{-4}	0.655	3.61×10^{4}
ADC gldm DependenceVariance	CD100um lbp 2D glszm ZoneVariance	2.50×10^{-4}	0.655	3.60×10^{4}
ADC gldm DependenceVariance	CD100um glrlm RunVariance	2.52×10^{-4}	0.655	3.55×10^{4}
ADC wav HLH gldm LDLGLE	CD150um glszm LALGLE	2.53×10^{-4}	0.655	3.52×10^{4}
ADC wav LLH gldm LDLGLE	CD150um glszm LALGLE	2.55×10^{-4}	0.655	3.47×10^{4}
ADC wav HLH ngtdm Strength	MEAN ODSum Haralick IMOC2 F12	2.66×10^{-4}	-0.654	3.32×10^{4}
ADC wav LLL glszm ZoneVariance	CD100um lbp 2D glszm ZoneVariance	2.67×10^{-4}	0.654	3.28×10^{4}
ADC wav HLL ngtdm Complexity	CD200um wav HH ngtdm Complexity	2.67×10^{-4}	0.654	3.27×10^{4}
ADC firstorder Minimum	MEAN ODSum Haralick IMOC2 F12	2.67×10^{-4}	-0.654	3.27×10^{4}
ADC gldm LDLGLE	CD150um glszm LALGLE	2.76×10^{-4}	0.653	3.14×10^{4}
ADC wav HHH gldm LDLGLE	CD150um glszm LALGLE	2.76×10^{-4}	0.653	3.13×10^{4}
ADC wav LLH firstorder 90Percentile	CD200um ngtdm Strength	2.77×10^{-4}	0.653	3.11×10^{4}
ADC lbp 3D m2 glcm JointAverage	CD200um wav LL firstorder Kurtosis	2.77×10^{-4}	-0.652	3.10×10^{4}
ADC wav LHH gldm LDLGLE	CD150um glszm LALGLE	2.78×10^{-4}	0.652	3.07×10^{4}
ADC wav LLH ngtdm Busyness	CD150um glszm LALGLE	2.83×10^{-4}	0.652	3.00×10^{4}
ADC wav HHL glszm ZoneVariance	CD150um glszm LALGLE	2.98×10^{-4}	0.651	2.85×10^{4}
ADC wav LLH glcm MaximumProbability	CD150um glszm LALGLE	2.99×10^{-4}	0.651	2.82×10^{4}
ADC wav HLH glszm LALGLE	CD150um glszm LALGLE	3.00×10^{-4}	0.65	2.80×10^{4}
ADC wav LHH glszm LALGLE	CD150um glszm LALGLE	3.01×10^{-4}	0.65	2.78×10^{4}
ADC wav HHH glszm LALGLE	CD150um glszm LALGLE	3.01×10^{-4}	0.65	2.77×10^{4}
ADC wav LLH gldm LDHGLE	CD200um ngtdm Strength	3.05×10^{-4}	0.65	2.71×10^{4}
ADC wav HHL ngtdm Strength	MEAN ODSum Haralick IMOC2 F12	3.05×10^{-4}	-0.65	2.71×10^{4}
ADC wav LLH gldm SDLGLE	MEAN ODSum Haralick IMOC2 F12	3.09×10^{-4}	-0.65	2.67×10^{4}
ADC wav HHH glrlm SRLGLE	CD100um lbp 2D glszm ZoneVariance	3.22×10^{-4}	0.649	2.55×10^{4}
ADC wav LLH gldm LDHGLE	MEAN ODSum Haralick ASM F0	3.24×10^{-4}	0.648	2.53×10^{4}
ADC ngtdm Busyness	CD150um glszm LALGLE	3.27×10^{-4}	0.648	2.48×10^{4}
ADC wav HLL firstorder Uniformity	CD100um glrlm RunVariance	3.27×10^{-4}	0.648	2.48×10^{4}

**Radiomic feature name**	**Pathomic feature name**	**ρ**	**Q-value**	**BF**
ADC wav LLL glszm ZoneVariance	CD100um glrlm RunVariance	3.27×10^{-4}	0.648	2.47×10^{4}
ADC wav LLL firstorder Minimum	MEAN ODSum Haralick IMOC2 F12	3.27×10^{-4}	-0.648	2.46×10^{4}
ADC wav LHL glcm Idm	CD100um lbp 2D glszm ZoneVariance	3.27×10^{-4}	0.648	2.45×10^{4}
ADC wav HLL firstorder Uniformity	CD100um lbp 2D glszm ZoneVariance	3.27×10^{-4}	0.648	2.45×10^{4}
ADC wav LHL gldm LDLGLE	CD150um glszm LALGLE	3.36×10^{-4}	0.647	2.37×10^{4}
ADC wav LLL glcm Imc2	CD150um glszm LALGLE	3.37×10^{-4}	-0.647	2.35×10^{4}
ADC wav HLL gldm LDLGLE	CD150um lbp 2D glrlm RunVariance	3.55×10^{-4}	0.646	2.23×10^{4}
ADC wav LHH firstorder Mean	CD100um ngtdm Strength	3.61×10^{-4}	-0.646	2.19×10^{4}
ADC wav HLL ngtdm Busyness	CD150um glszm LALGLE	3.62×10^{-4}	0.645	2.17×10^{4}
ADC wav LHL firstorder RootMeanSquared	MEAN ODSum Haralick IMOC2 F12	3.64×10^{-4}	-0.645	2.15×10^{4}
ADC wav HHL ngtdm Busyness	CD150um glszm LALGLE	3.66×10^{-4}	0.645	2.13×10^{4}
ADC wav HLH gldm SDLGLE	MEAN ODSum Haralick IMOC2 F12	3.76×10^{-4}	-0.644	2.07×10^{4}
ADC wav HHH firstorder Median	MEAN ODSum Haralick ASM F0	3.91×10^{-4}	0.644	1.99×10^{4}
ADC wav LHH glcm MaximumProbability	CD150um glszm LALGLE	3.95×10^{-4}	0.643	1.96×10^{4}
ADC wav LHL glcm Idm	CD100um glrlm RunVariance	3.95×10^{-4}	0.643	1.95×10^{4}
ADC wav LLH glszm LAHGLE	CD200um ngtdm Strength	4.01×10^{-4}	0.643	1.92×10^{4}
ADC wav HLL firstorder TotalEnergy	CD200um wav HL glcm ClusterProminence	4.15×10^{-4}	0.642	1.85×10^{4}
ADC wav LHL ngtdm Strength	MEAN ODSum Haralick IMOC2 F12	4.28×10^{-4}	-0.642	1.79×10^{4}
ADC wav HLL firstorder Uniformity	CD150um glszm LALGLE	4.44×10^{-4}	0.641	1.72×10^{4}
ADC wav LHL ngtdm Busyness	CD150um glszm LALGLE	4.44×10^{-4}	0.641	1.71×10^{4}
ADC wav LLL ngtdm Busyness	CD150um glszm LALGLE	4.45×10^{-4}	0.64	1.70×10^{4}
ADC wav HHH glrlm SRLGLE	CD100um glrlm RunVariance	4.45×10^{-4}	0.64	1.69×10^{4}
ADC wav HHH firstorder Mean	MEAN ODSum Haralick ASM F0	4.45×10^{-4}	-0.64	1.69×10^{4}
ADC wav HLH firstorder RootMeanSquared	MEAN ODSum Haralick IMOC2 F12	4.58×10^{-4}	-0.64	1.64×10^{4}
ADC wav HHH firstorder Maximum	CD200um ngtdm Strength	4.60×10^{-4}	0.64	1.62×10^{4}
ADC wav HLL gldm LDHGLE	CD200um wav HL glcm ClusterProminence	4.62×10^{-4}	0.639	1.61×10^{4}
ADC wav HHH glrlm SRLGLE	CD150um glszm LALGLE	4.69×10^{-4}	0.639	1.58×10^{4}
ADC wav HLL glszm ZoneVariance	CD150um glszm LALGLE	4.81×10^{-4}	0.638	1.54×10^{4}
ADC wav LHL gldm DependenceVariance	CD100um lbp 2D glszm ZoneVariance	4.88×10^{-4}	0.638	1.51×10^{4}

**Radiomic feature name**	**Pathomic feature name**	**ρ**	**Q-value**	**BF**
ADC wav LLH firstorder 90Percentile	MEAN ODSum Haralick ASM F0	4.91×10^{-4}	0.638	1.50×10^{4}
ADC wav HHH glrlm LRHGLE	MEAN ODSum Haralick IMOC2 F12	5.27×10^{-4}	-0.636	1.39×10^{4}
ADC wav HHH firstorder Mean	MEAN ODSum Haralick IMOC2 F12	5.44×10^{-4}	0.636	1.35×10^{4}
ADC wav HLL glcm ClusterProminence	CD200um wav HH firstorder InterquartileRange	5.51×10^{-4}	0.635	1.33×10^{4}
ADC wav HLL firstorder Uniformity	CD150um lbp 2D glrlm RunVariance	5.55×10^{-4}	0.635	1.31×10^{4}
ADC wav LLH firstorder Median	MEAN ODSum Haralick IMOC2 F12	5.58×10^{-4}	-0.635	1.30×10^{4}
ADC firstorder 10Percentile	CD100um ngtdm Strength	5.72×10^{-4}	0.634	1.27×10^{4}
ADC wav LLL gldm LDHGLE	CD150um wav HH glcm ClusterProminence	5.93×10^{-4}	0.634	1.22×10^{4}
ADC wav LLH gldm DependenceVariance	CD100um lbp 2D glszm ZoneVariance	6.00×10^{-4}	0.633	1.20×10^{4}
ADC wav LHH firstorder Maximum	MEAN ODSum Haralick ASM F0	6.00×10^{-4}	0.633	1.20×10^{4}
ADC wav HHL firstorder Mean	CD100um ngtdm Strength	6.13×10^{-4}	-0.633	1.17×10^{4}
ADC wav LHL gldm DependenceVariance	CD100um glrlm RunVariance	6.85×10^{-4}	0.63	1.05×10^{4}
ADC wav HLL gldm LDLGLE	CD100um glrlm RunVariance	6.97×10^{-4}	0.63	1.03×10^{4}
ADC glcm Imc2	CD150um glszm LALGLE	7.06×10^{-4}	-0.63	1.01×10^{4}
ADC wav HHH glcm MaximumProbability	CD100um glrlm RunVariance	7.06×10^{-4}	0.63	1.01×10^{4}
ADC wav LLH gldm DependenceVariance	CD150um lbp 2D glrlm RunVariance	7.10×10^{-4}	0.629	9.98×10^{3}
ADC wav LLH gldm DependenceVariance	CD100um glrlm RunVariance	7.11×10^{-4}	0.629	9.93×10^{3}
ADC glszm GLNUN	CD100um lbp 2D glszm ZoneVariance	7.11×10^{-4}	0.629	9.90×10^{3}
ADC wav HLL gldm LDLGLE	CD100um lbp 2D glszm ZoneVariance	7.21×10^{-4}	0.629	9.73×10^{3}
ADC wav HHH firstorder Median	MEAN ODSum Haralick IMOC2 F12	7.24×10^{-4}	-0.629	9.66×10^{3}
ADC firstorder Minimum	CD100um ngtdm Strength	7.34×10^{-4}	0.628	9.50×10^{3}
ADC wav HHH glcm MaximumProbability	CD100um lbp 2D glszm ZoneVariance	7.45×10^{-4}	0.628	9.33×10^{3}
ADC firstorder 10Percentile	MEAN ODSum Haralick IMOC2 F12	7.45×10^{-4}	-0.628	9.29×10^{3}
ADC wav HHH glrlm SRLGLE	CD150um lbp 2D glrlm RunVariance	7.45×10^{-4}	0.628	9.27×10^{3}
ADC glszm GLNUN	CD100um glrlm RunVariance	7.58×10^{-4}	0.627	9.10×10^{3}
ADC wav LLH glcm ClusterProminence	CD100um ngtdm Strength	7.79×10^{-4}	0.627	8.83×10^{3}
ADC wav LLH firstorder 10Percentile	MEAN ODSum Haralick IMOC2 F12	8.17×10^{-4}	0.626	8.41×10^{3}
ADC wav HLL glcm ClusterProminence	CD200um wav HH ngtdm Complexity	8.34×10^{-4}	0.625	8.22×10^{3}
ADC wav LHH firstorder Median	CD100um ngtdm Strength	8.55×10^{-4}	-0.625	8.00×10^{3}
ADC wav LLL firstorder Minimum	CD100um ngtdm Strength	8.82×10^{-4}	0.624	7.74×10^{3}
ADC wav LLH glrlm LRHGLE	MEAN ODSum Haralick IMOC2 F12	9.15×10^{-4}	-0.623	7.43×10^{3}

**Radiomic feature name**	**Pathomic feature name**	**ρ**	**Q-value**	**BF**
ADC wav HLH ngtdm Complexity	CD200um wav HL glcm ClusterProminence	9.15×10^{-4}	0.623	7.42×10^{3}
ADC wav HLL glcm ClusterShade	CD200um wav HH ngtdm Complexity	9.17×10^{-4}	-0.623	7.38×10^{3}
ADC wav HHL glcm ClusterProminence	CD100um ngtdm Strength	9.40×10^{-4}	0.622	7.19×10^{3}
ADC wav LLH firstorder Median	CD100um ngtdm Strength	9.41×10^{-4}	0.622	7.16×10^{3}
ADC wav LHL glcm ClusterShade	CD100um ngtdm Strength	9.46×10^{-4}	-0.622	7.10×10^{3}
ADC glszm GLNUN	CD150um glszm LALGLE	9.59×10^{-4}	0.622	6.98×10^{3}
ADC wav LHL firstorder Mean	CD100um ngtdm Strength	9.87×10^{-4}	0.621	6.74×10^{3}
ADC wav LLH ngtdm Contrast	CD100um ngtdm Strength	9.87×10^{-4}	0.621	6.69×10^{3}
ADC wav LHH ngtdm Contrast	CD100um ngtdm Strength	9.87×10^{-4}	0.621	6.68×10^{3}
ADC wav LHL ngtdm Contrast	CD100um ngtdm Strength	9.87×10^{-4}	0.621	6.68×10^{3}
ADC wav HHH ngtdm Contrast	CD100um ngtdm Strength	9.87×10^{-4}	0.621	6.67×10^{3}
ADC wav HLH ngtdm Contrast	CD100um ngtdm Strength	9.87×10^{-4}	0.621	6.66×10^{3}
ADC wav LLL ngtdm Contrast	CD100um ngtdm Strength	9.87×10^{-4}	0.621	6.64×10^{3}
ADC wav LHH glcm ClusterShade	CD100um ngtdm Strength	1.00×10^{-3}	0.62	6.54×10^{3}
ADC wav HLL ngtdm Contrast	CD100um ngtdm Strength	1.01×10^{-3}	0.62	6.48×10^{3}
ADC wav HLL ngtdm Complexity	CD200um wav HH firstorder InterquartileRange	1.02×10^{-3}	0.62	6.38×10^{3}
ADC wav HHH glcm ClusterShade	CD100um ngtdm Strength	1.02×10^{-3}	0.62	6.37×10^{3}
ADC wav LHL glcm DifferenceVariance	CD100um ngtdm Strength	1.03×10^{-3}	0.619	6.25×10^{3}
ADC wav LLH glcm ClusterShade	CD100um ngtdm Strength	1.04×10^{-3}	-0.619	6.20×10^{3}
ADC wav LLL glcm InverseVariance	CD100um lbp 2D glszm ZoneVariance	1.05×10^{-3}	0.619	6.14×10^{3}
ADC wav LHL firstorder Median	CD100um ngtdm Strength	1.07×10^{-3}	0.618	6.01×10^{3}
ADC wav LHL gldm DependenceVariance	CD150um lbp 2D glrlm RunVariance	1.11×10^{-3}	0.618	5.79×10^{3}
ADC glszm GLNUN	CD150um lbp 2D glrlm RunVariance	1.15×10^{-3}	0.617	5.58×10^{3}
ADC wav HHH firstorder RootMeanSquared	CD100um ngtdm Strength	1.15×10^{-3}	0.617	5.54×10^{3}
ADC wav LHH firstorder RootMeanSquared	CD100um ngtdm Strength	1.17×10^{-3}	0.616	5.43×10^{3}
ADC wav LHL firstorder Maximum	MEAN ODSum Haralick ASM F0	1.19×10^{-3}	0.616	5.33×10^{3}
ADC wav HLH glszm GrayLevelVariance	CD100um ngtdm Strength	1.19×10^{-3}	0.616	5.32×10^{3}
ADC wav HHL glcm InverseVariance	CD100um lbp 2D glszm ZoneVariance	1.19×10^{-3}	0.616	5.29×10^{3}
ADC wav HLL firstorder Mean	CD100um ngtdm Strength	1.19×10^{-3}	0.616	5.28×10^{3}

**Radiomic feature name**	**Pathomic feature name**	**ρ**	**Q-value**	**BF**
ADC wav HLL gldm LDLGLE	CD150um glszm LALGLE	1.19×10^{-3}	0.615	5.26×10^{3}
ADC wav HHL glcm InverseVariance	CD100um glrlm RunVariance	1.23×10^{-3}	0.615	5.12×10^{3}
ADC wav HHH firstorder Median	CD100um ngtdm Strength	1.26×10^{-3}	0.614	4.95×10^{3}
ADC lbp 3D m2 glrlm LRHGLE	CD200um wav LL firstorder Kurtosis	1.26×10^{-3}	-0.614	4.94×10^{3}
ADC wav HLL glcm InverseVariance	CD100um lbp 2D glszm ZoneVariance	1.28×10^{-3}	0.614	4.86×10^{3}
ADC glcm InverseVariance	CD100um lbp 2D glszm ZoneVariance	1.28×10^{-3}	0.614	4.83×10^{3}
ADC firstorder Median	CD100um ngtdm Strength	1.28×10^{-3}	0.613	4.83×10^{3}
ADC wav HLL glcm InverseVariance	CD100um glrlm RunVariance	1.32×10^{-3}	0.613	4.68×10^{3}
ADC wav LHH ngtdm Strength	CD100um ngtdm Strength	1.35×10^{-3}	0.612	4.58×10^{3}
ADC wav LLL glcm InverseVariance	CD100um glrlm RunVariance	1.43×10^{-3}	0.611	4.31×10^{3}
ADC wav LHL glszm SALGLE	MEAN ODSum Haralick IMOC2 F12	1.43×10^{-3}	-0.611	4.29×10^{3}
ADC gldm DependenceVariance	CD150um glszm LALGLE	1.49×10^{-3}	0.61	4.11×10^{3}
ADC wav HHH glcm MaximumProbability	CD150um lbp 2D glrlm RunVariance	1.52×10^{-3}	0.609	4.03×10^{3}
ADC wav LHL glcm Idm	CD150um glszm LALGLE	1.54×10^{-3}	0.609	3.98×10^{3}
ADC glcm InverseVariance	CD100um glrlm RunVariance	1.55×10^{-3}	0.609	3.94×10^{3}
ADC wav HHH ngtdm Strength	CD100um ngtdm Strength	1.57×10^{-3}	0.608	3.87×10^{3}
ADC wav LLL glszm ZoneVariance	CD150um glszm LALGLE	1.60×10^{-3}	0.608	3.80×10^{3}
ADC firstorder TotalEnergy	CD200um wav HH firstorder InterquartileRange	1.61×10^{-3}	0.608	3.76×10^{3}
ADC lbp 3D m2 gldm SDLGLE	MEAN ODSum Haralick IMOC2 F12	1.69×10^{-3}	-0.607	3.59×10^{3}
ADC wav LHL glcm Idm	CD150um lbp 2D glrlm RunVariance	1.73×10^{-3}	0.606	3.50×10^{3}
ADC wav LLH glszm LAHGLE	MEAN ODSum Haralick IMOC2 F12	1.74×10^{-3}	-0.606	3.47×10^{3}
ADC firstorder Median	MEAN ODSum Haralick IMOC2 F12	1.77×10^{-3}	-0.606	3.41×10^{3}
ADC wav LLH gldm SDLGLE	CD200um ngtdm Strength	1.80×10^{-3}	0.605	3.33×10^{3}
ADC wav LLH firstorder RootMeanSquared	MEAN ODSum Haralick IMOC2 F12	1.81×10^{-3}	-0.605	3.31×10^{3}
ADC wav LLH glrlm LRHGLE	CD100um ngtdm Strength	1.85×10^{-3}	0.604	3.23×10^{3}
ADC lbp 3D m2 firstorder Range	CD200um wav LL firstorder Kurtosis	1.86×10^{-3}	-0.604	3.22×10^{3}
ADC wav LLH ngtdm Strength	CD100um ngtdm Strength	1.90×10^{-3}	0.604	3.14×10^{3}
ADC wav HLL ngtdm Complexity	CD200um wav HL ngtdm Complexity	1.93×10^{-3}	0.603	3.09×10^{3}
ADC wav HHH glcm MaximumProbability	CD150um glszm LALGLE	1.93×10^{-3}	0.603	3.08×10^{3}
ADC wav LHL gldm LDHGLE	CD100um ngtdm Strength	2.00×10^{-3}	0.602	2.97×10^{3}
ADC wav LLL glcm InverseVariance	CD150um lbp 2D glrlm RunVariance	2.00×10^{-3}	0.602	2.96×10^{3}

Radiomic feature name	Pathomic feature name	ρ	Q-value	BF
ADC wav HLH glcm ClusterShade	CD100um ngtdm Strength	2.02×10^{-3}	-0.602	2.93×10^{3}
ADC wav HHH glrlm LRHGLE	CD100um ngtdm Strength	2.11×10^{-3}	0.601	2.79×10^{3}
ADC wav HLH firstorder RootMeanSquared	CD100um ngtdm Strength	2.16×10^{-3}	0.6	2.72×10^{3}
ADC wav HLL firstorder Maximum	CD150um wav HH glcm ClusterProminence	2.20×10^{-3}	0.6	2.68×10^{3}
ADC lbp 2D glszm ZoneVariance	MEAN Cytoplasm EosinODStdDev	2.32×10^{-3}	0.599	2.53×10^{3}
ADC wav LLL glcm InverseVariance	CD150um glszm LALGLE	2.45×10^{-3}	0.597	2.40×10^{3}
ADC wav LHL gldm DependenceVariance	CD150um glszm LALGLE	2.46×10^{-3}	0.597	2.38×10^{3}
ADC wav HLH ngtdm Strength	CD100um ngtdm Strength	2.58×10^{-3}	0.596	2.27×10^{3}
ADC wav HLL glcm InverseVariance	CD150um glszm LALGLE	2.62×10^{-3}	0.596	2.23×10^{3}
ADC wav HHH firstorder Maximum	MEAN ODSum Haralick ASM F0	2.66×10^{-3}	0.595	2.19×10^{3}
ADC lbp 3D m2 gldm LDLGLE	CD200um wav LL firstorder Kurtosis	2.91×10^{-3}	0.593	2.01×10^{3}
ADC lbp 3D k firstorder Minimum	MEAN ODSum Haralick IMOC2 F12	2.92×10^{-3}	-0.593	2.00×10^{3}
ADC lbp 3D m1 gldm SDLGLE	MEAN ODSum Haralick IMOC2 F12	3.09×10^{-3}	-0.592	1.89×10^{3}
ADC wav LLH firstorder RootMeanSquared	CD100um ngtdm Strength	3.14×10^{-3}	0.591	1.86×10^{3}
ADC wav HLL glcm ClusterShade	CD200um wav HL ngtdm Complexity	3.14×10^{-3}	-0.591	1.85×10^{3}
ADC wav LLL firstorder Maximum	MEAN ODSum Haralick ASM F0	3.35×10^{-3}	0.59	1.74×10^{3}
ADC glcm ClusterShade	MEAN ODSum Haralick ASM F0	3.36×10^{-3}	0.589	1.73×10^{3}
ADC wav LLH gldm LDHGLE	MEAN ODSum Haralick IMOC2 F12	3.37×10^{-3}	-0.589	1.72×10^{3}
ADC wav HLL firstorder TotalEnergy	CD200um wav HH ngtdm Complexity	3.37×10^{-3}	0.589	1.71×10^{3}
ADC wav LHL firstorder RootMeanSquared	CD100um ngtdm Strength	3.49×10^{-3}	0.588	1.66×10^{3}
ADC wav LHH firstorder Maximum	MEAN ODSum Haralick IMOC2 F12	3.54×10^{-3}	-0.588	1.63×10^{3}
ADC glcm InverseVariance	CD150um lbp 2D glrlm RunVariance	3.56×10^{-3}	0.588	1.62×10^{3}
ADC wav LHL firstorder Maximum	CD200um ngtdm Strength	3.57×10^{-3}	0.588	1.61×10^{3}
ADC glcm DifferenceAverage	MEAN ODSum Haralick ASM F0	3.95×10^{-3}	0.585	1.46×10^{3}
ADC wav HHL glcm InverseVariance	CD150um lbp 2D glrlm RunVariance	4.11×10^{-3}	0.584	1.40×10^{3}
ADC lbp 3D k ngtdm Strength	MEAN ODSum Haralick ASM F0	4.13×10^{-3}	0.584	1.39×10^{3}
ADC wav HLL glcm ClusterShade	CD200um wav HH firstorder InterquartileRange	4.17×10^{-3}	-0.584	1.37×10^{3}
ADC lbp 3D k ngtdm Strength	CD200um ngtdm Strength	4.25×10^{-3}	0.583	1.34×10^{3}
ADC wav HHL glcm ClusterShade	CD200um ngtdm Strength	4.27×10^{-3}	0.583	1.34×10^{3}

Radiomic feature name	Pathomic feature name	ρ	Q-value	BF
ADC glcm InverseVariance	CD150um glszm LALGLE	4.36×10^{-3}	0.582	1.31×10^{3}
ADC wav HHL glcm ClusterShade	MEAN ODSum Haralick IMOC2 F12	4.63×10^{-3}	-0.581	1.23×10^{3}
ADC wav LLH gldm LDHGLE	CD100um ngtdm Strength	4.84×10^{-3}	0.58	1.18×10^{3}
ADC wav LLH gldm DependenceVariance	CD150um glszm LALGLE	4.99×10^{-3}	0.579	1.14×10^{3}
ADC lbp 3D k ngtdm Strength	MEAN ODSum Haralick IMOC2 F12	5.01×10^{-3}	-0.579	1.13×10^{3}
ADC wav HLH glrlm SRLGLE	CD150um glszm LALGLE	5.01×10^{-3}	0.579	1.13×10^{3}
ADC wav LLH firstorder 10Percentile	CD100um ngtdm Strength	5.02×10^{-3}	-0.579	1.13×10^{3}
ADC wav LLH firstorder Mean	MEAN ODSum Haralick ASM F0	5.12×10^{-3}	0.578	1.10×10^{3}
ADC wav HHL glcm InverseVariance	CD150um glszm LALGLE	5.24×10^{-3}	0.578	1.08×10^{3}
ADC lbp 3D k firstorder Minimum	MEAN ODSum Haralick ASM F0	5.32×10^{-3}	0.577	1.06×10^{3}
ADC wav HLL gldm LDHGLE	CD200um wav HH ngtdm Complexity	5.36×10^{-3}	0.577	1.05×10^{3}
ADC wav LHL ngtdm Strength	CD100um ngtdm Strength	5.45×10^{-3}	0.576	1.03×10^{3}
ADC wav LLL ngtdm Strength	CD50um ngtdm Complexity	5.77×10^{-3}	0.575	9.73×10^{2}
ADC wav HLL firstorder Median	CD100um ngtdm Strength	5.77×10^{-3}	0.575	9.71×10^{2}
ADC wav LLL firstorder Maximum	CD200um ngtdm Strength	5.94×10^{-3}	0.574	9.44×10^{2}
ADC wav HLL glcm InverseVariance	CD150um lbp 2D glrlm RunVariance	6.43×10^{-3}	0.572	8.74×10^{2}
ADC wav HHL ngtdm Strength	CD100um ngtdm Strength	6.50×10^{-3}	0.572	8.63×10^{2}
ADC wav HLH firstorder Median	CD200um ngtdm Strength	6.51×10^{-3}	0.572	8.58×10^{2}
ADC wav LLH firstorder Mean	CD100um ngtdm Strength	6.62×10^{-3}	0.571	8.43×10^{2}
ADC wav HLL glcm ClusterProminence	CD50um wav HL glcm ClusterShade	6.74×10^{-3}	0.571	8.27×10^{2}
ADC wav LLH gldm SDLGLE	MEAN ODSum Haralick ASM F0	7.00×10^{-3}	0.57	7.96×10^{2}
ADC wav LLH glszm LAHGLE	CD100um ngtdm Strength	7.18×10^{-3}	0.569	7.75×10^{2}
ADC wav HHL glcm Idmn	MEAN ODSum Haralick IMOC2 F12	7.24×10^{-3}	0.569	7.68×10^{2}
ADC wav HLH firstorder Median	MEAN ODSum Haralick IMOC2 F12	7.24×10^{-3}	-0.569	7.66×10^{2}
ADC wav HLL glcm ClusterProminence	CD200um wav HL ngtdm Complexity	7.65×10^{-3}	0.567	7.26×10^{2}
ADC wav LLH firstorder 90Percentile	MEAN ODSum Haralick IMOC2 F12	7.67×10^{-3}	-0.567	7.22×10^{2}
ADC wav LLH firstorder 90Percentile	CD100um ngtdm Strength	7.73×10^{-3}	0.567	7.15×10^{2}
ADC wav HHL glszm LAHGLE	MEAN ODSum Haralick ASM F0	7.84×10^{-3}	0.566	7.04×10^{2}
ADC wav HLH gldm SDLGLE	MEAN ODSum Haralick ASM F0	7.85×10^{-3}	0.566	7.01×10^{2}
ADC lbp 2D glszm ZoneVariance	MEAN Cell EosinODMax	8.58×10^{-3}	0.564	6.43×10^{2}
ADC wav LHH glcm Idm	CD100um lbp 2D glszm ZoneVariance	8.92×10^{-3}	0.563	6.19×10^{2}

**Radiomic feature name**	**Pathomic feature name**	**ρ**	**Q-value**	**BF**
ADC wav LHH firstorder Maximum	CD100um ngtdm Strength	9.06×10^{-3}	0.563	6.09×10^{2}
ADC lbp 3D k firstorder Minimum	CD150um ngtdm Strength	9.27×10^{-3}	0.562	5.94×10^{2}
ADC wav HLL firstorder TotalEnergy	CD200um wav HH firstorder InterquartileRange	9.47×10^{-3}	0.561	5.81×10^{2}
ADC wav LHH glcm Idm	CD100um glrlm RunVariance	9.53×10^{-3}	0.561	5.76×10^{2}
ADC lbp 3D m1 glcm JointEnergy	CD200um ngtdm Strength	9.71×10^{-3}	0.56	5.65×10^{2}
ADC wav LHH ngtdm Coarseness	CD100um lbp 2D glcm JointEnergy	1.00×10^{-2}	0.56	5.48×10^{2}
ADC lbp 3D m2 glszm LGLZE	MEAN ODSum Haralick IMOC2 F12	1.03×10^{-2}	-0.559	5.30×10^{2}
ADC firstorder TotalEnergy	CD200um wav HH ngtdm Complexity	1.05×10^{-2}	0.558	5.23×10^{2}
ADC firstorder 10Percentile	CD150um ngtdm Strength	1.06×10^{-2}	0.558	5.15×10^{2}
ADC wav LLH glcm InverseVariance	CD100um lbp 2D glszm ZoneVariance	1.08×10^{-2}	0.558	5.06×10^{2}
ADC lbp 3D k glcm Correlation	MEAN ODSum Haralick ASM F0	1.09×10^{-2}	-0.557	5.00×10^{2}
ADC wav LHH firstorder Minimum	CD200um ngtdm Strength	1.14×10^{-2}	-0.556	4.77×10^{2}
ADC lbp 2D glcm JointEnergy	CD200um wav LL firstorder Kurtosis	1.14×10^{-2}	0.556	4.77×10^{2}
ADC wav LHL glszm SALGLE	CD200um ngtdm Strength	1.17×10^{-2}	0.555	4.63×10^{2}
ADC lbp 2D gldm LDLGLE	CD100um lbp 2D glcm JointEnergy	1.25×10^{-2}	0.553	4.35×10^{2}
ADC wav HLH gldm SDLGLE	CD200um ngtdm Strength	1.28×10^{-2}	0.553	4.24×10^{2}
ADC wav HLH gldm SDLGLE	CD200um lbp 2D glcm Idm	1.30×10^{-2}	0.552	4.19×10^{2}
ADC lbp 2D glszm ZoneVariance	MEAN Cell EosinODStdDev	1.30×10^{-2}	0.552	4.16×10^{2}
ADC lbp 3D m2 glrlm SRLGLE	MEAN ODSum Haralick IMOC2 F12	1.30×10^{-2}	-0.552	4.16×10^{2}
ADC wav LLH firstorder Maximum	MEAN ODSum Haralick ASM F0	1.33×10^{-2}	0.551	4.05×10^{2}
ADC wav HHH firstorder Mean	CD200um ngtdm Strength	1.33×10^{-2}	-0.551	4.05×10^{2}
ADC wav LHH gldm LDHGLE	CD200um ngtdm Strength	1.34×10^{-2}	0.551	4.01×10^{2}
ADC lbp 3D m2 gldm SDLGLE	CD200um ngtdm Strength	1.39×10^{-2}	0.55	3.87×10^{2}
ADC wav HHH firstorder Maximum	CD100um ngtdm Strength	1.40×10^{-2}	0.55	3.83×10^{2}
ADC lbp 3D m2 gldm SDHGLE	CD200um wav LL firstorder Kurtosis	1.48×10^{-2}	-0.548	3.64×10^{2}
ADC wav HLL firstorder Maximum	CD200um wav HL glcm ClusterProminence	1.53×10^{-2}	0.547	3.52×10^{2}
ADC wav HHL firstorder TotalEnergy	CD150um wav HH glcm ClusterProminence	1.53×10^{-2}	0.547	3.50×10^{2}
ADC wav LLL gldm LDHGLE	CD200um wav HL glcm ClusterProminence	1.53×10^{-2}	0.547	3.49×10^{2}
ADC lbp 3D m1 gldm SDLGLE	MEAN ODSum Haralick ASM F0	1.55×10^{-2}	0.547	3.46×10^{2}

**Radiomic feature name**	**Pathomic feature name**	**ρ**	**Q-value**	**BF**
ADC wav LHH ngtdm Coarseness	CD50um wav LL glcm Imc2	1.55×10^{-2}	-0.547	3.43×10^{2}
ADC wav HLH gldm SDLGLE	CD100um ngtdm Strength	1.60×10^{-2}	0.546	3.34×10^{2}
ADC wav HLL gldm LDHGLE	CD200um wav HH firstorder InterquartileRange	1.61×10^{-2}	0.546	3.30×10^{2}
ADC wav LLH glcm InverseVariance	CD150um glszm LALGLE	1.70×10^{-2}	0.544	3.14×10^{2}
ADC wav LLH glcm InverseVariance	CD100um glrlm RunVariance	1.75×10^{-2}	0.543	3.05×10^{2}
ADC wav HLH ngtdm Complexity	CD50um wav HL glcm ClusterShade	1.76×10^{-2}	0.543	3.03×10^{2}
ADC wav LHH ngtdm Coarseness	CD100um lbp 2D glcm SumEntropy	1.76×10^{-2}	-0.543	3.01×10^{2}
ADC firstorder Median	CD150um ngtdm Strength	1.77×10^{-2}	0.543	3.00×10^{2}
ADC lbp 3D m2 gldm SDLGLE	MEAN ODSum Haralick ASM F0	1.77×10^{-2}	0.543	2.99×10^{2}
ADC wav LHH firstorder Minimum	MEAN ODSum Haralick ASM F0	1.80×10^{-2}	-0.542	2.94×10^{2}
ADC firstorder Minimum	CD150um ngtdm Strength	1.81×10^{-2}	0.542	2.92×10^{2}
ADC wav LHH firstorder Mean	CD150um ngtdm Strength	1.81×10^{-2}	-0.542	2.91×10^{2}
ADC wav HLL glcm ClusterProminence	CD150um wav HH ngtdm Strength	1.84×10^{-2}	0.541	2.86×10^{2}
ADC wav LHH glcm Idm	CD150um glszm LALGLE	1.88×10^{-2}	0.541	2.80×10^{2}
ADC wav HHH firstorder Maximum	MEAN ODSum Haralick IMOC2 F12	1.93×10^{-2}	-0.54	2.72×10^{2}
ADC wav LHL firstorder Maximum	MEAN ODSum Haralick IMOC2 F12	1.97×10^{-2}	-0.539	2.66×10^{2}
ADC wav HLL firstorder TotalEnergy	CD200um wav HL ngtdm Complexity	1.98×10^{-2}	0.539	2.64×10^{2}
ADC lbp 3D m2 glszm SAHGLE	CD200um firstorder Kurtosis	1.98×10^{-2}	-0.539	2.63×10^{2}
ADC wav HLL glcm Idmn	MEAN ODSum Haralick IMOC2 F12	1.99×10^{-2}	0.539	2.62×10^{2}
ADC wav HHL glszm LAHGLE	CD200um ngtdm Strength	1.99×10^{-2}	0.539	2.61×10^{2}
ADC wav HLH ngtdm Complexity	CD200um wav HH firstorder InterquartileRange	1.99×10^{-2}	0.539	2.61×10^{2}
ADC wav HHL firstorder Mean	CD150um ngtdm Strength	2.01×10^{-2}	-0.538	2.58×10^{2}
ADC wav LLL firstorder Minimum	CD150um ngtdm Strength	2.01×10^{-2}	0.538	2.57×10^{2}
ADC lbp 2D gldm LDLGLE	CD100um lbp 2D glcm JointEntropy	2.01×10^{-2}	-0.538	2.56×10^{2}
ADC wav HLH gldm SDLGLE	MEAN ODSum HaralickInverseDifferenceMomen	2.01×10^{-2}	0.538	2.56×10^{2}
ADC wav HHL firstorder Maximum	MEAN ODSum Haralick ASM F0	2.01×10^{-2}	0.538	2.56×10^{2}
ADC lbp 3D m2 glrlm SRLGLE	CD200um ngtdm Strength	2.01×10^{-2}	0.538	2.55×10^{2}
ADC wav HLH glrlm SRLGLE	CD150um lbp 2D glrlm RunVariance	2.08×10^{-2}	0.537	2.47×10^{2}
ADC lbp 3D m2 glszm LGLZE	CD200um ngtdm Strength	2.11×10^{-2}	0.537	2.43×10^{2}
ADC wav LLH gldm SDLGLE	CD100um ngtdm Strength	2.14×10^{-2}	0.536	2.40×10^{2}
ADC wav HLH gldm SDLGLE	CD200um wav LH glcm ClusterShade	2.16×10^{-2}	-0.536	2.37×10^{2}

**Radiomic feature name**	**Pathomic feature name**	**ρ**	**Q-value**	**BF**
ADC wav LHH gldm DependenceVariance	CD100um lbp 2D glszm ZoneVariance	2.18×10^{-2}	0.536	2.35×10^{2}
ADC wav HLL ngtdm Complexity	CD50um wav HL glcm ClusterShade	2.22×10^{-2}	0.535	2.30×10^{2}
ADC wav HLL firstorder Maximum	CD50um wav HL glcm ClusterShade	2.24×10^{-2}	0.535	2.28×10^{2}
ADC wav HLH ngtdm Complexity	CD200um wav LH firstorder InterquartileRange	2.26×10^{-2}	0.534	2.25×10^{2}
ADC glcm ClusterTendency	CD150um wav HH glcm ClusterProminence	2.27×10^{-2}	0.534	2.23×10^{2}
ADC wav LHH gldm DependenceVariance	CD100um glrlm RunVariance	2.28×10^{-2}	0.534	2.22×10^{2}
ADC wav HLL gldm LDHGLE	CD200um wav HL ngtdm Complexity	2.35×10^{-2}	0.533	2.16×10^{2}
ADC lbp 3D m2 firstorder InterquartileRange	CD150um lbp 2D glcm InverseVariance	2.40×10^{-2}	0.533	2.11×10^{2}
ADC wav HLL glcm ClusterProminence	CD100um glcm DifferenceVariance	2.43×10^{-2}	0.532	2.08×10^{2}
ADC wav HLH glcm Idm	CD100um glrlm RunVariance	2.44×10^{-2}	0.532	2.07×10^{2}
ADC wav HLH glrlm SRLGLE	CD100um glrlm RunVariance	2.50×10^{-2}	0.531	2.02×10^{2}
ADC glcm ClusterTendency	CD50um ngtdm Complexity	2.50×10^{-2}	0.531	2.02×10^{2}
ADC wav LLH glrlm LRHGLE	CD150um ngtdm Strength	2.50×10^{-2}	0.531	2.02×10^{2}
ADC wav HLL gldm LDHGLE	CD50um ngtdm Complexity	2.52×10^{-2}	0.531	2.00×10^{2}
ADC wav LLH firstorder Median	CD150um ngtdm Strength	2.54×10^{-2}	0.531	1.98×10^{2}
ADC wav HHL glszm SALGLE	CD150um lbp 2D glrlm RunVariance	2.56×10^{-2}	0.53	1.95×10^{2}
ADC firstorder Range	CD50um ngtdm Complexity	2.56×10^{-2}	0.53	1.95×10^{2}
ADC lbp 2D glszm ZoneVariance	MEAN ODSum Max	2.59×10^{-2}	0.53	1.93×10^{2}
ADC wav HLH glrlm SRLGLE	CD100um lbp 2D glszm ZoneVariance	2.59×10^{-2}	0.53	1.92×10^{2}
ADC firstorder TotalEnergy	CD50um glcm DifferenceAverage	2.59×10^{-2}	0.53	1.92×10^{2}
ADC wav HLH ngtdm Complexity	CD150um glcm DifferenceVariance	2.60×10^{-2}	0.53	1.91×10^{2}
ADC wav HLH firstorder Mean	CD150um wav HH glcm ClusterProminence	2.61×10^{-2}	-0.529	1.90×10^{2}
ADC wav HLH glcm Idm	CD100um lbp 2D glszm ZoneVariance	2.63×10^{-2}	0.529	1.88×10^{2}
ADC wav LLL gldm LDHGLE	CD200um wav HH firstorder InterquartileRange	2.66×10^{-2}	0.529	1.86×10^{2}
ADC wav HLL glcm ClusterProminence	CD200um wav HH firstorder 10Percentile	2.66×10^{-2}	-0.529	1.86×10^{2}
ADC wav HLL glcm ClusterProminence	CD200um wav LH firstorder InterquartileRange	2.66×10^{-2}	0.529	1.86×10^{2}
ADC wav HLH ngtdm Complexity	MEAN ODSum Haralick ASM F0	2.67×10^{-2}	0.528	1.84×10^{2}
ADC wav LLH firstorder Mean	CD150um ngtdm Strength	2.67×10^{-2}	0.528	1.83×10^{2}
ADC wav HLL glcm ClusterProminence	CD150um glcm DifferenceVariance	2.67×10^{-2}	0.528	1.83×10^{2}

**Radiomic feature name**	**Pathomic feature name**	**ρ**	**Q-value**	**BF**
ADC lbp 3D m2 glrlm SRHGLE	CD200um firstorder Kurtosis	2.68×10^{-2}	-0.528	1.82×10^{2}
ADC lbp 3D m1 glcm JointAverage	CD200um wav LL firstorder Kurtosis	2.68×10^{-2}	-0.528	1.82×10^{2}
ADC wav LHL glszm SALGLE	MEAN ODSum Haralick ASM F0	2.68×10^{-2}	0.528	1.82×10^{2}
ADC wav LHH ngtdm Coarseness	CD150um lbp 2D glcm ClusterProminence	2.68×10^{-2}	-0.528	1.82×10^{2}
ADC wav LLL ngtdm Strength	CD200um wav HH glcm JointAverage	2.69×10^{-2}	0.528	1.81×10^{2}
ADC lbp 3D m1 glszm GLNUN	CD200um wav LL firstorder Kurtosis	2.70×10^{-2}	0.528	1.79×10^{2}
ADC wav HLH gldm SDLGLE	CD200um lbp 2D ngtdm Complexity	2.77×10^{-2}	-0.527	1.75×10^{2}
ADC lbp 3D m1 glrlm HighGrayLevelRunEmphasis	CD200um wav LL firstorder Kurtosis	2.77×10^{-2}	-0.527	1.75×10^{2}
ADC wav HLL firstorder 10Percentile	CD150um wav HH glcm ClusterProminence	2.78×10^{-2}	-0.527	1.74×10^{2}
ADC wav HLH gldm LDHGLE	CD200um lbp 2D glrlm ShortRunEmphasis	2.80×10^{-2}	0.526	1.72×10^{2}
ADC wav HHH gldm LDHGLE	CD200um ngtdm Strength	2.90×10^{-2}	0.525	1.67×10^{2}
ADC wav HLH ngtdm Complexity	CD150um wav HH ngtdm Strength	2.92×10^{-2}	0.525	1.65×10^{2}
ADC wav LHH gldm DependenceVariance	CD150um lbp 2D glrlm RunVariance	2.93×10^{-2}	0.525	1.64×10^{2}
ADC glcm DifferenceAverage	CD200um ngtdm Strength	2.98×10^{-2}	0.524	1.61×10^{2}
ADC wav HLH firstorder Mean	CD200um wav HL glcm ClusterProminence	3.03×10^{-2}	-0.524	1.58×10^{2}
ADC wav HLL ngtdm Complexity	CD200um wav HH firstorder Maximum	3.06×10^{-2}	0.524	1.57×10^{2}
ADC wav HLH gldm SDLGLE	CD150um lbp 2D glcm DifferenceEntropy	3.06×10^{-2}	-0.523	1.56×10^{2}
ADC wav LHL glcm DifferenceVariance	MEAN ODSum HaralickDifferenceVariance F9	3.08×10^{-2}	0.523	1.55×10^{2}
ADC wav HLL glcm ClusterProminence	CD50um glcm DifferenceAverage	3.10×10^{-2}	0.523	1.54×10^{2}
ADC lbp 3D m2 firstorder Range	CD200um ngtdm Strength	3.18×10^{-2}	-0.522	1.50×10^{2}
ADC lbp 2D glcm SumEntropy	CD200um wav LL firstorder Kurtosis	3.18×10^{-2}	-0.522	1.50×10^{2}
ADC wav LLH firstorder Mean	CD150um glcm DifferenceVariance	3.18×10^{-2}	0.522	1.50×10^{2}
ADC wav LLH glcm ClusterProminence	MEAN ODSum HaralickDifferenceVariance F9	3.19×10^{-2}	0.522	1.49×10^{2}
ADC wav HHH firstorder Median	CD150um ngtdm Strength	3.19×10^{-2}	0.522	1.49×10^{2}
ADC wav HLH firstorder Median	MEAN ODSum Haralick ASM F0	3.20×10^{-2}	0.522	1.48×10^{2}
ADC wav LHH firstorder Median	MEAN ODSum HaralickDifferenceVariance F9	3.21×10^{-2}	-0.522	1.47×10^{2}
ADC wav LLH glcm ClusterProminence	CD150um ngtdm Strength	3.23×10^{-2}	0.521	1.46×10^{2}
ADC wav HLL glcm ClusterProminence	CD200um wav HH firstorder Maximum	3.23×10^{-2}	0.521	1.46×10^{2}
ADC wav HLL glcm ClusterProminence	CD200um wav HL firstorder 90Percentile	3.23×10^{-2}	0.521	1.45×10^{2}
ADC glcm ClusterTendency	MEAN ODSum Haralick ASM F0	3.23×10^{-2}	0.521	1.45×10^{2}
ADC wav LHH firstorder Mean	MEAN ODSum HaralickDifferenceVariance F9	3.23×10^{-2}	-0.521	1.45×10^{2}

**Radiomic feature name**	**Pathomic feature name**	**ρ**	**Q-value**	**BF**
ADC wav HLL firstorder Median	MEAN ODSum HaralickDifferenceVariance F9	3.23×10^{-2}	0.521	1.45×10^{2}
ADC firstorder TotalEnergy	CD150um wav HH ngtdm Strength	3.25×10^{-2}	0.521	1.44×10^{2}
ADC wav HLH glszm GrayLevelVariance	CD150um ngtdm Strength	3.26×10^{-2}	0.521	1.43×10^{2}
ADC wav HHL glcm ClusterProminence	MEAN ODSum HaralickDifferenceVariance F9	3.26×10^{-2}	0.521	1.43×10^{2}
ADC wav HLL gldm LDHGLE	CD50um wav HL glcm ClusterShade	3.37×10^{-2}	0.52	1.38×10^{2}
ADC wav LHL glcm ClusterShade	MEAN ODSum HaralickDifferenceVariance F9	3.38×10^{-2}	-0.519	1.37×10^{2}
ADC glcm ClusterShade	CD200um ngtdm Strength	3.38×10^{-2}	0.519	1.37×10^{2}
ADC wav HHL glcm Idmn	MEAN ODSum Haralick ASM F0	3.38×10^{-2}	-0.519	1.37×10^{2}
ADC wav HHL firstorder Mean	MEAN ODSum HaralickDifferenceVariance F9	3.41×10^{-2}	-0.519	1.36×10^{2}
ADC firstorder TotalEnergy	CD200um wav HH firstorder 10Percentile	3.41×10^{-2}	-0.519	1.36×10^{2}
ADC wav HLL ngtdm Contrast	MEAN ODSum HaralickDifferenceVariance F9	3.41×10^{-2}	0.519	1.36×10^{2}
ADC wav LHL gldm LDHGLE	MEAN ODSum HaralickDifferenceVariance F9	3.42×10^{-2}	0.519	1.35×10^{2}
ADC wav LLL ngtdm Contrast	MEAN ODSum HaralickDifferenceVariance F9	3.42×10^{-2}	0.519	1.34×10^{2}
ADC wav HLH ngtdm Contrast	MEAN ODSum HaralickDifferenceVariance F9	3.42×10^{-2}	0.519	1.34×10^{2}
ADC wav LLH ngtdm Contrast	MEAN ODSum HaralickDifferenceVariance F9	3.42×10^{-2}	0.518	1.33×10^{2}
ADC wav LHL ngtdm Contrast	MEAN ODSum HaralickDifferenceVariance F9	3.42×10^{-2}	0.518	1.33×10^{2}
ADC wav HLL glcm ClusterProminence	CD50um ngtdm Complexity	3.42×10^{-2}	0.518	1.33×10^{2}
ADC wav HHH ngtdm Contrast	MEAN ODSum HaralickDifferenceVariance F9	3.42×10^{-2}	0.518	1.33×10^{2}
ADC wav LHH ngtdm Contrast	MEAN ODSum HaralickDifferenceVariance F9	3.42×10^{-2}	0.518	1.33×10^{2}
ADC lbp 3D k glszm ZoneEntropy	CD150um lbp 2D glcm ClusterTendency	3.43×10^{-2}	0.518	1.33×10^{2}
ADC wav LHH glcm ClusterShade	MEAN ODSum HaralickDifferenceVariance F9	3.44×10^{-2}	0.518	1.32×10^{2}
ADC wav LHH firstorder RootMeanSquared	MEAN ODSum HaralickDifferenceVariance F9	3.46×10^{-2}	0.518	1.31×10^{2}
ADC wav HHL glszm SALGLE	CD100um glrlm RunVariance	3.47×10^{-2}	0.518	1.30×10^{2}
ADC wav HHH glcm ClusterShade	MEAN ODSum HaralickDifferenceVariance F9	3.50×10^{-2}	0.518	1.29×10^{2}
ADC wav LLH firstorder RootMeanSquared	CD150um ngtdm Strength	3.50×10^{-2}	0.517	1.29×10^{2}
ADC wav LLH glcm ClusterShade	MEAN ODSum HaralickDifferenceVariance F9	3.54×10^{-2}	-0.517	1.27×10^{2}
ADC wav LLH gldm SDLGLE	MEAN ODSum HaralickDifferenceVariance F9	3.56×10^{-2}	0.517	1.27×10^{2}
ADC lbp 3D m2 glszm HGLZE	CD200um firstorder Kurtosis	3.61×10^{-2}	-0.516	1.25×10^{2}
ADC lbp 3D m2 glcm MaximumProbability	CD200um wav LL firstorder Kurtosis	3.61×10^{-2}	0.516	1.25×10^{2}

**Radiomic feature name**	**Pathomic feature name**	**ρ**	**Q-value**	**BF**
ADC wav LHL ngtdm Strength	MEAN ODSum HaralickDifferenceVariance F9	3.64×10^{-2}	0.516	1.23×10^{2}
ADC wav HHL glszm SALGLE	CD150um glszm LALGLE	3.65×10^{-2}	0.516	1.23×10^{2}
ADC lbp 2D gldm LDLGLE	CD100um lbp 2D glszm GLNUN	3.65×10^{-2}	0.516	1.23×10^{2}
ADC wav HLL ngtdm Complexity	CD50um ngtdm Complexity	3.66×10^{-2}	0.516	1.22×10^{2}
ADC wav HHL glcm ClusterShade	MEAN ODSum Haralick ASM F0	3.66×10^{-2}	0.516	1.22×10^{2}
ADC lbp 3D m1 glcm JointEnergy	CD200um wav LL firstorder Kurtosis	3.72×10^{-2}	0.515	1.20×10^{2}
ADC wav LHH firstorder Median	CD150um ngtdm Strength	3.76×10^{-2}	-0.515	1.18×10^{2}
ADC wav HLL firstorder Maximum	CD50um ngtdm Complexity	3.76×10^{-2}	0.515	1.18×10^{2}
ADC firstorder InterquartileRange	CD50um ngtdm Complexity	3.76×10^{-2}	0.515	1.18×10^{2}
ADC lbp 2D gldm LDLGLE	CD100um lbp 2D glcm SumEntropy	3.76×10^{-2}	-0.515	1.18×10^{2}
ADC lbp 2D glszm GLNUN	CD200um wav LL firstorder Kurtosis	3.79×10^{-2}	0.514	1.17×10^{2}
ADC wav LLH ngtdm Strength	MEAN ODSum HaralickDifferenceVariance F9	3.79×10^{-2}	0.514	1.17×10^{2}
ADC wav HLH glcm Idm	CD150um glszm LALGLE	3.79×10^{-2}	0.514	1.16×10^{2}
ADC wav LHH ngtdm Strength	MEAN ODSum HaralickDifferenceVariance F9	3.79×10^{-2}	0.514	1.16×10^{2}
ADC wav HLL ngtdm Complexity	CD200um wav HH glcm JointAverage	3.80×10^{-2}	0.514	1.16×10^{2}
ADC lbp 3D k glcm Correlation	MEAN ODSum HaralickDifferenceVariance F9	3.80×10^{-2}	-0.514	1.16×10^{2}
ADC wav HLL firstorder 10Percentile	CD50um ngtdm Complexity	3.80×10^{-2}	-0.514	1.15×10^{2}
ADC wav LLH firstorder Maximum	CD200um ngtdm Strength	3.80×10^{-2}	0.514	1.15×10^{2}
ADC wav HHL glszm SALGLE	CD100um lbp 2D glszm ZoneVariance	3.80×10^{-2}	0.514	1.15×10^{2}
ADC wav LHL firstorder RootMeanSquared	MEAN ODSum HaralickDifferenceVariance F9	3.80×10^{-2}	0.514	1.15×10^{2}
ADC wav HHH firstorder RootMeanSquared	MEAN ODSum HaralickDifferenceVariance F9	3.80×10^{-2}	0.514	1.15×10^{2}
ADC wav HLH firstorder RootMeanSquared	CD150um ngtdm Strength	3.80×10^{-2}	0.514	1.14×10^{2}
ADC glcm ClusterShade	MEAN ODSum Haralick IMOC2 F12	3.80×10^{-2}	-0.514	1.14×10^{2}
ADC wav HLH glszm GrayLevelVariance	MEAN ODSum HaralickDifferenceVariance F9	3.85×10^{-2}	0.513	1.13×10^{2}
ADC lbp 3D k glrlm LowGrayLevelRunEmphasis	CD200um ngtdm Strength	3.86×10^{-2}	0.513	1.12×10^{2}
ADC lbp 2D gldm LDLGLE	CD100um lbp 2D firstorder Uniformity	3.86×10^{-2}	0.513	1.12×10^{2}
ADC lbp 3D m2 glcm JointAverage	CD200um firstorder Kurtosis	3.92×10^{-2}	-0.512	1.10×10^{2}
ADC wav HHH firstorder RootMeanSquared	CD150um ngtdm Strength	3.92×10^{-2}	0.512	1.10×10^{2}
ADC wav LLL gldm LDHGLE	CD200um wav HH ngtdm Complexity	3.92×10^{-2}	0.512	1.10×10^{2}
ADC wav HLL glcm ClusterProminence	CD200um wav HH glcm JointAverage	3.94×10^{-2}	0.512	1.09×10^{2}
ADC wav LHH gldm LDHGLE	MEAN ODSum Haralick ASM F0	3.94×10^{-2}	0.512	1.08×10^{2}

**Radiomic feature name**	**Pathomic feature name**	**ρ**	**Q-value**	**BF**
ADC wav LHH ngtdm Coarseness	CD100um lbp 2D glcm JointEntropy	3.94×10^{-2}	-0.512	1.08×10^{2}
ADC firstorder TotalEnergy	CD50um wav HL glcm ClusterShade	3.94×10^{-2}	0.512	1.08×10^{2}
ADC wav HLL firstorder Mean	MEAN ODSum HaralickDifferenceVariance F9	3.94×10^{-2}	0.512	1.08×10^{2}
ADC wav HLL ngtdm Complexity	CD200um wav HL firstorder 90Percentile	3.94×10^{-2}	0.512	1.08×10^{2}
ADC wav LHL glcm DifferenceVariance	CD150um ngtdm Strength	3.94×10^{-2}	0.512	1.08×10^{2}
ADC wav HHL ngtdm Strength	MEAN ODSum HaralickDifferenceVariance F9	3.94×10^{-2}	0.512	1.08×10^{2}
ADC wav HHL glcm ClusterProminence	CD150um ngtdm Strength	3.94×10^{-2}	0.512	1.08×10^{2}
ADC wav HLH glszm LAHGLE	MEAN Cell EosinODMax	4.07×10^{-2}	0.511	1.05×10^{2}
ADC wav HLH ngtdm Complexity	CD100um glcm DifferenceVariance	4.07×10^{-2}	0.511	1.04×10^{2}
ADC wav LHH firstorder RootMeanSquared	CD150um ngtdm Strength	4.09×10^{-2}	0.511	1.04×10^{2}
ADC wav LLH ngtdm Strength	CD150um ngtdm Strength	4.13×10^{-2}	0.51	1.03×10^{2}
ADC wav LHL gldm LDHGLE	CD150um ngtdm Strength	4.13×10^{-2}	0.51	1.02×10^{2}
ADC wav HHL glcm Idmn	CD200um ngtdm Strength	4.14×10^{-2}	-0.51	1.02×10^{2}
ADC wav LLH gldm LDHGLE	CD150um ngtdm Strength	4.14×10^{-2}	0.51	1.02×10^{2}
ADC lbp 3D m1 gldm SDLGLE	CD200um ngtdm Strength	4.17×10^{-2}	0.51	1.01×10^{2}
ADC firstorder Minimum	MEAN ODSum HaralickDifferenceVariance F9	4.18×10^{-2}	0.51	1.01×10^{2}
ADC wav LHL glcm ClusterShade	CD150um ngtdm Strength	4.18×10^{-2}	-0.51	1.00×10^{2}
ADC wav HLH gldm SDLGLE	MEAN ODSum HaralickDifferenceVariance F9	4.19×10^{-2}	0.509	1.00×10^{2}
ADC wav LLL firstorder Maximum	CD100um ngtdm Strength	4.19×10^{-2}	0.509	1.00×10^{2}
ADC wav LLL gldm LDHGLE	CD150um wav HH ngtdm Strength	4.22×10^{-2}	0.509	9.92×10^{1}
ADC wav LHL firstorder Median	MEAN ODSum HaralickDifferenceVariance F9	4.22×10^{-2}	0.509	9.90×10^{1}
ADC wav LLL gldm LDHGLE	CD50um wav HL glcm ClusterShade	4.22×10^{-2}	0.509	9.88×10^{1}
ADC wav HLH firstorder Mean	CD200um wav HH glcm JointAverage	4.23×10^{-2}	-0.509	9.84×10^{1}
ADC wav HLH gldm DependenceVariance	CD100um glrlm RunVariance	4.23×10^{-2}	0.509	9.83×10^{1}
ADC wav LLL firstorder Minimum	MEAN ODSum HaralickDifferenceVariance F9	4.24×10^{-2}	0.509	9.79×10^{1}
ADC lbp 2D gldm LDLGLE	CD100um lbp 2D gldm DependenceEntropy	4.24×10^{-2}	-0.509	9.78×10^{1}
ADC glcm DifferenceAverage	MEAN ODSum Haralick IMOC2 F12	4.25×10^{-2}	-0.508	9.72×10^{1}
ADC wav HHH glszm LAHGLE	MEAN Cytoplasm HematoxylinODMin	4.25×10^{-2}	-0.508	9.71×10^{1}
ADC wav LHH glcm Idm	CD150um lbp 2D glrlm RunVariance	4.25×10^{-2}	0.508	9.71×10^{1}

**Radiomic feature name**	**Pathomic feature name**	**ρ**	**Q-value**	**BF**
ADC lbp 2D glszm ZoneVariance	MEAN Nucleus EosinODSum	4.25×10^{-2}	0.508	9.69×10^{1}
ADC lbp 3D m1 glcm Correlation	MEAN ODSum Haralick ASM F0	4.25×10^{-2}	-0.508	9.69×10^{1}
ADC wav HHH ngtdm Strength	MEAN ODSum HaralickDifferenceVariance F9	4.29×10^{-2}	0.508	9.59×10^{1}
ADC lbp 3D m1 glszm HGLZE	CD200um wav LL firstorder Kurtosis	4.29×10^{-2}	-0.508	9.58×10^{1}
ADC lbp 3D k glcm Correlation	CD150um lbp 2D glcm Idm	4.29×10^{-2}	-0.508	9.56×10^{1}
ADC wav LHH ngtdm Strength	CD150um ngtdm Strength	4.35×10^{-2}	0.507	9.42×10^{1}
ADC wav HLH gldm DependenceVariance	CD100um lbp 2D glszm ZoneVariance	4.35×10^{-2}	0.507	9.41×10^{1}
ADC wav HHL glszm LAHGLE	MEAN ODSum Haralick IMOC2 F12	4.38×10^{-2}	-0.507	9.27×10^{1}
ADC wav HLL gldm LDHGLE	CD200um wav HH glcm JointAverage	4.38×10^{-2}	0.507	9.25×10^{1}
ADC wav HLH ngtdm Contrast	CD150um ngtdm Strength	4.38×10^{-2}	0.507	9.24×10^{1}
ADC wav LLL gldm LDHGLE	CD200um wav HH glcm JointAverage	4.38×10^{-2}	0.507	9.24×10^{1}
ADC wav LLH ngtdm Contrast	CD150um ngtdm Strength	4.38×10^{-2}	0.507	9.23×10^{1}
ADC wav HHH glrlm LRHGLE	CD150um ngtdm Strength	4.38×10^{-2}	0.507	9.22×10^{1}
ADC wav LLL ngtdm Contrast	CD150um ngtdm Strength	4.38×10^{-2}	0.507	9.21×10^{1}
ADC wav HHH ngtdm Contrast	CD150um ngtdm Strength	4.38×10^{-2}	0.507	9.21×10^{1}
ADC wav LHL ngtdm Contrast	CD150um ngtdm Strength	4.38×10^{-2}	0.507	9.21×10^{1}
ADC wav LHH ngtdm Contrast	CD150um ngtdm Strength	4.38×10^{-2}	0.507	9.20×10^{1}
ADC wav LHH ngtdm Coarseness	CD100um lbp 2D firstorder Uniformity	4.39×10^{-2}	0.507	9.18×10^{1}
ADC wav HLL ngtdm Contrast	CD150um ngtdm Strength	4.43×10^{-2}	0.506	9.09×10^{1}
ADC wav LHH gldm LDHGLE	CD100um ngtdm Strength	4.46×10^{-2}	0.506	9.02×10^{1}
ADC lbp 3D m2 firstorder Kurtosis	MEAN Nucleus HematoxylinODSum	4.47×10^{-2}	0.506	8.97×10^{1}
ADC wav HLH glcm ClusterShade	MEAN ODSum HaralickDifferenceVariance F9	4.47×10^{-2}	-0.506	8.95×10^{1}
ADC wav LHH glcm ClusterShade	CD150um ngtdm Strength	4.47×10^{-2}	0.506	8.95×10^{1}
ADC wav HLH gldm DependenceVariance	CD150um lbp 2D glrlm RunVariance	4.50×10^{-2}	0.506	8.89×10^{1}
ADC wav HLL ngtdm Strength	CD150um wav HH glcm ClusterProminence	4.50×10^{-2}	0.506	8.88×10^{1}
ADC lbp 3D m2 glcm SumEntropy	CD200um ngtdm Strength	4.51×10^{-2}	-0.505	8.85×10^{1}
ADC wav HHH glcm ClusterShade	CD150um ngtdm Strength	4.60×10^{-2}	0.505	8.67×10^{1}
ADC wav LHL firstorder Median	CD150um ngtdm Strength	4.69×10^{-2}	0.504	8.50×10^{1}
ADC wav LLH glcm ClusterShade	CD150um ngtdm Strength	4.69×10^{-2}	-0.504	8.49×10^{1}
ADC wav HLH firstorder Range	CD50um wav HL glcm ClusterShade	4.70×10^{-2}	0.504	8.45×10^{1}
ADC lbp 3D m2 firstorder Median	CD200um wav HL firstorder Median	4.70×10^{-2}	-0.504	8.43×10^{1}

Radiomic feature name	Pathomic feature name	ρ	Q-value	BF
ADC lbp 3D k firstorder 10Percentile	CD200um ngtdm Strength	4.70×10^{-2}	0.504	8.43×10^{1}
ADC wav HHH glszm SizeZoneNonUniformityNormalized	CD50um wav HL glcm ClusterShade	4.70×10^{-2}	0.504	8.43×10^{1}
ADC wav HLL firstorder TotalEnergy	CD50um glcm DifferenceAverage	4.70×10^{-2}	0.504	8.41×10^{1}
ADC wav LLH glcm InverseVariance	CD150um lbp 2D glrlm RunVariance	4.70×10^{-2}	0.504	8.41×10^{1}
ADC wav HLL firstorder Mean	CD150um ngtdm Strength	4.71×10^{-2}	0.504	8.38×10^{1}
ADC lbp 3D m2 glszm LGLZE	MEAN ODSum Haralick ASM F0	4.73×10^{-2}	0.503	8.33×10^{1}
ADC wav LLL gldm LDHGLE	CD50um glcm DifferenceAverage	4.73×10^{-2}	0.503	8.32×10^{1}
ADC wav LLL firstorder Maximum	MEAN ODSum Haralick IMOC2 F12	4.74×10^{-2}	-0.503	8.29×10^{1}
ADC wav HLL ngtdm Strength	CD50um ngtdm Complexity	4.75×10^{-2}	0.503	8.27×10^{1}
ADC wav HLL firstorder TotalEnergy	CD50um ngtdm Complexity	4.76×10^{-2}	0.503	8.24×10^{1}
ADC wav HLL firstorder Maximum	CD200um wav HH firstorder InterquartileRange	4.78×10^{-2}	0.503	8.20×10^{1}
ADC wav LHL firstorder Mean	MEAN ODSum HaralickDifferenceVariance F9	4.81×10^{-2}	0.503	8.13×10^{1}
ADC firstorder TotalEnergy	CD200um wav HL firstorder 90Percentile	4.83×10^{-2}	0.502	8.08×10^{1}
ADC wav LLH firstorder 90Percentile	CD150um ngtdm Strength	4.85×10^{-2}	0.502	8.05×10^{1}
ADC wav HHH gldm SDLGLE	CD200um wav LH glcm ClusterShade	4.86×10^{-2}	-0.502	8.02×10^{1}
ADC wav HLL glcm ClusterProminence	CD200um wav HL ngtdm Strength	4.87×10^{-2}	0.502	8.00×10^{1}
ADC wav LLL gldm LDHGLE	CD50um ngtdm Complexity	4.88×10^{-2}	0.502	7.97×10^{1}
ADC lbp 3D k firstorder 10Percentile	MEAN ODSum Haralick IMOC2 F12	4.88×10^{-2}	-0.502	7.95×10^{1}
ADC lbp 3D m2 glrlm SRLGLE	MEAN ODSum Haralick ASM F0	4.94×10^{-2}	0.502	7.85×10^{1}
ADC wav HLL ngtdm Complexity	CD50um glcm DifferenceAverage	4.95×10^{-2}	0.501	7.83×10^{1}
ADC wav LHL firstorder Mean	CD150um ngtdm Strength	4.99×10^{-2}	0.501	7.75×10^{1}
ADC wav LLH firstorder Mean	MEAN ODSum Haralick IMOC2 F12	4.99×10^{-2}	-0.501	7.75×10^{1}
ADC wav HLH gldm SDLGLE	CD50um wav LL glcm Imc2	4.99×10^{-2}	-0.501	7.75×10^{1}
ADC wav LLH glrlm LRHGLE	MEAN ODSum HaralickDifferenceVariance F9	4.99×10^{-2}	0.501	7.73×10^{1}

Table S10 Summary of the significantly correlated radiomic-pathomic features, with radiomic features extracted from T1C. Abbreviations: T1C = post-contrast T1; LDLGLE = Large Dependence Low Gray Level Emphasis; LDHGLE = Large Dependence High Gray Level Emphasis; SDLGLE = Small Dependence Low Gray Level Emphasis; LALGLE = Large Area Low Gray Level Emphasis; GLNUN = Gray level non uniformity normalized; ASM = Angular Second Moment; IMOC = Information Measure of Correlation; glcm = gray level co-occurrence matrix; gldm = Gray

Level Dependence Matrix; glszm = Gray Level Size Zone Matrix; ngtdm = Neighbouring Gray Tone Difference Matrix; glrlm = Gray Level Run Length Matrix; wav = wavelet; lbp = local binary pattern; L = Low-pass filter; H = High-pass filter.

**Radiomic feature name**	**Pathomic feature name**	**ρ**	**Q-value**	**BF**
T1C wav HHL glcm ClusterProminence	CD200um wav HL gldm LDLGLE	3.45×10^{-6}	0.788	3.79×10^{8}
T1C wav HHH gldm LDHGLE	CD200um wav HL gldm LDLGLE	7.00×10^{-6}	0.774	9.79×10^{7}
T1C wav HHH glcm ClusterShade	CD200um wav HL gldm LDLGLE	1.31×10^{-5}	-0.762	3.61×10^{7}
T1C wav HHL gldm LDHGLE	CD200um wav HL gldm LDLGLE	4.03×10^{-5}	0.744	9.25×10^{6}
T1C wav HHL gldm SDLGLE	CD200um ngtdm Strength	5.19×10^{-4}	0.703	5.54×10^{5}
T1C wav HHL gldm LowGrayLevelEmphasis	CD200um ngtdm Strength	5.19×10^{-4}	0.702	5.16×10^{5}
T1C wav LLH ngtdm Contrast	CD200um ngtdm Strength	5.19×10^{-4}	0.7	4.61×10^{5}
T1C wav LHL gldm SDLGLE	CD200um ngtdm Strength	7.96×10^{-4}	0.692	2.69×10^{5}
T1C wav LLH gldm DependenceVariance	CD200um lbp 2D ngtdm Busyness	8.39×10^{-4}	0.689	2.28×10^{5}
T1C wav LLH gldm SDLGLE	CD200um ngtdm Strength	1.02×10^{-3}	0.683	1.65×10^{5}
T1C wav LLL gldm SDLGLE	MEAN ODSum Haralick IMOC2 F12	1.02×10^{-3}	-0.682	1.57×10^{5}
T1C wav LLH gldm DependenceVariance	CD150um wav HL glcm Imc2	1.27×10^{-3}	-0.677	1.17×10^{5}
T1C wav LLL gldm SDLGLE	MEAN ODSum HaralickASM F0	1.62×10^{-3}	0.672	8.57×10^{4}
T1C wav LLL gldm SDLGLE	CD200um ngtdm Strength	1.65×10^{-3}	0.67	7.83×10^{4}
T1C wav LLH ngtdm Contrast	CD100um ngtdm Strength	1.80×10^{-3}	0.667	6.74×10^{4}
T1C wav LHL gldm SDLGLE	MEAN ODSum Haralick IMOC2 F12	2.06×10^{-3}	-0.664	5.56×10^{4}
T1C wav LLH gldm DependenceVariance	CD50um ngtdm Busyness	2.28×10^{-3}	0.661	4.74×10^{4}
T1C wav HHL glrlm LRHGLE	CD200um wav HL gldm LDLGLE	2.28×10^{-3}	0.66	4.52×10^{4}
T1C wav LLH gldm DependenceVariance	CD200um wav LL glcm Imc2	2.34×10^{-3}	-0.658	4.18×10^{4}
T1C firstorder Kurtosis	CD50um wav LH glcm Imc2	2.39×10^{-3}	-0.657	3.90×10^{4}
T1C wav HHL gldm SDLGLE	MEAN ODSum Haralick IMOC2 F12	2.75×10^{-3}	-0.653	3.25×10^{4}
T1C firstorder RootMeanSquared	CD50um ngtdm Busyness	2.75×10^{-3}	-0.652	3.09×10^{4}
T1C wav LLH gldm DependenceVariance	CD200um firstorder TotalEnergy	2.75×10^{-3}	0.651	2.90×10^{4}
T1C firstorder Kurtosis	CD50um wav HL glcm Imc2	2.75×10^{-3}	-0.651	2.87×10^{4}
T1C wav LHL gldm SDLGLE	MEAN ODSum HaralickASM F0	2.78×10^{-3}	0.65	2.73×10^{4}
T1C wav LLH gldm DependenceVariance	CD50um wav HH glcm Imc2	3.13×10^{-3}	-0.647	2.35×10^{4}
T1C wav LHH gldm SDLGLE	MEAN ODSum Haralick IMOC2 F12	3.70×10^{-3}	-0.643	1.90×10^{4}
T1C wav HHL gldm LowGrayLevelEmphasis	MEAN ODSum Haralick IMOC2 F12	3.70×10^{-3}	-0.642	1.87×10^{4}
T1C wav LLL firstorder 10Percentile	CD50um ngtdm Busyness	3.90×10^{-3}	-0.641	1.72×10^{4}

**Radiomic feature name**	**Pathomic feature name**	**ρ**	**Q-value**	**BF**
T1C wav HLH gldm SDLGLE	MEAN ODSum Haralick IMOC2 F12	4.06×10^{-3}	-0.639	1.60×10^{4}
T1C firstorder Kurtosis	CD50um wav HH glcm Imc2	4.13×10^{-3}	-0.638	1.51×10^{4}
T1C wav HLH glrlm SRLGLE	MEAN ODSum Haralick IMOC2 F12	4.13×10^{-3}	-0.638	1.48×10^{4}
T1C lbp 3D m1 glcm DifferenceEntropy	CD200um ngtdm Strength	5.11×10^{-3}	-0.633	1.17×10^{4}
T1C wav HLH gldm SDLGLE	CD200um ngtdm Strength	5.63×10^{-3}	0.63	1.04×10^{4}
T1C wav HHL gldm SDLGLE	MEAN ODSum HaralickASM F0	6.12×10^{-3}	0.628	9.34×10^{3}
T1C lbp 3D m1 glcm SumEntropy	CD200um ngtdm Strength	6.50×10^{-3}	-0.626	8.59×10^{3}
T1C wav LLH glcm MaximumProbability	CD150um wav HL glcm Imc2	6.63×10^{-3}	-0.625	8.05×10^{3}
T1C wav HHL gldm LowGrayLevelEmphasis	MEAN ODSum HaralickASM F0	6.63×10^{-3}	0.624	7.90×10^{3}
T1C wav LLH glcm MaximumProbability	CD50um wav HH glcm Imc2	6.63×10^{-3}	-0.624	7.81×10^{3}
T1C wav LLH gldm DependenceVariance	CD200um glcm Imc2	6.85×10^{-3}	-0.623	7.39×10^{3}
T1C firstorder RootMeanSquared	CD200um glcm Imc2	7.38×10^{-3}	0.621	6.73×10^{3}
T1C wav LLH gldm DependenceVariance	CD50um wav HL glcm Imc2	7.50×10^{-3}	-0.62	6.47×10^{3}
T1C wav HLL gldm LDLGLE	MEAN ODSum Haralick IMOC2 F12	7.62×10^{-3}	-0.619	6.23×10^{3}
T1C wav HLH glrlm SRLGLE	CD200um ngtdm Strength	9.09×10^{-3}	0.615	5.16×10^{3}
T1C firstorder RootMeanSquared	CD200um wav LL glcm Imc2	9.29×10^{-3}	0.614	4.95×10^{3}
T1C wav LLH glcm MaximumProbability	CD200um lbp 2D ngtdm Busyness	1.01×10^{-2}	0.612	4.46×10^{3}
T1C wav LLH gldm SDLGLE	MEAN ODSum HaralickASM F0	1.08×10^{-2}	0.61	4.13×10^{3}
T1C wav LHH gldm DependenceVariance	CD200um glcm Imc2	1.08×10^{-2}	-0.609	4.04×10^{3}
T1C firstorder Kurtosis	CD150um wav HL glcm Imc2	1.12×10^{-2}	-0.608	3.79×10^{3}
T1C wav LLH glcm MaximumProbability	CD50um wav HL glcm Imc2	1.12×10^{-2}	-0.608	3.75×10^{3}
T1C wav HLH glrlm SRLGLE	MEAN ODSum HaralickASM F0	1.14×10^{-2}	0.607	3.61×10^{3}
T1C wav LLL firstorder 90Percentile	CD50um ngtdm Busyness	1.16×10^{-2}	-0.606	3.50×10^{3}
T1C wav LLH gldm DependenceVariance	CD50um wav LH glcm Imc2	1.19×10^{-2}	-0.605	3.35×10^{3}
T1C wav HHL gldm LowGrayLevelEmphasis	CD100um ngtdm Strength	1.29×10^{-2}	0.603	3.05×10^{3}
T1C wav LHL firstorder Median	CD100um ngtdm Strength	1.37×10^{-2}	-0.601	2.82×10^{3}
T1C lbp 2D glcm DifferenceEntropy	CD200um ngtdm Strength	1.39×10^{-2}	-0.6	2.70×10^{3}
T1C wav HHL gldm SDLGLE	CD100um ngtdm Strength	1.39×10^{-2}	0.6	2.70×10^{3}
T1C wav LHH gldm SDLGLE	MEAN ODSum HaralickASM F0	1.47×10^{-2}	0.598	2.46×10^{3}

Radiomic feature name	Pathomic feature name	ρ	Q-value	BF
T1C wav LLH glcm MaximumProbability	CD50um wav LH glcm Imc2	$1.47{\times}10^{-2}$	-0.598	$2.43{\times}10^{3}$
T1C firstorder RootMeanSquared	CD200um lbp 2D ngtdm Busyness	$1.47{\times}10^{-2}$	-0.597	$2.42{\times}10^{3}$
T1C wav LLL firstorder 10Percentile	CD200um lbp 2D ngtdm Busyness	$1.47{\times}10^{-2}$	-0.597	$2.40{\times}10^{3}$
T1C wav LLH glcm MaximumProbability	CD200um firstorder TotalEnergy	$1.53{\times}10^{-2}$	0.596	$2.27{\times}10^{3}$
T1C wav HLL gldm SDLGLE	CD200um ngtdm Strength	$1.58{\times}10^{-2}$	0.595	$2.18{\times}10^{3}$
T1C wav LHH gldm DependenceVariance	CD200um wav LL glcm Imc2	$1.62{\times}10^{-2}$	-0.594	$2.10{\times}10^{3}$
T1C wav HHH ngtdm Strength	CD50um wav HH firstorder Uniformity	$1.62{\times}10^{-2}$	0.594	$2.05{\times}10^{3}$
T1C wav LLH gldm SDLGLE	MEAN ODSum Haralick IMOC2 F12	$1.77{\times}10^{-2}$	-0.591	$1.85{\times}10^{3}$
T1C wav LHH glszm GLNUN	CD200um lbp 2D ngtdm Busyness	$1.77{\times}10^{-2}$	0.591	$1.84{\times}10^{3}$
T1C glcm MaximumProbability	MEAN ODSum Haralick IMOC2 F12	$1.80{\times}10^{-2}$	-0.59	$1.78{\times}10^{3}$
T1C wav LLH gldm DependenceVariance	CD200um wav HL firstorder TotalEnergy	$1.83{\times}10^{-2}$	0.589	$1.73{\times}10^{3}$
T1C lbp 3D m2 glcm SumEntropy	CD200um ngtdm Strength	$1.92{\times}10^{-2}$	-0.588	$1.63{\times}10^{3}$
T1C wav HHH firstorder Kurtosis	CD200um wav HL gldm LDLGLE	$2.03{\times}10^{-2}$	0.586	$1.53{\times}10^{3}$
T1C wav LLL firstorder 10Percentile	CD200um glcm Imc2	$2.04{\times}10^{-2}$	0.586	$1.50{\times}10^{3}$
T1C lbp 3D m1 firstorder Uniformity	CD200um ngtdm Strength	$2.07{\times}10^{-2}$	0.585	$1.46{\times}10^{3}$
T1C wav HLL gldm LDLGLE	MEAN ODSum HaralickASM F0	$2.10{\times}10^{-2}$	0.585	$1.43{\times}10^{3}$
T1C wav LHH glcm MaximumProbability	CD200um lbp 2D ngtdm Busyness	$2.17{\times}10^{-2}$	0.583	$1.36{\times}10^{3}$
T1C lbp 2D glcm SumEntropy	CD200um ngtdm Strength	$2.22{\times}10^{-2}$	-0.583	$1.32{\times}10^{3}$
T1C wav HHH ngtdm Strength	CD150um glcm JointEnergy	$2.29{\times}10^{-2}$	0.582	$1.26{\times}10^{3}$
T1C lbp 3D m2 glcm DifferenceEntropy	CD200um ngtdm Strength	$2.42{\times}10^{-2}$	-0.58	$1.19{\times}10^{3}$
T1C wav LLL firstorder 10Percentile	CD100um ngtdm Busyness	$2.42{\times}10^{-2}$	-0.58	$1.17{\times}10^{3}$
T1C wav LLL firstorder 90Percentile	CD200um wav LL glcm Imc2	$2.48{\times}10^{-2}$	0.579	$1.13{\times}10^{3}$
T1C wav LLL firstorder 10Percentile	CD200um wav LL glcm Imc2	$2.62{\times}10^{-2}$	0.577	$1.06{\times}10^{3}$
T1C wav LLL firstorder Minimum	CD200um ngtdm Strength	$2.72{\times}10^{-2}$	0.576	$9.97{\times}10^{2}$
T1C wav HLH gldm SDLGLE	MEAN ODSum HaralickASM F0	$2.72{\times}10^{-2}$	0.575	$9.83{\times}10^{2}$
T1C wav LHL gldm SDLGLE	CD100um ngtdm Strength	$2.72{\times}10^{-2}$	0.575	$9.83{\times}10^{2}$
T1C lbp 2D gldm SDHGLE	CD200um ngtdm Strength	$2.72{\times}10^{-2}$	-0.575	$9.78{\times}10^{2}$
T1C wav LLL firstorder 90Percentile	CD200um glcm Imc2	$2.75{\times}10^{-2}$	0.574	$9.58{\times}10^{2}$
T1C wav LHH gldm DependenceVariance	CD200um lbp 2D ngtdm Busyness	$2.80{\times}10^{-2}$	0.574	$9.33{\times}10^{2}$
T1C wav LLL firstorder 10Percentile	CD200um ngtdm Strength	$2.83{\times}10^{-2}$	0.573	$9.13{\times}10^{2}$
T1C wav HHH firstorder Skewness	MEAN Delaunay MaxTriangleArea	$2.88{\times}10^{-2}$	-0.572	$8.84{\times}10^{2}$

**Radiomic feature name**	**Pathomic feature name**	**ρ**	**Q-value**	**BF**
T1C wav HHH glcm MaximumProbability	CD200um lbp 2D ngtdm Busyness	2.88×10^{-2}	0.572	8.74×10^{2}
T1C wav HHH ngtdm Strength	CD100um lbp 2D glcm SumEntropy	2.88×10^{-2}	-0.572	8.70×10^{2}
T1C glcm MaximumProbability	CD200um ngtdm Strength	2.89×10^{-2}	0.572	8.59×10^{2}
T1C wav HHH ngtdm Strength	CD50um wav LL ngtdm Busyness	2.90×10^{-2}	0.571	8.46×10^{2}
T1C glrlm SRLGLE	CD200um ngtdm Strength	2.99×10^{-2}	0.57	8.15×10^{2}
T1C firstorder RootMeanSquared	CD100um ngtdm Busyness	3.15×10^{-2}	-0.569	7.69×10^{2}
T1C wav LLH firstorder Kurtosis	CD50um ngtdm Busyness	3.18×10^{-2}	0.568	7.51×10^{2}
T1C wav LHH gldm DependenceVariance	CD50um ngtdm Busyness	3.18×10^{-2}	0.568	7.45×10^{2}
T1C wav LHH glszm GLNUN	CD200um firstorder TotalEnergy	3.23×10^{-2}	0.567	7.28×10^{2}
T1C wav LHH glszm GLNUN	CD150um wav HL glcm Imc2	3.28×10^{-2}	-0.567	7.10×10^{2}
T1C wav LLH ngtdm Contrast	CD150um ngtdm Strength	3.43×10^{-2}	0.565	6.74×10^{2}
T1C wav LLL glcm ClusterShade	CD50um wav HL glcm ClusterShade	3.46×10^{-2}	0.565	6.63×10^{2}
T1C wav HLL gldm DependenceVariance	CD150um wav HL glcm Imc2	3.49×10^{-2}	-0.564	6.52×10^{2}
T1C wav LLH glcm MaximumProbability	CD50um ngtdm Busyness	3.56×10^{-2}	0.564	6.34×10^{2}
T1C wav LLH firstorder Median	CD100um ngtdm Strength	3.56×10^{-2}	-0.563	6.23×10^{2}
T1C firstorder RootMeanSquared	CD150um wav HL glcm Imc2	3.56×10^{-2}	0.563	6.22×10^{2}
T1C wav HHH ngtdm Strength	CD200um wav HL gldm LDLGLE	3.57×10^{-2}	0.563	6.15×10^{2}
T1C lbp 3D m1 glszm GrayLevelVariance	CD200um ngtdm Strength	3.67×10^{-2}	-0.562	5.94×10^{2}
T1C glrlm SRLGLE	MEAN ODSum Haralick IMOC2 F12	3.74×10^{-2}	-0.561	5.78×10^{2}
T1C firstorder Minimum	CD200um ngtdm Strength	3.76×10^{-2}	0.561	5.70×10^{2}
T1C wav HLH glcm Idmn	CD200um ngtdm Strength	3.76×10^{-2}	-0.561	5.66×10^{2}
T1C lbp 3D k firstorder Maximum	CD200um wav LH glcm ClusterShade	3.81×10^{-2}	0.56	5.48×10^{2}
T1C wav LHH glcm MaximumProbability	CD200um firstorder TotalEnergy	3.81×10^{-2}	0.56	5.46×10^{2}
T1C wav LHH glcm MaximumProbability	CD50um ngtdm Busyness	3.81×10^{-2}	0.559	5.44×10^{2}
T1C wav HHH firstorder Median	CD50um glcm ClusterProminence	3.99×10^{-2}	-0.558	5.18×10^{2}
T1C wav LLL glcm ClusterShade	CD200um wav HL glcm ClusterProminence	4.23×10^{-2}	0.556	4.86×10^{2}
T1C wav LLH firstorder Kurtosis	CD200um lbp 2D ngtdm Busyness	4.23×10^{-2}	0.556	4.78×10^{2}
T1C wav LLH glszm SALGLE	CD200um ngtdm Strength	4.23×10^{-2}	0.556	4.77×10^{2}
T1C wav LHH glszm GLNUN	CD50um ngtdm Busyness	4.24×10^{-2}	0.556	4.73×10^{2}

Radiomic feature name	Pathomic feature name	ρ	Q-value	BF
T1C lbp 3D m2 glszm GLNUN	CD200um ngtdm Strength	4.34×10^{-2}	0.555	4.59×10^{2}
T1C wav LLH glcm Idn	CD200um ngtdm Strength	4.36×10^{-2}	-0.554	4.54×10^{2}
T1C glrlm SRLGLE	MEAN ODSum HaralickASM F0	4.37×10^{-2}	0.554	4.49×10^{2}
T1C wav HLL gldm DependenceEntropy	MEAN ODSum Haralick IMOC2 F12	4.43×10^{-2}	0.554	4.40×10^{2}
T1C wav LLL firstorder 90Percentile	CD200um lbp 2D ngtdm Busyness	4.45×10^{-2}	-0.553	4.34×10^{2}
T1C lbp 2D glcm DifferenceEntropy	MEAN ODSum Haralick IMOC2 F12	4.51×10^{-2}	0.553	4.23×10^{2}
T1C wav HHH ngtdm Strength	CD50um wav HH ngtdm Busyness	4.51×10^{-2}	0.553	4.22×10^{2}
T1C wav LLH ngtdm Contrast	CD100um lbp 2D gldm SDHGLE	4.89×10^{-2}	-0.55	3.89×10^{2}
T1C wav LLL gldm LDLGLE	MEAN ODSum Haralick IMOC2 F12	4.91×10^{-2}	-0.55	3.85×10^{2}

4.2.2 Factor analysis

The factor analysis resulted in 24, 32, and 27 factors, respectively for ADC, T1C and pathomic. These results were obtained after features with cross-loadings lower than 0.4. These retained 79%, 78% and 76% of the covariation between the original 323 (for ADC), 341 (for T1C) and 332 (for pathomic) features. Hence, the factor solution was deemed to sufficiently represent the original feature-spaces. Factor loadings associated with ADC, T1C and pathomic features were reported in Table S11, S12 and S13, respectively.
Correlation analysis between factor scores revealed significant correlations based on adjusted p-values after FDR correction. Concerning ADC pipeline, 5 significant positive correlations were found. Concerning T1C pipeline, 3 significant correlations were found, of which 2 positive and 1 negative. Radiopathomic associations between factors sorted by ρ strength are presented in Table S14 and S15.

Table S11 Factor loadings for the 24 ADC factors. A darker grey in the column cell correspond to a higher loading measuring the association between features and factors. ADC = Apparent Diffusion Coefficient; LALGLE = Large Area Low Gray Level Emphasis; LDLGLE = Large Dependence Low Gray Level Emphasis; LDHGLE = Large Dependence High Gray Level Emphasis; SDLGLE = Small Dependence Low Gray Level Emphasis; LALGLE = Large Area Low Gray Level Emphasis; GLNUN = Gray level non uniformity normalized; SZNU = Size Zone Non-Uniformity; glcm = gray level co-occurrence matrix; gldm = Gray Level Dependence Matrix; glszm = Gray Level Size Zone Matrix; ngtdm = Neighbouring Gray Tone Difference Matrix; glrlm = Gray Level Run Length Matrix; wav = wavelet; lbp = local binary pattern; L = Low-pass filter; H = High-pass filter.

ADC feature name	ADC F1	ADC F2	ADC F3	ADC F4	ADC F5	ADC F6	ADC F7	ADC F8	ADC F9	ADC F10	ADC F11	ADC F12	ADC F13	ADC F14	ADC F15	ADC F16	ADC F17	ADC F18	ADC F19	ADC F20	ADC F21	ADC F22	ADC F23	ADC F24
ADC firstorder Minimum	0.774	-0.016	-0.02	-0.028	0.059	0.041	-0.063	-0.053	-0.081	-0.104	0.014	-0.018	0.015	0.023	0.013	-0.056	-0.015	0.008	-0.072	-0.012	0.01	-0.02	-0.001	-0.051
ADC firstorder Kurtosis	-0.045	-0.002	0.147	0.14	-0.013	-0.052	0.214	0.171	0.141	0.169	0.642	-0.025	0.026	0	-0.021	-0.063	-0.153	0.052	0.033	0.039	0.033	0.146	0.002	0.019
ADC firstorder 10Percentile	0.786	-0.047	-0.002	-0.016	0.118	0.014	-0.055	-0.011	-0.025	-0.019	0.009	0.006	0.02	0.011	0	-0.065	-0.019	0.015	-0.033	0.001	0.032	0.011	-0.006	-0.035
ADC firstorder InterquartileRange	0.599	-0.138	-0.084	-0.021	0.458	-0.083	0.029	-0.061	0.018	-0.019	-0.06	0.043	-0.044	-0.015	-0.012	0.098	0.102	-0.039	0.068	0.055	-0.012	-0.005	-0.012	-0.004
ADC firstorder Median	0.775	-0.062	-0.01	-0.017	0.176	0.004	-0.053	-0.016	-0.015	-0.022	-0.003	0.011	0.02	0.004	-0.004	-0.055	-0.006	0.012	-0.024	0.004	0.037	0.013	-0.011	-0.042
ADC firstorder Range	0.553	-0.158	-0.037	0.048	0.501	-0.097	0.085	0.062	0.069	0.059	0.061	0.042	-0.017	-0.016	0.004	0.041	0.051	-0.015	0.091	0.097	0.014	0.057	-0.005	0.015

ADC feature name	ADC F1	ADC F2	ADC F3	ADC F4	ADC F5	ADC F6	ADC F7	ADC F8	ADC F9	ADC F10	ADC F11	ADC F12	ADC F13	ADC F14	ADC F15	ADC F16	ADC F17	ADC F18	ADC F19	ADC F20	ADC F21	ADC F22	ADC F23	ADC F24
ADC firstorder TotalEnergy	0.233	-0.077	-0.022	0.129	0.671	0.034	-0.103	0.131	-0.046	-0.008	0.02	0.026	-0.009	0.047	0.002	-0.161	-0.042	0.068	-0.067	0.065	0.062	-0.006	-0.015	-0.082
ADC gldm LDLGLE	0.009	0.794	-0.048	-0.058	0.027	0.03	-0.011	0.001	0.01	-0.02	-0.04	0.015	0.008	-0.004	0.029	0.028	-0.003	-0.018	0.076	0.006	0.006	-0.005	-0.007	-0.017
ADC gldm DependenceEntropy	-0.244	-0.26	-0.099	0.379	0.433	-0.056	0.172	0.264	0.121	0.028	-0.026	0.063	-0.113	-0.019	0.006	0.086	0.064	-0.069	0.101	0.056	0.02	0.053	0.051	-0.005
ADC gldm GLNU	-0.119	0.559	0.152	0.23	-0.064	0.037	0.088	0.399	-0.028	0.106	0.165	-0.003	0.003	-0.043	-0.036	0.005	-0.009	0.051	-0.05	-0.047	-0.034	-0.012	-0.002	-0.047
ADC gldm LDHGLE	0.604	-0.086	0.016	0.04	0.41	-0.07	-0.002	0.04	0.131	0.222	-0.023	0.057	-0.008	-0.016	-0.02	-0.029	0.016	0.038	0.069	0.04	0.078	0.048	-0.011	0.032
ADC gldm DependenceVariance	-0.109	0.773	0.053	0.121	-0.054	0.058	0.052	0.027	0.002	0.023	-0.001	-0.011	-0.03	0.021	-0.006	0.019	0.006	0.016	0.004	-0.052	-0.018	-0.011	0.03	0.022
ADC ngtdm Busyness	-0.024	0.794	-0.035	-0.047	0.01	0.051	0.007	0.039	-0.012	-0.032	-0.039	-0.004	0.022	-0.017	0.036	0.033	0.002	-0.006	0.055	-0.013	0.018	-0.013	0.006	-0.019
ADC glcm Imc2	0.032	-0.794	-0.006	0.016	-0.027	-0.029	0.001	-0.083	0.001	0.003	0.006	-0.001	-0.021	0.022	-0.011	-0.029	-0.004	0.014	-0.052	0.009	-0.011	0	0.001	0.037
ADC glcm InverseVariance	-0.2	0.717	0.081	-0.009	-0.179	0.096	-0.029	0.003	0.007	-0.002	0.023	-0.05	-0.042	0.038	0.026	-0.033	-0.019	0.095	-0.082	-0.103	-0.019	-0.02	0.034	0.059
ADC glcm ClusterShade	0.674	-0.033	-0.016	0.004	0.39	0.002	-0.032	-0.052	-0.006	-0.033	0.051	-0.009	-0.043	0.055	0.035	0.016	0.092	-0.004	-0.048	0.054	0.019	0.024	0.016	0.02
ADC glcm Correlation	-0.431	0.039	-0.086	0.299	0.028	-0.061	-0.038	0.123	0.105	-0.002	0.018	0.033	-0.503	0.008	-0.048	-0.04	0.076	0.023	-0.041	-0.003	-0.042	-0.076	-0.013	0.008
ADC glcm DifferenceEntropy	-0.123	-0.463	-0.155	0.188	0.482	-0.053	0.135	0.116	0.104	-0.003	-0.025	0.046	0.04	-0.015	0.023	0.09	0.058	-0.12	0.157	0.126	0.046	0.033	0.005	0.01
ADC glcm ClusterTendency	0.612	-0.066	-0.031	-0.014	0.498	-0.047	-0.012	-0.061	0.012	-0.036	0.01	0.012	-0.039	0.012	0.008	0.041	0.082	-0.005	-0.004	0.042	0.006	0.001	0.003	0.018
ADC glcm DifferenceAverage	0.701	-0.106	-0.057	-0.053	0.337	-0.07	0.033	-0.052	-0.005	-0.032	-0.009	0.038	0.02	-0.027	-0.007	0.064	0.053	-0.028	0.054	0.041	0.004	0.015	-0.009	-0.011
ADC glcm Idmn	-0.411	-0.027	0.138	0.314	0.03	-0.001	0.083	0.387	0.145	0.146	0.121	-0.073	-0.199	-0.024	0.032	-0.127	-0.079	0.104	0.012	-0.038	-0.028	0.148	0.033	0.113
ADC glszm GLNUN	0.062	0.742	0.076	-0.158	-0.156	0.075	-0.083	-0.044	-0.043	-0.017	0.016	-0.028	0.061	0.023	0.017	-0.035	-0.019	0.054	-0.033	-0.043	-0.007	-0.034	-0.017	0.035
ADC wav LLH firstorder Maximum	0.672	-0.099	-0.027	-0.014	0.394	-0.042	0.035	0.001	-0.022	0.066	0.056	0.044	-0.013	-0.032	-0.032	0.068	-0.029	0.016	0.006	0.007	0.032	0.052	0.006	0.021
ADC wav LLH firstorder 10Percentile	-0.785	0.055	0.019	0.032	-0.139	0.048	-0.001	0.019	0.008	0.009	0.017	-0.01	-0.003	-0.006	0.00E+00	-0.008	-0.003	0.017	-0.051	-0.003	0.004	0.036	-0.009	-0.019
ADC wav LLH firstorder RootMeanSquared	0.774	-0.025	-0.02	-0.041	0.197	0.021	-0.001	-0.047	-0.039	-0.023	0.002	-0.006	0	-0.004	-0.026	-0.011	-0.03	-0.002	-0.029	-0.012	0.01	-0.012	0.006	-0.003
ADC wav LLH firstorder Median	0.788	0	-0.029	-0.051	0.028	0.07	0.01	-0.058	-0.043	-0.028	0.011	-0.03	0.008	0.002	-0.043	-0.009	-0.042	-0.019	-0.06	-0.009	-0.002	-0.02	0.012	-0.008
ADC wav LLH firstorder 90Percentile	0.755	-0.059	-0.041	-0.035	0.255	0.019	0.003	-0.04	-0.042	-0.027	-0.006	-0.001	-0.003	-0.002	-0.019	-0.01	-0.032	-0.005	-0.009	-0.002	0.016	-0.016	0.003	-0.004
ADC wav LLH firstorder Skewness	-0.109	0.131	0.086	0.202	0.027	0	-0.024	0.134	-0.041	0.044	0.153	0.039	0.035	0.007	-0.059	0.002	0.052	0.015	-0.015	0.047	0.066	0.571	-0.034	-0.003
ADC wav LLH firstorder Mean	0.688	-0.003	-0.065	-0.046	0.214	0.189	0.014	-0.089	-0.1	-0.059	0.027	-0.046	0.007	0.003	-0.078	-0.048	-0.092	0.002	-0.161	-0.016	0.037	0.029	0.01	-0.044
ADC wav LLH gldm LDLGLE	-0.008	0.795	-0.039	-0.05	0.032	0.018	-0.006	0.01	0.001	-0.02	-0.039	0.008	0.013	-0.003	0.021	0.025	-0.002	-0.022	0.079	0.008	0.03	0.008	-0.005	-0.01
ADC wav LLH gldm DependenceEntropy	-0.204	-0.288	-0.11	0.393	0.459	0.001	0.149	0.24	0.065	0.084	0.022	0.014	-0.058	0.002	-0.003	0.105	-0.034	-0.018	0.125	0.036	0.03	-0.023	0.101	0.042
ADC wav LLH gldm LDHGLE	0.762	-0.032	0.01	0.025	0.226	-0.014	0.015	-0.027	-0.025	0.089	-0.002	-0.011	-0.031	0.012	0.009	0.002	0.014	-0.004	-0.024	-0.011	0.003	-0.027	0.003	0.023
ADC wav LLH gldm DependenceVariance	-0.103	0.74	0.124	0.128	-0.037	0.007	0.059	-0.003	0.03	0.051	0.158	-0.057	0.021	0.061	0.015	0.05	0.054	-0.025	-0.017	-0.018	-0.046	0.063	0.014	-0.02
ADC wav LLH gldm SDLGLE	0.602	0.268	0.059	-0.204	-0.158	-0.042	-0.103	-0.281	-0.086	-0.056	-0.04	0.036	0.03	0.046	-0.048	-0.033	-0.015	-0.009	-0.035	0.09	-0.017	-0.038	-0.091	0.021
ADC wav LLH ngtdm Busyness	-0.036	0.795	-0.011	-0.02	0.011	0.033	-0.009	0.06	0.005	-0.036	-0.041	0.006	0.021	-0.014	0.013	0.017	0.01	-0.018	0.054	-0.005	0.023	0.012	-0.014	-0.026

ADC feature name	ADC F1	ADC F2	ADC F3	ADC F4	ADC F5	ADC F6	ADC F7	ADC F8	ADC F9	ADC F10	ADC F11	ADC F12	ADC F13	ADC F14	ADC F15	ADC F16	ADC F17	ADC F18	ADC F19	ADC F20	ADC F21	ADC F22	ADC F23	ADC F24
ADC wav LLH ngtdm Strength	0.797	-0.017	0.003	-0.059	0.081	-0.024	-0.006	-0.05	-0.028	-0.012	0.007	0.001	0	-0.012	-0.008	0.005	-0.008	0	-0.002	-0.011	-0.001	-0.004	0.006	0.002
ADC wav LLH ngtdm Contrast	0.799	0.008	0.022	-0.037	-0.094	-0.015	0.001	-0.011	-0.016	-0.017	0.001	-0.001	0.007	0.002	0.001	-0.003	0.004	-0.009	0.005	-0.007	-0.003	-0.005	0.005	-0.001
ADC wav LLH glrlm LRHGLE	0.779	-0.018	0.002	-0.026	0.189	-0.001	-0.009	-0.033	-0.033	0.01	-0.005	-0.005	-0.006	0	-0.007	-0.028	-0.015	0.006	-0.03	-0.024	0.007	-0.016	-0.002	-0.001
ADC wav LLH glcm InverseVariance	-0.249	0.671	0.167	0.038	-0.205	0.023	-0.012	-0.024	0.029	0.027	0.074	-0.05	0.011	0.04	0.041	-0.033	0.033	0.005	-0.179	-0.01	-0.035	0.07	-0.007	0.001
ADC wav LLH glcm JointEntropy	-0.273	-0.406	-0.036	0.409	0.196	0.054	0.104	0.385	0.079	0.035	-0.055	0.033	-0.058	-0.023	0.024	0.017	-0.03	-0.014	0.087	0.013	0.063	-0.023	0.037	0.061
ADC wav LLH glcm ClusterShade	-0.798	-0.008	-0.023	0.037	0.098	0.018	-0.002	0.011	0.015	0.017	0	0	-0.007	-0.002	-0.001	0	-0.005	0.011	-0.008	0.006	0.005	0.007	-0.005	0.00E+00
ADC wav LLH glcm Correlation	-0.107	-0.021	-0.321	0.204	0.115	-0.076	-0.04	0.152	0.063	-0.003	0.151	0.056	0.01	-0.018	-0.144	-0.02	0.007	-0.025	-0.027	0.523	-0.046	0.088	-0.001	0.021
ADC wav LLH glcm DifferenceEntropy	0.008	-0.507	-0.161	0.082	0.516	-0.016	0.07	0.067	0.025	0.02	-0.065	0.032	-0.034	-0.026	-0.015	0.077	-0.034	-0.042	0.2	0.014	0.057	-0.071	0.02	0.067
ADC wav LLH glcm SumEntropy	-0.191	-0.47	-0.176	0.224	0.438	0.017	0.076	0.188	0.069	0.024	-0.058	0.032	-0.04	-0.008	0.001	0.062	-0.028	-0.044	0.164	0.069	0.076	-0.056	0.031	0.086
ADC wav LLH glcm MaximumProbability	0.075	0.779	0.006	-0.142	-0.039	0.003	-0.023	-0.089	-0.019	-0.008	-0.009	0.003	0.012	0.033	0.023	0.01	0.02	-0.006	0.025	0.029	-0.01	0.012	-0.02	-0.026
ADC wav LLH glcm ClusterProminence	0.803	0.005	0.018	-0.037	-0.034	-0.007	-0.004	-0.018	-0.02	-0.018	0.003	-0.004	0.007	0.003	-0.001	-0.015	-0.002	-0.005	-0.01	-0.013	0	-0.004	0.003	-0.005
ADC wav LLH glszm LAHGLE	0.765	0.009	0.035	0.032	0.205	-0.016	0.029	-0.025	-0.023	0.087	0.065	-0.01	-0.023	0.014	0.001	0.001	0.012	-0.014	-0.043	-0.018	-0.007	-0.013	0.003	0.002
ADC wav HHH firstorder Maximum	0.746	-0.086	0.025	-0.027	0.153	-0.033	0.058	0.046	0.107	0.141	-0.067	0	0.018	0.00E+00	0.01	0.007	-0.028	-0.014	0.089	-0.012	0.031	-0.021	-0.027	-0.005
ADC wav HHH firstorder Kurtosis	-0.076	-0.08	0.018	0.157	-0.028	-0.098	0.046	0.177	0.29	0.563	-0.054	0.002	0.114	-0.067	0.032	-0.059	0.046	0.044	0.047	0.067	0.121	0.11	0.017	-0.014
ADC wav HHH firstorder RootMeanSquared	0.803	-0.006	0.021	-0.044	0.007	-0.016	0.002	-0.02	-0.006	-0.013	-0.008	-0.004	0.01	0.001	-0.002	-0.007	-0.006	-0.009	0.008	-0.014	-0.002	-0.015	-0.001	-0.001
ADC wav HHH firstorder Median	0.777	-0.005	0.021	-0.077	0.045	-0.001	-0.03	-0.051	-0.071	-0.041	0.006	-0.004	-0.007	0.042	0	-0.018	-0.064	-0.025	-0.023	0.017	-0.06	-0.021	0.008	0.027
ADC wav HHH firstorder Skewness	0.008	0.023	0.243	0.023	-0.047	-0.004	0.201	0.068	-0.063	-0.072	0.125	0.076	-0.046	-0.002	0.051	-0.027	-0.571	0.157	-0.001	-0.011	-0.037	-0.021	-0.061	-0.04
ADC wav HHH firstorder Mean	-0.652	-0.017	0.004	-0.025	0.237	0.052	0	-0.009	-0.047	-0.04	-0.025	-0.02	0.071	0.063	0.026	-0.158	-0.246	0.011	-0.065	0.009	0.036	-0.008	-0.106	-0.013
ADC wav HHH gldm LDLGLE	-0.023	0.796	-0.045	-0.041	0.032	0.024	-0.007	0.008	0.002	-0.021	-0.04	0.013	0.006	-0.013	0.007	0.024	-0.003	-0.026	0.07	0.019	0.027	0.002	-0.007	-0.009
ADC wav HHH gldm DependenceEntropy	-0.076	-0.378	0.102	0.254	0.498	-0.012	0.154	0.213	0.144	0.106	-0.059	0.03	-0.035	-0.024	0.084	0.114	-0.004	-0.073	0.149	-0.03	0	-0.034	0.017	0.022
ADC wav HHH gldm LDHGLE	0.645	-0.059	0.029	0.106	0.153	-0.048	0.055	0.04	0.096	0.36	-0.099	-0.011	-0.023	0.026	0.067	0.03	0.122	-0.05	0.058	0.024	0.03	-0.056	-0.021	0.056
ADC wav HHH gldm DependenceVariance	-0.154	0.563	0.045	0.293	-0.146	-0.018	0.048	-0.024	-0.018	0.188	0.175	0.014	-0.027	-0.111	-0.049	-0.057	0.001	-0.018	-0.237	0.13	-0.087	0.043	0.051	0.081
ADC wav HHH ngtdm Strength	0.801	-0.01	0.025	-0.053	-0.028	-0.029	0.008	-0.028	0.005	0.007	-0.012	0.00E+00	0.014	-0.006	0	0.013	0	-0.005	0.027	-0.012	0.001	-0.012	-0.004	-0.003
ADC wav HHH ngtdm Contrast	0.799	0.008	0.022	-0.037	-0.095	-0.015	0.001	-0.011	-0.016	-0.017	0.001	-0.001	0.007	0.002	0.001	-0.003	0.004	-0.009	0.005	-0.007	-0.003	-0.005	0.005	-0.001
ADC wav HHH glrlm LRHGLE	0.796	-0.022	0.014	-0.029	0.085	-0.015	0.011	-0.013	0.034	0.036	-0.029	-0.006	0.01	0.005	0.008	-0.009	0.029	-0.017	0.019	-0.008	0.016	-0.018	-0.009	0.003
ADC wav HHH glrlm SRLGLE	0.061	0.762	-0.053	-0.091	-0.046	-0.005	-0.05	-0.116	-0.065	-0.051	-0.036	0.015	-0.011	-0.001	-0.07	-0.029	-0.015	-0.019	0.017	0.089	-0.004	-0.027	-0.046	0
ADC wav HHH glcm Imc1	-0.534	0.153	-0.031	0.358	-0.247	0.122	-0.025	0.257	0.019	-0.049	0.071	-0.03	-0.045	-0.019	-0.023	-0.078	-0.013	-0.027	-0.145	0.009	0.089	0.016	0.065	0.082
ADC wav HHH glcm ClusterShade	0.798	0.008	0.023	-0.038	-0.101	-0.016	0.002	-0.01	-0.015	-0.017	0.001	-0.001	0.007	0.002	0.001	-0.002	0.004	-0.01	0.007	-0.006	-0.004	-0.006	0.005	-0.001
ADC wav HHH glcm SumEntropy	0.016	-0.503	0.042	-0.044	0.491	-0.009	0.091	0.107	0.131	0.01	-0.123	0.007	0.032	0.039	0.073	0.115	0.001	-0.061	0.231	-0.079	0.01	-0.043	-0.027	-0.001
ADC wav HHH glcm MaximumProbability	-0.023	0.763	-0.079	-0.055	-0.1	-0.008	-0.055	-0.097	-0.022	0.04	0.04	0.009	-0.009	-0.042	-0.037	-0.055	-0.004	-0.004	-0.076	0.09	-0.071	0.005	0.002	0.01
ADC wav HHH glcm Idm	-0.257	0.61	-0.095	0.129	-0.26	0.014	-0.071	-0.044	-0.041	0.062	0.112	-0.031	-0.029	-0.082	-0.077	-0.091	-0.017	0.004	-0.215	0.095	-0.03	0.013	0.025	0.066

ADC feature name	ADC F1	ADC F2	ADC F3	ADC F4	ADC F5	ADC F6	ADC F7	ADC F8	ADC F9	ADC F10	ADC F11	ADC F12	ADC F13	ADC F14	ADC F15	ADC F16	ADC F17	ADC F18	ADC F19	ADC F20	ADC F21	ADC F22	ADC F23	ADC F24
ADC wav HHH glszm LAHGLE	0.088	0.059	0.058	0.345	0.014	-0.084	0.081	0.083	-0.067	0.639	0.129	0.098	-0.033	0.022	-0.004	-0.015	0.064	0.005	-0.027	-0.003	-0.048	-0.057	0.001	-0.019
ADC wav HHH glszm SZNUN	0.472	-0.238	0.011	-0.269	0.412	-0.015	0.141	0.032	0.081	0.045	-0.048	0.059	0.06	0.064	0.066	0.127	0.038	-0.05	0.182	-0.078	-0.036	0.022	0.011	-0.031
ADC wav HHH glszm GLNU	-0.159	-0.119	0.042	0.333	-0.062	0.017	0.099	0.668	-0.041	0.015	0.023	0.039	-0.043	0.018	-0.018	-0.032	-0.002	0.051	-0.018	0.076	0.004	0.014	-0.032	-0.036
ADC wav HHH glszm LALGLE	-0.021	0.793	-0.041	-0.044	0.041	0.028	-0.001	0.024	0.007	-0.016	-0.041	0.013	0.009	-0.01	0.021	0.033	-0.001	-0.026	0.084	0.005	0.028	0.009	-0.002	-0.017
ADC wav HLL firstorder Maximum	0.409	-0.178	-0.03	-0.006	0.622	-0.063	0.099	0.037	0.054	0.028	0.029	-0.017	0.058	0.001	-0.025	0.068	-0.043	0.003	0.006	0.003	0.044	0.067	0.019	0.014
ADC wav HLL firstorder 10Percentile	-0.457	0.162	0.06	0.123	-0.576	0.081	-0.084	0.08	0.027	0.061	0.043	-0.045	-0.026	0.03	0.045	-0.096	-0.008	0.033	-0.074	-0.021	0.056	-0.021	0.002	0.047
ADC wav HLL firstorder Uniformity	0.055	0.76	0.031	-0.08	-0.162	0.03	-0.109	-0.046	-0.026	0.001	-0.014	-0.006	-0.017	0.012	-0.021	-0.048	-0.01	0.032	-0.051	-0.032	0.001	-0.058	-0.01	0.047
ADC wav HLL firstorder Median	0.778	-0.016	-0.002	-0.052	0.051	0.033	0.021	-0.075	-0.058	-0.017	0.025	-0.027	-0.066	-0.032	0.015	0.046	0.04	0.007	-0.021	-0.009	-0.027	-0.028	0.048	-0.004
ADC wav HLL firstorder Skewness	0.082	-0.018	0.014	0.002	-0.014	-0.206	-0.01	0.015	-0.086	-0.025	-0.011	-0.016	0.041	0.017	-0.035	-0.035	-0.035	-0.004	0.008	-0.022	0.602	0.05	-0.023	-0.006
ADC wav HLL firstorder Mean	0.778	0.008	0	0.012	-0.07	0.028	-0.014	-0.039	-0.015	-0.007	0.04	-0.025	-0.03	0.003	0.054	0.033	0.085	0.017	-0.046	0.025	0.084	0.002	0.021	0.01
ADC wav HLL firstorder TotalEnergy	0.076	-0.089	-0.051	0.113	0.761	0.068	0.05	0.117	-0.013	-0.002	0.032	0.003	-0.009	0.029	-0.015	-0.062	0.016	0.018	-0.085	0.033	0.001	0.003	-0.033	-0.017
ADC wav HLL gldm LDLGLE	0.261	0.735	0.001	-0.133	-0.03	-0.012	-0.022	-0.095	-0.042	-0.022	-0.021	0.033	0.014	0.01	0	0.014	-0.001	-0.013	0.059	0.037	0.015	-0.01	-0.033	-0.029
ADC wav HLL gldm LDHGLE	0.222	-0.093	-0.018	0.042	0.737	0.012	0.04	0.003	0.01	0.044	0.014	-0.031	0.01	-0.015	-0.016	-0.027	-0.009	0.014	-0.109	0.014	-0.098	0.057	0.035	0.022
ADC wav HLL ngtdm Busyness	0.004	0.794	0.001	-0.039	-0.01	0.023	-0.038	0.044	-0.009	-0.029	-0.043	0.021	-0.008	-0.021	0.015	0.013	0.004	-0.018	0.037	-0.01	0.062	-0.006	0.005	-0.014
ADC wav HLL ngtdm Strength	0.565	-0.105	-0.063	-0.129	0.49	-0.055	0.034	-0.166	-0.015	-0.038	-0.011	0.027	0.033	-0.013	-0.013	0.096	0.031	0.01	0.008	0.002	-0.058	0.036	-0.01	-0.012
ADC wav HLL ngtdm Complexity	0.146	-0.063	-0.054	-0.012	0.769	0.051	-0.018	-0.068	-0.022	-0.033	0.011	-0.014	0	0	-0.027	-0.057	-0.019	0.028	-0.112	-0.028	0.012	0.012	-0.009	-0.014
ADC wav HLL ngtdm Contrast	0.799	0.007	0.022	-0.039	-0.091	-0.017	0.001	-0.013	-0.017	-0.017	0.001	0	0.007	0.001	0.001	-0.001	0.004	-0.009	0.005	-0.007	-0.004	-0.004	0.004	-0.002
ADC wav HLL glcm InverseVariance	-0.187	0.728	-0.018	0.056	-0.164	0.059	-0.089	0.011	0.016	0.015	0.001	-0.016	-0.037	0.012	0.004	-0.042	-0.033	0.035	-0.081	-0.044	-0.026	-0.048	0.009	0.084
ADC wav HLL glcm ClusterShade	0.02	0.056	0.046	0.041	-0.771	-0.041	0.016	0.065	0.031	0.034	0.006	0.005	-0.003	0.039	0.046	0.062	0.065	-0.014	0.106	0.071	0.049	0.028	0.02	-0.003
ADC wav HLL glcm Correlation	-0.172	-0.034	-0.487	0.138	0.113	0.122	-0.038	0.014	0.145	-0.009	-0.05	0.097	0.086	0.032	0.116	-0.048	0.251	-0.033	0.013	0.031	-0.079	0.031	-0.243	0.21
ADC wav HLL glcm DifferenceEntropy	-0.177	-0.471	-0.052	0.174	0.478	0.012	0.182	0.148	0.11	-0.055	-0.001	-0.012	0.027	-0.024	0.04	0.109	0.02	-0.087	0.129	0.058	0.043	0.054	0.024	-0.012
ADC wav HLL glcm ClusterProminence	0.369	-0.034	-0.037	-0.025	0.667	0.061	-0.05	-0.089	-0.05	-0.026	0.017	-0.025	0.002	-0.002	-0.026	-0.095	-0.042	0.045	-0.146	-0.061	0.026	0.013	-0.012	-0.023
ADC wav HLL glcm Idmn	-0.464	-0.037	-0.052	0.301	0.064	0.08	-0.024	0.299	0.218	0.118	0.065	-0.085	0.092	0.088	-0.021	-0.001	-0.086	0.019	-0.108	-0.101	-0.009	0.007	-0.032	0.252
ADC wav HLL glszm ZoneVariance	-0.052	0.799	-0.012	0.021	0.006	0.027	0.005	0.024	-0.004	0.006	-0.031	0.005	0.006	-0.003	0.004	0.018	0.002	-0.011	0.057	-0.021	0.009	0.006	0.012	0.001
ADC wav LHH firstorder Maximum	0.775	-0.058	0.008	-0.006	0.127	-0.008	0.042	0.009	0.035	0.102	-0.036	-0.006	-0.013	0.016	0.007	0.03	0.006	-0.024	0.069	-0.001	0.014	-0.041	-0.018	0.02
ADC wav LHH firstorder Minimum	-0.682	0.082	-0.001	-0.012	-0.353	0.004	-0.046	-0.015	-0.048	-0.187	0.027	0.017	0.025	-0.02	-0.004	0.016	0.034	0.01	-0.038	0.01	-0.041	0.023	0.029	-0.018
ADC wav LHH firstorder Kurtosis	-0.022	-0.04	0.048	0.213	0.11	-0.004	0.167	0.125	0.175	0.647	0.111	-0.05	-0.071	0.055	0.011	0.097	-0.035	-0.01	0.047	0.002	0.062	0.012	-0.068	0.025
ADC wav LHH firstorder RootMeanSquared	0.804	-0.003	0.018	-0.042	-0.016	-0.015	0.002	-0.018	-0.014	-0.012	-0.004	-0.001	0.007	0	-0.002	-0.004	-0.001	-0.009	0.008	-0.009	-0.002	-0.01	0.001	-0.003
ADC wav LHH firstorder Median	-0.8	-0.007	-0.018	0.043	0.074	0.018	-0.004	0.014	0.014	0.017	-0.004	0.003	-0.005	0.005	-0.003	0.007	-0.006	0.001	0.002	0.011	0.003	0.008	-0.005	0.004

ADC feature name	ADC F1	ADC F2	ADC F3	ADC F4	ADC F5	ADC F6	ADC F7	ADC F8	ADC F9	ADC F10	ADC F11	ADC F12	ADC F13	ADC F14	ADC F15	ADC F16	ADC F17	ADC F18	ADC F19	ADC F20	ADC F21	ADC F22	ADC F23	ADC F24
ADC wav LHH firstorder Mean	-0.8	-0.012	-0.029	0.027	0.024	-0.004	0.012	0.014	0.014	0.026	-0.013	0.007	0.001	-0.015	-0.004	0.039	0.00E+00	-0.005	0.032	0.017	-0.009	0.004	-0.013	-0.006
ADC wav LHH gldm LDLGLE	-0.022	0.796	-0.043	-0.045	0.032	0.03	-0.009	0.009	0.008	-0.02	-0.04	0.018	0.01	-0.01	0.015	0.026	-0.001	-0.023	0.072	0.011	0.022	0	-0.003	-0.01
ADC wav LHH gldm DependenceEntropy	-0.121	-0.337	0.047	0.36	0.469	0.012	0.17	0.247	0.119	0.116	-0.031	0.008	-0.017	-0.061	0.032	0.067	0.014	-0.047	0.142	0.022	0	-0.001	0.031	0.007
ADC wav LHH gldm LDHGLE	0.659	-0.03	0.008	0.097	0.229	0.005	0.058	0.005	-0.001	0.344	-0.025	-0.018	-0.057	0.05	0.027	-0.01	0.017	-0.012	-0.005	-0.018	0.038	-0.061	-0.05	0.04
ADC wav LHH gldm DependenceVariance	-0.12	0.702	0.066	0.211	-0.075	0.001	0.11	0.017	0.075	0.059	0.152	0.016	-0.01	-0.024	-0.006	-0.002	0.039	0.003	-0.138	0.04	-0.084	0.053	0.055	0.017
ADC wav LHH gldm SDLGLE	0.407	0.151	-0.027	-0.231	-0.22	0.062	-0.223	-0.352	-0.058	-0.061	0.003	0.104	0.089	0.073	-0.029	-0.057	0.009	0.047	-0.12	0.08	-0.161	-0.168	-0.031	0.057
ADC wav LHH ngtdm Strength	0.803	-0.008	0.02	-0.051	-0.004	-0.02	0.006	-0.03	-0.008	0.003	-0.003	-0.001	0.003	0.001	-0.002	0.004	0	-0.006	0.015	-0.008	-0.001	-0.007	0	-0.004
ADC wav LHH ngtdm Coarseness	-0.13	-0.025	0.046	-0.382	-0.142	-0.104	-0.163	-0.541	-0.136	-0.021	-0.034	0.006	0.06	0.139	-0.044	-0.086	-0.012	0.051	-0.054	0.155	-0.112	-0.087	-0.154	-0.027
ADC wav LHH ngtdm Contrast	0.798	0.008	0.022	-0.037	-0.095	-0.015	0.001	-0.011	-0.016	-0.017	0.001	-0.001	0.007	0.002	0.001	-0.003	0.004	-0.009	0.005	-0.007	-0.003	-0.005	0.005	-0.001
ADC wav LHH glcm ClusterShade	0.798	0.008	0.022	-0.037	-0.098	-0.016	0.001	-0.011	-0.016	-0.017	0.001	-0.001	0.007	0.002	0.001	-0.002	0.005	-0.01	0.005	-0.006	-0.003	-0.005	0.005	-0.001
ADC wav LHH glcm SumEntropy	-0.038	-0.542	-0.024	0.049	0.493	-0.013	0.074	0.116	0.055	0.035	-0.101	0.005	0.035	-0.021	0.032	0.062	-0.017	-0.071	0.224	-0.01	0.042	-0.049	-0.018	0.019
ADC wav LHH glcm MaximumProbability	0.007	0.796	-0.032	-0.075	-0.049	0.012	-0.013	-0.051	0.018	-0.004	0.016	0.011	-0.001	0	0.009	0.003	0.006	0.001	-0.019	0.025	-0.023	0.016	0.007	-0.001
ADC wav LHH glcm Idm	-0.238	0.69	-0.03	0.08	-0.21	0.041	-0.03	-0.009	0.046	0.011	0.093	-0.012	-0.033	-0.009	-0.038	-0.032	0.004	0.04	-0.191	0.024	-0.057	0.028	0.027	0.046
ADC wav LHH glszm LAHGLE	0.273	0.091	0.138	0.228	0.094	-0.028	0.179	0.045	-0.019	0.41	0.438	0.024	-0.063	0.107	-0.039	0.051	0.063	-0.061	-0.052	-0.051	-0.016	-0.099	-0.077	-0.097
ADC wav LHH glszm LALGLE	-0.021	0.793	-0.041	-0.045	0.041	0.028	-0.001	0.024	0.007	-0.016	-0.041	0.012	0.009	-0.01	0.021	0.033	-0.001	-0.026	0.084	0.004	0.028	0.009	-0.002	-0.017
ADC wav HHL firstorder Maximum	0.643	-0.154	-0.007	-0.089	0.296	-0.078	0.076	0.095	0.136	0.054	-0.076	0.022	0.066	-0.021	0.042	0.056	0.114	0.038	0.081	0.085	0.036	0.06	-0.073	-0.092
ADC wav HHL firstorder Kurtosis	-0.136	-0.129	-0.014	0.005	-0.047	-0.016	0.185	0.17	0.271	0.408	-0.115	-0.14	0.057	0.078	0.062	0.101	-0.039	0.288	0.023	0.112	0.114	0.081	-0.156	-0.086
ADC wav HHL firstorder Median	0.449	0.013	-0.078	-0.013	-0.323	-0.159	0.057	0.004	-0.033	-0.03	-0.001	0.15	0.081	-0.154	-0.073	0.348	0.009	-0.07	-0.002	-0.032	-0.07	-0.153	0.017	0.052
ADC wav HHL firstorder Skewness	0.009	0.041	-0.242	-0.122	-0.094	0.072	-0.051	0.037	0.013	-0.032	-0.06	0.093	-0.109	-0.012	0.161	-0.066	0.584	0.154	-0.005	0.00E+00	-0.084	0.041	-0.014	-0.071
ADC wav HHL firstorder Mean	-0.795	-0.005	-0.026	0.034	-0.033	-0.012	-0.004	0.028	0.043	0.012	-0.002	0.028	-0.007	-0.012	0.007	0.057	0.069	-0.01	0.025	0.02	-0.02	-0.019	-0.011	0.015
ADC wav HHL firstorder TotalEnergy	0.197	-0.099	0.029	0.191	0.628	0.012	0.078	0.354	0.033	0.079	0.02	0.022	0.005	0.009	-0.017	-0.087	-0.003	0.001	-0.008	0.058	-0.016	-0.044	-0.062	-0.036
ADC wav HHL ngtdm Busyness	-0.026	0.796	-0.05	-0.026	0.013	0.022	-0.029	0.03	-0.003	-0.024	-0.034	0.01	-0.002	-0.017	-0.01	0.017	0.009	-0.033	0.055	-0.009	0.018	0.006	0	-0.024
ADC wav HHL ngtdm Strength	0.785	-0.038	0.025	-0.101	0.092	-0.046	0.022	-0.06	0.014	0.001	-0.019	0.012	0.03	-0.014	-0.001	0.031	0.01	0.013	0.029	0.002	0.004	0.006	-0.02	-0.029
ADC wav HHL glcm InverseVariance	-0.17	0.731	-0.121	0.066	-0.155	0.044	-0.094	-0.006	-0.026	0.025	0.01	-0.042	-0.041	-0.035	-0.093	-0.013	-0.023	0.006	-0.06	-0.014	0.006	-0.01	0.02	0.042
ADC wav HHL glcm ClusterShade	0.598	0.022	0.019	0	-0.474	-0.033	0.02	0.025	0.029	-0.015	-0.026	0.006	0	0.041	0.05	0.046	0.168	-0.058	0.082	0.071	-0.018	0.011	0.012	-0.011
ADC wav HHL glcm Correlation	-0.245	0.099	-0.466	-0.013	0.064	0.177	-0.048	0.117	-0.012	0.087	0.122	0.126	0.12	-0.031	-0.209	-0.048	-0.038	-0.014	-0.026	-0.035	0.057	-0.043	-0.045	0.052
ADC wav HHL glcm SumEntropy	-0.205	-0.503	0.042	0.153	0.429	0.019	0.142	0.224	0.159	-0.021	-0.033	0.005	0.063	-0.013	0.072	0.056	0.01	-0.079	0.118	0.025	0.041	0.022	0	0.022
ADC wav HHL glcm ClusterProminence	0.801	0.006	0.02	-0.039	-0.066	-0.014	0	-0.015	-0.016	-0.017	0.001	-0.001	0.008	0.001	-0.001	-0.005	0.002	-0.007	0	-0.009	-0.001	-0.004	0.003	-0.003
ADC wav HHL glcm Idmn	-0.473	-0.084	-0.051	0.171	0.041	0.049	0.092	0.348	0.239	0.215	0.008	-0.087	0.048	0.082	0.094	0.09	-0.057	0.175	-0.057	0.025	0.08	0.123	-0.032	-0.022
ADC wav HHL glszm LAHGLE	0.697	-0.056	0.041	-0.035	0.319	-0.012	0.048	0.027	0.091	0.105	-0.015	-0.033	0.035	-0.01	-0.034	-0.011	-0.111	0.041	0.03	0.003	0.049	-0.009	-0.041	-0.022
ADC wav HHL glszm ZoneVariance	-0.024	0.794	-0.042	-0.036	0.037	0.028	-0.001	0.022	0.005	-0.014	-0.04	0.012	0.006	-0.011	0.018	0.03	-0.001	-0.026	0.082	0.004	0.027	0.007	0	-0.016

ADC feature name	ADC F1	ADC F2	ADC F3	ADC F4	ADC F5	ADC F6	ADC F7	ADC F8	ADC F9	ADC F10	ADC F11	ADC F12	ADC F13	ADC F14	ADC F15	ADC F16	ADC F17	ADC F18	ADC F19	ADC F20	ADC F21	ADC F22	ADC F23	ADC F24
ADC wav HHL glszm SALGLE	0.398	0.596	-0.064	-0.173	-0.103	-0.03	-0.11	-0.186	-0.074	-0.03	-0.027	-0.009	0.018	0.028	-0.086	-0.03	0.013	-0.045	0.021	0.058	-0.053	-0.034	-0.094	-0.035
ADC wav HLH firstorder Maximum	0.574	-0.147	0	0.025	0.47	-0.014	0.069	0.013	0.007	0.203	-0.036	-0.01	0.008	-0.004	0.091	0.016	-0.009	-0.015	0.04	-0.053	-0.018	0.006	0.039	0.018
ADC wav HLH firstorder Kurtosis	-0.137	0.008	0.003	0.209	-0.081	-0.115	-0.006	0.101	0.072	0.551	0.063	-0.062	0.073	-0.107	0.124	-0.119	-0.105	-0.043	-0.085	-0.044	-0.227	0.148	0.161	0.037
ADC wav HLH firstorder RootMeanSquared	0.79	-0.016	0.014	-0.042	0.133	-0.006	0	-0.041	-0.024	-0.012	-0.009	-0.006	0.019	0.001	-0.012	-0.01	-0.018	0.005	-0.011	-0.028	0.004	-0.007	-0.005	-0.008
ADC wav HLH firstorder Median	0.668	0.038	0.024	0.098	-0.335	-0.046	0.028	0.041	0.086	-0.014	-0.053	-0.059	0.1	0.006	-0.055	0.136	0.019	-0.008	0.045	0.037	0.032	0.008	-0.009	-0.015
ADC wav HLH firstorder Skewness	-0.086	0.054	0.184	0.031	0.01	0.236	0.076	-0.07	-0.068	0.192	-0.066	-0.027	0.056	0.072	0.511	-0.032	0.058	0.059	0.018	-0.077	-0.096	-0.047	0.016	0.048
ADC wav HLH firstorder Range	0.608	-0.15	-0.028	0.002	0.44	-0.046	0.067	0.036	0.022	0.188	-0.039	0.017	0.005	-0.014	0.026	0.016	-0.047	-0.015	0.066	-0.052	-0.02	0.004	0.026	0.017
ADC wav HLH firstorder Mean	0.004	0.099	0.195	0.215	-0.477	0.07	0.054	-0.001	0.009	0.111	-0.118	-0.119	0.265	0.091	-0.08	0.075	-0.095	0.079	-0.018	0.005	0.188	0.087	-0.102	-0.13
ADC wav HLH gldm LDLGLE	-0.018	0.796	-0.036	-0.047	0.029	0.025	-0.008	0.004	0.002	-0.022	-0.04	0.017	0.008	-0.012	0.015	0.024	-0.004	-0.025	0.065	0.022	0.031	-0.001	-0.008	-0.006
ADC wav HLH gldm DependenceEntropy	-0.141	-0.35	0.017	0.354	0.485	-0.021	0.199	0.198	0.091	0.079	-0.012	0.011	-0.032	0.02	0.048	0.099	-0.024	-0.02	0.135	-0.038	-0.017	-0.015	0.039	0.042
ADC wav HLH gldm LDHGLE	0.504	-0.067	-0.003	0.198	0.245	-0.086	0.078	0.097	0.019	0.488	-0.046	0.046	-0.029	-0.001	-0.01	-0.036	-0.049	-0.008	0.034	-0.034	-0.034	-0.046	0.009	0.06
ADC wav HLH gldm DependenceVariance	-0.132	0.671	0.136	0.223	-0.088	-0.037	0.103	0.032	0.026	0.127	0.126	-0.039	0.009	-0.023	0.032	-0.033	-0.008	-0.05	-0.143	0.024	-0.087	0.072	0.035	0.039
ADC wav HLH gldm SDLGLE	0.526	0.163	0.132	-0.253	-0.182	-0.028	-0.089	-0.316	-0.096	-0.079	-0.029	0.088	0.065	0.042	0.031	-0.049	0.008	0.032	-0.11	0.132	0.05	-0.112	-0.09	0.067
ADC wav HLH ngtdm Strength	0.796	-0.021	0.01	-0.073	0.054	-0.031	0.001	-0.059	-0.026	0.004	-0.004	0.001	0.012	-0.001	0.004	0.001	-0.013	0.005	0.016	-0.028	-0.027	-0.009	0.001	-0.003
ADC wav HLH ngtdm Complexity	0.593	-0.034	-0.018	-0.029	0.51	0.043	-0.026	-0.061	-0.035	-0.007	-0.001	-0.022	0.005	0.004	-0.023	-0.087	-0.049	0.023	-0.088	-0.059	0.017	-0.015	-0.009	-0.015
ADC wav HLH ngtdm Contrast	0.799	0.007	0.022	-0.038	-0.093	-0.015	0.001	-0.012	-0.016	-0.017	0.001	-0.001	0.007	0.002	0.001	-0.003	0.004	-0.009	0.005	-0.007	-0.003	-0.005	0.004	-0.002
ADC wav HLH glrlm SRLGLE	0.248	0.648	0.031	-0.165	-0.108	0.002	-0.087	-0.211	-0.066	-0.072	-0.039	0.061	0.037	0.005	-0.009	-0.031	-0.002	-0.003	-0.055	0.126	0.048	-0.088	-0.081	0.053
ADC wav HLH glcm Imc1	-0.462	0.149	0.051	0.399	-0.239	0.116	-0.007	0.344	0.11	-0.005	0.043	-0.02	-0.059	-0.038	0.016	-0.08	-0.015	-0.022	-0.115	0.009	0.054	0.01	0.057	0.064
ADC wav HLH glcm JointEntropy	-0.225	-0.523	-0.026	0.245	0.306	0.075	0.11	0.279	0.099	-0.033	-0.063	0.015	-0.026	0.038	0.027	0.093	0.001	-0.045	0.166	-0.031	0.08	-0.026	0.014	0.043
ADC wav HLH glcm ClusterShade	-0.782	-0.01	-0.026	0.041	0.175	0.034	-0.008	0.001	0.007	0.026	-0.002	-0.005	-0.009	0.003	0	-0.02	-0.012	0.018	-0.029	-0.002	0.01	0.006	-0.008	-0.006
ADC wav HLH glcm Correlation	-0.151	-0.023	-0.301	0.269	0.113	0.194	-0.132	0.133	0.022	0.015	-0.012	0.25	-0.019	-0.175	-0.135	0.073	-0.012	-0.15	0.026	0.108	-0.05	-0.131	-0.065	0.198
ADC wav HLH glcm SumEntropy	-0.067	-0.527	-0.07	0.05	0.51	0.03	0.102	0.077	0.057	-0.019	-0.078	0.031	0.022	0.03	0.018	0.127	-0.001	-0.04	0.204	-0.048	0.035	-0.025	-0.009	0.028
ADC wav HLH glcm Idm	-0.246	0.669	-0.009	0.099	-0.222	-0.002	-0.046	0.022	0.037	0.074	0.083	-0.043	-0.012	-0.036	0.042	-0.099	-0.025	0.004	-0.184	0.036	-0.064	0.026	0.035	0.053
ADC wav HLH glszm LAHGLE	0.18	0.081	0.086	0.323	0.079	-0.068	0.168	0.075	-0.045	0.587	0.23	0.065	-0.057	0.051	-0.033	-0.004	0.041	-0.027	-0.04	-0.05	-0.045	-0.094	-0.027	-0.026
ADC wav HLH glszm GrayLevelVariance	0.801	-0.008	0.013	-0.042	0.058	-0.009	-0.002	-0.029	-0.021	-0.013	-0.001	-0.003	0.007	0.001	-0.004	-0.014	-0.008	-0.002	-0.008	-0.021	-0.004	-0.009	0.002	-0.002
ADC wav HLH glszm SZNUN	0.405	-0.347	-0.02	-0.323	0.352	-0.033	0.107	-0.065	-0.055	-0.019	-0.096	0.087	0.027	0.1	-0.034	0.166	0.014	-0.055	0.158	-0.043	0.062	0.032	-0.068	-0.041
ADC wav HLH glszm ZoneEntropy	-0.08	-0.408	-0.012	0.338	0.472	-0.036	0.152	0.192	0.086	0.093	0.016	-0.012	-0.005	-0.02	0.056	0.057	-0.03	0.025	0.139	-0.074	-0.065	-0.015	0.067	0.023
ADC wav HLH glszm LALGLE	-0.021	0.793	-0.041	-0.044	0.041	0.027	-0.001	0.023	0.007	-0.016	-0.041	0.013	0.008	-0.01	0.021	0.032	-0.001	-0.026	0.083	0.005	0.029	0.009	-0.002	-0.017
ADC wav LHL firstorder Maximum	0.73	-0.092	0.031	-0.024	0.243	-0.031	0.065	0.043	0.093	0.029	0.04	0.008	0.052	-0.03	0.023	0.042	-0.014	0.001	0.018	0.04	0.024	0.06	-0.055	-0.032

ADC feature name	ADC F1	ADC F2	ADC F3	ADC F4	ADC F5	ADC F6	ADC F7	ADC F8	ADC F9	ADC F10	ADC F11	ADC F12	ADC F13	ADC F14	ADC F15	ADC F16	ADC F17	ADC F18	ADC F19	ADC F20	ADC F21	ADC F22	ADC F23	ADC F24
ADC wav LHL firstorder RootMeanSquared	0.795	-0.028	0	-0.051	0.11	-0.021	0.016	-0.018	-0.009	-0.021	0.005	0.004	0.017	-0.011	0.004	0.002	0.006	-0.001	0.003	0.002	0.002	-0.001	-0.009	-0.007
ADC wav LHL firstorder Median	0.798	0.006	0.004	-0.046	-0.075	-0.02	0.015	-0.005	-0.033	-0.023	0.013	-0.013	0.003	0.006	0.001	0.005	-0.01	0.002	-0.012	-0.024	-0.014	-0.014	0.02	0.02
ADC wav LHL firstorder Skewness	-0.159	-0.011	0.152	0.056	-0.037	-0.199	0.048	-0.123	0.207	0.082	0.16	0.191	0.114	0.03	0.099	0.1	-0.205	0.028	0.024	0.011	0.148	0.13	-0.364	-0.044
ADC wav LHL firstorder Mean	0.797	0.01	0.003	-0.041	-0.087	-0.027	0.01	-0.009	-0.026	-0.026	0.02	-0.002	0.011	0.003	0.001	0.011	-0.014	0.008	-0.018	-0.026	-0.004	-0.008	0.004	0.02
ADC wav LHL gldm LDLGLE	0.1	0.78	-0.055	-0.078	0	0.015	-0.02	-0.039	-0.016	-0.034	-0.052	0.032	0.014	0.02	0.027	0.036	-0.016	-0.031	0.058	-0.012	0.027	0.017	-0.018	-0.026
ADC wav LHL gldm LDHGLE	0.8	-0.012	0.017	-0.027	0.076	0	0.007	-0.006	-0.007	-0.012	0.006	-0.017	0.006	0.003	0.005	-0.012	0.017	-0.012	-0.022	0.005	-0.007	0.009	0.005	0.002
ADC wav LHL gldm DependenceVariance	-0.112	0.782	0.004	0.1	-0.063	0.006	0.012	0.019	0.004	-0.006	-0.023	0.006	-0.044	-0.018	-0.01	-0.008	-0.008	0.022	-0.001	-0.035	0.009	0.036	0.023	0.03
ADC wav LHL ngtdm Busyness	-0.009	0.793	-0.018	-0.03	-0.018	0.019	-0.046	0.017	-0.022	-0.034	-0.045	0.035	-0.026	0.01	0.007	0.013	-0.007	-0.018	0.043	-0.039	0.049	0.004	-0.004	0.003
ADC wav LHL ngtdm Strength	0.787	-0.034	0.005	-0.08	0.123	-0.03	0.02	-0.055	-0.011	-0.017	0.005	0.008	0.017	-0.028	-0.004	0.018	0.007	0.001	0.01	0.00E+00	-0.002	0.008	-0.016	-0.015
ADC wav LHL ngtdm Contrast	0.798	0.008	0.022	-0.037	-0.095	-0.015	0.001	-0.011	-0.016	-0.017	0.001	-0.001	0.007	0.002	0.001	-0.003	0.005	-0.009	0.005	-0.007	-0.003	-0.005	0.005	-0.001
ADC wav LHL glcm DifferenceVariance	0.803	0.001	0.019	-0.04	-0.036	-0.014	0.001	-0.016	-0.016	-0.018	0.001	-0.001	0.008	-0.001	0.00E+00	-0.005	0.004	-0.007	0	-0.007	-0.002	-0.003	0.002	-0.003
ADC wav LHL glcm ClusterShade	-0.8	-0.007	-0.021	0.037	0.084	0.013	0	0.012	0.017	0.018	-0.001	0.002	-0.006	-0.003	-0.001	0.005	-0.004	0.01	-0.002	0.007	0.003	0.005	-0.005	0.002
ADC wav LHL glcm Correlation	-0.308	0.08	-0.599	0.136	-0.061	0.015	0.034	0.023	0.032	0.034	0.137	0.003	-0.044	-0.108	-0.061	-0.009	-0.058	-0.013	-0.031	-0.085	0.084	-0.184	-0.05	0.102
ADC wav LHL glcm SumEntropy	-0.265	-0.421	-0.068	0.28	0.386	0.024	0.151	0.27	0.14	0.03	-0.03	-0.011	0.048	-0.066	0.08	0.027	-0.002	-0.092	0.092	0.078	0.044	-0.004	0.014	0.017
ADC wav LHL glcm Idm	-0.164	0.754	-0.062	-0.009	-0.139	0.047	-0.082	-0.004	-0.003	-0.009	-0.001	-0.028	-0.06	0.028	-0.053	-0.013	-0.007	0.04	-0.045	-0.065	-0.01	0.013	0.013	0.036
ADC wav LHL glcm Idmn	-0.287	-0.017	-0.047	0.278	0.081	0.145	0.084	0.403	0.29	0.099	0.08	-0.087	0.033	0.01	0.006	0.156	-0.051	0.017	-0.127	-0.042	0.008	0.077	-0.069	0.048
ADC wav LHL glszm SALGLE	0.566	0.377	0.044	-0.225	-0.138	-0.074	-0.093	-0.251	-0.092	-0.031	-0.035	0.004	0.021	0.062	-0.035	-0.044	-0.008	0.008	-0.01	0.058	-0.01	-0.024	-0.095	0.026
ADC wav LLL firstorder Maximum	0.716	-0.098	-0.041	0.03	0.335	-0.019	0.007	0.005	-0.012	-0.032	0.052	0.013	-0.005	0.009	0.01	-0.022	0.015	0.004	-0.001	0.048	0.023	0.019	-0.004	-0.027
ADC wav LLL firstorder Minimum	0.784	-0.024	-0.01	-0.04	0.066	0.031	-0.037	-0.039	-0.048	-0.069	0.016	-0.014	0.015	0.011	0.009	-0.064	-0.019	0.004	-0.058	-0.008	0.014	0	-0.005	-0.055
ADC wav LLL firstorder Kurtosis	-0.063	0.004	0.19	0.186	-0.026	-0.022	0.138	0.147	0.035	0.118	0.677	-0.036	0.036	0.019	-0.018	-0.027	-0.057	0.015	-0.026	0.029	0	0.087	-0.016	0.031
ADC wav LLL firstorder Skewness	0.049	0.06	-0.1	0.218	0.072	-0.088	0.335	0.077	-0.13	-0.014	0.412	-0.068	-0.109	0.057	0.184	0.097	-0.008	-0.054	-0.041	0.182	-0.166	-0.072	-0.02	-0.099
ADC wav LLL gldm LDHGLE	0.459	-0.096	-0.022	0.065	0.612	-0.045	-0.069	0.002	0.062	0.112	-0.031	0.055	-0.011	0.009	-0.017	-0.021	0.051	0.043	0.031	0.042	0.063	0.015	-0.004	0.03
ADC wav LLL ngtdm Busyness	0.087	0.79	-0.029	-0.068	-0.012	0.047	0.017	0.024	-0.019	-0.032	-0.025	-0.007	0.032	-0.013	0.037	0.029	0.005	-0.003	0.045	-0.005	0.004	-0.015	0.004	-0.021
ADC wav LLL ngtdm Strength	0.395	-0.132	-0.053	-0.005	0.57	-0.145	0.012	-0.139	0.057	-0.043	0.074	0.048	-0.126	0.005	-0.005	0.146	0.166	-0.03	0.084	0.108	-0.015	-0.006	0.011	0.022
ADC wav LLL ngtdm Contrast	0.799	0.008	0.022	-0.037	-0.094	-0.016	0.001	-0.011	-0.016	-0.017	0.001	-0.001	0.007	0.002	0.001	-0.002	0.005	-0.009	0.005	-0.007	-0.003	-0.005	0.004	-0.001
ADC wav LLL glcm Imc2	0.022	-0.795	0.03	0.042	-0.038	-0.028	0.001	-0.035	-0.006	0.012	0.029	-0.01	-0.012	0.01	-0.019	-0.033	-0.002	0.025	-0.077	-0.001	-0.025	-0.006	0.003	0.022
ADC wav LLL glcm InverseVariance	-0.144	0.732	0.167	-0.055	-0.134	0.101	-0.038	0.017	-0.016	0.02	0.037	-0.038	-0.006	0.043	0.011	-0.009	-0.007	0.047	-0.071	-0.068	-0.019	-0.047	0.009	0.036
ADC wav LLL glcm Correlation	-0.529	0.044	0.088	0.207	0.028	-0.072	-0.043	0.138	0.081	0.02	0.028	0.004	-0.475	-0.034	-0.058	-0.038	0.06	0.01	-0.008	-0.044	-0.061	-0.04	0.041	0.024
ADC wav LLL glcm Idn	-0.407	-0.013	0.249	0.336	0.012	0.018	0.058	0.33	0.062	0.142	0.225	-0.063	-0.128	0.012	0.029	-0.061	-0.109	0.11	0.005	-0.025	0.008	0.087	0.062	0.168
ADC wav LLL glszm ZoneVariance	-0.088	0.784	0.077	0.041	-0.032	0.049	0.031	0.029	-0.008	-0.001	0.045	0.013	-0.005	0.039	-0.001	0.012	0.018	0.018	0.014	-0.055	0.005	-0.03	0.007	-0.014
ADC lbp 2D firstorder Kurtosis	-0.116	0.001	-0.704	-0.007	-0.001	-0.23	-0.009	-0.005	-0.029	0.013	0.057	-0.1	0.159	0.028	-0.011	-0.052	0.084	0.117	0.01	-0.067	0.015	-0.051	0.086	0.054

ADC feature name	ADC F1	ADC F2	ADC F3	ADC F4	ADC F5	ADC F6	ADC F7	ADC F8	ADC F9	ADC F10	ADC F11	ADC F12	ADC F13	ADC F14	ADC F15	ADC F16	ADC F17	ADC F18	ADC F19	ADC F20	ADC F21	ADC F22	ADC F23	ADC F24
ADC lbp 2D firstorder Mean	0.052	0.097	0.155	-0.047	-0.104	0.704	0.04	0.055	-0.041	-0.053	0.106	-0.177	0.121	0.051	0.067	-0.047	0.009	0.023	-0.011	-0.102	0.017	-0.014	-0.021	0.057
ADC lbp 2D firstorder Skewness	-0.073	-0.082	-0.547	0.05	0.079	-0.539	0.038	0.016	-0.037	0.002	-0.041	0.097	0.005	0.029	0.021	0.044	-0.009	-0.026	-0.024	0.037	0.087	-0.001	0.052	-0.01
ADC lbp 2D glcm ClusterShade	0.084	-0.104	-0.377	-0.003	0.1	-0.445	0.091	0.078	-0.036	-0.006	-0.062	0.277	-0.25	0.045	0.003	0.024	0.094	-0.053	0.055	0.015	0.096	0.139	0.092	-0.141
ADC lbp 2D glcm ClusterTendency	0.036	0.065	0.758	-0.074	-0.005	0.073	-0.03	0.019	-0.046	0.045	0.04	-0.005	-0.093	0.054	0.03	-0.038	-0.074	-0.082	0.018	-0.021	0.057	0.035	-0.054	0.072
ADC lbp 2D glcm Correlation	-0.218	0.138	-0.621	0.077	0.025	0.15	0.01	0.063	-0.009	0.005	0.027	-0.034	-0.177	0.034	0.186	-0.076	-0.072	-0.054	0.08	-0.02	0.017	0.027	0.062	0.13
ADC lbp 2D glcm DifferenceEntropy	-0.025	-0.008	0.748	0.088	0.043	0.149	-0.022	0.103	0.095	0.028	-0.033	0.057	-0.071	-0.047	-0.059	0.031	-0.019	-0.064	-0.023	0.001	0.094	0.032	-0.004	0.004
ADC lbp 2D glcm DifferenceVariance	0.155	-0.039	0.746	-0.034	-0.031	-0.02	-0.069	0.002	-0.001	0.033	0.039	0.014	0.038	0.159	-0.047	-0.029	-0.018	-0.037	-0.028	-0.047	0.073	0.021	-0.084	0.018
ADC lbp 2D glcm JointEnergy	0.268	-0.046	-0.426	-0.285	-0.136	-0.284	0.012	-0.183	-0.207	0.008	0.095	-0.104	0.18	0.242	0.034	-0.085	0.048	0.081	-0.007	0.015	-0.017	-0.033	0.033	-0.043
ADC lbp 2D glcm Idm	-0.093	-0.03	-0.75	0.092	-0.026	-0.068	-0.037	0.006	0.026	-0.019	0.034	-0.045	0.064	0.183	0.043	-0.043	0.01	0.022	-0.004	-0.054	0.055	-0.008	0.033	0.036
ADC lbp 2D glcm InverseVariance	0.034	0.006	-0.718	0.105	-0.028	-0.035	-0.01	0.041	-0.046	-0.053	0.066	-0.138	0.1	0.052	0.044	-0.081	0.045	0.044	0.052	-0.082	-0.037	-0.006	0.026	0.075
ADC lbp 2D glcm MaximumProbability	0.144	-0.104	-0.509	-0.217	-0.092	-0.135	-0.014	-0.169	-0.175	0.037	0.066	0.024	0.163	0.398	0.036	0.052	0.039	0.038	0.002	0.052	-0.052	0.021	0.044	-0.068
ADC lbp 2D glcm SumEntropy	-0.325	0.066	0.522	0.257	0.121	0.212	-0.04	0.193	0.161	0.026	-0.031	0.032	-0.109	-0.115	0.005	0.068	-0.083	-0.109	-0.009	-0.047	0.066	0.032	-0.031	0.109
ADC lbp 2D glrlm GLNUN	0.006	-0.019	-0.551	-0.143	-0.08	-0.225	0.006	-0.037	-0.167	-0.002	0.115	-0.136	0.322	0.271	0.06	-0.051	0.015	0.024	-0.055	-0.048	0.064	-0.04	-0.015	0.095
ADC lbp 2D glrlm HGLRE	0.07	0.085	0.397	-0.085	-0.115	0.618	0.025	0.033	-0.053	-0.034	0.095	-0.157	0.085	0.056	0.068	-0.037	0.002	0.033	-0.008	-0.061	0.014	-0.006	-0.041	0.058
ADC lbp 2D glrlm LRHGLE	0.093	0.032	0.355	0.202	-0.103	0.547	0.023	0.044	-0.035	-0.006	0.13	-0.158	0.106	0.212	0.041	-0.055	-0.034	0.007	-0.006	-0.145	0.06	-0.012	-0.052	0.091
ADC lbp 2D glrlm LRLGLE	0.044	-0.019	0.68	-0.028	-0.019	-0.344	-0.06	-0.078	-0.018	0.084	-0.026	0.029	-0.04	0.145	-0.038	-0.043	-0.036	0.019	0.002	0.057	-0.041	0.041	-0.068	0.02
ADC lbp 2D glrlm RunEntropy	-0.178	-0.026	0.295	0.534	0.11	0.161	0.006	0.143	0.221	0.044	-0.089	0.119	-0.282	-0.134	-0.023	0.011	-0.023	-0.015	0.018	0.007	0.049	0.019	0.049	-0.043
ADC lbp 2D glszm GLNUN	0.006	0.01	-0.501	-0.217	-0.089	-0.219	0.048	-0.034	-0.187	-0.036	0.13	-0.17	0.332	0.215	0.104	-0.09	0.006	0.059	-0.039	-0.051	0.076	-0.045	-0.006	0.098
ADC lbp 2D glszm GrayLevelVariance	0.112	-0.04	0.768	-0.071	-0.068	-0.031	-0.062	-0.026	-0.015	0.056	-0.001	-0.02	-0.077	0.071	-0.023	-0.027	-0.023	-0.009	-0.006	0.059	-0.007	0.016	-0.028	-0.011
ADC lbp 2D glszm HGLZE	-0.064	0.118	0.207	-0.068	-0.107	0.713	0.044	0.032	-0.038	-0.038	0.038	-0.127	0.037	-0.003	0.122	-0.01	0.037	0.096	0.011	0.021	-0.009	0.022	-0.032	0.035
ADC lbp 2D glszm LAHGLE	0.015	-0.041	0.142	0.528	-0.041	0.21	0.064	0.021	-0.072	0.132	0.196	-0.059	0.066	0.406	-0.021	-0.015	-0.053	-0.022	-0.012	-0.181	0.063	-0.013	-0.085	0.045
ADC lbp 2D glszm SZNUN	0.316	0.132	0.308	-0.548	-0.044	0.082	0.059	-0.09	-0.104	-0.057	-0.02	0.044	-0.074	-0.036	0.019	0.062	0.031	0.026	0.079	0.086	-0.154	0.106	0.007	-0.078
ADC lbp 2D glszm SAHGLE	-0.02	0.189	0.143	-0.328	-0.09	0.595	0.081	-0.03	-0.061	-0.056	-0.004	-0.057	-0.048	-0.068	0.136	0.023	0.069	0.138	0.057	0.101	-0.122	0.102	-0.034	-0.025
ADC lbp 2D glszm ZoneVariance	-0.073	-0.063	-0.311	0.534	0.006	0.026	0.057	0.036	-0.046	0.15	0.01	0.111	-0.015	0.408	0.035	0.128	-0.044	0.015	0.019	0.012	0.049	0.035	0.039	-0.029
ADC lbp 2D gldm DependenceEntropy	-0.363	-0.011	0.04	0.604	0.114	0.116	0.032	0.157	0.22	0.002	-0.039	0.067	-0.156	-0.015	0.032	-0.013	-0.051	-0.023	0.003	-0.052	0.087	0.012	0.023	0.027
ADC lbp 2D gldm DependenceVariance	-0.035	-0.025	-0.351	0.578	0.038	0.054	0.029	0.018	0.081	-0.001	-0.017	0.104	-0.073	0.314	0.096	0.054	-0.005	0.041	0.031	0.047	0.076	0.076	0.064	-0.003
ADC lbp 2D gldm LDLGLE	-0.136	0.022	0.505	0.247	-0.001	-0.384	-0.065	-0.091	0.004	0.097	-0.045	0.044	0.002	0.281	0.03	-0.048	-0.059	0.057	-0.002	0.036	-0.01	0.04	-0.149	0.102
ADC lbp 2D gldm SDHGLE	0.083	0.159	0.295	-0.485	-0.087	0.45	0.056	-0.031	-0.084	-0.056	0.011	-0.062	-0.02	-0.118	0.085	0.008	0.057	0.083	0.044	0.068	-0.115	0.062	-0.013	-0.031
ADC lbp 3D m1 firstorder 10Percentile	-0.008	0.004	-0.736	0.076	0.067	0.195	0.044	0.039	-0.043	-0.038	-0.013	0.076	0.019	0.068	0.046	0.057	-0.062	-0.084	-0.03	0.042	0.043	-0.034	-0.03	-0.002

**ADC feature name**	**ADC F1**	**ADC F2**	**ADC F3**	**ADC F4**	**ADC F5**	**ADC F6**	**ADC F7**	**ADC F8**	**ADC F9**	**ADC F10**	**ADC F11**	**ADC F12**	**ADC F13**	**ADC F14**	**ADC F15**	**ADC F16**	**ADC F17**	**ADC F18**	**ADC F19**	**ADC F20**	**ADC F21**	**ADC F22**	**ADC F23**	**ADC F24**
ADC lbp 3D m1 firstorder 90Percentile	0.335	0.076	0.454	-0.143	-0.097	0.457	0.094	-0.002	-0.028	-0.092	0.089	0.027	0.106	0.041	0.134	0.036	-0.005	-0.042	0.019	0.017	-0.003	-0.107	0.033	-0.038
ADC lbp 3D m1 firstorder InterquartileRange	0.187	0.023	0.708	-0.154	-0.065	0.045	-0.012	-0.053	-0.064	-0.026	0.123	-0.111	0.056	-0.029	0.086	0.008	-0.058	0.036	0.045	-0.006	-0.011	-0.08	-0.017	0.048
ADC lbp 3D m1 firstorder Kurtosis	-0.18	0.001	-0.714	0.084	0.032	-0.199	-0.001	0.064	-0.041	0.048	-0.042	0.034	0.045	0.123	-0.1	-0.019	0.07	0.037	-0.029	0.011	0.025	0.04	0.047	-0.015
ADC lbp 3D m1 firstorder Median	0.08	0.067	-0.087	-0.09	-0.062	0.662	-0.034	0.024	0.074	-0.069	0.109	-0.177	-0.026	-0.159	-0.07	-0.17	0.08	0.046	-0.004	-0.01	0.024	-0.031	0.131	0.004
ADC lbp 3D m1 firstorder RootMeanSquared	0.129	0.089	-0.288	-0.066	-0.041	0.694	0.077	0.00E+00	-0.004	-0.067	0.066	-0.078	0.049	-0.055	0.051	-0.044	-0.004	-0.055	-0.014	-0.02	0.047	-0.056	0.031	-0.047
ADC lbp 3D m1 firstorder Skewness	0.093	-0.058	-0.453	-0.086	0.054	-0.432	0.106	0.052	-0.174	-0.004	0.099	0.06	0.081	0.239	0.208	-0.044	0.006	-0.05	0.032	0.008	0.042	-0.142	0.014	0.087
ADC lbp 3D m1 firstorder Uniformity	0.112	-0.027	-0.728	0.008	-0.024	-0.199	-0.011	-0.014	-0.093	0.024	-0.011	0.003	0.135	0.159	-0.019	-0.02	0.008	0.001	-0.06	0.014	0.036	0.029	0.027	-0.003
ADC lbp 3D m1 glcm JointAverage	-0.244	0.016	0.137	0.027	0.043	0.692	0.053	0.021	0.089	-0.032	-0.103	0.136	-0.025	0.022	-0.016	0.071	-0.046	-0.074	-0.065	0.01	-0.039	0.018	-0.057	-0.097
ADC lbp 3D m1 glcm ClusterProminence	-0.091	0.042	0.706	-0.1	-0.09	0.142	0.005	-0.006	-0.061	-0.01	0.092	-0.072	0.154	0.128	0.005	0.025	-0.056	-0.011	-0.003	-0.023	0.028	-0.125	0.034	0.069
ADC lbp 3D m1 glcm ClusterShade	0.073	-0.082	-0.205	0.001	0.162	-0.392	0.055	0.064	-0.167	0.005	0.07	0.285	-0.051	0.257	0.2	0.015	0.068	-0.174	0.102	0.098	-0.025	-0.067	-0.051	-0.008
ADC lbp 3D m1 glcm Correlation	-0.396	0.18	-0.427	0.195	0.088	0.096	-0.22	-0.02	-0.003	0.016	-0.001	-0.048	-0.009	-0.008	0.05	-0.004	-0.114	-0.067	0.018	0.126	-0.036	-0.132	-0.006	0.259
ADC lbp 3D m1 glcm DifferenceEntropy	-0.249	-0.012	0.679	0.079	0.01	0.235	0.093	0.155	0.072	-0.019	0.036	-0.014	0.019	-0.031	0.03	0.064	-0.026	-0.039	0.011	-0.084	0.043	-0.009	0.07	0.00E+00
ADC lbp 3D m1 glcm DifferenceVariance	0.056	-0.049	0.725	-0.096	-0.102	0.147	0.101	0.063	-0.015	-0.002	0.08	-0.018	0.109	0.122	0.011	0.035	-0.012	-0.045	-0.018	-0.061	0.044	-0.018	0.062	-0.08
ADC lbp 3D m1 glcm JointEnergy	0.559	-0.016	-0.321	-0.264	-0.131	-0.208	-0.024	-0.199	-0.153	0.008	0.009	-0.003	0.075	0.123	-0.024	-0.056	0.032	0.041	-0.023	0.058	-0.033	-0.013	-0.019	-0.066
ADC lbp 3D m1 glcm JointEntropy	-0.408	0.004	0.317	0.367	0.117	0.177	0.085	0.345	0.16	0.004	0.035	-0.04	-0.056	-0.116	0.022	0.038	-0.034	-0.064	0.016	-0.086	0.046	0.01	0.094	0.052
ADC lbp 3D m1 glcm SumEntropy	-0.48	0.041	0.466	0.21	0.074	0.249	0.029	0.197	0.1	-0.009	0.039	-0.037	-0.019	-0.079	0.028	0.057	-0.046	-0.06	0.022	-0.05	0.022	-0.027	0.074	0.085
ADC lbp 3D m1 glrlm HGLRE	-0.267	0.068	0.245	-0.031	0.048	0.668	0.089	0.034	0.057	-0.031	-0.043	0.128	-0.01	-0.014	0.007	0.102	-0.009	-0.038	-0.019	-0.028	-0.024	0.019	0.002	-0.089
ADC lbp 3D m1 glszm GLNUN	0.268	-0.011	-0.632	-0.188	-0.036	-0.23	-0.043	-0.127	-0.128	-0.034	0.041	-0.082	0.143	0.095	-0.058	-0.049	0.016	-0.042	-0.019	0.002	0.05	0.004	-0.024	0
ADC lbp 3D m1 glszm GrayLevelVariance	0.171	0.008	0.738	0.05	-0.091	0.111	0.08	0.075	0.017	0.042	0.053	0.028	-0.009	0.031	0.029	0.003	0.04	0.059	0.002	0.004	-0.054	-0.079	0.092	-0.007
ADC lbp 3D m1 glszm HGLZE	-0.252	0.079	0.104	0.07	0.077	0.675	0.101	0.06	0.048	-0.009	-0.046	0.167	-0.034	0.005	0.068	0.149	-0.01	-0.055	0.009	-0.032	-0.022	0.004	0.027	-0.046
ADC lbp 3D m1 glszm LALGLE	0.032	-0.04	0.071	0.481	-0.068	-0.292	0.061	0.077	-0.157	0.391	0.031	-0.073	0.086	0.153	0.033	0.023	0.054	0.145	0.012	0.032	-0.054	0.1	0.169	0.092
ADC lbp 3D m1 glszm LGLZE	0.499	0.006	0.509	-0.014	-0.12	-0.256	-0.031	0.019	-0.044	0.019	0.02	-0.083	0.073	0.021	-0.077	-0.004	0.006	0.076	0.034	0.002	-0.071	0.042	0.086	-0.016
ADC lbp 3D m1 glszm SZNU	-0.144	-0.034	0.184	0.26	0.078	0.05	0.17	0.663	0.045	0.105	0.124	0.022	-0.049	0.007	-0.046	-0.034	0.007	-0.01	0.014	0.043	-0.015	-0.035	-0.039	-0.028
ADC lbp 3D m1 glszm SmallAreaEmphasis	0.24	0.036	0.321	-0.578	-0.1	0.237	-0.007	-0.072	-0.004	0.001	-0.045	0.081	-0.06	0.116	0.02	0.004	0.004	0.051	0.005	0.054	0.07	-0.061	0.046	0.004
ADC lbp 3D m1 glszm SAHGLE	-0.095	0.096	0.14	-0.201	0.028	0.665	0.066	0.041	0.043	-0.01	-0.045	0.185	-0.064	0.061	0.092	0.142	-0.01	-0.028	0.03	-0.022	0	-0.069	0.072	0.004
ADC lbp 3D m1 gldm DependenceEntropy	-0.464	-0.013	0.117	0.513	0.124	0.069	0.064	0.266	0.116	0.055	0.017	-0.007	-0.076	-0.091	0.031	0.07	-0.004	-0.044	-0.013	-0.009	-0.01	0.037	0.126	-0.029
ADC lbp 3D m1 gldm DependenceVariance	-0.098	-0.047	-0.433	0.513	0.02	-0.06	0.047	0.134	-0.027	0.104	-0.059	0.083	0.035	0.156	0.07	0.102	0.025	0.024	-0.078	0.131	0.046	0.079	0.196	-0.032
ADC lbp 3D m1 gldm LDHGLE	-0.213	-0.03	-0.241	0.604	0.06	0.186	0.089	0.134	0.016	0.079	-0.096	0.125	0.012	0.051	-0.018	0.105	0.003	0.019	-0.106	0.085	-0.04	0.092	0.094	-0.109
ADC lbp 3D m1 gldm SDE	0.225	0.035	0.359	-0.629	-0.093	0.176	-0.022	-0.111	-0.026	-0.044	-0.003	0.022	-0.023	0.044	-0.017	-0.032	0.001	0.023	0.038	0.001	0.054	-0.055	-0.026	0.009
ADC lbp 3D m1 gldm SDHGLE	0.007	0.081	0.308	-0.492	-0.034	0.517	0.016	-0.039	0.016	-0.04	-0.009	0.098	-0.023	0.032	0.029	0.047	0.004	-0.004	0.043	-0.035	0.028	-0.075	0.021	0.006
ADC lbp 3D m1 gldm SDLGLE	0.582	0.01	0.444	-0.202	-0.125	-0.141	-0.039	-0.036	-0.047	-0.002	-0.001	-0.064	0.059	0.033	-0.065	-0.012	-0.002	0.055	0.059	-0.03	-0.041	0.017	0.024	-0.012

ADC feature name	ADC F1	ADC F2	ADC F3	ADC F4	ADC F5	ADC F6	ADC F7	ADC F8	ADC F9	ADC F10	ADC F11	ADC F12	ADC F13	ADC F14	ADC F15	ADC F16	ADC F17	ADC F18	ADC F19	ADC F20	ADC F21	ADC F22	ADC F23	ADC F24
ADC lbp 3D m1 ngtdm Complexity	0.04	-0.009	0.733	-0.195	-0.058	0.124	0.079	-0.066	-0.034	-0.034	0.035	0.015	0.082	0.054	0.029	0.053	-0.01	0.032	-0.001	-0.045	-0.055	-0.008	-0.059	-0.015
ADC lbp 3D m2 firstorder 90Percentile	0.093	0.014	0.397	-0.085	-0.002	0.494	-0.05	-0.013	-0.041	0.004	0.12	0.023	0.048	0.025	-0.022	-0.363	0.125	-0.044	0.003	0.077	-0.026	-0.119	0.028	-0.032
ADC lbp 3D m2 firstorder InterquartileRange	0.147	0.018	0.727	-0.101	-0.035	0.005	-0.069	-0.083	-0.09	0.004	0.051	-0.014	0.11	0.069	0.05	-0.131	-0.022	-0.029	-0.026	0.016	0.002	-0.019	-0.058	0.091
ADC lbp 3D m2 firstorder Kurtosis	-0.268	0.008	-0.561	0.13	0.055	0.186	0.056	0.064	0.038	0.017	-0.15	0.131	-0.018	0.107	-0.209	0.219	-0.024	0.039	0.002	0.05	-0.04	0.121	0.055	-0.089
ADC lbp 3D m2 firstorder Maximum	-0.184	0.006	0.057	0.357	0.011	-0.002	0.089	0.29	0.063	0.033	0.057	0.036	-0.067	-0.217	0.088	-0.394	0.046	0.01	-0.111	-0.042	0.216	-0.004	0.078	0.07
ADC lbp 3D m2 firstorder Median	0.171	-0.001	-0.376	0.069	0.044	0.536	-0.148	0.004	0.018	0.024	-0.009	-0.085	-0.041	0.016	-0.229	-0.202	0.03	-0.007	0.043	-0.019	-0.068	-0.01	0.084	0.147
ADC lbp 3D m2 firstorder Range	-0.522	0.01	0.31	0.209	0.128	0.195	0.086	0.175	0.106	0.031	-0.105	0.281	-0.088	-0.076	0.025	-0.048	0.01	0.012	-0.048	-0.022	0.042	0.101	0.002	-0.008
ADC lbp 3D m2 firstorder RootMeanSquared	0.149	0.026	-0.535	0.081	0.044	0.46	-0.136	-0.025	0.003	-0.003	-0.002	-0.003	0.002	0.022	-0.14	-0.222	0.048	-0.064	0.013	0.052	0.006	-0.066	-0.007	0.082
ADC lbp 3D m2 firstorder Skewness	0.056	-0.001	-0.14	-0.111	-0.053	-0.513	0.157	-0.042	-0.111	-0.07	0.199	-0.006	0.08	-0.044	0.439	-0.035	0.095	-0.035	-0.026	-0.084	0.097	-0.085	-0.137	-0.062
ADC lbp 3D m2 glcm JointAverage	-0.362	-0.02	0.046	0.129	0.168	0.537	-0.047	0.026	0.118	0.001	-0.159	0.29	-0.085	0.044	-0.107	-0.023	-0.002	-0.061	-0.037	0.087	-0.06	0.064	-0.095	0.027
ADC lbp 3D m2 glcm ClusterProminence	-0.165	0.016	0.74	-0.006	-0.021	0.014	-0.053	-0.023	-0.051	0.006	0	-0.005	0.139	0.035	-0.076	-0.064	-0.05	0.021	-0.052	0.034	0.069	-0.028	0.065	0.105
ADC lbp 3D m2 glcm ClusterShade	0.062	-0.027	-0.268	-0.03	-0.025	-0.443	0.106	-0.042	-0.103	-0.033	0.145	0.158	-0.05	-0.073	0.337	0.056	0.195	-0.011	0.01	-0.079	-0.006	0.055	-0.167	-0.249
ADC lbp 3D m2 glcm Correlation	-0.253	0.141	-0.555	0.176	0.087	-0.223	-0.12	-0.068	-0.08	-0.003	-0.039	0.011	-0.083	-0.062	-0.084	-0.119	-0.026	0.009	0.004	0.044	0.085	-0.003	0.042	0.187
ADC lbp 3D m2 glcm DifferenceVariance	-0.062	-0.065	0.749	-0.041	-0.036	0.107	0.014	0.062	0.033	0.02	0.044	-0.015	0.172	0.084	-0.015	-0.006	0.019	-0.002	-0.01	-0.026	0.049	0.008	0.028	-0.053
ADC lbp 3D m2 glcm MaximumProbability	0.419	-0.027	-0.434	-0.28	-0.164	-0.198	-0.044	-0.237	-0.141	-0.015	0.022	-0.05	0.121	0.111	-0.012	-0.057	0.065	0.039	0.025	0.022	-0.002	-0.023	-0.065	-0.113
ADC lbp 3D m2 glcm SumEntropy	-0.513	0.033	0.456	0.257	0.121	0.098	-0.006	0.199	0.088	0.01	0.008	-0.002	-0.019	-0.095	-0.007	0	-0.016	-0.026	0.002	-0.053	0.058	0.033	0.094	0.104
ADC lbp 3D m2 glrlm LRHGLE	-0.37	-0.004	0.035	0.28	0.17	0.502	0	0.047	0.074	0.036	-0.132	0.297	-0.038	0.027	-0.113	-0.045	0.022	-0.03	0.012	0.047	-0.045	0.092	-0.046	0.021
ADC lbp 3D m2 glrlm LRLGLE	0.591	-0.012	0.353	-0.082	-0.145	-0.241	-0.006	-0.032	-0.077	0.003	0.062	-0.188	0.067	0.029	-0.055	0.022	-0.022	0.044	0.049	-0.041	-0.056	0.026	0.089	-0.007
ADC lbp 3D m2 glrlm SRHGLE	-0.397	0.031	0.141	0.061	0.164	0.538	-0.028	0.051	0.085	0.014	-0.132	0.316	-0.056	0.03	-0.073	-0.025	0.018	-0.03	0.01	0.035	-0.047	0.073	-0.032	0.025
ADC lbp 3D m2 glszm GLNUN	0.266	0.037	-0.67	-0.197	-0.042	-0.038	-0.062	-0.139	-0.11	-0.021	0.02	-0.064	0.04	0.09	-0.03	0.051	-0.046	-0.102	-0.029	0.037	-0.006	-0.072	-0.047	0.011
ADC lbp 3D m2 glszm GrayLevelVariance	-0.068	-0.023	0.748	0.161	-0.051	0.006	0.041	0.05	0.04	0.025	0.019	0.007	0.084	0.029	-0.049	-0.1	0.052	0.104	0.021	-0.011	0.001	0.037	0.053	-0.003
ADC lbp 3D m2 glszm HGLZE	-0.459	0.029	-0.001	0.114	0.142	0.482	0.024	0.066	0.073	0.03	-0.127	0.344	-0.045	0.035	-0.016	0.038	0.026	-0.028	0.014	0.03	-0.027	0.062	-0.077	-0.027
ADC lbp 3D m2 glszm LAHGLE	-0.1	-0.044	-0.168	0.639	0.075	0.132	0.182	0.059	-0.093	0.184	-0.034	0.108	-0.044	0.021	-0.094	-0.011	0.083	0.189	-0.009	0.049	-0.026	0.023	-0.095	-0.011
ADC lbp 3D m2 glszm LALGLE	0.168	-0.049	-0.125	0.506	-0.115	-0.285	0.191	0.031	-0.145	0.182	0.168	-0.155	0.062	0.028	-0.016	-0.009	0.095	0.252	0.014	-0.048	0.026	-0.05	0.001	-0.016
ADC lbp 3D m2 glszm LGLZE	0.609	-0.001	0.372	0.037	-0.144	-0.181	-0.003	0.007	-0.045	-0.003	0.049	-0.158	0.039	0.032	-0.095	-0.036	-0.015	0.042	0.043	-0.019	-0.091	0.046	0.114	-0.033
ADC lbp 3D m2 glszm SmallAreaEmphasis	-0.234	0.118	0.29	-0.593	-0.049	0.072	-0.021	-0.009	-0.077	-0.008	-0.085	-0.022	-0.009	0.085	0.051	0.08	0.086	0.133	0.017	0.006	-0.016	-0.006	0.031	-0.024
ADC lbp 3D m2 glszm SAHGLE	-0.524	0.087	0.036	-0.155	0.114	0.402	0.008	0.052	0.016	0.031	-0.138	0.286	-0.037	0.09	0.03	0.112	0.04	0.02	0.024	0.009	-0.03	0.023	-0.068	-0.003
ADC lbp 3D m2 glszm ZoneEntropy	-0.384	-0.059	0.083	0.539	0.142	0.044	0.12	0.304	0.108	0.067	0.034	0.066	-0.044	-0.105	0.024	-0.021	-0.051	-0.024	0.01	-0.039	0.06	0.082	0.069	0.003
ADC lbp 3D m2 glszm ZoneVariance	-0.082	-0.035	-0.205	0.606	-0.011	0.022	0.226	0.068	-0.106	0.2	0.027	0.047	-0.037	0.015	-0.046	0.007	0.125	0.252	-0.016	0.022	0.001	-0.016	-0.1	-0.054

ADC feature name	ADC F1	ADC F2	ADC F3	ADC F4	ADC F5	ADC F6	ADC F7	ADC F8	ADC F9	ADC F10	ADC F11	ADC F12	ADC F13	ADC F14	ADC F15	ADC F16	ADC F17	ADC F18	ADC F19	ADC F20	ADC F21	ADC F22	ADC F23	ADC F24
ADC lbp 3D m2 gldm DependenceVariance	-0.162	-0.081	-0.435	0.527	-0.013	-0.078	0.074	-0.001	0.013	0.058	-0.005	-0.072	0.099	0.045	-0.101	0.002	0.145	0.039	0.054	0.035	0.079	0.032	-0.074	-0.156
ADC lbp 3D m2 gldm LDLGLE	0.282	-0.071	-0.094	0.176	-0.131	-0.475	0.05	-0.047	-0.124	0.048	0.18	-0.312	0.175	0.03	0.022	0.044	0.027	0.111	0.052	-0.082	0.069	-0.044	0.063	0.023
ADC lbp 3D m2 gldm SDE	-0.016	0.091	0.346	-0.691	-0.058	0.061	-0.048	-0.029	-0.042	-0.028	-0.05	0.009	-0.035	0.042	0.055	0.039	0.033	0.052	-0.015	-0.007	-0.013	-0.019	0.033	-0.019
ADC lbp 3D m2 gldm SDHGLE	-0.351	0.107	0.245	-0.502	0.052	0.339	-0.042	0.02	0.016	-0.007	-0.105	0.21	-0.049	0.065	0.035	0.06	0.04	0.023	0	-0.002	-0.028	0.003	-0.009	-0.006
ADC lbp 3D m2 gldm SDLGLE	0.608	0.017	0.395	-0.139	-0.146	-0.128	-0.009	0.00E+00	-0.049	-0.017	0.006	-0.132	0.02	0.053	-0.053	-0.049	-0.012	0.026	0.031	-0.008	-0.079	0.041	0.099	-0.053
ADC lbp 3D m2 ngtdm Complexity	-0.196	0.019	0.695	0.011	0.037	0.177	0.105	0.106	-0.014	0.018	-0.016	0.114	0.064	-0.033	0.03	-0.115	0.008	-0.031	-0.07	0.001	0.11	0.042	0.007	0.058
ADC lbp 3D k firstorder 10Percentile	0.513	0.017	0.465	-0.12	-0.118	-0.046	-0.033	-0.048	-0.064	0.012	0.149	-0.12	-0.022	0.008	0.037	-0.09	-0.018	0.196	-0.059	-0.107	-0.054	0.029	-0.059	0.078
ADC lbp 3D k firstorder 90Percentile	0.14	-0.08	0.076	-0.141	0.062	-0.053	0.723	0.082	-0.092	0.064	0.071	-0.05	0.029	0.015	0.062	-0.028	-0.066	0.104	0.003	-0.057	0.012	0.074	-0.042	-0.011
ADC lbp 3D k firstorder Kurtosis	-0.026	-0.068	0.089	0.098	0.041	0.07	0.039	-0.001	0.736	0.09	0.024	0.012	-0.033	-0.019	-0.037	0.019	0.023	0.009	0.026	0.03	-0.056	-0.059	0.019	-0.083
ADC lbp 3D k firstorder Maximum	-0.024	-0.07	0.142	0.066	0.123	0.053	0.53	0.198	0.447	0.086	0.14	-0.025	-0.029	-0.048	-0.014	-0.013	-0.075	0.059	0.046	0.002	-0.015	0.028	0.037	0.021
ADC lbp 3D k firstorder Median	0.415	-0.037	0.325	-0.244	-0.042	-0.097	0.343	-0.009	-0.152	0.026	0.186	-0.087	0.03	-0.007	0.098	-0.04	-0.055	0.256	-0.047	-0.09	-0.047	0.015	0.056	-0.038
ADC lbp 3D k firstorder Minimum	0.679	0.035	0.001	-0.08	-0.127	-0.069	-0.17	-0.174	0.015	-0.039	-0.028	-0.092	0.017	-0.031	0.075	-0.009	-0.032	0.137	-0.057	0.052	0.014	0.001	0.043	0.032
ADC lbp 3D k firstorder RMAD	0.151	-0.089	-0.043	-0.137	0.048	-0.061	0.729	0.041	-0.157	0.053	-0.007	-0.009	0.043	0.01	0.05	-0.008	-0.042	0.034	0.049	0.004	-0.001	0.063	0.014	-0.038
ADC lbp 3D k firstorder RootMeanSquared	0.385	-0.059	0.291	-0.192	-0.005	-0.067	0.499	0.022	-0.098	0.043	0.16	-0.095	0.026	-0.002	0.089	-0.031	-0.064	0.211	-0.039	-0.088	-0.026	0.035	0.005	0.013
ADC lbp 3D k firstorder Skewness	0.014	-0.079	-0.026	0.243	0.052	0.081	0.135	0.048	0.674	0.079	-0.031	0.044	-0.053	-0.016	-0.05	0.061	0.077	-0.106	-0.015	0.074	0.011	-0.029	-0.06	-0.016
ADC lbp 3D k glcm ClusterShade	0.155	-0.066	-0.063	0.146	0.027	0.076	0.638	0.079	0.298	0.093	-0.014	0.017	0.064	0.027	-0.095	-0.005	0.01	-0.046	0.014	0.052	0.012	0.026	-0.05	0.029
ADC lbp 3D k glcm DifferenceVariance	0.139	-0.096	-0.031	0.08	0.019	0.085	0.741	0.068	0.06	-0.002	0.022	-0.013	0.028	0.005	-0.008	0.034	-0.027	-0.058	-0.042	0.033	0.059	0.021	0.016	0.008
ADC lbp 3D k glcm JointEnergy	0.17	0.029	0.359	-0.086	-0.069	0.09	-0.573	-0.056	0.011	-0.034	0.135	-0.104	0.048	0.065	-0.026	-0.059	-0.032	0.15	0.018	-0.117	-0.099	0.036	0.061	0.129
ADC lbp 3D k glcm Imc2	0.421	-0.067	-0.179	-0.38	-0.097	-0.2	0.237	-0.205	-0.011	0.115	-0.086	0.111	-0.078	0.127	0.015	-0.012	0.022	0.006	0.062	0.111	-0.056	-0.002	-0.149	-0.028
ADC lbp 3D k glcm Idmn	-0.187	-0.095	0.282	0.093	0.132	0.185	0.109	0.213	0.547	0.057	0.069	0	-0.051	-0.025	0.012	-0.115	0.017	0.08	0.015	-0.118	-0.115	0.028	0.007	0.157
ADC lbp 3D k glcm MaximumProbability	0.045	0.01	0.486	-0.087	-0.012	0.083	-0.42	0.017	0.006	-0.043	0.169	-0.118	0.01	0.023	0.098	-0.022	-0.105	0.229	-0.024	-0.162	-0.041	0.038	0.092	0.169
ADC lbp 3D k glrlm GLNUN	0.171	0.101	0.091	-0.248	-0.126	0.06	-0.667	-0.137	0.039	-0.052	0.012	-0.046	0.021	0.055	-0.018	-0.086	0.004	0.086	-0.059	-0.029	-0.057	-0.061	0.027	0.073
ADC lbp 3D k glrlm HGLRE	-0.289	-0.069	0.238	-0.094	0.095	-0.027	0.615	0.049	-0.028	0.08	0.178	-0.102	0.004	-0.002	0.062	-0.006	-0.045	0.195	-0.032	-0.078	-0.028	0.038	0.009	0.013
ADC lbp 3D k glrlm LGLRE	0.47	0.014	-0.328	0.237	-0.09	0.035	-0.281	0.001	0.056	-0.045	-0.167	0.146	0.015	-0.003	-0.123	0.036	0.073	-0.268	0.056	0.126	0.024	-0.03	-0.022	-0.051
ADC lbp 3D k glrlm RunEntropy	-0.198	-0.101	0.255	0.617	0.093	-0.037	0.289	0.174	0.051	0.076	0.09	-0.027	-0.032	-0.006	0.037	0.02	-0.023	0.022	0.027	-0.005	-0.015	0.021	0.011	0.033
ADC lbp 3D k glrlm ShortRunEmphasis	0.019	0.047	-0.383	-0.632	0.007	0.055	0.234	-0.039	-0.027	-0.024	-0.071	0.081	0.007	-0.034	-0.004	0.063	0.02	-0.063	-0.004	0.004	0.049	0.007	-0.004	-0.068
ADC lbp 3D k glrlm SRHGLE	-0.209	-0.057	0.079	-0.274	0.086	-0.015	0.667	0.037	-0.027	0.078	0.136	-0.068	0.007	-0.012	0.052	0.011	-0.022	0.159	-0.014	-0.057	-0.017	0.033	0.007	-0.013
ADC lbp 3D k glrlm SRLGLE	0.399	0.037	-0.42	0.049	-0.078	0.087	-0.253	-0.011	0.047	-0.091	-0.19	0.18	0.028	-0.016	-0.13	0.068	0.055	-0.309	0.038	0.11	0.055	-0.014	-0.013	-0.063
ADC lbp 3D k glszm GLNU	-0.159	-0.031	0.065	0.092	0.144	0.049	0.111	0.726	0.063	0.08	0.039	-0.009	0.046	0.004	-0.015	-0.032	0.022	0.011	-0.021	0.024	-0.024	0.06	0.005	0.006
ADC lbp 3D k glszm GLNUN	-0.116	0.022	0.018	-0.004	0.039	-0.078	-0.68	0.054	0.035	0.017	-0.033	-0.01	0.153	-0.069	-0.036	-0.03	-0.018	0.163	-0.026	0.022	0.045	0.077	0.112	-0.055
ADC lbp 3D k glszm GrayLevelVariance	-0.085	-0.107	0.272	0.119	0.01	0.098	0.602	0.073	0.215	-0.007	0.193	-0.023	-0.045	-0.033	0.02	-0.024	-0.095	0.061	-0.075	-0.134	-0.01	-0.13	-0.067	0.022

ADC feature name	ADC F1	ADC F2	ADC F3	ADC F4	ADC F5	ADC F6	ADC F7	ADC F8	ADC F9	ADC F10	ADC F11	ADC F12	ADC F13	ADC F14	ADC F15	ADC F16	ADC F17	ADC F18	ADC F19	ADC F20	ADC F21	ADC F22	ADC F23	ADC F24
ADC lbp 3D k glszm HGLZE	-0.039	-0.091	0.014	0.415	0.083	0.04	0.58	0.089	0.168	0.082	-0.052	0.078	0.032	-0.012	-0.016	-0.005	0.029	0.056	0.009	0.059	-0.04	-0.102	0.165	-0.022
ADC lbp 3D k glszm LAHGLE	-0.08	-0.031	0.165	0.508	0.028	-0.124	-0.062	0.438	-0.082	0.159	0.131	0.103	-0.035	-0.052	-0.015	-0.071	-0.023	0.131	0.016	0.111	-0.037	-0.03	0.029	-0.113
ADC lbp 3D k glszm LGLZE	-0.118	0.007	0.303	-0.418	-0.107	0.039	-0.213	-0.058	-0.078	-0.109	0.191	-0.114	-0.081	-0.015	0.054	-0.024	-0.113	0.04	-0.13	-0.242	0.068	0.004	-0.252	0.107
ADC lbp 3D k glszm SZNU	-0.122	-0.037	0.002	0.1	0.093	0.121	0.382	0.608	0.084	0.094	0.048	0.023	0.024	0.103	-0.096	-0.001	0.019	-0.092	0.013	0.011	-0.059	-0.051	-0.019	0.076
ADC lbp 3D k glszm SAHGLE	0.082	-0.039	0.01	0.314	0.00E+00	0.11	0.547	0.084	0.207	-0.006	-0.057	0.106	0.073	0.026	-0.057	-0.042	-0.003	-0.006	-0.035	0.005	-0.052	-0.227	0.245	0.056
ADC lbp 3D k glszm ZonePercentage	0.183	0.007	-0.026	-0.706	-0.004	-0.01	0.205	-0.079	-0.117	0.014	-0.012	0.027	0.039	0.077	0.01	-0.004	0.09	0.084	0.051	0.004	0.001	0.054	0.055	-0.034
ADC lbp 3D k gldm DependenceEntropy	-0.316	-0.029	0.026	0.56	0.133	-0.011	0.281	0.221	0.104	0.047	0.019	0.011	-0.066	-0.029	0.04	0.102	0.005	-0.13	0.084	0.01	0.043	0.082	-0.025	0.053
ADC lbp 3D k gldm DependenceVariance	-0.059	-0.001	0.438	0.527	0.052	0.036	0.004	0.099	-0.048	0.022	0.212	-0.076	0.019	0.069	0.018	0.038	-0.014	-0.029	0.085	-0.095	-0.027	0.148	0.016	0.13
ADC lbp 3D k gldm LDLGLE	0.367	-0.046	0.091	0.626	-0.051	-0.065	-0.153	0.07	0.019	0.093	0.039	0.003	-0.029	0.064	-0.056	-0.033	0.083	-0.084	0.101	0.074	-0.051	0.014	-0.025	0.021
ADC lbp 3D k gldm SDE	0.146	0.046	-0.166	-0.673	0.017	0.072	0.297	0.02	-0.07	-0.013	-0.015	0.044	0.063	0.056	-0.025	0.026	0.063	0.016	0.051	-0.003	0.034	0.027	0.098	-0.002
ADC lbp 3D k gldm SDHGLE	0.18	-0.027	-0.099	-0.458	0.021	0.091	0.565	0.025	0.031	-0.006	-0.02	0.044	0.115	0.071	0.003	-0.025	0.046	0.045	0.051	0.025	-0.028	-0.03	0.14	0.005
ADC lbp 3D k gldm SDLGLE	-0.039	0.027	-0.083	-0.735	0.037	0.115	0.127	0.057	-0.082	-0.032	0.007	0.016	-0.023	0	-0.09	0.065	0.01	0.032	-0.013	-0.06	0.115	0.033	0.023	0.026
ADC lbp 3D k ngtdm Busyness	-0.097	0.00E+00	-0.092	0.466	0.041	-0.063	-0.07	0.517	-0.147	0.179	-0.047	0.151	-0.018	0.027	0.004	-0.005	-0.003	0.022	0.08	0.161	0	-0.015	0.007	-0.091
ADC lbp 3D k ngtdm Complexity	-0.018	-0.089	0.052	0.086	0.086	0.128	0.559	0.132	0.452	0.044	0.022	0.033	0.014	-0.057	-0.078	-0.098	-0.01	-0.052	0.009	0.002	-0.025	0.036	0.026	0.083
ADC lbp 3D k ngtdm Contrast	0.247	0.054	-0.412	-0.107	-0.101	-0.195	0.085	-0.158	-0.434	0.016	-0.075	0.047	0.056	0.007	-0.05	0.138	0.014	-0.094	0.066	0.131	0.067	-0.019	-0.016	-0.207
ADC lbp 3D k ngtdm Strength	0.678	-0.035	0.091	-0.221	-0.114	0.012	0.039	-0.235	0.172	-0.01	-0.008	0.038	0.045	0.049	0.042	-0.047	-0.049	0.073	0.009	0.028	-0.083	-0.03	-0.048	0.02

Table S12 Factor loadings for the 32 T1C factors. A darker grey in the column cell correspond to a higher loading measuring the association between features and factors. Abbreviations: T1C = post-contrast T1; LDLGLE = Large Dependence Low Gray Level Emphasis; LDHGLE = Large Dependence High Gray Level Emphasis; SDLGLE = Small Dependence Low Gray Level Emphasis; LALGLE = Large Area Low Gray Level Emphasis; GLNUN = Gray level non uniformity normalized; glcm = gray level co-occurrence matrix; gldm = Gray Level Dependence Matrix; glszm = Gray Level Size Zone Matrix; ngtdm = Neighbouring Gray Tone Difference Matrix; glrlm = Gray Level Run Length Matrix; wav = wavelet; lbp = local binary pattern; L = Low-pass filter; H = High-pass filter.

T1C feature name	T1C F1	T1C F2	T1C F3	T1C F4	T1C F5	T1C F6	T1C F7	T1C F8	T1C F9	T1C F10	T1C F11	T1C F12	T1C F13	T1C F14	T1C F15	T1C F16	T1C F17	T1C F18	T1C F19	T1C F20	T1C F21	T1C F22	T1C F23	T1C F24	T1C F25	T1C F26	T1C F27	T1C F28	T1C F29	T1C F30	T1C F31	T1C F32
T1C glrlm SRLGLE	-0.754	-0.165	0.049	0.025	-0.03	-0.007	-0.081	-0.08	-0.087	0.065	-0.032	0.002	-0.069	-0.017	0.052	-0.058	-0.072	-0.061	-0.069	-0.021	-0.023	-0.115	0.014	0.056	0.085	0.004	-0.005	0.05	0.052	0.029	0.065	-0.018
T1C gldm SDHGLE	0.161	0.637	0.017	-0.003	-0.029	-0.002	0.094	0.381	0.093	-0.04	0.01	0.052	0.063	0.195	0.07	0.038	0.069	-0.028	0.025	-0.023	-0.015	0.057	0.042	-0.032	-0.063	-0.005	0.091	-0.015	-0.059	-0.071	-0.022	0.047
T1C gldm LDLGLE	-0.242	-0.5	-0.181	0.113	0.299	0.119	-0.114	-0.025	-0.15	0.191	-0.019	-0.039	-0.09	0.04	0.108	-0.114	-0.067	0.048	-0.099	-0.082	0.027	-0.099	0.031	0.058	0.039	0.085	-0.088	0.036	0.124	0.054	0.13	-0.088
T1C gldm LDHGLE	0.354	0.232	-0.443	-0.022	0.134	0.097	0.172	0.074	0.293	-0.036	-0.033	0.105	0.016	0.06	0.059	-0.008	-0.003	0.001	0.099	0.004	-0.022	0.114	0.005	-0.124	-0.082	0.075	0.089	0.033	-0.139	-0.133	-0.041	0.094
T1C gldm DependenceVariance	0.169	-0.528	-0.392	0.094	0.377	0.131	-0.02	0.001	-0.009	0.113	-0.025	0.005	0.077	-0.064	0.059	-0.041	-0.014	0.087	-0.029	-0.027	0.005	-0.035	0.015	-0.04	0.001	0.084	-0.076	0.018	-0.012	0.064	0.071	-0.005
T1C glcm JointAverage	0.278	0.61	-0.098	-0.037	-0.039	0.016	0.136	0.193	0.178	-0.12	-0.014	0.078	0.042	0.157	0.069	0.039	0.072	-0.031	0.09	-0.009	-0.04	0.102	0.013	-0.085	-0.07	-0.021	0.105	-0.027	-0.084	-0.107	-0.061	0.082

T1C feature name	T1C F1	T1C F2	T1C F3	T1C F4	T1C F5	T1C F6	T1C F7	T1C F8	T1C F9	T1C F10	T1C F11	T1C F12	T1C F13	T1C F14	T1C F15	T1C F16	T1C F17	T1C F18	T1C F19	T1C F20	T1C F21	T1C F22	T1C F23	T1C F24	T1C F25	T1C F26	T1C F27	T1C F28	T1C F29	T1C F30	T1C F31	T1C F32
T1C glcm Idn	0.64	-0.168	-0.213	0.082	0.101	0.098	0.129	0.098	0.216	0.134	0.009	-0.05	0.167	-0.037	0.018	0.007	0.034	0.025	-0.045	-0.031	0.004	-0.037	-0.045	-0.122	0.055	0.022	0.111	-0.01	-0.115	0.05	0.05	-0.008
T1C glcm ClusterShade	0.094	0.243	0.082	0.124	0.011	0.029	0.117	0.548	0.013	-0.039	0.137	-0.037	0.068	-0.015	-0.027	0.054	0.095	0.025	-0.114	0.015	0.003	0.034	0.065	0.03	0.447	0.047	0.085	0.027	-0.004	0.036	-0.04	0.008
T1C glcm Correlation	0.527	-0.078	-0.221	0.005	0.135	0.191	0.032	-0.034	0.028	0.241	0.074	-0.024	-0.176	0.127	0.085	0.116	-0.167	-0.007	-0.012	-0.002	0.008	-0.111	0.082	0.004	0.203	0.043	0.227	-0.02	-0.102	-0.062	0.062	-0.055
T1C glcm ClusterTendency	0.148	0.658	0.008	0.019	0.011	0.198	-0.003	0.256	-0.069	0.057	0.054	-0.007	-0.041	0.265	0.106	0.045	-0.002	-0.01	-0.034	-0.03	0.032	-0.05	0.08	0.029	0.064	0.048	0.079	0.055	-0.001	-0.039	0.065	-0.079
T1C glcm InverseVariance	0.077	-0.688	-0.256	0.044	0.2	0.106	0.022	-0.024	0.063	0.22	0.022	-0.046	0.046	-0.07	0.047	-0.057	-0.033	0.017	0.036	-0.043	0.011	-0.042	0.021	-0.047	0.026	0.092	0.001	-0.031	-0.053	0.083	0.032	-0.032
T1C glcm SumEntropy	0.565	0.55	-0.078	-0.001	-0.011	0.027	-0.004	0.119	0.033	0.01	0.044	0.001	-0.075	0.158	0.004	0.044	-0.003	-0.036	-0.009	-0.047	-0.019	-0.023	0.005	0.036	0.031	0.029	0.072	0.035	0.049	0.028	-0.004	-0.008
T1C glcm MaximumProbability	-0.745	-0.324	0.058	0.007	0.055	0.089	0.025	-0.031	-0.02	0.027	0.022	-0.014	0.029	-0.041	0.024	-0.012	0.039	0.029	-0.007	-0.034	0.025	-0.046	0.062	-0.054	-0.018	0.037	-0.008	-0.023	-0.041	0.001	0.037	-0.01
T1C glcm DifferenceVariance	0.059	0.636	0.107	0.023	-0.004	0.095	0.033	0.378	-0.118	-0.142	0.001	-0.054	0.041	0.158	-0.001	0.012	0.056	0.003	-0.154	-0.03	-0.01	0.053	0.013	0.021	-0.047	0.002	-0.068	0.06	0.025	0.061	-0.056	-0.072
T1C glcm DifferenceEntropy	0.239	0.724	0.131	-0.008	-0.11	-0.033	-0.006	0.132	-0.068	-0.167	0.023	0.022	-0.013	0.115	-0.037	0.03	0.066	-0.025	-0.025	-0.016	-0.005	0.039	-0.025	0.039	-0.039	-0.03	-0.022	0.03	0.083	0.004	-0.021	0.014
T1C firstorder RootMeanSquared	-0.142	0.497	0.097	-0.195	-0.116	-0.11	-0.061	0.108	0.069	-0.052	-0.034	0.547	0.001	0.053	0.105	-0.006	0.023	-0.013	0.037	-0.002	0.061	0.074	0.024	0.025	-0.066	0.029	0.059	-0.012	0.006	-0.014	0.005	-0.001
T1C firstorder Kurtosis	0.207	-0.304	-0.048	0.083	0.008	0.059	0.244	0.213	0.045	-0.044	0.017	-0.094	0.531	-0.071	-0.004	-0.039	0.077	0.051	-0.121	-0.004	0.029	-0.085	-0.024	-0.155	0.082	0.071	0.025	0.017	-0.06	0.111	0.033	0.069
T1C firstorder Maximum	0.132	0.633	0.129	-0.031	-0.076	0.002	0.015	0.279	0.127	-0.048	-0.023	0.269	0.144	0.107	0.042	0.03	0.069	0.004	-0.128	-0.036	0.039	0.011	-0.037	-0.015	0.021	0.015	0.076	0.028	0.01	0.081	0.044	-0.024
T1C firstorder InterquartileRange	0.125	0.71	0.022	-0.033	-0.054	0.089	-0.077	0.124	-0.151	0.005	0.045	-0.043	-0.112	0.259	0.019	0.06	0.025	0.029	0.022	-0.027	0.028	-0.014	0.037	0.034	-0.043	0.021	0.045	0.033	0.027	-0.041	0.055	-0.091
T1C firstorder Minimum	-0.411	0.03	0.177	-0.211	-0.09	-0.134	-0.195	-0.069	-0.063	0.037	-0.039	0.552	-0.042	-0.065	0.065	-0.029	-0.045	0.006	-0.025	0.008	0.09	-0.003	0.012	0.095	-0.008	0.041	-0.022	0.015	0.082	0.078	0.05	-0.058
T1C ngtdm Complexity	0.151	0.584	-0.005	0.04	0.004	0.05	0.061	0.498	0.064	-0.049	-0.014	-0.013	0.053	0.153	0.034	-0.001	0.076	-0.035	-0.148	-0.038	-0.017	0.027	0.053	0.008	0.003	0.038	0.039	0.036	-0.001	0.034	-0.041	-0.015
T1C ngtdm Strength	-0.297	0.397	0.16	-0.028	-0.063	0.113	0.01	0.163	-0.433	-0.133	0.024	-0.125	0.054	0.061	-0.001	0.256	-0.012	0.057	-0.133	0.043	0.058	-0.03	0.025	-0.014	0.058	-0.079	0.125	0.057	-0.056	-0.056	0.087	-0.008
T1C ngtdm Busyness	0.304	-0.459	-0.206	0.08	0.309	-0.051	-0.106	-0.063	0.328	0.077	-0.06	0.004	-0.101	-0.087	0.067	-0.046	-0.025	0.017	-0.03	0.05	0.04	0.019	0.024	0.083	0.014	0.041	-0.232	-0.012	0.078	-0.003	0.004	-0.082
T1C ngtdm Contrast	-0.323	0.531	0.223	-0.001	-0.044	0.02	-0.055	0.021	-0.347	-0.153	-0.001	-0.018	-0.072	0.154	-0.005	0.12	-0.004	0.13	0.004	0.021	0.044	0.008	-0.006	0.05	-0.086	-0.011	-0.092	-0.008	0.044	-0.064	0.009	-0.114
T1C glszm SZNU	0.396	0.287	-0.102	0.121	-0.018	-0.094	0.009	0.134	0.616	-0.038	-0.013	0.024	0.045	0.05	0.03	-0.044	0.027	-0.005	-0.027	0.004	-0.02	-0.014	-0.003	-0.009	-0.037	-0.016	-0.02	0.012	0.012	-0.051	-0.028	0.021
T1C glszm ZoneEntropy	0.669	0.282	-0.289	0.074	0.144	0.076	0.028	0.094	0.112	0.056	-0.018	0.01	-0.006	0.086	0.009	0.027	-0.004	-0.03	-0.04	-0.028	0.041	0.049	0.02	-0.001	-0.008	0.022	0.011	0.034	0.02	0.018	0.012	0
T1C glszm LAHGLE	0.248	-0.111	-0.354	0.024	0.642	0.083	0.065	0.017	0.18	-0.033	-0.01	0.039	-0.009	-0.043	0.043	0.018	-0.015	0.003	0.046	0.011	-0.007	0.078	0.011	-0.06	-0.034	0.046	0.045	0.013	-0.077	-0.062	-0.015	0.03
T1C wav LHH gldm SDLGLE	-0.735	-0.236	0.023	-0.036	-0.089	0.006	-0.063	-0.039	-0.042	-0.042	0.045	-0.03	-0.113	0.062	-0.007	0.065	0.072	-0.031	0.036	-0.072	-0.057	-0.043	-0.016	0.023	0.019	0.085	0.118	-0.081	0.007	-0.009	-0.051	0.033
T1C wav LHH gldm DependenceEntropy	0.541	0.427	-0.317	0.056	-0.008	-0.051	0.075	0.047	0.158	0.096	-0.05	0.001	-0.117	-0.081	-0.04	-0.074	0.007	0.023	0.021	0.018	-0.015	0.017	-0.051	0.008	0.031	0.076	-0.08	0.02	-0.08	0.07	0.001	0.045
T1C wav LHH gldm LDHGLE	0.342	0.228	-0.235	0.117	0.113	0.026	0.493	0.15	0.223	0.128	0.05	0.048	0.022	0.008	0.077	-0.127	-0.072	0.041	-0.08	0.088	0.02	0.017	0.072	-0.033	0.026	-0.03	-0.051	0.172	-0.047	0.02	0.025	-0.054
T1C wav LHH gldm DependenceVariance	0.212	-0.56	-0.354	0.046	0.296	0.016	-0.025	0.036	-0.036	-0.003	0.028	-0.007	0.008	0.291	0.055	-0.008	0.041	0.053	-0.029	-0.018	0.004	0	0	0.039	-0.001	0.009	-0.033	0.014	-0.092	0.068	-0.006	0.058
T1C wav LHH glcm Idmn	0.585	-0.068	0.026	-0.024	0.06	0.08	0.156	0.064	0.193	0.091	0.123	0.054	0.333	0.012	-0.038	-0.136	-0.125	0.079	-0.085	0.137	0.098	0.019	0.009	0.096	-0.046	-0.094	-0.02	0.084	-0.052	-0.058	0.041	-0.02
T1C wav LHH glcm Imc1	0.637	-0.225	-0.313	0.021	0.026	0.006	-0.06	0.043	0.252	0.001	0.003	0.031	0.001	0.05	0.026	-0.117	0.01	-0.183	-0.01	-0.043	-0.026	-0.007	0.014	0.014	-0.047	0.059	0.029	-0.035	0.034	-0.031	-0.048	-0.023
T1C wav LHH glcm MaximumProbability	-0.061	-0.694	-0.151	0.001	0.196	0.076	-0.043	0.05	-0.081	-0.017	0.055	0.016	0.086	0.322	0.029	0.027	0.013	0.03	-0.067	-0.056	-0.007	-0.034	0.023	0.002	-0.026	0.016	0.026	0.033	0.052	-0.01	-0.002	0.031
T1C wav LHH glcm JointEntropy	0.227	0.733	0.077	0.015	-0.141	-0.055	0.069	0.013	0.089	0.078	-0.03	-0.007	-0.062	-0.188	-0.037	-0.05	-0.001	-0.028	0.039	0.017	-0.004	0.026	-0.044	-0.026	0.065	0.035	-0.05	-0.045	0.011	0.046	0.015	-0.03
T1C wav LHH firstorder RootMeanSquared	-0.016	0.383	-0.235	-0.047	-0.016	-0.053	-0.075	-0.031	-0.076	0.083	-0.092	0.025	0.034	0.001	0.127	0.614	-0.003	0.099	-0.007	-0.068	0.061	0.038	-0.01	0.035	0.069	-0.024	-0.028	0.024	0.007	0.045	-0.022	-0.063
T1C wav LHH firstorder Kurtosis	0.167	-0.132	0.273	0.064	0.019	0.176	0.368	0.012	0.043	0.057	0.02	-0.004	0.505	0.146	-0.025	-0.06	-0.178	0.09	-0.061	0.126	0.02	0.012	-0.04	0.072	-0.002	-0.069	0.016	0.02	-0.076	-0.115	0.056	-0.019

T1C feature name	T1C F1	T1C F2	T1C F3	T1C F4	T1C F5	T1C F6	T1C F7	T1C F8	T1C F9	T1C F10	T1C F11	T1C F12	T1C F13	T1C F14	T1C F15	T1C F16	T1C F17	T1C F18	T1C F19	T1C F20	T1C F21	T1C F22	T1C F23	T1C F24	T1C F25	T1C F26	T1C F27	T1C F28	T1C F29	T1C F30	T1C F31	T1C F32
T1C wav LHH firstorder Maximum	0.186	0.561	0.222	-0.009	-0.057	0.022	0.281	0.012	0.161	0.185	0.012	-0.021	0.24	-0.08	0.011	-0.067	-0.067	0.136	0.007	0.069	-0.007	0.041	-0.079	0.08	0.123	-0.01	0.01	0.003	-0.023	0.039	0.093	0.022
T1C wav LHH firstorder Mean	-0.035	0.119	-0.332	-0.045	-0.018	-0.028	-0.137	-0.069	-0.051	0.044	-0.096	0.008	0.024	0.015	0.13	0.664	0.02	0.047	-0.018	-0.059	0.033	0.033	0.011	0.057	0.02	-0.047	0.016	0.027	-0.003	-0.004	-0.047	-0.049
T1C wav LHH firstorder Skewness	-0.001	-0.074	0.099	0.031	-0.045	0.191	-0.157	0.002	0.078	0.132	-0.106	-0.007	0.595	-0.02	0.073	0.02	0.08	-0.039	0.105	-0.075	-0.142	0.091	-0.189	0.17	0.054	0.033	0.046	-0.093	-0.043	-0.014	0.085	0.031
T1C wav LHH firstorder InterquartileRange	-0.093	0.744	0.129	-0.013	-0.062	-0.142	0.019	-0.01	-0.049	0.051	-0.067	0.021	-0.034	-0.133	0.012	-0.004	-0.015	0.077	0.051	-0.023	0.069	-0.029	-0.037	-0.086	0.102	0.055	-0.118	-0.044	0.019	0.095	0.013	-0.034
T1C wav LHH firstorder Median	-0.133	0.063	-0.256	-0.084	0.008	-0.02	-0.003	-0.042	-0.103	-0.041	0.015	-0.084	-0.163	0.007	0.003	0.599	0.017	0.291	-0.022	0.085	0.196	-0.05	0.157	-0.023	-0.012	0.006	0.054	-0.076	-0.001	-0.063	-0.019	0.083
T1C wav LHH ngtdm Contrast	-0.337	0.497	0.181	-0.039	-0.023	-0.163	-0.034	-0.081	-0.332	-0.034	-0.099	0.026	-0.082	-0.119	0.031	0.099	-0.062	0.185	0.044	0.004	0.073	-0.063	-0.106	-0.062	0.142	0.003	-0.103	-0.002	0.007	0.115	-0.04	0.006
T1C wav LHH glszm SALGLE	-0.526	-0.49	-0.126	-0.074	-0.002	-0.032	-0.084	0.002	-0.075	-0.043	0.059	0.004	-0.061	0.243	0.006	0.039	0.062	-0.032	0.026	-0.091	-0.041	-0.028	-0.047	0.021	0.011	0.083	0.117	-0.035	0.134	-0.025	-0.049	0.041
T1C wav LHH glszm ZoneEntropy	0.456	0.555	-0.117	0.026	-0.136	-0.042	0.126	0.013	0.206	0.072	-0.043	0.044	-0.08	-0.178	-0.051	-0.073	-0.008	-0.007	-0.008	-0.037	-0.02	0.109	-0.026	0.006	-0.001	0.01	-0.069	0.019	0.013	-0.061	-0.013	-0.051
T1C wav LHH glszm GLNUN	-0.16	-0.707	-0.089	0.002	0.19	0.066	-0.103	0.03	-0.049	-0.038	0.005	-0.004	0.146	0.246	0.062	0.019	0.005	-0.037	-0.02	-0.084	0.003	-0.069	0.048	-0.042	-0.031	0.002	0.013	0.017	0.013	-0.028	-0.004	0.015
T1C wav LHH glszm LAHGLE	0.156	-0.336	-0.25	0.082	0.671	0.043	-0.029	-0.021	0.08	-0.032	-0.007	0.055	-0.024	0.033	0.015	0.027	0.02	0.043	-0.023	0	0.003	0.033	0.021	0.001	0.003	-0.045	0.029	0.013	-0.045	0.052	0.009	0.028
T1C wav LHH glszm GrayLevelVariance	0.129	0.712	0.064	0.009	-0.014	-0.041	0.212	0.086	-0.032	0.131	0.002	0.044	0.042	-0.047	0.003	-0.003	-0.059	0.155	0.016	0.016	0.041	0.054	-0.049	-0.021	0.12	0.062	-0.082	0.019	0.022	0.104	0.066	-0.056
T1C wav LHH glszm SZNUN	-0.009	0.45	0.408	0.013	0.049	-0.04	0.028	0.048	-0.11	0.064	0.062	-0.114	0.073	0.004	-0.025	0.085	-0.077	0.158	0.055	0.24	0.089	-0.133	-0.019	-0.068	0.085	0.084	-0.013	0.016	-0.077	0.242	0.053	0.077
T1C wav HLL glrlm LRHGLE	0.129	0.315	-0.005	-0.007	0.054	0.042	0.201	0.662	0.072	-0.013	0.038	-0.061	0.047	-0.014	0.011	-0.063	0.043	-0.041	-0.227	0.015	-0.002	0.001	-0.045	0.042	0.036	0.026	0.023	0.056	0.032	0.037	-0.101	-0.031
T1C wav HLL gldm SDLGLE	-0.797	-0.076	0.022	0.073	-0.045	-0.015	-0.027	-0.037	-0.029	-0.043	-0.031	-0.007	-0.026	0.013	0.009	0.029	-0.008	0.055	0.054	0.134	-0.028	-0.035	0.039	-0.051	-0.008	-0.014	0.059	-0.038	-0.008	0.047	0.011	-0.024
T1C wav HLL gldm DependenceEntropy	0.67	0.3	-0.278	-0.025	0.039	-0.049	-0.035	0.209	0.062	0.048	0.012	0.03	0.015	0.036	-0.005	-0.081	0.016	-0.019	0.005	0.018	0.056	0.001	-0.053	-0.019	-0.003	-0.01	-0.066	-0.025	0.025	-0.019	-0.027	-0.011
T1C wav HLL gldm LDLGLE	-0.697	-0.232	-0.062	0.041	-0.024	0.082	-0.045	-0.043	-0.048	-0.023	-0.085	-0.042	-0.112	0.015	0.081	0.015	-0.05	0.051	0.175	0.102	-0.059	-0.052	-0.011	-0.046	0.02	0.022	0.017	-0.093	-0.034	0.074	-0.142	0.002
T1C wav HLL gldm DependenceVariance	0.231	-0.492	-0.404	0.033	0.366	0.129	-0.05	-0.004	-0.04	0.064	-0.035	-0.026	0.16	0.038	0.029	0.037	-0.012	0.043	0.054	-0.021	-0.006	-0.003	-0.02	0.002	0.027	0.136	-0.045	-0.002	0.036	0.083	0.023	0.014
T1C wav HLL glcm Idn	0.522	-0.187	-0.276	0.047	0.132	0.064	0.234	0.219	0.135	0.093	0.008	-0.095	0.121	-0.088	-0.056	-0.029	0.055	-0.09	0.075	0.056	0.053	0.071	-0.147	0.114	0.056	0.12	0.028	0.037	0.098	0.109	-0.068	0.001
T1C wav HLL glcm ClusterShade	-0.08	-0.189	0.073	0.016	0.011	-0.195	0.012	-0.36	-0.015	0.023	-0.17	0.116	-0.011	-0.023	0.028	0.028	-0.005	0.036	0.608	0.009	0.015	0.087	0.021	0.028	-0.018	0.075	-0.01	-0.024	0.016	-0.054	0.132	0.046
T1C wav HLL glcm InverseVariance	0.03	-0.667	-0.269	0.1	0.195	0.157	0.004	-0.053	0.102	0.123	-0.033	-0.003	0.065	0.03	0.002	0.021	-0.025	-0.018	0.013	-0.03	-0.043	0.065	-0.029	0.079	0.039	0.15	-0.018	0.004	0.063	0.111	0.045	-0.044
T1C wav HLL glcm SumEntropy	0.469	0.601	-0.021	-0.095	-0.106	-0.102	0.009	0.189	-0.018	-0.015	0.014	-0.005	-0.039	0.026	-0.026	-0.083	0.031	-0.048	-0.012	-0.013	0.044	-0.077	-0.039	-0.038	-0.029	-0.076	-0.056	-0.011	0.04	0.004	-0.022	0.055
T1C wav HLL glcm ClusterProminence	0.023	0.231	0.031	-0.004	0.045	-0.011	0.032	0.766	0.012	0.043	-0.019	0.07	-0.045	0.009	-0.02	-0.001	-0.04	0	0.045	-0.027	0.011	-0.035	-0.053	0.021	0.045	0.018	0.006	-0.038	-0.036	-0.076	0.076	0.01
T1C wav HLL glcm DifferenceVariance	0.065	0.374	0.074	-0.022	0.007	0.004	0.016	0.706	-0.047	0.045	0.036	0.069	-0.033	-0.019	-0.013	-0.028	-0.019	-0.031	-0.012	-0.027	0.057	-0.075	-0.083	-0.059	0.009	-0.064	-0.033	-0.046	-0.029	-0.041	0.044	0.027
T1C wav HLL firstorder RootMeanSquared	0.129	0.308	0.321	-0.179	0.032	-0.099	0.164	0.46	0.002	0.044	0.001	-0.019	-0.122	0.001	-0.071	-0.138	0.071	0.082	0.143	0.003	0.118	-0.129	0.126	-0.16	0.024	-0.084	0.006	0.002	0.067	0.156	0.09	-0.038
T1C wav HLL firstorder Kurtosis	0.199	-0.224	-0.169	0.124	0.269	0.139	0.408	0.268	-0.013	0.036	-0.006	-0.203	0.18	-0.123	-0.073	-0.05	0.082	-0.08	0.052	0.074	-0.046	0.095	-0.129	0.082	-0.014	0.028	0.067	0.06	0.092	0.175	-0.13	-0.023
T1C wav HLL firstorder Maximum	0.174	0.389	0.036	0.003	-0.048	-0.054	0.21	0.546	-0.017	0.03	-0.048	-0.05	0.05	-0.091	-0.028	-0.054	0.018	-0.036	0.287	0.088	0.057	-0.046	-0.079	-0.099	0.002	-0.068	0.012	0.028	-0.016	0.006	-0.031	0.069
T1C wav HLL firstorder 10Percentile	-0.025	-0.584	-0.069	0.045	0.089	0.004	0.11	-0.305	0.198	-0.011	-0.115	-0.08	0.022	0.025	-0.02	-0.032	0.026	0.041	0.099	-0.003	-0.1	0.122	0.087	0.193	0.07	0.219	0.076	0.048	0.042	0.052	-0.039	-0.024
T1C wav HLL firstorder Skewness	-0.099	0.108	0.151	-0.035	-0.197	-0.205	0.149	-0.011	-0.055	-0.066	-0.14	-0.004	0.025	-0.041	0.115	-0.061	-0.046	-0.022	0.674	0.113	-0.012	-0.039	-0.038	-0.12	-0.001	-0.009	0.012	0.043	-0.015	0.048	0.001	-0.007
T1C wav HLL firstorder Range	0.227	0.425	0.007	0.003	0.012	-0.004	0.237	0.599	0.035	0.017	0.02	-0.046	0.07	-0.063	-0.022	-0.06	0.041	-0.07	-0.025	0.036	0.063	-0.033	-0.088	-0.02	0.02	-0.016	0.00E+00	0.033	0.018	0.011	-0.068	0.027

T1C feature name	T1C F1	T1C F2	T1C F3	T1C F4	T1C F5	T1C F6	T1C F7	T1C F8	T1C F9	T1C F10	T1C F11	T1C F12	T1C F13	T1C F14	T1C F15	T1C F16	T1C F17	T1C F18	T1C F19	T1C F20	T1C F21	T1C F22	T1C F23	T1C F24	T1C F25	T1C F26	T1C F27	T1C F28	T1C F29	T1C F30	T1C F31	T1C F32
T1C wav HLL firstorder 90Percentile	0.073	0.627	0.247	-0.173	-0.064	-0.128	0.008	0.257	-0.113	0.001	0.025	0.063	-0.083	-0.023	-0.004	-0.103	0.042	0.041	-0.014	-0.017	0.095	-0.138	0.035	-0.144	-0.037	-0.133	-0.1	-0.014	0.002	-0.001	0.013	0.019
T1C wav HLL firstorder Median	0.069	-0.121	0.329	-0.138	0.043	0.146	0.151	-0.139	0.103	0.035	0.137	-0.137	-0.107	0.151	-0.175	-0.025	0.094	0.104	-0.157	-0.111	0.25	-0.061	0.186	-0.227	-0.055	-0.096	0.075	0.034	0.103	0.27	0.07	-0.077
T1C wav HLL ngtdm Complexity	0.08	0.331	0.017	-0.001	0.028	-0.031	0.031	0.73	0.05	0.054	-0.009	0.085	-0.033	0.004	-0.012	-0.008	-0.038	-0.02	0.016	-0.039	0.03	-0.06	-0.05	-0.029	0.023	-0.004	-0.016	-0.062	-0.031	-0.073	0.064	0.028
T1C wav HLL ngtdm Strength	-0.2	0.318	0.157	-0.032	-0.03	0.017	0.052	0.546	-0.351	0.004	0.016	-0.027	0.023	-0.101	-0.074	0.032	-0.01	0.017	0.035	0.118	0.091	-0.098	-0.142	-0.043	-0.021	-0.144	-0.015	-0.008	-0.044	-0.003	0.007	0.015
T1C wav HLL ngtdm Busyness	0.112	-0.441	-0.197	0.127	0.198	-0.051	-0.137	-0.157	0.406	-0.025	-0.077	0.134	-0.167	0.042	0.125	0.021	-0.082	0.086	0.048	-0.025	-0.075	0.095	0.12	-0.038	-0.032	0.035	0.034	0.051	-0.111	0.089	0.02	-0.033
T1C wav HLL ngtdm Contrast	-0.435	0.393	0.254	-0.061	-0.052	-0.077	-0.056	0.179	-0.347	-0.092	0.035	0.062	-0.04	-0.043	0.012	0.065	-0.018	0.093	-0.003	0.039	0.128	-0.057	-0.03	-0.129	-0.061	-0.205	-0.053	-0.008	-0.017	-0.011	0.038	-0.02
T1C wav HLL glszm LAHGLE	0.171	-0.083	-0.215	0.041	0.663	0.074	0.106	0.32	0.061	0.018	0.036	-0.084	0.065	-0.055	-0.037	-0.018	0.008	-0.024	-0.127	-0.01	-0.014	0.00E+00	-0.027	0.021	0.021	0.045	0.035	0.047	0.077	0.001	-0.05	-0.029
T1C wav LHL glrlm LRHGLE	0.155	0.488	0.006	0.049	-0.024	0.04	0.037	0.531	0.157	-0.088	0.01	-0.005	0.115	0.052	0.119	-0.05	-0.027	0.042	-0.087	0.005	-0.036	0.087	0.123	0.048	-0.019	0.013	0.001	0.061	0.081	0.036	-0.085	-0.005
T1C wav LHL gldm SDLGLE	-0.808	-0.103	0.012	0.037	-0.015	0.036	-0.007	-0.036	0.038	0.03	0.006	0.026	-0.04	-0.021	-0.003	-0.015	0.041	-0.015	-0.026	-0.037	-0.009	-0.064	0.042	-0.011	0.029	0.06	0.012	0.005	-0.069	0.041	0.009	0.031
T1C wav LHL gldm LDHGLE	0.274	0.291	-0.201	0.082	-0.035	0.1	-0.007	0.444	0.267	-0.104	0.029	0.009	0.19	0.014	0.164	-0.024	0.009	0.042	-0.067	0.036	-0.032	0.114	0.165	0.06	-0.049	0.043	-0.029	0.076	0.13	0.048	-0.095	-0.032
T1C wav LHL glcm Idn	0.575	-0.105	-0.2	0.047	0.003	0.096	0.05	0.135	0.298	0.058	0.018	0.003	0.248	0.02	0.143	-0.083	-0.023	0.055	0.053	0.01	-0.026	0.03	0.187	-0.046	-0.031	0.005	-0.021	-0.057	0.105	-0.02	-0.04	-0.04
T1C wav LHL glcm ClusterShade	-0.061	-0.298	0.049	0.019	-0.025	-0.046	0.046	-0.723	-0.043	0.025	0.052	-0.018	-0.021	-0.047	0.03	-0.039	-0.085	0.019	0.004	0.057	0.098	-0.096	-0.067	-0.061	0.035	-0.042	-0.023	-0.01	-0.041	-0.076	-0.008	0.039
T1C wav LHL glcm SumEntropy	0.402	0.651	0.023	0.009	-0.121	-0.091	0.053	0.118	-0.003	-0.16	-0.069	0.038	-0.107	0.069	0.029	-0.02	0.023	-0.039	0.045	-0.016	-0.014	0.026	-0.038	0.043	-0.01	0.01	-0.031	0.037	0.05	0.05	-0.011	0.041
T1C wav LHL glcm DifferenceVariance	0.027	0.615	0.118	0.082	-0.005	0.017	0.044	0.411	-0.137	-0.144	-0.127	0.089	0.032	0.119	0.065	-0.041	-0.034	0.044	-0.038	-0.061	-0.055	0.006	0.064	0.035	-0.025	-0.016	-0.086	0.015	0.038	0.006	0.025	-0.035
T1C wav LHL firstorder RootMeanSquared	0.045	0.252	0.534	0.166	0.037	0.009	0.091	0.032	-0.229	-0.049	-0.046	-0.069	0.041	0.053	-0.128	-0.099	-0.055	0.064	0.056	0.235	0.14	-0.023	0.089	-0.011	0.018	-0.053	-0.069	0.199	0.079	0.029	0.14	0.131
T1C wav LHL firstorder Kurtosis	0.293	-0.235	-0.12	0.097	-0.059	0.228	0.026	0.246	0.071	0.029	0.033	0.064	0.442	-0.02	0.174	0.005	0.022	0.078	0.045	-0.048	-0.034	-0.046	0.19	-0.035	-0.047	0.053	-0.061	-0.021	0.253	-0.006	-0.015	-0.048
T1C wav LHL firstorder Maximum	0.225	0.617	0.137	0.115	-0.068	-0.036	0.156	0.139	0.018	-0.076	-0.127	0.123	0.008	0.147	0.095	-0.12	-0.045	0.058	0.079	-0.024	0.011	-0.131	0.21	0.031	0.023	-0.041	-0.078	0.011	0.014	-0.072	-0.047	0.009
T1C wav LHL firstorder 10Percentile	-0.017	-0.701	-0.041	0	0.068	0.093	-0.016	-0.189	0.149	0.23	0.105	-0.049	0.066	-0.087	-0.025	-0.041	-0.016	0.001	0.007	0.135	0.019	-0.025	0.015	-0.096	0.056	-0.009	0.069	-0.02	0.039	-0.048	0.031	0.025
T1C wav LHL firstorder Mean	0.051	-0.222	0.497	0.141	0.046	0.051	0.07	-0.2	-0.119	0.086	0.062	-0.12	0.049	-0.024	-0.154	-0.109	-0.032	0.036	0.075	0.306	0.136	-0.023	0.069	-0.031	0.044	-0.054	0.003	0.189	0.079	0.008	0.131	0.163
T1C wav LHL firstorder InterquartileRange	-0.133	0.656	0.147	0.032	-0.056	-0.148	0.022	0.14	-0.172	-0.232	-0.155	0.047	-0.092	0.062	-0.018	0.062	-0.037	0.053	0.014	-0.041	0.096	-0.047	-0.005	0.041	-0.018	-0.01	-0.106	0.094	-0.031	0.068	0.002	0.04
T1C wav LHL firstorder Median	0.287	-0.083	0.452	0.094	0.063	0.166	0.064	-0.002	-0.165	0.039	0.026	-0.202	0.077	0.079	-0.2	0.02	0.067	0.031	0.04	0.241	0.16	0.051	0.116	0.053	-0.027	-0.04	0.062	0.177	0.122	0.159	0.049	0.089
T1C wav LHL ngtdm Strength	-0.377	0.423	0.214	0.039	-0.04	-0.029	-0.006	0.126	-0.383	-0.201	-0.15	0.01	0.067	0.026	0.086	0.151	-0.068	0.16	0.018	0.069	-0.033	0.011	0.037	0.06	-0.007	-0.067	-0.059	0.041	0.053	-0.016	0.035	-0.007
T1C wav LHL ngtdm Busyness	0.149	-0.41	-0.241	0.037	0.567	-0.008	-0.015	-0.072	0.145	0.168	-0.025	-0.059	-0.082	-0.06	-0.1	-0.024	-0.029	0.023	-0.069	-0.006	0.008	0.008	-0.037	-0.012	0.033	-0.038	0.026	-0.009	-0.106	0.03	0.001	0.004
T1C wav LHL glszm ZoneVariance	0.095	-0.32	-0.21	0.037	0.718	0.008	-0.053	0.004	-0.018	0.02	0.021	-0.097	0.041	0.001	0.012	-0.015	-0.018	0.00E+00	-0.01	-0.015	0.011	-0.005	0.008	-0.027	0.008	0.011	-0.03	-0.022	0.016	-0.001	0.019	-0.011
T1C wav LHL glszm ZoneEntropy	0.619	0.369	-0.251	0.073	0.052	-0.036	0.046	0.124	0.071	-0.121	-0.092	0.048	-0.05	0.085	0.039	0.008	0.011	0.009	0.023	-0.003	0.017	0.128	0.036	0.06	-0.024	0.01	-0.122	0.057	0.028	0.043	0.027	0.033
T1C wav LHL glszm LALGLE	-0.243	-0.379	-0.192	0.023	0.636	-0.008	-0.052	0	-0.022	0.081	0.009	-0.095	-0.038	0.034	0.017	-0.055	-0.002	-0.01	-0.058	-0.031	0.01	0.015	0.014	-0.02	0.038	0.013	-0.03	-0.029	0.002	0.005	0.024	0.008
T1C wav LHL glszm LAHGLE	0.283	-0.002	-0.305	0.086	0.432	0.101	-0.045	0.35	0.196	-0.081	0.028	-0.029	0.211	-0.006	0.138	-0.008	-0.01	0.042	-0.037	0.013	-0.008	0.062	0.14	0.026	-0.035	0.062	-0.061	0.053	0.123	0.041	-0.048	-0.034
T1C wav LHL glszm SZNUN	-0.186	0.568	0.49	0.015	-0.142	-0.077	0.023	0.037	-0.089	-0.126	-0.013	0.052	-0.046	0.024	-0.008	-0.058	-0.043	0.019	-0.037	-0.005	0.047	-0.14	-0.037	0.032	0.039	-0.019	0.039	0.001	0.012	-0.001	-0.036	0.019
T1C wav HHH glrlm SRLGLE	-0.508	-0.5	-0.024	-0.006	0.149	0.029	-0.148	0.033	-0.048	-0.052	-0.001	0.018	0.075	0.194	0.034	-0.006	-0.117	-0.127	-0.036	-0.018	0.005	-0.025	0.035	-0.084	0.009	0.006	0.073	0.059	0.131	-0.125	0.043	-0.044
T1C wav HHH gldm SDLGLE	-0.661	-0.281	0.165	0.028	-0.023	0.068	-0.136	-0.004	-0.113	-0.072	-0.034	0.038	0.034	0.078	0.005	0.001	-0.143	-0.101	-0.03	0.098	0	-0.029	0.052	-0.066	-0.007	-0.059	0.093	0.047	0.059	-0.162	0.052	-0.074
T1C wav HHH gldm DependenceEntropy	0.543	0.472	-0.215	0.06	-0.042	-0.023	0.187	-0.035	0.079	0.155	-0.041	0.008	-0.051	-0.099	-0.117	-0.016	-0.025	0.072	-0.022	0.025	0.088	0.026	-0.019	-0.001	0.023	0.066	-0.012	-0.042	0.078	0.009	-0.021	-0.013

T1C feature name	T1C F1	T1C F2	T1C F3	T1C F4	T1C F5	T1C F6	T1C F7	T1C F8	T1C F9	T1C F10	T1C F11	T1C F12	T1C F13	T1C F14	T1C F15	T1C F16	T1C F17	T1C F18	T1C F19	T1C F20	T1C F21	T1C F22	T1C F23	T1C F24	T1C F25	T1C F26	T1C F27	T1C F28	T1C F29	T1C F30	T1C F31	T1C F32
T1C wav HHH gldm LDHGLE	0.209	0.077	-0.159	0.071	-0.038	0.11	0.724	-0.028	0.095	0.094	-0.011	-0.057	-0.022	-0.066	-0.01	-0.011	0.023	-0.007	-0.072	0.034	-0.037	0.076	-0.037	0.032	0.053	0.019	-0.01	0.027	-0.044	0.032	-0.156	-0.01
T1C wav HHH gldm DependenceVariance	0.193	-0.577	-0.399	-0.013	0.282	0.03	-0.037	-0.006	-0.008	-0.034	-0.01	0.045	0.005	0.24	0.014	0.026	0.046	0.036	-0.042	0.036	0.006	0.045	-0.012	0.022	-0.005	0.042	-0.043	0.078	-0.029	0.03	0.014	-0.006
T1C wav HHH glcm Idmn	0.682	-0.046	-0.06	0.001	-0.024	0.064	0.169	-0.026	0.168	-0.041	0.152	-0.037	-0.036	0.014	-0.044	-0.044	0.21	0.002	0.016	0.073	-0.063	0.04	-0.038	0.082	-0.072	0.066	-0.114	-0.031	-0.088	0.115	-0.104	0.073
T1C wav HHH glcm ClusterShade	-0.051	0	-0.014	-0.058	0.018	0.046	-0.744	0.046	0.05	-0.111	0.072	0.009	-0.026	0.094	0.001	-0.065	0.061	0.031	0.063	0.077	-0.204	-0.031	0.065	-0.055	-0.034	0.018	-0.033	-0.076	0.036	0.003	0.089	0.065
T1C wav HHH glcm Correlation	0.191	-0.097	0.055	0.024	-0.017	-0.112	0.141	0.084	0.023	-0.035	0.131	0.079	0.066	0.036	-0.067	0.013	0.114	0	0	0.086	-0.183	0.065	-0.048	0.668	0.027	0.013	0.058	-0.006	0.058	0.095	-0.039	0.003
T1C wav HHH glcm Imc1	0.615	-0.172	-0.321	-0.026	0.013	0.004	-0.188	0.098	0.225	-0.039	0.035	0.029	0.003	0.043	0.069	-0.093	0.02	-0.197	0.025	-0.066	-0.105	-0.002	-0.016	0	-0.023	0.032	0.027	-0.002	0.01	0.003	-0.008	-0.03
T1C wav HHH glcm MaximumProbability	-0.076	-0.696	-0.144	0.004	0.202	0.085	-0.069	0.029	-0.075	-0.044	0.034	0.022	0.089	0.303	0.007	0.055	0.012	-0.02	-0.043	0.015	-0.014	0.015	0.014	-0.001	-0.03	0.011	-0.023	0.086	-0.028	-0.044	-0.012	-0.032
T1C wav HHH firstorder RootMeanSquared	-0.193	0.308	0.072	-0.002	0.033	-0.147	-0.072	-0.098	-0.019	0.149	-0.091	-0.025	0.057	-0.008	-0.093	0.155	-0.002	0.642	0.005	0.001	-0.033	-0.007	-0.015	-0.043	0.041	0.041	-0.026	-0.039	0.027	0.032	0.057	0.02
T1C wav HHH firstorder Kurtosis	0.238	-0.075	0.088	0.069	-0.064	0.207	0.559	-0.018	0.008	-0.042	0.119	-0.087	-0.079	0.035	0.029	-0.046	0.211	-0.052	0.036	0.019	-0.146	0.027	-0.133	0.163	-0.02	0.053	-0.044	-0.004	-0.055	0.126	-0.259	0.087
T1C wav HHH firstorder Maximum	0.204	0.459	0.252	0.149	-0.066	-0.071	0.427	-0.053	0.097	0.116	0.129	-0.104	-0.034	-0.04	-0.03	-0.08	0.048	0.032	0.018	0.067	0.012	0.049	-0.019	-0.059	-0.026	0.067	-0.117	-0.067	0.067	0.065	0.119	0.06
T1C wav HHH firstorder Mean	-0.237	-0.083	-0.076	-0.073	0.045	-0.081	-0.389	-0.092	0.062	0.003	-0.112	0.027	0.089	0.007	-0.071	0.148	0.046	0.549	0.004	-0.017	-0.158	-0.02	-0.004	-0.005	0.01	-0.016	0.036	0.005	-0.042	0	0.025	0.016
T1C wav HHH firstorder Skewness	-0.159	-0.029	0.008	-0.008	0.021	-0.26	-0.256	-0.021	0.028	0.007	0.029	0.016	0.12	0.019	-0.017	-0.103	-0.097	0.055	0.138	0.052	-0.082	0.002	0.135	-0.168	-0.111	-0.01	0.007	0.043	-0.001	0.035	0.591	-0.019
T1C wav HHH firstorder Median	0.271	-0.127	0.162	0.09	0.019	0.01	-0.126	0.004	-0.202	-0.073	-0.011	-0.01	0.07	-0.024	-0.108	0.179	-0.021	0.341	-0.099	0.26	-0.091	0.048	-0.034	0.058	0.035	-0.057	-0.059	0.087	0.029	-0.28	-0.122	0.142
T1C wav HHH ngtdm Strength	-0.245	0.284	0.25	0.045	-0.03	-0.045	0.498	-0.107	-0.26	0.034	0.032	-0.11	-0.099	-0.072	-0.028	0.111	0.065	0.253	0.054	0.147	0.024	-0.021	-0.034	-0.033	0.01	0.063	-0.091	-0.011	0.016	0.056	-0.01	0.065
T1C wav HHH ngtdm Contrast	-0.317	0.426	0.222	0.008	0.013	-0.179	0.057	-0.013	-0.266	0.088	-0.076	-0.014	-0.014	-0.04	-0.035	0.147	-0.125	0.315	-0.008	0.062	0.261	0.008	-0.029	-0.063	0.013	0.009	-0.032	-0.004	0.076	0.01	-0.002	-0.017
T1C wav HHH glszm ZoneVariance	0.126	-0.343	-0.233	0.04	0.699	0.006	-0.056	-0.013	0.044	-0.041	0.01	0.002	-0.023	0.075	0.024	0.015	0.021	0.015	-0.011	0.004	0.006	0.023	0.007	0.005	0.008	-0.031	-0.027	0.006	-0.038	0.015	0.001	0.012
T1C wav HHH glszm ZoneEntropy	0.409	0.627	0.081	0.062	-0.132	-0.003	0.173	-0.025	0.093	0.093	-0.015	-0.013	-0.026	-0.19	-0.06	-0.022	-0.007	0.022	-0.003	-0.034	0.031	0	0.002	-0.046	-0.008	0.015	0.037	-0.021	0.071	-0.042	-0.019	0.002
T1C wav HHH glszm GrayLevelVariance	0.089	0.494	0.073	0.125	-0.046	-0.041	0.536	-0.078	-0.075	0.198	0.024	-0.06	-0.058	-0.051	-0.055	0.007	-0.04	0.126	0.017	0.044	0.098	0.032	-0.023	-0.028	0.026	0.09	-0.077	-0.049	0.075	0.022	0.05	-0.009
T1C wav LLH glrlm SRLGLE	-0.543	-0.383	0.046	0.023	-0.055	0.107	-0.022	0.013	-0.027	-0.061	0.019	0.149	-0.104	0.079	0.096	-0.016	0.018	-0.032	-0.042	-0.054	0.046	-0.006	0.011	0.051	-0.075	0.076	0.004	-0.027	0.375	-0.021	-0.005	0.077
T1C wav LLH glrlm LRHGLE	0.119	0.577	-0.227	-0.062	-0.021	-0.05	-0.022	0.004	0.042	0.433	-0.068	0.025	0.149	0.025	-0.064	0.006	-0.019	0.014	-0.076	-0.014	-0.112	0.047	0.068	0.033	0.043	0.02	0.055	0.059	-0.052	-0.011	0.003	-0.032
T1C wav LLH gldm SDLGLE	-0.775	-0.152	0.073	0.044	-0.076	0.071	-0.003	-0.01	0.013	-0.063	0.045	0.054	-0.06	-0.005	0.029	-0.006	0.044	-0.055	-0.024	-0.005	0.068	-0.059	0.01	0.04	-0.059	0.057	-0.012	-0.03	0.112	0.004	-0.003	0.054
T1C wav LLH gldm DependenceEntropy	0.591	0.294	-0.447	0.018	0.06	-0.08	0.011	0.025	0.143	0.115	-0.032	0.03	0.006	0	0.017	-0.049	0.007	0.026	0.004	0.028	0.013	0.04	0.01	0.03	-0.009	0.047	-0.059	-0.009	-0.042	0.003	-0.018	-0.023
T1C wav LLH gldm LDLGLE	0.013	-0.556	-0.115	0.055	0.198	0.152	-0.044	0.049	-0.089	-0.031	-0.019	0.176	0.015	0.127	0.127	0.017	0.042	0.036	-0.04	-0.133	0.007	-0.008	0.024	0.019	-0.066	0.068	-0.005	-0.005	0.413	0.023	0.02	0.076
T1C wav LLH gldm LDHGLE	0.242	0.063	-0.502	0.036	0.095	0.005	-0.017	-0.048	0.022	0.419	-0.093	-0.104	0.156	0.143	-0.268	-0.046	-0.03	0.068	-0.097	-0.014	-0.058	0.034	0.004	0.022	0.046	-0.027	0.036	0.105	-0.123	-0.014	0.012	-0.007
T1C wav LLH gldm DependenceVariance	0.197	-0.552	-0.324	0.077	0.306	0.061	-0.041	0.03	-0.061	0.02	-0.008	-0.05	0.193	0.249	-0.024	-0.003	0.009	0.065	0.015	-0.078	0.025	-0.085	0.007	-0.063	-0.021	0.025	-0.041	0.038	-0.039	0.07	0.014	0.051
T1C wav LLH glcm Idn	0.576	-0.225	-0.214	0.061	0.127	0.171	-0.004	-0.068	0.044	0.109	-0.104	0.011	0.151	0.236	-0.071	-0.049	-0.091	0.049	-0.117	-0.004	-0.002	0.062	0.021	-0.151	0.027	-0.108	0.061	0.121	-0.029	0.003	0.112	0.005
T1C wav LLH glcm ClusterShade	-0.214	0.101	0.069	-0.134	0.089	-0.044	0.038	-0.097	0.105	0.099	0.049	0.08	0.182	0.08	0.635	0.222	-0.022	-0.096	0.059	0.009	0.058	-0.008	0.032	-0.002	-0.101	-0.017	0.057	0.094	0.001	0.024	-0.062	0.047
T1C wav LLH glcm Correlation	0.194	-0.294	-0.335	-0.009	0.089	0.056	-0.065	-0.058	-0.088	-0.295	-0.03	0.089	0.036	0.355	-0.109	0.039	-0.07	-0.208	-0.141	-0.007	0.039	-0.01	0.095	0.102	-0.01	-0.141	0.086	0.018	0.075	0.023	0.051	-0.083
T1C wav LLH glcm MaximumProbability	-0.194	-0.597	-0.042	0.084	0.152	0.164	-0.071	0.023	-0.137	-0.033	0.015	0.014	0.277	0.3	-0.07	0.052	-0.043	0	0	-0.062	0.018	-0.135	0.019	-0.072	-0.033	-0.016	0.019	0.059	-0.003	-0.01	-0.007	0.057

T1C feature name	T1C F1	T1C F2	T1C F3	T1C F4	T1C F5	T1C F6	T1C F7	T1C F8	T1C F9	T1C F10	T1C F11	T1C F12	T1C F13	T1C F14	T1C F15	T1C F16	T1C F17	T1C F18	T1C F19	T1C F20	T1C F21	T1C F22	T1C F23	T1C F24	T1C F25	T1C F26	T1C F27	T1C F28	T1C F29	T1C F30	T1C F31	T1C F32
T1C wav LLH firstorder Maximum	0.127	0.645	-0.086	-0.042	0.002	-0.012	0.051	-0.043	0.146	0.334	-0.011	0.085	0.096	0.042	0.208	0.022	-0.016	0.049	-0.042	0.023	0.026	0.077	0.014	-0.047	0.029	0.02	-0.035	0.065	-0.015	0.012	0.062	0.019
T1C wav LLH firstorder Mean	0.338	-0.481	-0.058	-0.007	0.14	0.223	0.005	-0.162	0.058	0.18	-0.187	-0.108	0.084	0.064	-0.052	-0.01	-0.034	0.071	0.007	0.062	0.048	-0.175	-0.036	-0.198	0.116	-0.094	0.108	0.059	0.052	-0.061	-0.046	0.072
T1C wav LLH firstorder Skewness	-0.141	0.11	0.194	-0.128	-0.037	0.069	0.124	-0.063	0.148	-0.043	-0.044	0.327	-0.08	-0.162	0.544	0.052	0	-0.04	0.012	-0.02	0.029	0.108	-0.043	-0.057	-0.03	-0.094	-0.04	0.038	0.133	-0.031	0.014	-0.097
T1C wav LLH firstorder InterquartileRange	-0.299	0.669	-0.027	-0.093	-0.057	-0.211	0.013	0.106	0.001	0.055	0.005	0.103	-0.012	-0.121	0.098	0.044	-0.002	0.01	0.023	0.006	-0.032	0.027	-0.006	0.037	0.026	0.113	-0.096	-0.106	-0.031	0.029	-0.022	-0.065
T1C wav LLH firstorder 90Percentile	-0.153	0.67	-0.074	-0.15	-0.027	-0.169	-0.021	0.005	0.014	0.24	-0.064	0.052	0.099	-0.128	0.149	0.054	-0.025	0.013	0.01	-0.031	0.005	-0.09	-0.02	0.034	0.109	0.007	-0.058	-0.025	-0.047	-0.009	-0.018	-0.022
T1C wav LLH firstorder Median	0.455	-0.367	-0.12	0.093	0.116	0.282	-0.007	-0.115	-0.003	0.143	-0.069	-0.15	0.017	0.142	-0.174	0.006	0.023	0.082	-0.003	0.168	0.019	-0.132	0.007	-0.129	0.051	-0.014	0.111	0.003	0.055	-0.07	-0.013	0.123
T1C wav LLH ngtdm Busyness	0.292	-0.494	-0.082	0.135	0.366	0.056	-0.021	0.014	0.263	-0.099	-0.027	0.091	-0.131	-0.068	0.123	0.066	-0.048	-0.006	0.004	-0.067	-0.011	-0.001	0.007	-0.015	-0.083	0.094	0.015	-0.032	0.166	0.004	-0.043	-0.026
T1C wav LLH ngtdm Contrast	-0.695	0.325	0.027	-0.025	-0.026	-0.154	-0.007	0.027	-0.013	0.055	0.049	0.058	-0.028	-0.094	0.101	0.083	0.032	0.044	0.011	-0.065	-0.017	-0.048	-0.017	0.036	0.049	0.104	-0.093	-0.058	-0.044	0.048	-0.025	0.016
T1C wav LLH glszm SALGLE	-0.663	-0.334	-0.009	0.063	-0.049	0.14	-0.019	0.009	-0.053	-0.088	0.028	0.097	0.008	0.034	0.042	0.008	0.054	-0.043	-0.021	-0.021	0.07	-0.082	0.013	0.041	-0.082	0.059	0.004	-0.026	0.231	0.01	0.013	0.073
T1C wav LLH glszm LAHGLE	0.094	-0.255	-0.2	0.048	0.743	-0.001	-0.039	-0.012	-0.003	0.004	0.043	-0.076	0.049	0.061	-0.064	0.012	-0.01	-0.009	0.014	-0.035	-0.009	-0.057	-0.004	-0.023	0.003	-0.014	0.027	0.022	0.016	-0.019	-0.018	0.002
T1C wav LLH glszm GrayLevelVariance	-0.09	0.643	-0.168	-0.095	0.007	-0.086	-0.022	0.045	0.019	0.364	-0.01	0.084	0.127	0.029	0.159	0.103	0.001	-0.009	-0.009	0.016	-0.008	-0.001	0.063	0.078	-0.038	0.067	0.031	0.015	-0.036	-0.008	-0.002	0.019
T1C wav HLH glrlm SRLGLE	-0.707	-0.303	-0.023	0.098	-0.028	0.126	-0.047	0.007	0.113	-0.014	-0.043	0.105	-0.032	0.106	0.042	-0.024	-0.017	-0.093	-0.053	-0.079	0	0.005	0.086	-0.006	0.008	0.009	0.029	-0.016	0.048	0.058	0.009	0.017
T1C wav HLH gldm SDLGLE	-0.753	-0.189	0.107	0.125	-0.05	0.128	-0.058	-0.016	0.049	-0.014	-0.062	0.104	0.014	0.048	-0.006	0.002	-0.044	-0.059	-0.053	0	-0.022	0.017	0.078	-0.021	-0.011	-0.019	0.002	-0.012	-0.01	-0.006	-0.036	-0.032
T1C wav HLH gldm DependenceEntropy	0.545	0.411	-0.362	0.021	0.038	-0.072	0.048	0.016	0.101	0.215	-0.041	0.011	-0.022	-0.041	-0.038	0.003	-0.026	0.04	0.018	0.021	-0.014	0.054	-0.068	0.017	-0.001	0.05	-0.046	-0.037	0.056	0.026	-0.027	0.004
T1C wav HLH gldm LDLGLE	-0.302	-0.549	-0.195	0.043	0.145	0.046	-0.06	0.044	0.044	-0.004	-0.027	0.134	-0.07	0.348	0.103	-0.038	0.062	-0.033	-0.078	-0.121	0.024	0.028	0.032	0.025	0.029	-0.02	-0.003	0.019	0.152	0.083	0.02	0.086
T1C wav HLH gldm DependenceVariance	0.163	-0.552	-0.351	0.025	0.337	-0.022	-0.004	0.012	-0.019	-0.035	-0.012	0.034	0.024	0.33	0.007	-0.017	0.019	0.011	-0.014	-0.032	0.025	0.037	-0.022	0.023	0.027	-0.021	0.003	0.055	0.054	0.049	-0.023	0.036
T1C wav HLH glcm Idmn	0.723	-0.04	-0.047	-0.001	0.109	0.02	0.136	0.042	0.055	0.006	0.106	-0.103	0.04	0.107	-0.067	-0.015	0.018	0.023	0.069	-0.009	0.049	0.05	-0.058	0.057	-0.004	0.143	0.002	0.029	-0.085	-0.075	-0.037	-0.113
T1C wav HLH glcm ClusterShade	-0.018	-0.136	-0.144	0.045	-0.104	0.091	0.009	-0.231	-0.019	0.286	-0.088	-0.173	-0.068	-0.169	-0.517	-0.087	0.012	0.102	-0.153	-0.074	-0.11	-0.003	-0.145	0.036	0.009	-0.199	-0.025	0.029	0.064	0.019	-0.07	-0.058
T1C wav HLH glcm Correlation	0.115	0.178	-0.04	-0.15	-0.073	0.016	0.008	-0.177	-0.124	0.062	0.013	0.101	-0.035	0.078	0.016	0.14	-0.125	-0.02	-0.15	0.04	0.007	0.136	0.123	0.518	-0.136	-0.024	-0.057	-0.034	-0.079	-0.133	-0.083	-0.075
T1C wav HLH glcm Imc1	0.595	-0.222	-0.285	0.052	0.011	0.008	-0.024	0.047	0.267	-0.14	0.012	0.05	0.003	0.05	0.061	-0.153	-0.01	-0.203	-0.001	-0.116	-0.014	0.042	0.062	0.038	-0.02	0.096	0.006	-0.009	0.038	0.023	-0.003	-0.057
T1C wav HLH glcm MaximumProbability	-0.118	-0.665	-0.128	0.033	0.168	0.067	0.003	0.029	-0.053	-0.066	0.014	0.068	0.075	0.377	-0.001	0.03	-0.026	-0.046	-0.044	-0.053	0.023	0.008	-0.002	0.017	0.002	-0.027	0.041	0.084	0.087	-0.02	-0.032	0.015
T1C wav HLH glcm JointEntropy	0.241	0.703	0.041	-0.033	-0.122	-0.057	0.022	0.023	0.104	0.216	-0.009	-0.014	-0.014	-0.22	-0.012	-0.03	0.008	0.006	0.03	0.033	-0.022	-0.018	-0.038	-0.023	-0.011	0.01	-0.062	-0.071	0.003	0.014	0.013	0.025
T1C wav HLH firstorder RootMeanSquared	0.383	0.194	-0.071	0.133	-0.04	0.1	-0.018	0.059	0.054	0.159	-0.061	-0.02	0.055	-0.022	-0.053	0.006	-0.043	0.036	0.006	0.002	-0.012	-0.005	-0.635	-0.031	-0.125	-0.043	-0.025	-0.034	0.01	0.004	-0.036	0.032
T1C wav HLH firstorder Kurtosis	0.316	-0.132	-0.062	0.082	0.316	0.075	0.498	0.041	0.115	0.07	0.062	-0.156	0.031	0.139	-0.102	-0.025	0.036	-0.094	0.167	-0.036	-0.005	0.002	-0.136	-0.014	0.058	0.077	0.005	0.068	-0.049	-0.106	-0.064	-0.049
T1C wav HLH firstorder Maximum	0.247	0.547	0.032	0.054	-0.014	0.05	0.259	-0.035	0.169	0.382	0.003	-0.118	0.018	-0.046	-0.075	0.005	0.014	0.058	0.08	0.00E+00	-0.02	-0.015	-0.119	0.008	-0.008	0.053	-0.058	-0.012	0.018	-0.021	-0.008	-0.045
T1C wav HLH firstorder Mean	0.381	-0.095	-0.085	0.177	-0.039	0.118	-0.038	0.024	0.063	-0.068	-0.055	-0.029	0.035	-0.015	-0.061	-0.058	-0.038	-0.002	0.001	-0.032	-0.02	0.012	-0.656	-0.023	-0.109	-0.038	-0.031	-0.001	-0.002	-0.007	-0.049	-0.009
T1C wav HLH firstorder 90Percentile	-0.005	0.686	0.06	-0.045	-0.058	-0.061	-0.011	0.03	-0.013	0.343	-0.016	0.017	0.021	-0.112	0.002	0.128	-0.007	0.129	-0.012	0.049	-0.001	-0.04	-0.145	-0.058	-0.048	-0.013	-0.055	-0.078	0.009	0.04	0.014	0.075
T1C wav HLH firstorder Median	0.526	-0.065	-0.007	0.086	0.03	0.039	0.023	0.058	0.03	-0.061	-0.09	0.061	0.072	-0.002	0.099	-0.179	-0.126	0.028	0.067	0.09	0.118	0.053	-0.481	0.057	-0.058	0.08	0.05	0.049	-0.048	-0.019	-0.101	-0.003
T1C wav HLH ngtdm Strength	-0.282	0.436	0.182	-0.067	-0.013	-0.047	0.129	0.01	-0.282	0.288	0.008	-0.109	-0.073	-0.066	-0.078	0.169	0.047	0.306	0.065	0.113	0.053	-0.04	-0.102	-0.034	-0.035	0.029	-0.025	0.006	-0.02	0.002	0.009	0.039
T1C wav HLH ngtdm Contrast	-0.462	0.496	0.097	-0.055	-0.04	-0.125	-0.023	-0.035	-0.178	0.181	-0.044	0.078	0.001	-0.108	0.018	0.148	-0.005	0.165	-0.01	0.067	0.011	-0.045	-0.046	-0.108	-0.004	-0.061	-0.071	-0.089	0.045	0.087	0.024	0.082
T1C wav HLH glszm ZoneEntropy	0.497	0.538	-0.173	0.023	-0.04	-0.03	0.074	-0.005	0.183	0.138	0.005	-0.03	-0.053	-0.153	-0.105	0.021	-0.01	0.031	0.061	0.061	0.016	0.033	-0.062	0.033	-0.054	0.036	-0.02	-0.016	-0.006	-0.056	-0.024	-0.044

T1C feature name	T1C F1	T1C F2	T1C F3	T1C F4	T1C F5	T1C F6	T1C F7	T1C F8	T1C F9	T1C F10	T1C F11	T1C F12	T1C F13	T1C F14	T1C F15	T1C F16	T1C F17	T1C F18	T1C F19	T1C F20	T1C F21	T1C F22	T1C F23	T1C F24	T1C F25	T1C F26	T1C F27	T1C F28	T1C F29	T1C F30	T1C F31	T1C F32
T1C wav HLH glszm LALGLE	0.116	-0.413	-0.22	0.062	0.566	-0.003	-0.054	0.007	-0.027	-0.023	-0.009	0.05	-0.069	0.269	0.063	-0.019	0.093	0.036	-0.053	-0.031	0.016	0.031	-0.003	0.034	0.023	-0.105	-0.029	0.002	-0.017	0.052	0.002	0.069
T1C wav HLH glszm LAHGLE	0.123	-0.277	-0.23	0.042	0.733	0.01	-0.014	-0.027	0.06	-0.044	0.028	-0.006	-0.007	0.042	-0.032	0.022	-0.004	-0.002	0.011	-0.001	-0.003	0.007	-0.005	-0.011	0.005	-0.015	0.027	0.029	-0.025	-0.009	-0.012	0.015
T1C wav HLH glszm GrayLevelVariance	0.107	0.579	-0.098	-0.04	0	0.005	0.101	0.045	0.012	0.521	-0.037	-0.012	0.035	-0.038	-0.072	0.114	-0.018	0.084	0.007	0.087	0.028	0.001	-0.037	0.016	-0.047	-0.009	0.013	-0.041	0.026	0.002	0.008	0.052
T1C wav HLH glszm GLNU	0.45	0.07	-0.058	0.143	0.038	-0.056	0.004	0.065	0.659	-0.035	-0.028	-0.013	0.038	-0.056	0.039	-0.025	0.019	0.006	-0.023	-0.008	-0.022	-0.051	-0.01	-0.028	-0.018	0.006	-0.024	-0.01	-0.012	-0.013	-0.002	-0.018
T1C wav HLH glszm SZNUN	-0.094	0.447	0.385	-0.062	-0.041	-0.073	0.079	0.07	-0.059	0.396	0.003	0.012	0.05	-0.103	0.023	0.03	0.013	0.163	-0.129	0.067	0.006	0.01	0.016	-0.149	0.101	-0.008	-0.124	-0.011	0.014	0.118	-0.009	0.07
T1C wav HHL glrlm LRHGLE	0.161	0.267	0.122	0.142	-0.027	-0.114	0.673	0.084	0.063	-0.056	-0.014	0.078	0.008	0.039	0.025	-0.107	-0.102	-0.02	0.068	-0.032	0.049	-0.07	0.018	-0.044	0.005	-0.002	0.016	-0.05	0.035	-0.02	0.106	0.045
T1C wav HHL gldm SDLGLE	-0.8	-0.1	-0.025	0.027	-0.024	0.031	-0.022	-0.021	0.105	0.051	0.005	0.013	-0.042	0.003	0.01	0	0.052	0.017	-0.007	-0.017	-0.026	0	0.049	-0.057	0.046	0.077	-0.01	0.011	-0.056	0.048	-0.004	0.036
T1C wav HHL gldm DependenceEntropy	0.685	0.354	-0.148	0.08	0.03	-0.048	0.157	0.066	0.01	-0.108	-0.014	0.025	-0.037	0.072	-0.042	0.006	-0.006	0.018	-0.028	-0.011	0.04	0.021	0.011	0.084	-0.014	-0.002	-0.006	-0.006	0.089	-0.015	-0.025	0.046
T1C wav HHL gldm LowGrayLevelEmphasis	-0.781	-0.145	-0.065	0.024	0.008	0.037	-0.033	-0.013	0.111	0.066	-0.007	0.004	-0.053	0.018	0.031	-0.02	0.055	0.004	-0.025	-0.036	-0.016	0.014	0.05	-0.06	0.038	0.077	-0.041	0.002	-0.082	0.068	0.014	0.027
T1C wav HHL gldm LDLGLE	-0.485	-0.387	-0.237	0.007	0.21	0.023	-0.083	0.024	0.063	0.133	-0.058	-0.068	-0.064	0.078	0.131	-0.09	0.046	0.005	-0.103	-0.041	0.024	0.116	0.049	-0.049	0.052	0.055	-0.172	-0.053	-0.164	0.112	0.08	0.012
T1C wav HHL gldm LDHGLE	0.213	0.1	-0.031	0.152	0.024	-0.064	0.717	-0.018	0.115	-0.059	-0.011	0.077	0.026	0.042	0.027	-0.073	-0.091	-0.048	0.085	-0.021	-0.009	-0.054	-0.001	-0.057	0.022	-0.007	0.042	-0.016	0.079	-0.033	0.091	0.021
T1C wav HHL glcm Idn	0.654	-0.09	-0.092	-0.004	0.036	0.035	0.261	0.048	0.24	0.057	0.114	-0.013	0.095	0.009	-0.032	-0.022	0.201	-0.021	0.074	-0.025	-0.009	-0.099	0.061	0.044	0.055	-0.014	-0.05	0.001	0.072	0.04	-0.026	-0.008
T1C wav HHL glcm ClusterShade	0.092	0.152	0.041	0.111	-0.009	-0.014	0.566	0.452	-0.003	-0.037	-0.001	-0.072	-0.005	0.004	0.032	0.026	0.165	-0.118	-0.068	-0.061	-0.023	0.039	0.113	0.067	0.077	0.015	0.034	0	-0.033	-0.062	-0.032	0.002
T1C wav HHL glcm Correlation	0.409	0.076	0.203	-0.105	-0.118	-0.09	0.073	0.007	0.029	-0.05	-0.064	-0.039	0.011	-0.108	0.002	-0.071	0.174	-0.054	-0.035	-0.079	-0.158	-0.022	-0.004	0.545	0.017	0.005	-0.071	-0.012	0.005	-0.047	-0.049	0.049
T1C wav HHL glcm ClusterProminence	0.064	0.105	0.067	0.084	0.001	-0.045	0.758	0.218	-0.032	-0.024	-0.064	0.015	0.003	-0.01	0.058	-0.009	-0.013	-0.059	0.026	-0.034	0.029	0.004	0.061	-0.004	0.033	-0.017	0.012	-0.012	-0.047	-0.044	0.052	-0.001
T1C wav HHL firstorder RootMeanSquared	0.04	0.413	0.161	0.043	-0.014	-0.098	0.388	0.21	-0.165	-0.083	-0.017	0.011	-0.073	0.068	0.031	0.062	0.117	0.043	-0.025	-0.104	0.436	0.033	0.095	-0.046	0.023	0.018	0.029	-0.058	0.018	0.018	-0.014	0.01
T1C wav HHL firstorder Kurtosis	0.297	-0.152	0.004	0.031	-0.039	0.101	0.577	0.102	0.075	-0.017	0.065	-0.066	0.183	-0.024	-0.006	-0.017	0.335	-0.068	0.095	-0.058	-0.047	-0.071	0.089	0.068	0.038	-0.09	0.012	0.01	0.057	0.054	-0.037	-0.022
T1C wav HHL firstorder Maximum	0.236	0.437	0.139	0.093	-0.067	-0.037	0.464	0.256	0.053	-0.089	0.046	0.007	0.011	0.06	-0.005	0.008	0.229	-0.041	-0.01	-0.013	0.113	-0.07	0.132	0.06	0.058	-0.048	-0.029	0.013	-0.021	0.03	-0.076	0.033
T1C wav HHL firstorder Mean	0.024	0.242	0.076	-0.025	-0.012	-0.052	0.256	0.102	-0.124	-0.016	0.027	0.007	-0.089	0.062	0.068	0.122	0.197	-0.004	-0.031	-0.154	0.601	0.107	0.083	-0.139	0.064	0.014	0.071	-0.04	0.03	0.012	-0.035	-0.022
T1C wav HHL firstorder Skewness	0.053	-0.018	0.07	0.004	-0.026	0.123	0.153	0.221	0.03	0.024	0.152	-0.019	0.009	0.016	-0.012	0.063	0.687	0.008	-0.117	-0.148	0.033	0.103	0.152	0.095	0.049	-0.026	-0.014	0.002	-0.023	-0.032	-0.057	-0.031
T1C wav HHL firstorder Range	0.249	0.453	0.164	0.124	-0.062	-0.083	0.525	0.189	0.063	-0.101	0.026	0.037	0.01	0.065	-0.007	-0.052	0.085	-0.019	0.023	-0.009	0.092	-0.1	0.082	0.03	0.025	-0.033	-0.004	-0.022	0.027	0.016	-0.008	0.051
T1C wav HHL firstorder InterquartileRange	0.027	0.624	0.226	0.072	-0.051	-0.175	0.084	0.179	-0.204	-0.168	-0.037	0.081	-0.041	0.032	-0.023	0.014	-0.074	0.084	-0.036	-0.036	0.228	-0.064	0.023	0.069	-0.026	-0.042	-0.01	-0.041	-0.038	0.033	-0.024	0.058
T1C wav HHL firstorder Median	-0.043	0.095	0.012	0.01	0.007	-0.041	0.106	-0.01	-0.048	-0.004	-0.05	0.171	-0.001	-0.03	0.049	0.072	-0.069	-0.092	0.025	0	0.733	-0.028	-0.075	-0.114	0.013	-0.001	-0.04	0.062	-0.011	-0.001	-0.016	-0.011
T1C wav HHL ngtdm Strength	-0.166	0.277	0.28	0.035	-0.044	-0.075	0.516	0.07	-0.331	-0.167	-0.044	-0.057	-0.021	-0.016	-0.011	0.036	0.043	0.121	0.044	0.076	0.152	-0.065	0.098	0.106	-0.015	-0.051	0.012	-0.027	-0.005	0.017	0.015	0.049
T1C wav HHL ngtdm Busyness	0.152	-0.438	-0.32	-0.005	0.376	-0.013	-0.168	-0.006	0.175	0.111	-0.075	-0.023	-0.076	-0.033	0.127	-0.006	-0.023	0.035	-0.086	0.067	0.017	0.193	0.036	-0.017	0.037	0.015	-0.151	-0.013	-0.205	0.051	0.051	-0.043
T1C wav HHL glszm ZoneVariance	0.082	-0.27	-0.181	0.034	0.748	0.014	-0.051	-0.005	-0.003	0.002	0.008	-0.079	-0.023	-0.025	0.029	-0.007	-0.01	-0.009	-0.018	0	0.001	0.021	0.011	-0.008	0.009	0	-0.037	-0.021	0.017	-0.02	0.016	-0.018
T1C wav HHL glszm SmallAreaEmphasis	0.002	0.557	0.513	0.001	-0.163	-0.045	0.059	0.095	-0.143	-0.041	0.028	0.017	0.013	-0.014	-0.064	-0.119	0.024	-0.017	-0.005	0.003	0.103	-0.068	0.015	0.017	-0.03	-0.071	0.047	-0.043	-0.044	-0.041	-0.046	0.038
T1C wav HHL glszm LAHGLE	0.135	-0.178	-0.165	0.085	0.739	0.015	0.175	-0.021	0.049	-0.044	0.023	-0.025	0.006	-0.044	-0.004	-0.002	-0.041	-0.03	0.042	-0.02	-0.013	-0.039	0.008	-0.027	-0.003	0.01	0.024	-0.002	0.081	-0.044	0.025	-0.02
T1C wav LLL glrlm LRHGLE	0.192	0.589	-0.21	-0.069	0.009	0.009	-0.089	0.198	0.106	-0.013	-0.08	0.091	0.082	0.078	0.107	0.019	0.044	-0.092	-0.013	0.002	-0.06	0.111	0.095	-0.049	-0.148	0.11	0.238	-0.001	-0.023	0.001	-0.027	0.022

T1C feature name	T1C F1	T1C F2	T1C F3	T1C F4	T1C F5	T1C F6	T1C F7	T1C F8	T1C F9	T1C F10	T1C F11	T1C F12	T1C F13	T1C F14	T1C F15	T1C F16	T1C F17	T1C F18	T1C F19	T1C F20	T1C F21	T1C F22	T1C F23	T1C F24	T1C F25	T1C F26	T1C F27	T1C F28	T1C F29	T1C F30	T1C F31	T1C F32
T1C wav LLL gldm SDLGLE	-0.809	-0.126	0.062	0.009	-0.043	0.061	0.002	-0.044	0.011	0.014	0.004	0.003	-0.028	0.05	0.003	-0.015	-0.033	-0.013	-0.007	-0.018	0.011	-0.038	0.019	-0.006	0.038	0.044	-0.017	-0.01	0.012	-0.029	-0.041	0.028
T1C wav LLL gldm LDLGLE	-0.643	-0.246	-0.014	-0.026	-0.02	0.036	-0.03	0.003	-0.095	0.179	0.167	0.032	-0.049	0.033	0.011	-0.047	-0.104	-0.047	-0.074	-0.058	0.055	-0.097	-0.078	-0.165	0.04	-0.106	-0.071	0.011	0.082	0.016	0.002	-0.007
T1C wav LLL gldm DependenceVariance	0.277	-0.542	-0.263	0.138	0.376	0.105	-0.002	-0.027	0.023	0.138	-0.035	0.04	-0.019	-0.101	0.114	-0.014	0.034	0.111	-0.051	-0.002	-0.023	0.049	0.025	-0.073	0.027	0.047	-0.058	-0.026	0.008	0.032	0.061	0.02
T1C wav LLL glcm Idn	0.6	-0.178	-0.085	0.141	0.138	0.125	0.039	0.118	0.222	0.143	0.014	-0.019	0.163	-0.033	0.029	0.015	0.046	-0.01	0.014	-0.092	-0.002	0.05	0.09	-0.09	0.12	0.11	0.207	0.018	-0.074	0.105	0.044	0.028
T1C wav LLL glcm ClusterShade	0.095	0.119	0.064	0.132	0.009	0.097	0.105	0.109	-0.025	-0.004	0.167	-0.009	0.004	-0.034	-0.07	0.014	0.075	0.038	0.046	-0.036	0.036	0.019	0.122	-0.048	0.707	0.028	0.003	0.036	0.003	0.008	-0.018	-0.02
T1C wav LLL glcm Correlation	0.624	-0.117	-0.004	0.146	0.125	0.232	0.066	0.015	0.036	0.187	0.079	-0.053	-0.083	0.15	0.037	0.109	-0.104	-0.035	0.031	-0.061	-0.037	-0.015	0.068	0.002	0.194	0.032	0.173	-0.013	-0.007	-0.001	0.015	0
T1C wav LLL glcm ClusterTendency	0.175	0.621	-0.114	-0.026	0.013	0.22	-0.016	0.139	-0.032	0.127	0.04	0.004	-0.057	0.269	0.139	0.042	-0.019	-0.038	0.034	-0.018	0.007	-0.07	0.112	0.012	0.131	0.061	0.142	0.06	-0.003	-0.057	0.05	-0.048
T1C wav LLL glcm Idm	0.205	-0.692	0.022	0.141	0.207	0.122	0.026	-0.014	0.046	0.209	0.002	-0.019	0.043	-0.1	0.048	-0.016	0.013	0.028	0.017	-0.082	-0.046	0.007	0.003	-0.064	0.008	0.089	0.025	-0.002	-0.022	0.032	0.035	-0.046
T1C wav LLL glcm DifferenceEntropy	0.332	0.694	-0.145	-0.149	-0.101	-0.075	-0.03	0.091	-0.039	-0.131	-0.008	0.02	-0.048	0.105	-0.006	-0.002	0.008	-0.04	-0.015	0.028	-0.001	-0.015	-0.014	0.026	-0.01	-0.018	-0.002	0.008	0.032	-0.018	-0.038	0.008
T1C wav LLL firstorder Maximum	0.1	0.594	-0.051	-0.124	-0.07	0.005	-0.014	0.226	0.104	0	-0.009	0.389	0.095	0.128	0.101	0.055	0	-0.023	-0.025	-0.007	0.06	0.009	0.081	-0.015	0.115	0.069	0.141	0.038	-0.038	-0.004	0.014	0.037
T1C wav LLL firstorder 10Percentile	-0.216	0.268	0.092	-0.188	-0.114	-0.147	-0.052	0.067	0.136	-0.048	-0.034	0.662	0.029	-0.024	0.097	-0.034	0.022	-0.033	0.044	0.012	0.055	0.103	0.01	0.014	-0.05	0.018	0.029	-0.019	0.001	0.002	-0.025	0.031
T1C wav LLL firstorder Skewness	0.325	0.047	0.098	0.072	0.095	0.093	0.287	0.014	-0.104	0.078	0.068	-0.083	0.07	0.083	-0.046	0.061	-0.097	-0.012	-0.094	0.073	0.027	-0.16	0.067	0.021	0.549	-0.014	-0.066	0.052	-0.078	-0.034	-0.092	0.118
T1C wav LLL firstorder Range	0.307	0.598	-0.164	-0.021	0.009	0.067	-0.021	0.251	0.103	-0.016	-0.026	0.045	0.124	0.108	0.071	0.07	0.035	-0.039	-0.048	-0.016	0	0.015	0.105	-0.055	0.099	0.089	0.209	0.04	-0.05	0.02	0.006	0.042
T1C wav LLL firstorder InterquartileRange	0.151	0.662	-0.182	-0.062	-0.053	0.063	-0.108	0.121	-0.094	0.067	-0.026	0.005	-0.068	0.286	0.056	0.086	-0.074	-0.043	-0.036	-0.029	-0.02	-0.046	0.014	0.047	-0.037	-0.005	0.132	0.032	0.013	-0.062	0.054	-0.044
T1C wav LLL firstorder 90Percentile	-0.072	0.58	-0.014	-0.178	-0.097	-0.052	-0.066	0.109	0.049	-0.022	-0.036	0.496	-0.021	0.118	0.109	0.014	-0.015	-0.029	0.034	-0.001	0.033	0.045	0.026	0.037	-0.046	0.02	0.091	-0.006	0.007	-0.039	-0.005	0.003
T1C wav LLL firstorder Minimum	-0.358	0.018	0.196	-0.186	-0.142	-0.109	0.011	-0.035	0.006	0.027	0.027	0.612	-0.046	0.04	0.06	-0.026	-0.061	0.026	0.037	0.016	0.106	-0.01	-0.038	0.069	0.03	-0.032	-0.112	-0.001	0.02	-0.044	0.013	-0.008
T1C wav LLL firstorder TotalEnergy	0.35	0.222	-0.275	0.018	0.021	-0.048	-0.04	0.114	0.513	0.018	-0.06	0.31	0.028	0.009	0.103	0.03	-0.013	-0.019	0.038	0.054	0.015	0.045	0.083	-0.016	-0.092	0.028	0.023	0.005	-0.054	-0.05	0.009	0.058
T1C wav LLL ngtdm Complexity	0.171	0.645	-0.183	-0.061	0.006	0.078	0.002	0.311	0.093	0.042	-0.034	0.076	0.04	0.165	0.132	0.024	-0.005	-0.065	-0.043	0.006	-0.02	0.01	0.11	-0.01	-0.027	0.086	0.097	0.04	-0.007	-0.023	-0.003	-0.004
T1C wav LLL ngtdm Strength	-0.208	0.463	0.048	-0.057	-0.046	0.199	-0.021	0.043	-0.363	-0.076	0.01	-0.124	-0.052	0.128	0.056	0.245	-0.023	-0.014	-0.023	0.027	0.04	-0.038	0.151	-0.03	0.176	0.009	0.245	0.04	-0.013	-0.063	0.112	0.021
T1C wav LLL ngtdm Busyness	0.185	-0.471	-0.148	0.064	0.07	-0.13	0.058	-0.064	0.332	0.131	-0.033	-0.073	-0.03	-0.046	0.034	-0.129	-0.008	0.014	-0.031	0.068	0.068	0.009	-0.04	0.05	0.059	-0.022	-0.387	-0.008	-0.08	-0.047	-0.02	0.014
T1C wav LLL ngtdm Contrast	-0.587	0.426	-0.001	-0.073	-0.065	-0.063	-0.057	-0.034	-0.289	-0.128	-0.075	-0.004	-0.04	0.067	0.061	0.124	-0.038	0.068	-0.017	0.015	-0.032	-0.052	-0.035	0.008	0.001	-0.058	-0.017	0.008	-0.002	-0.049	0.033	-0.041
T1C wav LLL glszm GLNUN	-0.573	-0.521	0.14	0.066	0.029	0.031	0.046	-0.063	0.046	0.059	-0.008	0.041	0.03	-0.142	0.062	-0.082	0.072	0.005	0.022	-0.037	0.004	-0.008	0.015	-0.03	-0.008	0.036	-0.101	0.006	-0.048	0.032	-0.009	0.014
T1C wav LLL glszm GLNU	0.404	-0.193	-0.212	0.161	0.192	-0.054	0.053	-0.024	0.564	-0.03	-0.054	0.025	0.001	-0.126	0.046	-0.025	0.017	0.043	-0.016	0.018	0.019	0.03	-0.001	-0.018	-0.018	-0.013	-0.133	0.006	-0.032	-0.022	-0.006	-0.001
T1C lbp 2D firstorder Kurtosis	-0.43	0.056	0.002	-0.635	-0.017	-0.153	-0.091	-0.044	-0.013	0.026	-0.028	0.067	0.065	-0.03	0.091	0.037	0.034	0.056	-0.088	-0.119	-0.086	0.054	-0.02	-0.005	0.028	0.054	0.029	-0.06	0.022	-0.056	-0.012	-0.034
T1C lbp 2D firstorder RMAD	0.19	0.051	0.497	0.533	-0.118	0.022	0.113	0.011	0.024	-0.078	0.014	0.084	-0.159	-0.009	0.029	-0.121	0.024	0.005	0.098	0.099	0.103	-0.034	0.059	0.018	-0.023	-0.051	-0.028	0.045	-0.035	0.014	0.009	-0.026
T1C lbp 2D firstorder Uniformity	-0.212	0.05	0.737	-0.107	-0.076	-0.123	0.009	-0.043	-0.051	0.009	-0.003	0.042	0.01	-0.081	-0.013	-0.118	0.045	0.073	0.103	-0.012	0.073	0.081	0.069	0.06	-0.055	0.038	-0.036	0.028	0.015	-0.042	0.033	-0.02
T1C lbp 2D glcm ClusterShade	-0.286	0.271	0.293	-0.439	-0.131	-0.242	-0.054	-0.014	-0.005	-0.067	-0.061	0.141	-0.11	-0.101	0.167	-0.01	0.117	0.098	-0.005	-0.07	0.006	-0.106	-0.046	-0.046	0.005	0.14	-0.019	-0.035	-0.049	0.048	0.01	0.146
T1C lbp 2D glcm ClusterTendency	0.15	0.058	0.405	0.499	-0.096	-0.044	0.163	0.007	0.02	-0.053	0.043	0.023	-0.276	-0.117	-0.017	-0.106	0.029	0.024	0.075	0.14	0.126	-0.171	0.042	0.034	-0.058	0.06	0.01	0.03	-0.083	0.027	0.077	-0.061
T1C lbp 2D glcm Correlation	0.262	0.017	-0.41	-0.028	-0.046	0.123	0.074	0.04	-0.013	0.085	0.101	-0.039	-0.393	-0.125	-0.108	0.043	0.026	-0.177	-0.115	0.115	-0.02	-0.209	-0.061	0.105	0.05	0.176	0.059	0.048	-0.2	0.034	0.034	-0.057
T1C lbp 2D glcm DifferenceEntropy	0.709	0.075	-0.079	0.296	-0.023	-0.087	0.07	0.082	0.046	-0.114	0.013	-0.006	-0.072	-0.001	0.066	-0.041	0.018	-0.01	0.041	0.044	0.028	-0.109	-0.007	-0.03	0.016	-0.059	0.019	-0.066	0.1	0.059	0.04	-0.011
T1C lbp 2D glcm DifferenceVariance	-0.065	0.048	0.721	0.142	-0.062	-0.108	0.062	-0.081	0.021	-0.048	-0.019	0.113	0	-0.058	0.05	-0.163	-0.008	0.164	0.089	0.063	0.125	-0.022	0.103	0.033	-0.042	0.004	0.013	-0.013	0.014	-0.021	0.018	-0.015

T1C feature name	T1C F1	T1C F2	T1C F3	T1C F4	T1C F5	T1C F6	T1C F7	T1C F8	T1C F9	T1C F10	T1C F11	T1C F12	T1C F13	T1C F14	T1C F15	T1C F16	T1C F17	T1C F18	T1C F19	T1C F20	T1C F21	T1C F22	T1C F23	T1C F24	T1C F25	T1C F26	T1C F27	T1C F28	T1C F29	T1C F30	T1C F31	T1C F32
T1C lbp 2D glcm Idm	0.014	-0.082	0.081	-0.711	-0.047	0.013	-0.068	-0.092	-0.052	0.141	0.067	0.053	0.05	-0.041	-0.051	-0.069	-0.033	-0.027	-0.106	0.03	-0.038	-0.034	0.078	0.117	0.089	0.207	0.095	0.005	-0.018	-0.075	-0.021	0.011
T1C lbp 2D glcm Idn	-0.011	-0.057	-0.401	-0.637	0.023	0.073	-0.096	-0.034	-0.039	0.115	0.034	0.001	0.013	0.004	-0.029	0.069	-0.028	-0.096	-0.15	-0.027	-0.103	-0.027	-0.024	0.07	0.095	0.134	0.062	0.013	-0.053	-0.034	-0.044	0.025
T1C lbp 2D glcm InverseVariance	-0.047	-0.025	-0.056	-0.544	0.092	0.152	-0.102	-0.157	-0.05	0.092	-0.133	0.061	-0.02	-0.006	0.088	0.114	-0.191	-0.097	-0.209	0.008	-0.11	-0.022	-0.003	0.121	0.123	-0.045	0.101	0.087	-0.227	-0.104	-0.108	-0.004
T1C lbp 2D glcm MaximumProbability	-0.414	0.025	0.664	0.035	-0.086	-0.069	0.049	-0.09	-0.022	-0.032	0.021	0.009	0.042	-0.052	-0.072	-0.101	0.055	0.039	0.095	-0.025	0.068	0.012	0.04	0.038	-0.016	0.028	-0.008	0.036	0.039	-0.022	0.072	0.014
T1C lbp 2D glcm SumEntropy	0.711	0.063	-0.265	0.213	-0.009	-0.033	0.066	0.071	0.012	-0.035	0.029	-0.036	-0.173	-0.016	0.013	0.017	0.032	-0.052	0.023	0.073	-0.048	-0.08	-0.037	0.006	0	0.005	0.024	-0.014	0.013	0.068	0.02	-0.048
T1C lbp 2D glrlm LRLGLE	-0.071	0.156	0.717	-0.046	-0.145	-0.183	0.002	-0.032	-0.027	-0.107	0.038	0.085	-0.13	-0.033	0.05	-0.056	0.137	0.07	0.054	-0.035	0.026	0.024	0.004	0.046	0.037	0.09	0.018	-0.043	0.035	0.006	-0.006	-0.088
T1C lbp 2D glrlm RunLengthNonUniformityNormalized	-0.14	0.091	0.662	0.225	-0.049	0.153	-0.002	0.031	-0.08	-0.03	-0.067	-0.095	0.007	0.042	-0.016	0.148	-0.03	-0.037	-0.053	-0.06	-0.028	-0.053	-0.037	-0.029	0.002	-0.287	-0.01	0.02	-0.083	-0.035	0.031	-0.008
T1C lbp 2D glrlm RunVariance	0.349	-0.09	-0.523	-0.152	-0.004	-0.042	-0.015	-0.005	0.198	0.067	0.123	0.073	0.018	-0.046	0.015	-0.184	0.069	0.021	0.073	0.024	0.023	0.064	0.027	0.066	0.097	0.332	-0.033	-0.014	0.074	0.008	0.036	-0.049
T1C lbp 2D glrlm SRHGLE	0.396	-0.193	-0.307	0.478	0.169	0.209	0.081	0.006	-0.003	0.109	0.005	-0.118	0.076	0.041	-0.19	-0.007	-0.034	0.001	0.055	0.037	0.069	-0.009	0.025	0.035	-0.022	-0.121	-0.011	0.047	0.031	0.032	0.008	0.009
T1C lbp 2D glszm GrayLevelVariance	0.353	-0.032	0.576	0.387	-0.043	0.083	0.03	0.018	-0.041	-0.039	0.014	0.033	0.015	0.035	-0.022	-0.083	0.026	-0.008	0.133	0.105	0.052	0.024	0.09	0.034	0.005	-0.112	-0.055	0.017	0.083	-0.017	-0.029	-0.044
T1C lbp 2D glszm LALGLE	0.118	0.19	0.507	-0.12	-0.153	-0.261	0.003	-0.052	0.013	-0.116	0.077	0.149	-0.203	-0.015	0.056	-0.097	0.171	0.104	0.055	-0.048	0.107	0.052	-0.038	0.093	0.093	0.21	0.036	-0.055	0.096	0.051	0.005	-0.155
T1C lbp 2D glszm SZNUN	-0.314	0.042	0.568	0.193	-0.027	0.175	-0.003	-0.043	-0.051	-0.048	-0.082	-0.107	0.072	0.079	0.018	0.196	-0.08	-0.047	-0.04	-0.029	-0.073	-0.064	0.002	-0.101	0.028	-0.218	-0.025	0.085	-0.107	-0.062	-0.033	-0.015
T1C lbp 2D glszm SALGLE	-0.321	0.016	0.711	0.003	-0.111	-0.053	-0.002	-0.014	0.013	-0.044	0.007	0.066	0.007	-0.075	0.097	-0.09	-0.032	-0.012	0.055	-0.011	-0.018	-0.014	0.102	0.009	0.015	0.022	-0.026	0.002	-0.029	-0.061	-0.043	-0.012
T1C lbp 2D glszm ZoneVariance	0.284	-0.086	-0.559	-0.102	0	0.007	0.01	-0.075	0.188	0.035	0.082	0.046	0.022	0.004	0.064	-0.138	-0.057	0.023	0.128	0.097	0.064	0.056	0.059	-0.028	0.095	0.372	-0.024	0.06	0.045	0.001	-0.026	-0.082
T1C lbp 2D gldm DependenceVariance	0.133	-0.125	-0.585	-0.235	0.039	-0.094	0.003	-0.085	0.156	0.045	0.102	0.051	0.086	0.014	0.035	-0.069	0.015	0.035	0.06	0.043	-0.074	0.034	0.1	-0.065	0.076	0.367	-0.029	0.084	0.077	0.01	-0.04	-0.022
T1C lbp 2D gldm LDHGLE	0.198	-0.193	-0.524	0.338	0.103	-0.002	0.152	-0.028	0.135	0.094	0.102	-0.028	0.003	-0.091	-0.057	-0.196	-0.078	0.021	0.084	0.059	0.113	-0.07	0.11	0.017	-0.055	0.213	0.024	0.041	-0.018	0.006	0.024	0.029
T1C lbp 2D gldm SDHGLE	0.41	-0.115	0.024	0.403	0.13	0.29	0.006	0.005	-0.083	0.061	-0.061	-0.169	0.1	0.137	-0.206	0.144	0.003	-0.041	-0.006	0.02	-0.023	0.02	-0.014	-0.007	0.016	-0.305	-0.019	0.043	0.031	-0.01	0.00E+00	-0.008
T1C lbp 2D ngtdm Complexity	0.222	0.098	0.642	0.295	-0.084	-0.037	0.035	0.001	-0.072	-0.061	0	0.032	-0.025	-0.035	-0.04	-0.138	0.069	0.024	0.156	0.126	-0.035	0.032	0.086	0.027	-0.089	-0.089	-0.096	0.019	0.087	-0.057	0.02	0.029
T1C lbp 2D ngtdm Contrast	-0.243	0.027	0.557	0.391	-0.024	-0.048	0.11	-0.038	0.069	-0.02	0.014	0.094	0.043	-0.036	0.036	-0.132	-0.013	0.114	0.087	-0.063	0.276	0.033	0.034	0.055	-0.027	-0.038	-0.025	0.044	-0.03	0.043	-0.036	-0.044
T1C lbp 3D m1 firstorder 90Percentile	0.417	-0.216	-0.395	0.375	0.152	0.226	0.015	-0.063	0.034	0.09	0.004	-0.136	-0.012	0.087	-0.133	0.057	-0.098	-0.03	-0.055	-0.051	-0.051	-0.078	-0.027	-0.003	0.043	-0.039	0.038	0.059	-0.042	-0.008	0.065	-0.005
T1C lbp 3D m1 firstorder InterquartileRange	0.533	-0.089	0.316	0.35	0.034	0.221	0.067	0.021	-0.026	-0.024	0.092	-0.046	0.023	0.075	-0.031	0.015	0.184	-0.093	-0.062	-0.121	0.042	0.012	-0.022	-0.016	0.06	0.014	0.075	-0.013	0.025	-0.001	0.03	0.061
T1C lbp 3D m1 firstorder Kurtosis	-0.035	0.157	-0.032	-0.147	-0.111	-0.168	-0.055	-0.048	-0.013	0.054	-0.065	0.027	-0.032	-0.048	0.046	-0.038	-0.165	0.029	0.085	0.701	-0.127	-0.12	-0.006	0.054	-0.003	0.042	-0.032	-0.034	-0.04	-0.007	0.006	-0.063
T1C lbp 3D m1 firstorder Median	0.223	-0.18	-0.695	0.048	0.145	0.175	0.00E+00	-0.005	0.034	0.047	-0.013	-0.109	0.049	0.05	-0.129	0.075	-0.036	-0.032	-0.067	-0.078	0.033	-0.045	0.031	-0.029	0.007	-0.07	0.055	-0.011	0.048	-0.018	-0.019	0.028
T1C lbp 3D m1 firstorder RMAD	0.405	-0.183	0.312	0.424	0.066	0.251	0.077	-0.036	0.036	0.019	0.106	-0.123	-0.044	0.079	-0.101	0.043	0.119	-0.11	-0.073	-0.194	-0.022	-0.012	-0.027	-0.032	0.004	0.065	0.062	-0.006	0.067	-0.011	0.005	0.059
T1C lbp 3D m1 firstorder Skewness	0.143	0.168	0.628	0.159	-0.129	-0.076	0.009	-0.043	0.002	0.04	-0.024	0.085	-0.154	-0.026	0.059	-0.015	-0.064	0.038	0.024	0.331	-0.059	-0.062	-0.039	0.047	0.07	0.025	-0.013	0.084	-0.072	-0.013	0.084	-0.025
T1C lbp 3D m1 firstorder Uniformity	-0.629	0.059	0.288	-0.306	-0.075	-0.163	-0.064	-0.009	-0.007	0.012	-0.029	0.041	0.06	-0.077	0.038	-0.09	0.082	0.037	0.13	0.055	-0.008	0.149	0.01	0.007	-0.063	0.025	-0.033	-0.028	-0.016	0.013	-0.001	-0.069
T1C lbp 3D m1 glcm Autocorrelation	0.286	-0.249	-0.585	0.186	0.172	0.186	-0.02	-0.031	0.01	0.126	-0.02	-0.124	0.084	0.046	-0.183	0.038	-0.116	-0.06	-0.061	0.008	0.002	-0.072	-0.004	0.007	0.023	-0.017	0.042	0.049	-0.051	-0.021	-0.01	0.003
T1C lbp 3D m1 glcm ClusterShade	0.189	0.018	0.519	0.35	-0.15	0.166	-0.037	-0.104	0.096	0.018	-0.034	0.05	-0.079	0.004	0.06	0.026	-0.097	-0.095	-0.046	0.276	-0.005	-0.045	-0.074	0.007	0.008	0.009	0.029	0.014	-0.135	-0.119	0.099	-0.007
T1C lbp 3D m1 glcm DifferenceEntropy	0.713	-0.079	-0.016	0.361	0.061	0.079	0.038	-0.019	0.078	0.062	0.008	-0.09	0.001	0.009	-0.033	-0.003	0.009	-0.031	-0.032	-0.025	-0.052	-0.029	-0.041	-0.003	0.044	0.04	0.043	0.01	0.044	0.018	0.04	0.06

T1C feature name	T1C F1	T1C F2	T1C F3	T1C F4	T1C F5	T1C F6	T1C F7	T1C F8	T1C F9	T1C F10	T1C F11	T1C F12	T1C F13	T1C F14	T1C F15	T1C F16	T1C F17	T1C F18	T1C F19	T1C F20	T1C F21	T1C F22	T1C F23	T1C F24	T1C F25	T1C F26	T1C F27	T1C F28	T1C F29	T1C F30	T1C F31	T1C F32
T1C lbp 3D m1 glcm DifferenceVariance	0.478	-0.22	0.142	0.502	0.043	0.202	0.036	-0.085	0.097	0.02	0.017	-0.109	0.007	0.048	-0.068	0.041	0.001	-0.061	-0.09	0.052	-0.032	-0.078	-0.028	-0.043	0.093	0.042	0.099	-0.007	0.022	-0.054	0.072	0.089
T1C lbp 3D m1 glcm Idm	-0.363	0.084	-0.018	-0.619	-0.114	-0.121	-0.033	-0.014	-0.038	-0.119	0.049	0.028	-0.051	-0.013	0.052	-0.08	0.2	-0.069	0.118	0.085	0.043	0.038	-0.006	0.019	-0.044	-0.002	-0.032	-0.178	0.019	0.045	-0.008	-0.044
T1C lbp 3D m1 glcm Idn	0.244	0.189	-0.196	-0.574	-0.123	-0.111	-0.055	0.008	-0.132	-0.081	0.056	0.029	-0.118	-0.018	0.001	-0.056	0.053	-0.086	0.111	0.297	-0.094	-0.02	0.021	-0.005	-0.069	-0.034	-0.077	-0.11	0.026	0.026	-0.043	-0.057
T1C lbp 3D m1 glcm InverseVariance	-0.358	0.146	-0.115	-0.621	-0.095	-0.162	0.038	-0.002	0.002	-0.077	0.014	0.135	-0.101	0.01	0.064	0.003	0.029	0.018	0.023	0.07	0.164	0.004	0.026	0.017	-0.002	0.023	-0.033	-0.062	0.013	0.099	-0.054	-0.06
T1C lbp 3D m1 glcm MaximumProbability	-0.718	-0.057	0.287	-0.161	-0.069	0.021	-0.056	-0.047	-0.09	-0.029	-0.013	-0.065	0.058	-0.008	-0.012	-0.014	0.105	0.045	0.099	0.039	-0.029	0.069	0.016	-0.002	-0.023	-0.069	0.034	-0.037	-0.006	-0.031	0.045	-0.032
T1C lbp 3D m1 glcm SumEntropy	0.764	-0.028	-0.128	0.227	0.056	0.083	0.029	-0.004	0.076	0.044	0.007	-0.06	-0.059	0.01	-0.032	0.001	0.012	-0.065	-0.027	-0.005	-0.023	-0.043	-0.037	0.042	0.023	0.043	0.026	0.017	0.004	0.047	0.041	0.018
T1C lbp 3D m1 glrlm ShortRunEmphasis	-0.016	0.048	0.658	0.194	-0.085	0.104	0.094	0.051	-0.021	0.17	0.036	-0.028	0.046	0.02	-0.099	0.136	-0.164	0.079	-0.176	-0.107	0.006	-0.127	0.045	-0.019	0.058	-0.047	0.042	0.161	0.023	-0.013	0	0.03
T1C lbp 3D m1 glszm GrayLevelVariance	0.521	-0.171	-0.133	0.448	0.103	0.279	0.02	-0.061	0.048	0.035	0.019	-0.134	-0.012	0.093	-0.079	0.076	-0.032	-0.056	-0.028	0.057	-0.043	-0.088	-0.039	-0.054	0.1	-0.011	0.049	0.064	-0.022	-0.011	0.035	0.048
T1C lbp 3D m1 glszm HGLZE	0.418	-0.205	-0.486	0.222	0.187	0.219	0.005	-0.048	0.016	0.129	-0.009	-0.118	0.058	0.071	-0.181	0.065	-0.015	-0.003	-0.056	-0.051	-0.023	-0.06	-0.035	-0.005	0.02	-0.054	0.044	0.093	0	-0.039	0.012	0.08
T1C lbp 3D m1 glszm LALGLE	0.086	0.013	0.554	-0.102	-0.051	-0.063	-0.058	-0.026	0.127	-0.077	0.004	-0.064	0.031	0.02	-0.03	-0.078	0.432	0.064	0.176	-0.044	-0.039	0.096	-0.176	0.08	0.012	0.006	0.065	-0.133	0.077	0.009	0.014	-0.04
T1C lbp 3D m1 glszm LGLZE	-0.315	0.076	0.704	0.061	-0.087	-0.04	-0.022	-0.002	0.031	-0.031	0.043	0.021	-0.017	-0.024	0.061	-0.087	0.027	-0.017	0.082	0.015	-0.002	0.112	0.006	0.016	0.07	0.101	-0.055	-0.105	-0.017	0.129	-0.01	-0.029
T1C lbp 3D m1 glszm SmallAreaEmphasis	-0.016	0.017	0.622	0.162	-0.063	0.198	0.119	0.031	0.023	0.175	0.116	-0.035	0.042	0.091	-0.158	0.092	-0.122	-0.035	-0.116	-0.113	-0.033	-0.122	0.053	-0.037	0.095	-0.037	0.003	0.14	-0.119	0.021	-0.05	0.055
T1C lbp 3D m1 glszm SAHGLE	0.48	-0.157	-0.309	0.243	0.157	0.266	0.035	-0.043	0.003	0.187	0.008	-0.121	0.035	0.081	-0.218	0.076	0.003	0.005	-0.075	-0.071	-0.029	-0.103	-0.026	-0.035	0.044	-0.07	0.043	0.159	0.002	-0.058	-0.017	0.099
T1C lbp 3D m1 glszm SALGLE	-0.424	0.04	0.573	0.162	-0.092	0.023	-0.026	0.008	0.122	0.032	0.043	0.045	0.023	0.008	0.081	-0.064	-0.166	-0.08	0.02	0.026	-0.024	0.104	-0.032	-0.006	0.138	0.074	-0.069	-0.055	-0.068	0.159	0.042	-0.005
T1C lbp 3D m1 glszm ZoneVariance	0.165	-0.096	-0.612	-0.156	0.119	-0.059	-0.106	-0.086	-0.021	-0.149	-0.08	-0.005	0.064	0.059	0.043	-0.055	-0.019	-0.109	0.203	0.205	-0.061	0.149	-0.074	0.057	-0.033	0.005	-0.005	-0.087	-0.014	0.079	0.034	-0.075
T1C lbp 3D m1 gldm DependenceVariance	0.118	-0.1	-0.558	-0.22	0.071	-0.008	-0.049	-0.063	0.097	-0.123	0.057	0.005	-0.016	0.095	0.035	-0.137	0.248	-0.152	0.18	0.062	-0.012	0.089	-0.053	0.032	0.004	0.068	-0.092	-0.185	-0.1	0.004	-0.059	-0.008
T1C lbp 3D m1 gldm SDHGLE	0.422	-0.161	-0.14	0.319	0.101	0.297	0.069	-0.022	-0.011	0.233	0.024	-0.15	0.056	0.088	-0.25	0.101	-0.06	0.009	-0.12	-0.096	-0.031	-0.143	-0.008	-0.029	0.052	-0.091	0.069	0.162	-0.005	-0.054	-0.017	0.085
T1C lbp 3D m1 gldm SDLGLE	-0.32	0.028	0.715	0.139	-0.077	0.041	-0.007	0.01	0.076	0.046	0.056	0.025	0.032	0.012	0.024	-0.035	-0.095	-0.029	0	-0.004	-0.003	0.071	-0.007	0	0.094	0.059	-0.044	-0.032	-0.059	0.088	0.005	-0.009
T1C lbp 3D m1 ngtdm Busyness	0.415	-0.011	0.05	0.234	0.15	-0.063	0.043	0.031	0.637	-0.014	-0.045	-0.019	0.008	-0.034	0.043	-0.022	0.052	0.052	-0.004	-0.031	-0.008	-0.025	-0.072	-0.009	0.016	0.003	0.007	-0.018	0.01	0.004	0.058	-0.009
T1C lbp 3D m1 ngtdm Complexity	0.541	-0.025	0.363	0.361	-0.032	0.188	0.012	-0.004	0.042	0.019	0.076	-0.102	0.022	0.049	-0.065	-0.036	0.029	-0.142	0.004	0.042	-0.108	0.023	-0.018	0.014	-0.009	0.057	-0.026	-0.048	0.019	0	-0.039	0.12
T1C lbp 3D m1 ngtdm Contrast	0.023	-0.282	0.225	0.581	0.099	0.248	0.08	-0.033	0.074	0.043	0.047	-0.116	0.016	0.088	-0.079	0.058	0.102	0.044	-0.069	-0.186	0.047	-0.07	-0.017	-0.059	0.056	0	0.143	0.014	0.052	0.012	0.036	0.035
T1C lbp 3D m2 firstorder 90Percentile	0.292	-0.182	-0.508	0.391	0.158	0.167	0.067	-0.035	0.133	0.025	0.091	-0.098	0.014	0.022	-0.063	0.04	-0.081	-0.139	-0.092	-0.039	-0.034	-0.001	-0.072	0.092	0.068	-0.017	0	0.02	-0.07	-0.021	-0.022	-0.047
T1C lbp 3D m2 firstorder InterquartileRange	0.367	-0.009	0.567	0.265	0.057	0.1	0.084	-0.016	0.114	0.035	0.113	-0.077	0.031	-0.067	-0.067	0.006	0.196	-0.057	-0.048	-0.074	-0.038	0.083	0.022	-0.043	0.049	0.123	0.012	-0.087	0.027	0.006	-0.057	-0.073
T1C lbp 3D m2 firstorder Kurtosis	-0.121	-0.043	-0.499	-0.203	-0.01	-0.087	-0.104	0.017	-0.216	-0.074	-0.159	-0.061	0.025	0.092	-0.023	-0.013	-0.361	0.021	0.04	0.242	-0.064	-0.021	-0.033	0.074	-0.082	-0.146	0.031	0.08	-0.088	-0.069	0.12	0.047
T1C lbp 3D m2 firstorder Median	0.115	-0.112	-0.774	0.013	0.141	0.082	0.02	-0.015	0.021	0.044	0.024	-0.118	0.004	-0.012	-0.062	0.067	-0.035	-0.02	-0.066	-0.053	0.004	-0.031	-0.014	0.048	0.008	-0.035	0.026	-0.045	0.013	0.004	-0.038	-0.012
T1C lbp 3D m2 firstorder Range	0.59	0.116	-0.183	0.188	0.049	0.032	0.088	0.098	-0.076	-0.185	0.002	-0.089	-0.106	-0.064	0.047	0.026	-0.024	-0.102	0.141	0.187	-0.137	-0.029	0.028	0.09	0.11	-0.09	-0.051	-0.066	-0.001	-0.141	-0.052	-0.016
T1C lbp 3D m2 firstorder Skewness	0.132	0.107	0.73	0.145	-0.124	-0.064	-0.001	0.027	-0.06	-0.082	-0.043	0.101	-0.027	0.018	0.037	-0.047	-0.054	-0.018	0.012	0.159	-0.046	-0.038	-0.01	-0.06	0.006	-0.019	0.011	0.099	-0.045	-0.063	0.055	-0.008
T1C lbp 3D m2 firstorder Uniformity	-0.676	-0.041	-0.058	-0.298	-0.06	-0.087	-0.121	-0.014	-0.136	-0.011	-0.134	-0.041	0.032	0.018	-0.031	-0.034	-0.092	0.007	0.148	0.056	-0.02	0.097	-0.019	0.047	-0.1	-0.053	0.05	0.024	0.029	0.041	0.096	0.073
T1C lbp 3D m2 glcm Autocorrelation	0.174	-0.196	-0.691	0.126	0.178	0.106	-0.002	-0.031	0.065	0.101	0.026	-0.128	0.017	-0.009	-0.118	0.039	-0.102	-0.069	-0.056	0.042	-0.012	-0.037	-0.01	0.066	0.056	0.004	0.02	-0.001	-0.08	-0.012	-0.028	-0.079
T1C lbp 3D m2 glcm ClusterShade	0.019	0.091	0.7	0.155	-0.124	-0.032	-0.008	0.047	-0.022	-0.101	-0.096	0.063	0.139	0.051	0.061	-0.034	-0.127	-0.036	-0.043	0.108	-0.006	0.097	-0.026	-0.057	-0.107	-0.062	-0.018	0.06	-0.037	-0.121	0.055	-0.017
T1C lbp 3D m2 glcm ClusterTendency	0.268	-0.032	0.438	0.437	0.036	0.122	0.042	-0.05	0.181	-0.084	0.13	-0.024	-0.013	-0.043	0.008	0.042	0.069	-0.151	-0.13	0.083	-0.034	0.051	-0.046	0.088	0.02	0.123	0.005	-0.133	-0.047	-0.043	-0.038	-0.224

T1C feature name	T1C F1	T1C F2	T1C F3	T1C F4	T1C F5	T1C F6	T1C F7	T1C F8	T1C F9	T1C F10	T1C F11	T1C F12	T1C F13	T1C F14	T1C F15	T1C F16	T1C F17	T1C F18	T1C F19	T1C F20	T1C F21	T1C F22	T1C F23	T1C F24	T1C F25	T1C F26	T1C F27	T1C F28	T1C F29	T1C F30	T1C F31	T1C F32
T1C lbp 3D m2 glcm Correlation	0.216	0.108	0.055	-0.453	-0.036	-0.018	-0.11	-0.039	0.05	-0.092	-0.002	0.026	-0.139	-0.008	-0.01	0.09	0.119	-0.169	-0.066	0.133	0.087	0.054	0.058	0.022	-0.095	0.04	0.036	-0.083	-0.071	0.022	0.045	-0.491
T1C lbp 3D m2 glcm DifferenceAverage	0.127	-0.049	0.402	0.639	0.05	0.095	0.103	-0.021	0.113	-0.01	0.11	-0.015	0.081	-0.074	0.026	-0.038	-0.003	-0.036	-0.082	-0.006	-0.065	0.043	-0.058	0.067	0.056	0.079	-0.022	-0.068	-0.03	-0.072	-0.066	0.022
T1C lbp 3D m2 glcm DifferenceEntropy	0.708	0.036	0.126	0.35	0.06	0.011	0.082	-0.001	0.083	-0.013	0.069	-0.046	0.003	-0.061	0.02	-0.02	0.026	0.001	-0.028	0.034	-0.07	0.028	-0.068	0.053	0.033	0.068	-0.014	-0.065	0.032	0.004	-0.012	0.022
T1C lbp 3D m2 glcm DifferenceVariance	0.255	-0.093	0.306	0.578	0.065	0.163	0.078	-0.049	0.183	-0.09	0.153	-0.042	-0.018	0.002	0.003	0.039	0.018	-0.051	-0.096	0.073	-0.092	0.018	-0.078	0.06	0.074	0.106	0.009	-0.134	0.022	-0.029	-0.034	0.073
T1C lbp 3D m2 glcm Idm	-0.099	-0.067	-0.372	-0.588	-0.061	0.01	-0.111	0.012	-0.057	-0.095	-0.047	-0.018	-0.103	0.136	-0.068	0.098	0.116	-0.011	0.093	0.09	0.052	0.013	0.085	-0.067	-0.111	-0.084	0.087	-0.019	0.08	0.065	0.145	0.062
T1C lbp 3D m2 glcm Idn	0.207	0.071	-0.492	-0.517	-0.038	-0.062	-0.063	0.054	-0.158	-0.111	-0.051	-0.024	-0.128	0.065	0.008	0.031	0.02	0.014	0.127	0.101	0.007	-0.021	0.068	0.017	-0.024	-0.115	-0.037	0.055	0.059	-0.025	0.053	0.007
T1C lbp 3D m2 glcm InverseVariance	-0.057	-0.035	-0.426	-0.587	-0.078	-0.01	-0.132	0.016	-0.051	-0.031	-0.062	-0.04	-0.149	0.12	-0.018	0.142	0.038	-0.047	0.062	-0.123	0.032	-0.072	0.02	-0.039	-0.034	-0.05	0.045	0.024	0.038	0.091	0.053	0.045
T1C lbp 3D m2 glcm MaximumProbability	-0.751	-0.057	0.139	-0.092	-0.09	0.025	-0.068	-0.024	-0.186	-0.081	-0.012	-0.044	-0.002	0.011	-0.011	0.024	-0.016	0.024	0.079	0.1	0.014	0.018	-0.002	0.034	-0.062	-0.095	0.081	0.016	0.03	0.005	0.046	0.014
T1C lbp 3D m2 glcm SumEntropy	0.786	0.074	-0.011	0.122	0.042	-0.028	0.047	0.015	0.102	-0.02	0.054	-0.035	-0.056	-0.035	0.035	-0.013	0.053	-0.063	-0.021	-0.017	-0.035	0.014	-0.044	0.057	0.009	0.072	-0.002	-0.06	0.025	0.062	-0.001	-0.084
T1C lbp 3D m2 glrlm GrayLevelVariance	0.282	-0.059	0.358	0.518	0.034	0.183	0.102	-0.031	0.177	-0.053	0.186	-0.107	-0.043	-0.026	-0.009	0.006	0.143	-0.128	-0.041	-0.018	-0.044	0.01	-0.038	0.03	0.077	0.078	0.03	-0.121	-0.012	0.05	-0.074	-0.084
T1C lbp 3D m2 glrlm RunEntropy	0.667	0.003	-0.347	0.239	0.097	0.057	0.07	0.009	0.162	-0.074	0.063	0.016	-0.057	0.016	0.042	-0.002	0.04	-0.06	-0.049	0.059	0.003	-0.021	0.015	-0.017	0.041	0.017	-0.01	-0.031	-0.007	0.018	-0.016	-0.036
T1C lbp 3D m2 glszm GLNUN	-0.613	-0.018	0.428	-0.257	-0.041	-0.05	-0.082	-0.023	-0.037	0.088	-0.056	0.071	0.082	-0.014	-0.058	-0.037	0.018	0.028	0.015	-0.086	0.05	0.081	0.046	-0.011	-0.067	0.049	-0.035	0.031	0.001	-0.037	-0.016	0.008
T1C lbp 3D m2 glszm GrayLevelVariance	0.318	-0.067	-0.165	0.523	0.066	0.222	0.087	-0.026	0.141	-0.107	0.118	-0.199	-0.16	-0.007	0.002	-0.056	0.112	-0.166	0.056	0.068	-0.079	0.022	-0.019	0.014	0.122	0.02	0.069	-0.01	-0.026	0.167	0.029	0.049
T1C lbp 3D m2 glszm HGLZE	0.195	-0.141	-0.703	0.149	0.151	0.109	0.046	-0.043	0.044	0.06	0.014	-0.16	0.012	0.003	-0.13	0.061	-0.038	-0.023	-0.013	-0.008	0	-0.009	-0.041	0.038	0.02	-0.086	0.054	0.017	-0.056	0.019	0.065	0.026
T1C lbp 3D m2 glszm LALGLE	0.002	0.1	0.542	-0.124	0.051	-0.12	-0.058	-0.116	0.112	-0.072	-0.043	0.015	0.004	-0.059	-0.021	-0.037	0.3	0.071	0.199	-0.029	0.143	0.207	-0.125	0.083	-0.117	0.099	-0.022	-0.091	0.024	0.182	0.044	-0.004
T1C lbp 3D m2 glszm LGLZE	-0.229	0.071	0.67	0.024	-0.059	0.008	-0.032	-0.005	0.107	0.011	0.048	-0.014	-0.083	-0.026	0.04	-0.151	0.167	-0.106	0.106	-0.091	-0.061	0.135	0.033	0.032	0.039	0.121	-0.063	-0.017	0.02	0.145	-0.038	0.053
T1C lbp 3D m2 glszm SmallAreaEmphasis	-0.018	0.062	0.62	0.199	-0.086	0.164	-0.039	0.012	-0.076	0.074	0.048	-0.1	0.098	0.041	-0.094	0.015	-0.193	-0.142	-0.068	-0.205	-0.1	-0.121	-0.082	0.132	0.068	-0.045	-0.024	0.001	-0.058	0.005	-0.066	0.038
T1C lbp 3D m2 glszm SAHGLE	0.164	-0.087	-0.534	0.238	0.082	0.215	0.056	-0.047	0.001	0.107	0.021	-0.257	0.033	-0.011	-0.207	0.09	-0.063	-0.077	-0.021	-0.114	-0.005	-0.091	-0.077	0.052	0.069	-0.154	0.07	0.039	-0.071	0.035	0.12	0.095
T1C lbp 3D m2 glszm SALGLE	-0.282	0.065	0.62	0.113	-0.065	0.033	-0.042	0.024	0.145	0.048	0.022	-0.042	-0.081	0.022	0.036	-0.132	0.085	-0.175	0.08	-0.082	-0.111	0.12	0.044	0.011	0.056	0.107	-0.104	0.055	0.033	0.158	-0.043	0.085
T1C lbp 3D m2 glszm ZoneEntropy	0.623	0.003	-0.484	-0.006	0.117	-0.066	0.033	0.01	0.158	-0.031	0.001	0.068	-0.034	0.014	0.02	-0.014	0.033	0.011	0.028	0.077	0.04	0.088	0.014	-0.034	-0.021	0.021	-0.013	-0.005	0.016	0.009	0.024	-0.012
T1C lbp 3D m2 gldm DependenceVariance	0.222	-0.158	-0.648	-0.007	0.147	0.031	-0.094	-0.025	0.019	-0.127	-0.08	0.024	-0.035	0.131	-0.003	-0.043	-0.045	-0.068	0.111	0.204	0.021	0.12	-0.025	0.065	-0.077	-0.033	0.069	-0.068	0.044	0.098	0.054	-0.004
T1C lbp 3D m2 gldm LDLGLE	-0.024	0.1	0.639	-0.081	0.005	-0.117	-0.024	-0.097	0.046	-0.052	0.014	-0.016	0.011	-0.05	-0.029	-0.02	0.293	0.145	0.145	-0.075	0.124	0.155	-0.094	0.06	-0.079	0.074	-0.01	-0.122	0.032	0.134	0.026	0.001
T1C lbp 3D m2 ngtdm Complexity	0.337	0.044	0.392	0.403	0.06	0.099	0.108	0.053	-0.024	-0.075	0.151	-0.11	-0.02	-0.053	0.007	-0.092	0.109	-0.022	0.114	-0.004	-0.088	0.1	0.01	0.19	0.073	0.023	-0.178	-0.045	0.083	-0.051	-0.102	0.158
T1C lbp 3D m2 ngtdm Contrast	0.023	-0.094	0.52	0.455	0.028	0.145	0.078	-0.06	0.196	0.059	0.135	-0.086	0.056	-0.023	-0.028	-0.017	0.129	-0.091	-0.094	-0.119	-0.01	0.013	-0.071	-0.04	0.025	0.101	0.063	-0.134	-0.028	0.079	-0.065	-0.042
T1C lbp 3D k firstorder 10Percentile	0.28	-0.14	0.235	0.476	0.096	-0.001	0.07	0.07	-0.113	0.016	-0.002	0.039	-0.075	0.18	0.084	0.015	-0.115	0.02	0.035	0.059	0.034	-0.119	0.05	-0.062	0.151	-0.018	0.066	0.325	0.017	-0.009	-0.036	-0.049
T1C lbp 3D k firstorder 90Percentile	0.156	-0.22	0.142	0.306	0.078	0.655	-0.013	0.019	-0.055	0.003	0.07	-0.052	0.004	0.045	0.047	0.018	-0.051	-0.035	-0.05	-0.003	0.027	-0.049	-0.013	-0.049	0.104	-0.027	0.016	0.137	0.013	0.045	-0.046	0.051
T1C lbp 3D k firstorder Maximum	0.378	-0.1	0.049	0.17	0.11	0.352	-0.026	-0.009	0.051	0.001	0.562	-0.005	0.034	0.013	0.02	-0.061	-0.027	0.005	-0.079	0.001	-0.003	0.068	0.025	-0.006	0.09	0.048	-0.036	-0.002	0.004	0.013	0.012	0.026
T1C lbp 3D k firstorder Minimum	-0.417	-0.084	-0.015	0.058	-0.099	0.015	0.056	-0.018	-0.125	0.024	-0.112	0.028	-0.15	0.188	0.098	0.014	0.032	-0.01	0.215	-0.187	-0.035	-0.199	-0.062	-0.159	0.065	0.098	0.285	0.218	-0.097	0.049	0.127	-0.041
T1C lbp 3D k firstorder RMAD	0.02	-0.145	0.085	0.158	0.057	0.755	-0.007	-0.043	-0.027	-0.015	0.042	-0.044	0.037	0.004	0.043	0.098	-0.047	-0.01	-0.087	-0.033	0.003	-0.007	-0.05	-0.032	0.083	0.016	-0.064	0.021	0.018	0.039	-0.074	0.07

T1C feature name	T1C F1	T1C F2	T1C F3	T1C F4	T1C F5	T1C F6	T1C F7	T1C F8	T1C F9	T1C F10	T1C F11	T1C F12	T1C F13	T1C F14	T1C F15	T1C F16	T1C F17	T1C F18	T1C F19	T1C F20	T1C F21	T1C F22	T1C F23	T1C F24	T1C F25	T1C F26	T1C F27	T1C F28	T1C F29	T1C F30	T1C F31	T1C F32
T1C lbp 3D k firstorder Skewness	0.211	-0.059	0.073	0.064	0.002	0.21	0.036	0.051	-0.079	-0.01	0.697	0.012	-0.054	0.055	-0.113	-0.087	0.115	-0.079	-0.024	-0.024	-0.051	0.044	0.051	0.065	0.043	0.087	0.044	-0.04	0.004	0.019	0.039	-0.047
T1C lbp 3D k glcm ClusterShade	0.096	-0.114	0.009	0.15	-0.03	0.537	0.001	0.056	-0.174	-0.021	0.5	-0.034	0.013	-0.049	0.052	0.005	0.052	0.02	-0.097	-0.12	-0.041	-0.061	-0.027	-0.002	-0.035	-0.026	-0.014	-0.013	0.029	0.042	-0.032	-0.117
T1C lbp 3D k glcm Contrast	-0.136	-0.118	0.025	0.145	0.002	0.757	-0.008	0.046	-0.053	0.011	0.15	-0.016	0.104	0.023	-0.026	-0.067	-0.003	-0.016	-0.034	-0.024	-0.027	-0.013	-0.038	-0.03	-0.045	0.012	0.01	-0.016	-0.011	-0.057	0.011	-0.074
T1C lbp 3D k glcm Correlation	0.494	-0.209	-0.019	-0.099	-0.079	0.104	-0.043	-0.007	-0.056	-0.064	0.354	-0.045	-0.081	-0.098	0.162	-0.064	0.027	-0.06	-0.096	-0.186	0.007	-0.086	0.087	0.002	0.034	-0.075	-0.152	0.081	0.017	0.124	-0.176	0.027
T1C lbp 3D k glcm DifferenceAverage	-0.23	-0.089	-0.079	0.053	-0.015	0.746	-0.021	0.049	-0.003	0.022	0.11	-0.034	0.093	0.004	-0.07	-0.05	0.039	0.004	-0.016	-0.028	-0.055	-0.01	-0.035	-0.031	-0.071	0.017	0.019	-0.135	0	-0.017	-0.003	-0.06
T1C lbp 3D k glcm Imc2	-0.471	-0.226	0.249	0.009	-0.127	0.357	-0.053	-0.03	-0.194	-0.022	0.287	-0.07	0.073	-0.046	-0.02	-0.03	-0.011	-0.087	-0.099	-0.057	0.029	-0.086	0.073	-0.017	-0.024	-0.117	-0.04	0	-0.083	-0.082	-0.073	-0.007
T1C lbp 3D k glcm Idmn	0.629	-0.088	0.139	0.08	0.057	0.142	-0.009	-0.016	0.078	0.01	0.386	-0.011	-0.012	0.028	0.084	-0.043	0.004	-0.058	-0.059	0.014	0.035	0.045	0.057	0.034	0.089	-0.03	0.013	0.102	-0.06	-0.083	-0.02	0.127
T1C lbp 3D k glcm InverseVariance	-0.335	0.071	-0.226	-0.18	-0.074	0.342	-0.103	-0.004	0.062	0.008	-0.032	0.027	0.077	-0.044	-0.182	0.046	0.1	0.083	-0.015	0.088	-0.096	0.091	-0.019	0.028	-0.099	-0.068	0.014	-0.435	0.06	0.025	-0.092	-0.08
T1C lbp 3D k glcm MaximumProbability	0.346	0	0.212	0.259	0.072	-0.48	0.068	-0.014	-0.042	0.001	-0.044	-0.034	-0.133	0.03	0.142	0.087	-0.063	-0.028	0.031	0.035	0.104	-0.061	-0.013	-0.014	0.136	0.027	0.031	0.34	0.002	-0.018	0.045	0.089
T1C lbp 3D k glrlm GLNUN	-0.121	0.144	0.375	-0.083	-0.128	-0.653	0.023	0.005	-0.087	0.075	-0.06	0.049	-0.014	0.035	-0.02	0.076	-0.023	0.092	0.027	0.027	-0.006	0.024	0.001	-0.032	-0.073	0.027	-0.013	0.108	0.013	-0.03	0.01	-0.095
T1C lbp 3D k glrlm LRLGLE	0.095	0.006	-0.669	-0.13	0.187	-0.254	-0.075	-0.077	0.169	-0.085	-0.106	0.11	0.009	-0.022	0.015	-0.081	-0.033	-0.022	0.008	0.017	0.005	0.144	0.01	0.023	-0.064	0.034	-0.083	-0.07	-0.078	-0.001	0.02	-0.013
T1C lbp 3D k glrlm RunEntropy	0.434	-0.118	-0.59	0.155	0.205	0.135	-0.001	-0.03	0.099	-0.067	0.019	0.032	-0.076	0.009	0.085	-0.067	-0.003	-0.05	0.017	-0.009	0.02	0.023	-0.001	-0.016	0.064	0.048	-0.031	0.112	-0.022	0.042	-0.007	0.048
T1C lbp 3D k glrlm ShortRunEmphasis	-0.227	0.049	0.658	-0.091	-0.154	0.306	-0.01	0.046	-0.076	0.074	0.098	-0.082	0.082	-0.024	-0.109	0.034	0.061	-0.008	-0.033	-0.026	-0.03	-0.052	-0.004	0.005	-0.042	-0.019	0.057	-0.131	0.041	-0.005	-0.015	-0.016
T1C lbp 3D k glrlm SRHGLE	0.143	-0.169	0.358	0.265	0.034	0.595	0.031	0.036	-0.115	0.018	0.168	-0.052	-0.012	0.051	-0.002	0.00E+00	0.018	-0.059	-0.012	-0.022	-0.013	-0.064	-0.021	-0.054	0.074	-0.011	0.05	0.148	0.041	0.01	-0.021	-0.006
T1C lbp 3D k glrlm SRLGLE	-0.351	0.228	-0.162	-0.423	-0.108	-0.263	-0.058	-0.029	0.104	-0.015	-0.065	0.008	0.083	-0.141	-0.049	-0.019	0.059	0.045	-0.019	-0.033	0.001	0.07	0.028	0.104	-0.132	0.008	-0.032	-0.364	-0.012	-0.012	0.025	-0.026
T1C lbp 3D k glszm GLNU	0.429	0.059	0.102	0.192	0.057	-0.043	0.126	0.072	0.614	0.03	-0.079	-0.02	0.083	0.041	-0.018	0.022	0.024	-0.018	-0.034	-0.005	-0.084	-0.078	-0.106	0.006	0.044	-0.011	0.067	-0.032	0.008	0.046	0.057	0.009
T1C lbp 3D k glszm GLNUN	-0.074	0.296	-0.166	-0.075	-0.09	-0.438	-0.031	-0.076	0.075	-0.005	-0.32	0.156	0.12	-0.032	-0.154	0.042	-0.205	0.023	0.023	0.015	-0.159	0.125	-0.024	0.006	-0.069	-0.051	-0.14	-0.113	0.024	0.021	0.055	-0.029
T1C lbp 3D k glszm GrayLevelVariance	0.323	-0.052	0.236	0.164	0.134	0.309	0.046	0.079	-0.085	0.012	0.484	-0.097	-0.073	-0.015	0.155	-0.026	0.149	-0.044	-0.11	-0.062	0.089	-0.097	0.026	-0.018	0.099	-0.078	0.081	0.124	0.06	0.025	0.004	-0.042
T1C lbp 3D k glszm HGLZE	0.381	-0.097	-0.345	0.088	0.147	0.456	-0.025	-0.052	-0.035	-0.04	0.232	0.04	-0.03	-0.02	-0.02	-0.031	0.136	0.004	0.041	-0.032	-0.045	0.247	-0.004	0.041	0.019	0.021	-0.145	0.052	0.055	-0.03	0.04	-0.01
T1C lbp 3D k glszm LGLZE	-0.1	0.042	0.559	0.013	-0.032	-0.285	0.062	0.116	-0.009	0.069	0.003	-0.083	-0.019	-0.02	0.157	0.009	-0.031	0.006	-0.132	-0.057	0.167	-0.321	0.026	-0.044	0.047	-0.034	0.229	0.066	-0.012	0.073	-0.028	-0.001
T1C lbp 3D k glszm SZNU	0.435	-0.024	0.156	0.212	0.136	0.022	0.151	0.07	0.557	0.025	0.001	-0.038	0.055	0.044	0.041	0.033	0.083	-0.029	-0.054	-0.001	-0.083	0.047	-0.104	0.008	0.046	0.028	0.11	-0.003	0.016	0.104	0.029	0.02
T1C lbp 3D k glszm SZNUN	0.188	-0.066	0.148	-0.091	0.053	-0.275	0.005	-0.056	0.042	0.037	0.054	0.13	0.073	-0.001	0.068	0.019	0.117	-0.028	0.011	-0.157	0.056	0.63	-0.021	0.141	-0.059	0.03	0.037	-0.063	-0.03	0.009	-0.015	-0.026
T1C lbp 3D k glszm SAHGLE	0.509	-0.08	-0.108	0.024	0.114	0.2	-0.031	-0.044	-0.042	-0.018	0.273	0.072	-0.028	-0.043	0.048	0.034	0.171	-0.018	-0.007	-0.035	0.017	0.456	-0.034	0.077	0.043	0.002	-0.124	0.022	0.027	-0.032	0.016	0.006
T1C lbp 3D k glszm SALGLE	0.362	-0.002	0.468	0.005	-0.014	-0.254	0.052	0.092	-0.045	0.054	-0.018	-0.039	0.1	0.04	0.05	-0.05	-0.064	-0.079	-0.063	-0.094	0.118	-0.058	0.049	0.098	-0.057	-0.14	0.371	-0.017	-0.016	-0.019	-0.053	-0.094
T1C lbp 3D k glszm ZoneEntropy	0.524	-0.071	0.025	0.181	0.055	0.405	0.057	0.122	0.135	0.031	0.168	-0.131	-0.082	0.043	0.007	-0.037	0.027	-0.133	-0.048	0.027	-0.006	-0.269	0.027	0	0.047	0.017	0.061	0.084	-0.041	0.023	-0.029	0.03
T1C lbp 3D k glszm ZonePercentage	-0.189	-0.085	0.657	0.14	-0.091	0.269	0.023	0.029	-0.096	-0.013	0.06	-0.041	0.055	0.082	-0.073	0.032	-0.118	-0.148	-0.062	0.06	-0.024	-0.05	-0.065	0.031	0.069	-0.015	0.101	0.048	-0.061	-0.02	-0.002	0.029
T1C lbp 3D k gldm DependenceEntropy	0.618	-0.096	-0.43	0.081	0.08	0.108	-0.025	-0.011	0.055	-0.078	0.009	0.002	-0.061	0.01	0.049	-0.039	-0.052	-0.11	0.003	0.034	0.007	-0.092	-0.01	-0.01	0.062	0.112	0.005	0.098	-0.017	0.081	-0.058	0.081
T1C lbp 3D k gldm DependenceVariance	0.404	-0.105	-0.452	0.233	0.248	-0.156	-0.037	-0.082	0.096	-0.124	-0.083	0.021	-0.116	-0.021	0.099	-0.02	-0.048	-0.067	0.044	0.074	0.048	-0.041	0.009	-0.003	0.126	0.122	0.076	0.159	-0.062	-0.008	0.003	0.071
T1C lbp 3D k gldm SDE	-0.009	-0.065	0.714	0.091	-0.09	0.265	0.008	0.026	-0.117	-0.004	0.058	-0.063	0.072	0.051	-0.077	0.071	-0.068	-0.107	-0.036	0.048	-0.026	0.029	-0.068	0.044	0.041	-0.045	0.101	0.008	-0.029	-0.049	-0.013	0.027
T1C lbp 3D k gldm SDHGLE	0.073	-0.081	0.559	0.175	-0.054	0.434	0.012	0.015	-0.144	-0.024	0.2	-0.034	0.047	0.047	-0.069	0.063	0.014	-0.129	-0.007	0.028	-0.03	0.135	-0.076	0.037	0.061	-0.045	0.037	0.078	0.011	-0.029	-0.004	0.026
T1C lbp 3D k gldm SDLGLE	0.009	-0.02	0.755	0.02	-0.098	0.079	0.012	0.043	-0.09	0.02	-0.021	-0.09	0.065	0.022	-0.026	0.063	-0.098	-0.058	-0.091	0.047	0.009	-0.094	-0.041	0.06	0.031	-0.052	0.162	-0.046	-0.044	-0.061	-0.011	0.001

T1C feature name	T1C F1	T1C F2	T1C F3	T1C F4	T1C F5	T1C F6	T1C F7	T1C F8	T1C F9	T1C F10	T1C F11	T1C F12	T1C F13	T1C F14	T1C F15	T1C F16	T1C F17	T1C F18	T1C F19	T1C F20	T1C F21	T1C F22	T1C F23	T1C F24	T1C F25	T1C F26	T1C F27	T1C F28	T1C F29	T1C F30	T1C F31	T1C F32
T1C lbp 3D k ngtdm Busyness	0.286	0.11	-0.346	-0.039	0.065	-0.18	-0.026	0.053	0.46	0.028	-0.235	0.142	0.039	-0.068	-0.108	-0.001	-0.048	-0.035	-0.016	0.022	-0.03	0.065	0.005	0.017	-0.117	0.008	-0.182	-0.096	-0.02	-0.023	-0.002	-0.129
T1C lbp 3D k ngtdm Complexity	0.27	0.009	0.018	0.163	0.076	0.369	-0.011	-0.036	0.061	-0.008	0.6	-0.046	0.02	-0.009	0.088	-0.045	0.017	0.009	-0.059	0.007	-0.042	0.084	0.055	0.011	0.151	0.051	-0.029	-0.021	0.033	0.024	-0.005	0.078
T1C lbp 3D k ngtdm Strength	-0.302	-0.059	0.267	0.112	-0.076	0.275	-0.062	-0.044	-0.22	-0.031	0.534	0.041	0.018	-0.033	-0.027	0.051	-0.057	-0.094	-0.076	0.076	-0.003	0.038	0.011	0.057	0.066	-0.093	-0.035	-0.022	-0.087	-0.105	0.029	0.02

Table S13 Factor loadings for the 27 PF factors. The number of each feature reflect the importance weight in the factor in which it is present. In bold…*** Abbreviations: XXX

Pathomic feature name	P F1	P F2	P F3	P F4	P F5	P F6	P F7	P F8	P F9	P F10	P F11	P F12	P F13	P F14	P F15	P F16	P F17	P F18	P F19	P F20	P F21	P F22	P F23	P F24	P F25	P F26	P F27
MEAN Nucleus Area	0.15	0.153	-0.071	0.072	-0.267	-0.115	0.074	0.161	-0.181	-0.077	-0.027	-0.032	-0.674	0.044	0.019	-0.022	-0.04	0.032	-0.004	0.025	-0.009	-0.008	-0.007	0.027	0.018	-0.007	0.006
MEAN Nucleus Perimeter	0.218	0.053	-0.038	0.109	-0.284	-0.06	0.07	0.141	-0.132	-0.069	-0.019	-0.067	-0.646	-0.019	0.00E+00	-0.014	-0.024	0.217	0.011	0.035	0.005	-0.031	-0.008	0.033	0.031	-0.002	0.007
MEAN Nucleus Circularity	-0.262	0.306	-0.024	-0.107	0.145	-0.178	-0.031	-0.017	-0.034	-0.043	0.013	0.124	0.169	0.062	0.047	-0.059	-0.033	-0.584	-0.063	-0.048	-0.022	0.038	-0.012	-0.031	-0.02	-0.001	0.011
MEAN Nucleus MinCaliper	0.032	0.211	-0.045	-0.029	-0.233	-0.167	0.062	0.182	-0.106	-0.072	-0.112	-0.052	-0.676	0.044	0.026	-0.021	-0.058	-0.065	0.014	0.051	-0.011	-0.004	0.016	-0.019	-0.018	-0.009	-0.028
MEAN Nucleus Eccentricity	0.356	-0.371	-0.004	0.157	-0.251	0.244	0.045	-0.072	-0.11	-0.017	0.12	-0.087	0.011	-0.071	-0.068	0.02	0.014	0.411	0.036	-0.046	-0.063	-0.059	0.011	0.057	0.051	-0.025	0.055
MEAN Nucleus HematoxylinODSum	0.323	0.082	0.054	0.129	-0.171	0.369	0.064	0.081	-0.029	0.047	0.001	0.011	-0.205	0.554	0.085	0.067	-0.028	-0.05	-0.001	-0.038	-0.025	0.045	-0.027	0.016	-0.009	0.009	0.009
MEAN Nucleus HematoxylinODStdDev	0.213	-0.034	-0.013	0.059	-0.1	0.506	0.036	-0.064	-0.013	0.024	-0.022	0.027	0.229	0.479	0.075	0.12	0.018	0.055	-0.073	-0.039	-0.085	0.071	-0.004	0.076	0.022	-0.069	-0.027
MEAN Nucleus HematoxylinODMin	0.296	-0.012	0.196	0.133	-0.094	0.278	0.013	0.008	-0.041	0.065	-0.021	0.06	-0.006	0.604	0.136	0.015	0.041	-0.149	0.059	-0.029	0.041	-0.022	0.045	-0.011	-0.029	0.052	0.025
MEAN Nucleus EosinODMean	-0.008	-0.078	0.073	-0.024	-0.017	0.258	0.067	0.747	-0.006	-0.012	0.093	0.058	0.002	-0.092	-0.015	-0.062	-0.005	-0.02	0.04	0.036	0.027	-0.009	-0.02	-0.013	-0.034	-0.03	0.015
MEAN Nucleus EosinODSum	0.083	0.031	0.009	0.004	-0.087	0.214	0.084	0.711	-0.061	-0.06	0.033	0.011	-0.266	-0.055	0.004	-0.053	-0.051	-0.001	0.045	0.035	0.037	0.002	-0.027	-0.034	-0.034	-0.059	0.017
MEAN Nucleus EosinODStdDev	-0.107	-0.024	-0.095	-0.078	0.07	0.705	0	0.066	0.101	0.051	0.051	0.069	0.237	0.018	0.012	-0.028	0.03	-0.023	0.023	0.077	-0.005	0.1	0.007	0.038	-0.069	0.034	-0.039
MEAN Nucleus EosinODMin	0.031	-0.083	0.114	0.02	-0.016	-0.038	0.082	0.781	-0.046	-0.043	0.09	0.034	-0.058	-0.106	-0.011	-0.054	-0.002	-0.009	0.011	-0.004	0.014	-0.059	0.004	-0.011	-0.012	-0.04	0.033
MEAN Nucleus EosinODRange	-0.046	-0.029	-0.054	-0.083	0.003	0.736	0.03	0.136	0.107	0.056	0.029	0.02	0.154	0.077	-0.008	-0.005	0.01	0.017	0.029	0.073	-0.022	0.108	-0.037	0.035	-0.029	0.026	-0.06
MEAN Cell MaxCaliper	-0.087	0.218	-0.647	0.066	-0.059	-0.049	0.039	0.033	-0.092	0.078	0.038	0.253	-0.269	-0.033	-0.043	0.018	0.009	0.045	0.017	0.016	0.004	0.008	-0.1	0.085	0.067	-0.008	0.046
MEAN Cell Eccentricity	0.334	-0.347	0.308	0.06	-0.433	0.067	0.067	0.094	-0.113	-0.084	0.017	-0.173	-0.191	-0.056	-0.034	-0.068	-0.005	0.185	0.065	0.008	-0.066	-0.035	0.017	0.021	0.025	-0.008	0.017
MEAN Cell HematoxylinODStdDev	0.274	-0.044	0.116	0.087	-0.137	0.504	0.043	0.058	-0.008	0.012	0.002	-0.031	0.072	0.503	0.106	0.061	-0.02	-0.031	-0.059	-0.052	-0.086	0.083	0.002	0.046	-0.012	0	-0.025
MEAN Cell EosinODMean	0.106	-0.115	0.116	0.033	-0.04	0.125	0.106	0.777	-0.025	0.008	0.075	0.012	-0.035	0.015	0.038	-0.034	0.012	0.01	-0.013	0.006	-0.002	0.01	0.012	-0.002	0.01	0.021	-0.015
MEAN Cell EosinODStdDev	-0.118	-0.117	-0.07	-0.104	0.016	0.644	-0.016	0.319	0.067	0.056	0.061	-0.028	0.033	-0.166	-0.026	-0.058	0.055	0.16	0.044	0.09	0.018	0.067	0.052	-0.046	-0.003	0.114	-0.031
MEAN Cell EosinODMax	0.013	-0.1	0.031	-0.02	0.008	0.402	0.086	0.689	0.048	0.019	0.075	-0.002	0.041	-0.019	0.012	-0.032	0.017	0.046	-0.004	0.021	-0.037	0.055	0.007	0.023	0.011	0.067	-0.038
MEAN Cytoplasm HematoxylinODMean	0.297	-0.041	0.363	0.139	-0.066	0.069	0.028	-0.037	0.014	0.087	-0.11	0.009	-0.095	0.543	0.017	0.024	0.051	0.02	0.118	0.018	0.11	-0.118	-0.007	-0.045	0.012	0.036	0.015
MEAN Cytoplasm HematoxylinODStdDev	0.252	-0.122	0.259	0.073	-0.103	0.435	0.073	0.287	0.044	0.001	-0.048	-0.136	-0.035	0.367	0.098	0.036	-0.004	0.192	-0.047	-0.06	-0.004	0.011	-0.016	-0.003	0.029	0.062	-0.056

Pathomic feature name	P F1	P F2	P F3	P F4	P F5	P F6	P F7	P F8	P F9	P F10	P F11	P F12	P F13	P F14	P F15	P F16	P F17	P F18	P F19	P F20	P F21	P F22	P F23	P F24	P F25	P F26	P F27
MEAN Cytoplasm HematoxylinODMax	0.281	-0.066	0.197	0.103	-0.113	0.422	0.05	0.023	0.022	0.034	-0.091	-0.043	0.009	0.569	0.037	0.074	0.003	0.083	0	-0.027	-0.038	-0.009	-0.017	0.019	0.018	-0.013	-0.018
MEAN Cytoplasm HematoxylinODMin	0.096	0.044	0.113	0.112	-0.018	-0.179	-0.092	-0.554	-0.062	0.019	-0.086	0.138	-0.026	0.334	-0.045	-0.006	0.018	-0.183	0.118	0.031	0.065	-0.151	0.042	-0.016	-0.074	-0.1	0.1
MEAN Cytoplasm EosinODStdDev	-0.031	-0.174	-0.048	-0.071	-0.017	0.528	0.022	0.439	0.038	0.111	0.072	-0.041	-0.017	-0.059	0.021	-0.033	0.107	0.246	-0.006	0.064	0.047	0.058	0.079	-0.087	0.062	0.139	-0.051
MEAN Cytoplasm EosinODMin	0.162	-0.082	0.175	0.097	0.005	-0.082	0.157	0.727	-0.04	-0.043	0.046	0.072	-0.022	0.068	0.037	-0.032	-0.009	-0.072	-0.049	-0.029	-0.07	-0.04	0.033	0.041	-0.04	-0.021	0.039
MEAN Nucleus CellAreaRatio	0.244	-0.076	0.508	0.05	-0.245	-0.064	0.039	0.105	-0.081	-0.154	-0.05	-0.263	-0.392	-0.018	0.035	-0.047	-0.045	0.054	-0.016	-0.004	-0.02	-0.037	0.066	-0.049	-0.006	-0.006	-0.026
MEAN Delaunay MaxTriangleArea	-0.174	0.39	-0.176	0.131	0.599	-0.102	-0.028	-0.107	0.1	-0.001	0.027	0.141	0.046	-0.017	0.056	0.008	0.035	-0.038	-0.134	-0.006	0.072	-0.029	-0.042	-0.032	-0.009	0.063	0.04
MEAN ODSum Std dev	0.09	-0.004	0	-0.041	-0.117	0.753	-0.022	0.077	0.004	-0.056	0.044	-0.031	-0.074	0.186	0.027	0.028	-0.015	0.005	0.038	0.019	0.018	0.015	0.012	-0.024	-0.07	-0.037	0.002
MEAN ODSum Min	0.204	-0.091	0.26	0.115	-0.04	-0.058	0.123	0.697	-0.014	0.026	0.027	0.016	-0.027	0.174	0.03	-0.029	0.007	-0.054	-0.001	-0.002	0.002	-0.014	-0.002	0.012	0.017	0.029	-0.001
MEAN ODSum Max	0.18	-0.045	0.138	0.056	-0.098	0.428	0.081	0.586	0.006	0.007	0.057	0.022	-0.046	0.263	0.041	0.01	-0.003	-0.055	0.024	0.006	0.019	0.012	-0.024	0.002	-0.014	0.006	-0.012
MEAN ODSum Haralick ASM F0	-0.006	-0.026	0.261	0.037	0.383	-0.55	-0.013	-0.096	0.132	0.159	-0.041	-0.101	0.132	-0.005	-0.025	0.003	0.007	0.039	0.069	0.082	0.022	0.207	0.065	-0.008	-0.098	0.062	-0.057
MEAN ODSum HaralickCorrelation F2	-0.095	0.159	-0.595	-0.036	-0.09	0.165	0.028	0.038	-0.194	-0.024	-0.035	0.204	-0.303	0.009	0.077	-0.04	0.018	-0.016	0.074	-0.038	-0.021	-0.106	0.102	0.024	-0.043	-0.082	0.148
MEAN ODSum Haralick IDM	-0.14	0.07	-0.195	0.024	0.232	-0.685	0.034	-0.147	0.045	0.162	-0.031	0.073	0.022	-0.093	-0.042	0	0.02	-0.025	0.018	0.042	-0.035	0.079	-0.003	0.031	0	0.039	-0.035
MEAN ODSum HaralickSumAverage F5	0.216	-0.103	0.232	0.083	-0.083	0.176	0.103	0.675	-0.02	0.015	0.037	0.003	-0.071	0.219	0.054	-0.02	0.01	-0.008	0.013	-0.007	0.024	-0.016	0.006	-0.009	0.009	0.028	-0.006
MEAN ODSum Haralick DV F9	-0.008	-0.14	0.147	0.024	0.147	-0.72	0.04	-0.096	0.042	0.15	-0.05	-0.078	-0.049	-0.04	0.009	-0.031	0.049	0.104	0.054	0.029	-0.034	0.061	0.06	0.024	-0.025	0.058	-0.049
MEAN ODSum Haralick IMOC1 F11	-0.194	0.13	-0.336	0.017	0.187	-0.598	0.033	-0.112	0.054	0.172	-0.012	0.141	0.046	-0.093	-0.056	0.022	0.008	-0.064	-0.002	0.034	-0.023	0.082	-0.067	0.03	0.04	0.039	-0.04
MEAN ODSum Haralick IMOC2 F12	0.105	-0.027	0.072	-0.012	-0.301	0.625	-0.02	0.101	-0.127	-0.251	0.024	-0.038	-0.092	-0.006	0.034	-0.016	-0.012	-0.018	-0.076	-0.097	-0.004	-0.217	0.008	-0.016	0.043	-0.066	0.074
CD50um firstorder 10Percentile	0.162	-0.114	0.53	-0.391	-0.111	0.014	0.025	-0.014	0.015	-0.221	0.011	-0.362	-0.052	0.003	-0.007	-0.014	-0.021	0.053	0.005	-0.007	-0.022	-0.008	0.063	0.011	-0.007	0.041	-0.017
CD50um firstorder Kurtosis	-0.034	0.266	-0.277	0.055	0.483	-0.107	-0.003	-0.168	0.036	-0.034	0.077	0.185	0.104	0.065	-0.065	0.366	0.002	0.045	0.048	-0.077	-0.051	-0.179	-0.076	0.014	-0.001	0.039	0.056
CD50um firstorder Maximum	0.45	-0.198	0.574	-0.073	-0.167	-0.028	0.111	0.028	-0.002	0.108	0.023	-0.11	-0.065	0.027	0.015	-0.017	0.039	0.049	0.059	0.046	-0.054	0.039	0.027	-0.04	0.074	0.019	0.05
CD50um firstorder Minimum	-0.086	-0.046	0.284	-0.116	-0.028	0.06	-0.165	-0.03	0.035	-0.157	-0.011	-0.608	-0.188	-0.041	-0.004	0.106	-0.074	0.016	-0.025	-0.048	0.053	0.054	0.088	0.047	0.154	0.032	-0.094
CD50um firstorder Range	0.496	-0.187	0.482	-0.031	-0.162	-0.052	0.177	0.041	-0.015	0.17	0.027	0.12	0.006	0.044	0.017	-0.059	0.068	0.044	0.071	0.066	-0.075	0.019	-0.006	-0.059	0.015	0.008	0.087
CD50um firstorder Skewness	-0.146	0.149	-0.47	-0.22	0.334	-0.124	-0.066	-0.063	0.035	0.35	0.038	0.119	-0.007	-0.037	-0.084	0.198	0.013	0.004	0.06	0.003	-0.001	-0.005	-0.063	0.039	0.131	0.027	0.063
CD50um glcm ClusterProminence	0.087	-0.202	0.259	0.679	-0.014	-0.049	-0.03	0.08	0.004	0.19	-0.034	0.084	0.117	0.056	0.018	0.037	0.007	0.032	0.087	0.016	0.023	0.096	0.006	-0.082	-0.072	-0.025	-0.018
CD50um glcm Correlation	0.425	-0.108	0.239	0.244	0.039	-0.096	0.026	-0.044	-0.033	0.54	-0.116	0.086	0.039	0.055	0.054	-0.157	-0.057	0.044	-0.049	-0.013	-0.015	-0.029	-0.035	-0.038	0	-0.009	-0.03
CD50um glcm DifferenceAverage	0.126	-0.587	0.35	-0.142	-0.147	0.032	0.028	0.155	0.031	-0.094	0.011	0.004	-0.061	0.031	-0.069	-0.026	0.134	0.085	0.083	0.188	-0.086	0.039	0.111	-0.04	-0.101	0.024	0.045
CD50um glcm JointEnergy	-0.321	0.333	-0.102	0.486	0.392	-0.027	-0.054	-0.069	0.062	-0.077	0.002	0.07	0.101	-0.023	0.059	0.069	0.007	-0.068	-0.083	-0.059	0.081	-0.042	-0.019	-0.07	-0.016	0.015	0.026
CD50um glcm Imc2	-0.243	-0.44	0.131	0.141	0.026	-0.087	-0.051	-0.019	0.037	0.525	-0.112	0.111	0.101	0.054	0.049	-0.14	0.028	0.069	-0.081	-0.039	-0.029	-0.122	0.028	-0.041	-0.002	-0.026	-0.044
CD50um glcm Idm	-0.169	0.484	-0.177	0.388	0.348	-0.015	-0.06	-0.113	0.061	0.105	0.028	0.085	0.124	-0.011	0.045	0.022	-0.044	-0.066	-0.079	-0.143	0.098	-0.078	-0.065	-0.016	0.028	-0.033	-0.006
CD50um glcm Idn	0.632	0.186	0.226	0.098	0.043	-0.115	0.149	-0.111	-0.035	0.245	0.029	0.124	0.044	0.044	0.059	-0.043	-0.029	-0.036	0.004	-0.027	-0.057	-0.042	-0.066	-0.042	0.055	-0.02	0.097
CD50um glcm InverseVariance	-0.164	0.54	-0.313	-0.055	0.263	0.007	-0.063	-0.163	0.015	0.091	-0.023	0.082	0.13	0.011	-0.013	0.046	-0.099	-0.078	-0.097	-0.159	0.147	-0.059	-0.092	0.109	0.089	-0.092	-0.022
CD50um glcm MaximumProbability	-0.168	0.155	0.002	0.69	0.32	-0.052	-0.042	-0.047	0.06	-0.029	-0.031	0.064	0.072	-0.012	0.081	0.046	0.007	-0.051	-0.036	-0.04	0.029	-0.059	-0.003	-0.108	-0.038	0.007	0.032

**Pathomic feature name**	**P F1**	**P F2**	**P F3**	**P F4**	**P F5**	**P F6**	**P F7**	**P F8**	**P F9**	**P F10**	**P F11**	**P F12**	**P F13**	**P F14**	**P F15**	**P F16**	**P F17**	**P F18**	**P F19**	**P F20**	**P F21**	**P F22**	**P F23**	**P F24**	**P F25**	**P F26**	**P F27**
CD50um glrlm RunVariance	0.033	-0.049	0.127	0.802	-0.029	0.024	-0.015	0.043	-0.001	0.003	-0.02	0.018	0.06	0.013	0.033	0.038	-0.02	-0.044	0.009	-0.035	0.006	-0.009	-0.015	-0.074	-0.038	-0.028	0.011
CD50um glszm SALGLE	-0.468	0.406	-0.186	0.083	0.397	-0.043	-0.097	-0.026	0.146	-0.004	0.044	0.1	0.056	-0.039	0.011	0.056	0.045	-0.024	-0.132	-0.03	0.143	0.001	-0.027	0.03	0.03	0.061	-0.002
CD50um gldm LDLGLE	-0.049	-0.021	0.112	0.804	0.03	0.019	-0.024	0.041	-0.003	-0.003	-0.025	0.036	0.063	0.003	0.034	0.046	-0.009	-0.042	-0.005	-0.029	0.022	-0.01	-0.011	-0.073	-0.032	-0.024	0.011
CD50um ngtdm Busyness	0.208	0.41	0.079	-0.04	-0.075	0.029	0.625	0.107	-0.049	-0.085	0.043	0.065	-0.102	0.003	0.001	-0.019	-0.006	-0.017	0.038	0.066	-0.02	0.095	-0.039	0.04	-0.013	-0.03	0.032
CD50um ngtdm Complexity	0.377	-0.218	0.56	-0.039	-0.08	-0.017	0.199	0.145	-0.011	0.089	0.04	0.17	0.046	0.083	-0.052	-0.013	0.114	0.039	0.03	0.086	-0.024	0.023	0.005	-0.018	-0.084	0.028	0.087
CD50um ngtdm Contrast	-0.32	-0.523	0.194	0.339	-0.123	0.051	-0.137	0.12	0.089	0.153	-0.062	-0.039	0.022	0.01	0.019	-0.085	0.103	0.042	0.022	0.103	0.004	0.043	0.095	-0.065	-0.055	-0.031	-0.078
CD100um firstorder Kurtosis	0.159	0.251	-0.18	0.051	0.327	0.013	0.021	-0.144	0.042	-0.05	-0.01	0.026	0.042	0.059	-0.053	0.628	-0.017	0.049	0.071	-0.019	-0.02	-0.058	-0.016	-0.009	-0.002	0.04	0
CD100um firstorder Maximum	0.446	-0.202	0.56	-0.034	-0.162	-0.012	0.114	0.057	0.002	0.114	-0.057	-0.184	-0.043	0.081	0.034	0.098	0	0.073	-0.001	0.049	0.002	0.023	0.004	-0.009	-0.03	0.046	0.064
CD100um firstorder Minimum	0.006	-0.094	0.081	-0.224	-0.124	-0.022	-0.127	-0.077	0.084	-0.161	-0.019	-0.675	-0.004	0.097	0.034	-0.006	0.017	-0.026	0.002	0.062	-0.053	0.112	-0.068	-0.029	0.083	-0.021	0.116
CD100um firstorder Range	0.457	-0.162	0.538	0.073	-0.107	-0.001	0.179	0.096	-0.038	0.195	-0.051	0.14	-0.043	0.036	0.019	0.103	-0.009	0.088	-0.001	0.019	0.029	-0.031	0.038	0.006	-0.072	0.058	0.008
CD100um firstorder Uniformity	-0.382	0.412	-0.287	0.115	0.433	-0.076	-0.049	-0.085	0.085	-0.151	0.031	0.053	0.077	-0.035	0.037	0.13	0.01	-0.044	-0.024	-0.005	0.05	-0.002	-0.019	-0.051	-0.001	0.036	0.024
CD100um glcm Correlation	0.459	0.156	0.184	0.181	0.116	-0.174	0.076	-0.026	-0.209	0.418	-0.164	-0.011	-0.048	0.004	0.001	-0.212	-0.096	0.012	-0.068	-0.011	-0.086	0.06	-0.029	0.006	0.003	0.056	0.029
CD100um glcm DifferenceAverage	-0.074	-0.656	0.334	0.022	-0.061	0.143	-0.025	0.026	0.101	0.046	0.043	0.112	-0.009	0.07	-0.013	-0.042	0.161	0.049	0.007	0.135	0.031	-0.069	0.097	-0.056	-0.107	-0.021	-0.002
CD100um glcm DifferenceEntropy	0.2	-0.664	0.326	0.022	-0.168	0.108	-0.001	0.036	0.054	0.05	0.022	0.067	-0.06	0.062	-0.02	-0.023	0.065	0.083	0.059	0.091	-0.008	-0.067	0.037	-0.025	-0.062	-0.034	-0.046
CD100um glcm DifferenceVariance	0.001	-0.545	0.401	0.293	0.104	0.084	-0.053	0.048	0.075	0.098	0.009	0.105	0.066	0.098	-0.097	0.031	0.142	0.013	0.028	0.135	0.02	-0.047	0.089	-0.005	-0.115	-0.054	0.007
CD100um glcm Imc2	-0.318	-0.459	0.067	0.067	0.067	-0.106	-0.198	0.02	0.113	0.36	-0.137	-0.016	0.133	0.028	-0.013	-0.137	0.03	0.064	-0.168	-0.119	0.024	-0.172	0.072	0.052	0.055	0.007	-0.054
CD100um glcm Idm	0.091	0.493	-0.206	0.294	0.424	-0.149	0.001	-0.1	0.04	-0.038	-0.069	-0.016	0.061	0.007	-0.093	0.032	-0.144	-0.016	-0.067	-0.035	-0.095	-0.025	-0.092	0.072	0.038	-0.04	0.067
CD100um glcm Idn	0.658	0.256	0.2	0.059	0.131	-0.112	0.12	0.009	-0.128	0.12	-0.13	0.009	-0.086	0.008	-0.027	0.108	-0.104	0	-0.024	0.012	-0.075	0.001	-0.004	0.041	-0.011	0.087	0.056
CD100um glcm InverseVariance	-0.077	0.642	-0.17	0.036	0.383	-0.089	0.001	-0.038	-0.054	-0.059	-0.106	-0.045	0.044	-0.047	0.017	0.11	-0.018	-0.127	-0.081	-0.057	0.033	0.035	-0.042	0.022	0.04	0.05	-0.048
CD100um glcm MaximumProbability	-0.332	0.069	-0.061	0.691	0.159	-0.118	-0.069	-0.03	0.046	-0.009	-0.041	0.1	0.013	0.026	-0.042	0.023	0.054	0.099	-0.012	0.053	0.022	0.006	-0.027	0.077	0.017	-0.01	-0.035
CD100um glcm SumEntropy	0.51	-0.228	0.435	-0.004	-0.174	-0.046	0.057	0.079	-0.086	0.28	-0.09	0.024	-0.028	0.019	-0.007	-0.184	-0.026	0.044	-0.059	0.008	-0.049	0.008	0.005	0.017	-0.007	0.034	-0.005
CD100um glrlm RunVariance	0.125	0.052	-0.039	0.656	-0.02	-0.141	-0.018	-0.043	0.022	0.005	0.048	0.111	-0.108	0.083	-0.061	-0.059	0.043	0.207	-0.041	0.073	-0.016	0.001	-0.033	0.312	0.051	-0.029	0.036
CD100um gldm LDLGLE	-0.071	-0.037	0.017	0.785	-0.053	-0.049	-0.033	-0.005	0.045	0.005	0.021	0.088	-0.02	0.053	-0.01	-0.003	0.017	0.11	-0.042	0.02	0.023	0.002	-0.034	0.144	0.029	-0.036	0.048
CD100um gldm SDHGLE	0.235	-0.148	0.661	0.143	-0.088	0.045	0.092	0.068	-0.013	-0.069	-0.015	0.186	0.005	-0.034	-0.006	-0.017	-0.087	0.086	-0.068	-0.057	0.035	-0.085	0.089	-0.027	-0.111	0.069	-0.118
CD100um gldm SDLGLE	-0.677	0.098	-0.183	0.112	-0.008	0.045	-0.08	-0.04	0.234	0.029	-0.012	0.045	0.149	-0.008	-0.003	0.096	-0.062	0.106	-0.051	-0.065	0.061	0.022	-0.081	0.023	0.075	-0.067	0.153
CD100um ngtdm Busyness	0.156	0.503	-0.13	-0.043	0.013	0.016	0.482	0.082	-0.044	-0.105	0.124	0.08	-0.068	-0.004	-0.022	0.031	0.013	-0.063	0.034	0.124	-0.041	0.144	-0.148	0.028	-0.031	-0.098	0.087
CD100um ngtdm Complexity	0.274	-0.23	0.582	0.248	-0.042	0.037	0.19	0.086	0.002	0.172	0.004	0.175	0.054	0.062	-0.024	-0.001	0.029	0.085	0.033	0.075	0.065	0.03	-0.009	-0.06	-0.091	0.026	-0.068
CD100um ngtdm Contrast	-0.47	-0.533	0.113	0.123	-0.009	0.082	-0.087	-0.016	0.106	0.179	-0.015	0.041	0.046	0.032	0.018	-0.08	0.161	0.03	-0.022	0.112	0	-0.002	0.139	-0.054	-0.074	-0.087	0.074
CD100um ngtdm Strength	-0.263	-0.485	0.046	0.084	0.221	-0.123	-0.095	-0.03	-0.066	0.385	-0.148	-0.01	0.055	0.082	-0.021	0.032	0.031	0.087	0.146	0.149	0.015	0.128	0.101	-0.071	-0.097	0.08	0.02

Pathomic feature name	P F1	P F2	P F3	P F4	P F5	P F6	P F7	P F8	P F9	P F10	P F11	P F12	P F13	P F14	P F15	P F16	P F17	P F18	P F19	P F20	P F21	P F22	P F23	P F24	P F25	P F26	P F27
CD150um firstorder InterquartileRange	0.093	-0.366	0.484	0.043	-0.07	-0.069	-0.062	0.057	-0.062	0.308	-0.111	0.133	0.033	0.029	0.02	-0.301	0.048	-0.079	-0.135	0.019	-0.074	-0.007	0.007	0.017	-0.023	-0.029	0.033
CD150um firstorder Kurtosis	0.187	0.13	-0.166	0.006	0.097	0.025	0.016	-0.14	-0.074	-0.025	0.07	0.006	0.03	0.081	0.014	0.698	0.027	-0.036	0.055	0.008	0.008	0.025	0.043	-0.003	-0.024	-0.03	-0.014
CD150um firstorder Minimum	0.051	-0.078	0.146	-0.285	-0.128	0.046	-0.126	-0.07	0.059	-0.085	0.001	-0.656	-0.01	-0.088	0.038	-0.136	-0.006	0.021	0.043	0.125	-0.013	0.084	-0.044	-0.027	-0.084	0.025	0.005
CD150um firstorder Range	0.438	-0.156	0.555	0.088	-0.142	-0.038	0.161	0.061	-0.073	0.175	-0.007	0.133	-0.017	0.124	0.006	0.135	0.016	0.006	-0.037	0.001	0.029	0.002	0.028	-0.017	-0.016	0.022	0.016
CD150um firstorder Skewness	-0.039	0.041	-0.41	-0.163	0.28	-0.094	-0.003	-0.082	-0.056	0.498	0.037	0.187	0.039	0.052	-0.102	0.2	0.057	-0.091	0.05	0.021	0.011	-0.048	-0.093	-0.017	-0.005	0.012	0.034
CD150um firstorder Uniformity	-0.584	0.233	-0.352	0.096	0.243	0.009	-0.059	-0.087	0.156	-0.108	0.06	0.036	0.107	-0.034	0.004	0.136	0.016	0.021	-0.015	-0.015	0.07	-0.016	0.009	-0.048	0.013	-0.024	0.043
CD150um glcm JointAverage	0.311	-0.132	0.662	0.102	-0.156	0.004	0.125	0.072	-0.012	-0.134	-0.041	0.049	-0.023	0.082	0.024	0.024	-0.058	0.047	-0.075	-0.092	-0.011	-0.05	0.094	-0.037	0.025	0.037	-0.036
CD150um glcm ClusterShade	-0.014	0.039	-0.139	-0.711	0.13	-0.136	0.005	-0.019	0.04	0.245	0.01	-0.024	0.006	0.02	-0.043	-0.02	0.026	0.062	0.103	0.06	0.069	0.164	0.015	0.046	0.026	0.03	-0.043
CD150um glcm Correlation	0.516	0.196	0.139	0.127	0.077	-0.153	0.103	-0.1	-0.104	0.321	-0.257	0.01	-0.007	0.029	-0.048	-0.187	-0.054	0.054	-0.099	0.057	-0.131	0.067	0.002	0.024	-0.02	0.031	0.149
CD150um glcm DifferenceEntropy	0.513	-0.418	0.41	0.1	-0.102	0.044	-0.037	0.092	0.018	0.001	0.058	0.107	-0.01	0.036	-0.004	-0.053	0.044	0.005	0.053	0.004	0.059	-0.054	-0.038	-0.016	-0.019	0.01	-0.102
CD150um glcm DifferenceVariance	0.019	-0.501	0.447	0.368	0.1	-0.02	-0.087	0.081	0.082	0.064	0.004	0.069	0.021	0.089	-0.024	0.044	0.063	-0.075	0.084	0.041	0.02	-0.012	0.056	0.021	-0.099	-0.054	-0.083
CD150um glcm JointEnergy	-0.794	-0.046	-0.103	0.072	-0.087	0.032	-0.056	0.005	0.071	0.034	-0.004	0.037	0.052	-0.041	0.035	0.011	0.053	0.061	0.003	-0.024	0.063	0.002	0.012	0.009	0.022	-0.015	0.022
CD150um glcm Idm	0.228	0.577	-0.138	0.225	0.238	-0.094	-0.006	0.061	0.145	0.034	-0.162	-0.085	0.023	-0.069	-0.088	0.062	-0.097	-0.013	0.08	0.03	-0.053	0.102	0.018	0.148	0.047	-0.043	-0.067
CD150um glcm Idmn	0.732	0.242	0.104	-0.017	0.029	-0.05	0.088	-0.048	-0.013	0.081	-0.054	0.006	-0.048	0.065	-0.012	0.115	-0.05	-0.001	-0.043	0.055	-0.068	0.029	0.033	-0.007	-0.007	0.052	0.137
CD150um glcm InverseVariance	0.07	0.617	-0.235	-0.019	0.258	0.026	0.001	0.058	0.101	-0.054	-0.13	-0.097	0.138	-0.082	-0.18	0.033	-0.108	0.011	0.027	0.012	-0.044	0.05	-0.042	0.077	0.073	-0.035	-0.075
CD150um glcm MaximumProbability	-0.39	-0.135	-0.103	0.644	0.007	-0.06	-0.083	0.017	0.138	0.046	0.016	0.089	-0.004	-0.018	0.024	-0.005	0.083	0.107	-0.018	0.056	0.026	-0.027	-0.007	0.131	0.024	-0.038	0.038
CD150um glszm LALGLE	-0.092	-0.036	-0.054	0.68	-0.006	-0.118	-0.029	-0.058	0.015	0.008	0.026	0.119	-0.111	0.083	-0.028	-0.079	0.058	0.193	-0.068	0.078	0.024	0.019	-0.033	0.277	0.055	-0.008	0.032
CD150um gldm DependenceVariance	0.337	0.277	0.05	0.566	0.005	-0.139	-0.001	0.055	0.09	0.086	-0.077	0.009	-0.141	-0.007	0.006	0.048	0.001	0.038	0.098	0.038	-0.042	0.068	0.072	0.222	0.021	-0.051	-0.038
CD150um ngtdm Busyness	-0.156	0.541	-0.21	0.038	0.304	0.02	0.27	-0.001	0.036	-0.058	0.164	0.164	0.028	-0.057	-0.022	-0.003	0.02	-0.11	-0.017	0.106	0.011	0.077	-0.163	-0.019	-0.048	0.004	0.025
CD150um ngtdm Strength	-0.177	-0.54	0.137	0.117	0.145	-0.126	-0.155	-0.038	0.044	0.365	-0.179	-0.032	0.052	0.115	0.061	0.004	0.006	0.045	0.069	0.053	-0.032	0.069	0.131	-0.041	-0.073	0.074	0.12
CD200um firstorder Kurtosis	0.261	0.156	-0.099	0.034	0.154	0.076	0.073	-0.108	-0.032	-0.007	0.089	0.028	-0.016	0.036	0.064	0.513	0.006	0.062	0.457	-0.041	-0.018	0.006	-0.018	-0.009	0.01	-0.016	0.047
CD200um firstorder Minimum	0.06	-0.128	0.232	-0.307	-0.098	-0.022	-0.133	-0.011	-0.008	0.007	-0.068	-0.587	0.035	0.004	0.015	0.01	-0.044	0.112	-0.071	0.117	0.044	-0.147	-0.002	-0.013	-0.179	0.045	-0.01
CD200um firstorder Range	0.446	-0.105	0.553	0.145	-0.181	-0.013	0.188	0.051	-0.015	0.143	-0.019	0.074	-0.036	0.097	0.029	-0.003	-0.011	0.006	0.065	-0.042	0.009	0.093	-0.009	-0.026	0.065	0.002	0.018
CD200um firstorder RMAD	0.134	-0.36	0.497	0.148	-0.124	-0.02	-0.079	0.036	0	0.246	-0.094	0.036	0.005	0.047	0.01	-0.273	0.05	-0.112	-0.114	0.057	0.001	-0.023	0.032	-0.07	0.185	0.02	-0.008
CD200um firstorder Skewness	-0.143	0.024	-0.324	-0.155	0.244	-0.067	-0.036	-0.041	0.018	0.598	-0.011	0.194	0.076	0.054	-0.074	0.109	0.056	0.012	0.063	-0.008	0.041	-0.109	-0.068	0.013	-0.038	-0.004	0.045
CD200um firstorder TotalEnergy	0.179	0.209	0.318	-0.053	-0.106	0.004	0.675	0.079	-0.036	-0.055	-0.004	-0.045	0.019	-0.029	0.028	-0.018	-0.034	0.085	0.005	-0.037	-0.01	0.01	-0.033	-0.022	0.019	-0.035	-0.001
CD200um firstorder Uniformity	-0.73	0.126	-0.231	0.041	0.154	0.011	-0.057	-0.06	0.059	-0.039	0.014	0.045	0.056	-0.029	0.096	0.087	0.083	0.001	0.001	0.006	0.078	-0.016	0.054	-0.057	-0.005	0.024	0.043
CD200um glcm JointAverage	0.334	-0.073	0.615	0.134	-0.177	0.042	0.146	0.035	0.008	-0.207	-0.022	0.03	-0.062	0.026	0.041	-0.079	-0.049	-0.017	0.003	-0.107	-0.084	0.108	0.049	-0.03	0.067	0.023	-0.03
CD200um glcm ClusterProminence	0.089	-0.155	0.246	0.652	-0.02	-0.12	-0.027	0.06	0.015	0.147	-0.032	0.032	0.103	0.093	0.031	0.035	-0.025	-0.003	0.173	0.03	0.059	0.185	0.017	-0.113	0.111	0.001	0.011
CD200um glcm ClusterShade	-0.04	0.005	-0.124	-0.648	0.151	-0.15	-0.001	0.022	0.045	0.316	-0.029	-0.015	0.037	0.02	-0.049	-0.009	0.019	0.076	0.071	0.023	0.033	0.171	-0.007	0.103	-0.158	0.008	-0.076
CD200um glcm Correlation	0.277	0.463	0.116	0.131	-0.079	-0.286	0.066	-0.054	-0.069	0.299	-0.177	0.079	0.05	-0.053	0.061	-0.223	-0.032	0.046	0.048	-0.045	0.036	0.176	0.057	-0.031	0.06	0.015	0.087

Pathomic feature name	P F1	P F2	P F3	P F4	P F5	P F6	P F7	P F8	P F9	P F10	P F11	P F12	P F13	P F14	P F15	P F16	P F17	P F18	P F19	P F20	P F21	P F22	P F23	P F24	P F25	P F26	P F27
CD200um glcm DifferenceAverage	-0.046	-0.665	0.345	0.138	0.043	0.142	-0.038	0.007	0.064	0.013	-0.018	0.037	-0.083	0.068	-0.03	-0.072	0.068	-0.105	-0.08	0.079	0.002	-0.113	-0.005	0.011	-0.062	0.039	0.022
CD200um glcm DifferenceEntropy	0.694	-0.173	0.32	0.106	-0.114	0.083	-0.02	0.089	0.034	-0.01	0.016	0.056	-0.015	0.003	-0.024	-0.004	-0.042	-0.017	-0.007	-0.017	0.08	0.003	-0.091	0.044	0.046	0.011	-0.024
CD200um glcm DifferenceVariance	0.047	-0.499	0.409	0.335	0.081	0.225	-0.053	-0.009	0.078	0.054	0.077	-0.027	0.033	0.076	-0.071	-0.026	0.074	-0.06	-0.076	0.077	0.082	-0.081	0.034	-0.025	-0.002	-0.069	-0.007
CD200um glcm Imc2	-0.124	-0.321	-0.064	0.048	0.051	-0.13	-0.593	-0.058	0.053	0.171	-0.062	-0.08	0.041	-0.023	-0.026	-0.056	0.017	0.022	-0.192	-0.134	0.074	-0.125	0.081	0.04	0.057	-0.031	-0.043
CD200um glcm Idm	0.059	0.644	-0.13	0.156	0.286	-0.089	0.006	-0.004	-0.009	-0.053	0.221	-0.031	0.182	-0.011	0.004	0.012	-0.054	0.049	-0.015	-0.032	0.124	0.09	0.019	0.017	0.081	-0.045	0.024
CD200um glcm Idmn	0.515	0.542	0.073	-0.004	-0.131	-0.139	0.053	0.008	-0.002	0.052	-0.016	0.06	0.063	0.029	0.034	0.102	-0.05	0.062	0.119	-0.079	0.036	0.134	-0.016	0.002	0.043	-0.021	0.06
CD200um glcm InverseVariance	-0.099	0.621	-0.119	-0.026	0.287	0.049	-0.008	-0.093	0.049	0.028	0.116	-0.013	0.224	-0.019	0.042	0.032	0.005	0.026	0.015	-0.055	0.259	0.04	0.011	0.015	-0.036	-0.051	-0.019
CD200um glcm MaximumProbability	-0.616	-0.105	-0.111	0.334	0.034	-0.083	-0.088	0.015	-0.06	-0.079	0.203	0.104	0.078	0.026	-0.023	-0.01	0.043	0.064	0.004	0.066	0.027	-0.038	0.033	0.013	0.249	-0.007	0.043
CD200um gldm LDLGLE	-0.171	0.015	0.034	0.589	0.053	-0.13	-0.055	-0.068	0.018	-0.036	0.079	0.088	-0.024	0.126	0.011	-0.036	0.078	0.073	0.07	0.149	0.171	0.071	0.054	0.061	0.31	0.074	0.089
CD200um gldm SDE	-0.295	-0.497	0.033	-0.315	-0.13	0.185	-0.049	-0.099	0.096	0.115	-0.219	0.069	-0.017	-0.007	0.033	0.03	0.084	-0.032	0.031	-0.008	0.053	-0.11	-0.05	-0.026	-0.124	-0.003	-0.083
CD200um ngtdm Busyness	-0.411	0.406	-0.217	0.021	0.318	0.099	0.207	-0.013	0.049	0.088	0.081	0.179	-0.03	-0.036	0.06	0.074	0.081	-0.077	-0.04	0.102	0.09	-0.031	-0.098	0.009	-0.045	0.023	0.079
CD200um ngtdm Coarseness	-0.626	-0.004	-0.297	-0.013	0.221	-0.134	-0.149	-0.059	0.124	-0.001	-0.06	0.011	0.203	-0.03	-0.054	-0.003	-0.06	0.111	-0.061	-0.09	-0.001	-0.033	-0.01	-0.065	0.059	-0.006	-0.017
CD200um ngtdm Contrast	-0.522	-0.437	0.049	0.006	0.057	0.081	0.025	-0.106	-0.134	0.15	-0.084	-0.002	-0.129	0.012	0.032	-0.088	0.153	0.006	-0.026	0.179	-0.063	-0.027	0.168	-0.108	-0.078	0.013	0.086
CD200um ngtdm Strength	-0.123	-0.395	0.147	0.134	0.092	-0.242	-0.209	-0.003	0.11	0.396	-0.135	-0.089	0.144	0.131	0	-0.019	-0.036	0.06	0.183	0.025	0.074	0.218	0.106	-0.064	0.028	0.043	0.018
CD50um wav LH glcm Correlation	0.077	0.237	0.012	0.039	0.09	-0.109	-0.041	0.088	0.652	-0.054	-0.068	-0.062	0.162	0.084	0.032	-0.133	0.024	-0.069	0.12	0.006	0.124	0.117	-0.072	-0.059	0.014	-0.008	-0.148
CD50um wav LH glcm Imc2	0.019	-0.154	-0.078	0.032	0.054	0.041	-0.742	-0.094	0.019	-0.068	0.011	-0.023	-0.008	-0.027	0.006	-0.022	0.011	0.007	0.046	0.059	-0.037	0.076	-0.078	-0.018	-0.019	-0.017	0.033
CD50um wav LH glcm InverseVariance	-0.101	0.745	0.015	0.017	0.084	-0.039	0.061	-0.056	-0.078	-0.036	0.037	0.053	-0.121	0.065	0.133	-0.052	0.069	-0.103	0.028	0.00E+00	0.079	-0.088	0.044	0.003	-0.086	0.041	-0.045
CD50um wav LH glrlm RunVariance	0.357	0.553	0.066	0.058	0.165	0.131	0.087	-0.104	-0.061	-0.048	0.078	0.111	-0.109	0.076	0.026	0.066	0.066	-0.088	-0.118	0.011	0.001	-0.03	0.022	0.128	-0.13	0.041	-0.034
CD50um wav LH ngtdm Busyness	-0.765	-0.145	-0.146	-0.036	0.01	-0.036	0.062	-0.05	-0.134	0.014	-0.034	0.036	-0.064	-0.048	0.051	-0.026	0.017	0.003	-0.023	-0.008	-0.102	-0.024	0.021	-0.064	-0.004	0.033	0.007
CD50um wav LH ngtdm Coarseness	-0.493	0.12	-0.334	0.035	0.217	-0.065	-0.252	-0.065	0.291	-0.036	-0.042	0.029	0.181	-0.015	0.086	0.062	0.096	0.036	-0.044	-0.053	0.077	-0.102	0.024	-0.108	0.031	0.028	-0.102
CD50um wav LH ngtdm Strength	-0.125	-0.608	0.253	0.049	0.058	-0.042	-0.234	-0.007	0.066	0.04	-0.122	-0.274	0.041	0.053	-0.011	0.003	-0.028	-0.032	0.036	-0.06	0.161	0.055	0.035	0.069	-0.019	0.091	0.089
CD50um wav HL glcm ClusterShade	0.073	-0.269	0.311	-0.057	0.127	-0.075	-0.029	-0.045	0.429	-0.203	0.028	-0.113	0.024	0.167	-0.069	0.039	-0.024	-0.189	0.017	-0.169	-0.08	0.136	-0.111	0.253	-0.078	-0.023	-0.008
CD50um wav HL glcm Imc2	0.031	-0.165	-0.075	0.028	0.036	0.053	-0.727	-0.091	0.027	-0.071	0.013	-0.016	-0.003	-0.022	0.013	-0.017	0.019	-0.002	0.052	0.063	-0.054	0.086	-0.096	-0.029	-0.012	-0.019	0.023
CD50um wav HL glcm InverseVariance	0.251	0.596	-0.116	0.04	0.277	-0.131	0.04	-0.061	0.109	0.013	-0.122	0.06	0.027	0.004	-0.217	0.036	-0.062	0.094	0.033	0.073	-0.12	-0.079	0.003	0.026	0.021	-0.082	-0.043
CD50um wav HL glrlm RunVariance	0.289	0.506	-0.137	0.029	0.099	-0.043	0.086	-0.013	-0.04	-0.065	-0.076	-0.003	-0.046	0.033	0.014	-0.014	0.111	0.005	-0.124	0.051	0.023	0.004	0.423	-0.035	-0.066	0.022	-0.029
CD50um wav HL ngtdm Busyness	-0.785	-0.039	-0.138	-0.021	0.021	-0.011	0.053	-0.055	-0.051	-0.019	-0.1	0.012	-0.017	-0.019	0.084	-0.045	0.041	-0.006	-0.056	-0.034	0.019	0.005	0.04	-0.031	-0.014	0.087	0.024
CD50um wav HL ngtdm Coarseness	-0.299	0.166	-0.343	0.067	0.444	-0.086	-0.273	-0.097	0.135	0.066	-0.084	0.03	0.186	-0.076	-0.013	0.08	-0.036	-0.018	-0.064	-0.136	0.149	-0.07	0.055	-0.047	0.044	0.093	0.039
CD50um wav HH firstorder Uniformity	-0.785	0.119	-0.158	-0.019	-0.009	-0.027	-0.035	-0.035	0.1	0.029	-0.021	0.025	0.049	-0.04	0.059	0.007	0.045	0.014	-0.019	-0.024	0.039	-0.017	0.015	-0.038	-0.003	-0.002	0.018
CD50um wav HH glcm Imc2	-0.012	-0.212	-0.093	0.035	0.05	0.026	-0.749	-0.112	-0.005	-0.048	-0.001	-0.03	-0.025	-0.024	0.003	-0.009	0.009	-0.002	0.037	0.048	-0.043	0.055	-0.066	-0.019	-0.01	0.002	0.01

Pathomic feature name	P F1	P F2	P F3	P F4	P F5	P F6	P F7	P F8	P F9	P F10	P F11	P F12	P F13	P F14	P F15	P F16	P F17	P F18	P F19	P F20	P F21	P F22	P F23	P F24	P F25	P F26	P F27
CD50um wav HH glcm Idm	-0.127	0.754	-0.082	0.011	0.044	0.054	0.011	-0.048	0.211	0.044	-0.036	0.055	0.046	0.003	-0.094	0.034	0.049	-0.021	-0.021	0.028	0.069	0.026	-0.007	-0.013	-0.004	0.006	0.051
CD50um wav HH glcm Idn	0.623	0.341	0.269	-0.041	-0.06	0.056	0.123	0.116	-0.041	0.059	0.012	-0.042	-0.022	0.081	-0.07	-0.001	0.019	-0.001	0.177	-0.013	-0.009	0.036	0.034	0.068	0.002	-0.013	-0.074
CD50um wav HH glcm InverseVariance	0.118	0.667	-0.09	0.026	0.245	0.058	0.04	-0.107	0.102	0.061	-0.019	0.064	0.044	-0.019	-0.277	0.095	0.024	0.046	-0.061	0.063	-0.049	0.034	0.014	-0.015	-0.031	0.012	0.018
CD50um wav HH glrlm RunVariance	0.074	0.686	-0.034	-0.016	-0.24	0.02	0.058	0.038	0.147	-0.005	-0.07	-0.011	-0.051	0.115	0.037	-0.103	0.012	-0.127	0.061	0.041	0.064	-0.029	-0.032	-0.02	0.06	0.039	0.059
CD50um wav HH ngtdm Busyness	-0.736	0.27	-0.069	0.01	0.01	0.012	0.022	0.004	0.12	-0.005	0.011	0.088	0.07	-0.056	0.067	0.015	0.084	0.007	-0.067	-0.006	0.131	0.019	-0.007	-0.003	0.002	-0.005	0.034
CD50um wav LL glcm Imc2	0.727	0.014	-0.004	0.017	0.113	0.063	-0.048	0.006	0.287	-0.027	0.104	-0.007	0.158	0.041	-0.054	0.001	-0.005	0.037	-0.048	0.047	0.021	0.00E+00	0.038	0.003	-0.011	-0.054	0.057
CD50um wav LL glcm Idmn	0.676	0.315	0.152	0.048	-0.005	-0.095	0.101	0.022	-0.151	0.102	0.067	0.104	-0.051	0.087	0.001	0.027	-0.034	-0.017	0.091	0.016	-0.083	-0.004	-0.014	-0.007	-0.017	-0.005	0.055
CD50um wav LL glcm InverseVariance	0.152	0.68	-0.067	-0.015	0.127	0.009	0.101	-0.04	-0.111	-0.036	0.194	0.013	-0.061	-0.01	0.156	0.08	-0.06	-0.098	0.059	0.134	-0.052	-0.046	0.121	-0.05	-0.075	0.028	0.084
CD50um wav LL glrlm ShortRunEmphasis	-0.376	-0.424	-0.054	0.009	0.112	0.036	-0.132	-0.081	0	0.017	-0.075	0.05	0.202	-0.106	0.168	-0.025	0.116	0.147	-0.236	-0.271	-0.019	0.025	0.05	-0.13	-0.041	0.097	0.004
CD50um wav LL ngtdm Busyness	-0.806	-0.064	-0.093	-0.035	-0.092	0.006	-0.025	-0.006	0.039	0.038	-0.015	0.018	-0.002	-0.044	0.048	0.00E+00	0.034	0.002	0.022	-0.041	0.038	-0.008	-0.012	-0.005	0.001	-0.003	-0.009
CD50um wav LL ngtdm Coarseness	-0.353	0.268	-0.324	0.067	0.44	-0.054	-0.25	-0.117	-0.066	0.017	0.004	0.101	0.116	0.03	0.036	0.083	0.015	0.054	-0.11	-0.097	0.02	-0.157	0.053	-0.042	0.008	0.051	-0.029
CD50um lbp 2D firstorder Kurtosis	0.044	-0.129	0.339	0.297	-0.353	0.023	0.118	0.043	-0.429	-0.053	0.027	-0.034	-0.223	0.115	-0.052	-0.134	-0.006	-0.114	-0.003	0.06	-0.027	0.053	0.024	-0.048	-0.107	0.064	-0.026
CD50um lbp 2D firstorder RMAD	0.148	0.506	-0.273	-0.096	0.374	-0.068	-0.031	0.016	0.3	0.021	-0.074	-0.088	0.147	-0.067	-0.019	0.108	-0.022	0.08	0.083	-0.063	-0.068	-0.04	0.033	0.02	0.055	-0.047	0.054
CD50um lbp 2D firstorder Skewness	-0.51	-0.348	-0.077	-0.166	0.441	-0.096	-0.07	-0.098	-0.05	0.143	-0.015	0.125	0.033	-0.04	0.05	0.017	0.05	0.024	0.01	0.034	0.002	-0.035	-0.008	-0.011	-0.01	-0.008	0.014
CD50um lbp 2D glcm Autocorrelation	0.513	0.486	0.036	0.311	-0.136	0.055	0.046	0.052	0.169	-0.077	0.026	-0.08	0.008	0.039	-0.085	-0.019	-0.037	-0.021	-0.005	-0.029	0.02	0.034	0.013	-0.023	-0.003	0.018	-0.052
CD50um lbp 2D glcm ClusterProminence	0.081	-0.042	-0.132	-0.064	0.671	-0.026	-0.068	0.128	0.044	0.174	0.149	-0.063	0.083	-0.008	-0.148	0.068	0.049	0.027	0.17	0.004	-0.067	0.059	0.07	0.104	-0.008	0.055	-0.14
CD50um lbp 2D glcm ClusterShade	-0.442	-0.512	0.068	-0.383	0.037	-0.027	-0.039	-0.014	-0.171	0.066	-0.03	0.019	0.004	-0.007	-0.032	0.02	0.089	-0.067	0.029	0.071	0.033	0.001	0.006	0.037	-0.04	-0.074	0.024
CD50um lbp 2D glcm ClusterTendency	0.294	0.072	-0.12	0.096	0.524	-0.005	-0.065	0.137	0.178	0.087	0.222	-0.091	0.139	0.004	-0.192	0.055	0.086	-0.01	0.186	-0.001	-0.035	0.051	0.069	0.072	-0.006	-0.037	-0.185
CD50um lbp 2D glcm DifferenceAverage	-0.158	0.364	-0.215	-0.53	-0.06	-0.032	0.001	0	0.116	-0.114	-0.18	-0.155	0.03	-0.052	0.066	0.117	-0.058	0.045	0.077	-0.088	-0.083	-0.088	0.005	0.072	0.084	-0.116	0.157
CD50um lbp 2D glcm JointEntropy	0.086	-0.576	0.085	-0.316	-0.104	-0.037	-0.038	-0.113	-0.002	0.078	0.187	0.281	0.009	-0.004	0.078	-0.033	0.001	0.072	-0.154	0.036	0.149	0.047	-0.113	0.002	0.036	0.042	-0.046
CD50um lbp 2D glcm Imc2	-0.701	-0.238	-0.041	0.154	0.031	0.054	-0.059	0.01	0.029	-0.004	-0.174	-0.085	0.073	-0.006	0.141	0.028	0.059	0.001	0.052	-0.004	-0.017	-0.057	0.091	-0.03	-0.029	-0.024	0.099
CD50um lbp 2D glcm Idm	0.251	-0.009	0.092	0.695	0.214	0.043	0.007	0.022	-0.09	0.088	-0.034	-0.054	-0.04	-0.003	-0.102	-0.059	-0.017	-0.049	-0.002	0.028	-0.038	0.013	0.045	-0.09	-0.082	0.079	-0.125
CD50um lbp 2D glcm Id	0.215	-0.061	0.111	0.715	0.196	0.031	0.003	0.01	-0.078	0.096	-0.003	-0.02	-0.034	-0.005	-0.1	-0.059	-0.015	-0.056	-0.01	0.022	-0.019	0.015	0.028	-0.097	-0.085	0.069	-0.119
CD50um lbp 2D glcm MaximumProbability	0.028	0.005	0.103	0.774	0.017	0.032	0.004	0.047	-0.016	0.029	-0.09	-0.101	-0.033	0.054	-0.023	0.02	-0.002	-0.102	0.108	-0.06	-0.04	-0.032	0.064	-0.071	-0.077	0.024	0.01
CD50um lbp 2D glcm SumSquares	0.073	0.431	-0.268	-0.282	0.309	-0.024	-0.026	0.091	0.169	-0.045	-0.105	-0.253	0.079	-0.056	-0.074	0.12	-0.004	0.026	0.192	-0.072	-0.144	-0.071	0.073	0.079	0.058	-0.117	0.013
CD50um lbp 2D glrlm LRLGLE	-0.198	0.144	-0.099	0.16	0.704	-0.168	-0.057	-0.051	0.054	0.116	-0.105	0.031	0.07	-0.051	0.018	0.057	-0.009	0.038	0.074	-0.02	-0.052	-0.04	-0.005	-0.042	-0.022	-0.016	0.044
CD50um lbp 2D glrlm RunEntropy	0.331	-0.506	0.144	0.283	-0.335	0.116	-0.027	-0.046	-0.04	0.045	0.082	0.075	-0.058	-0.031	-0.066	-0.06	-0.077	-0.021	-0.108	-0.027	0.095	0.033	-0.077	-0.034	-0.014	0.025	-0.125
CD50um lbp 2D glrlm RunVariance	0.063	-0.09	0.138	0.793	-0.062	0.025	-0.009	0.051	-0.01	-0.003	-0.024	0.005	0.048	0.009	0.017	0.041	-0.026	-0.04	0.023	-0.031	-0.009	-0.005	-0.01	-0.074	-0.043	-0.03	0.007
CD50um lbp 2D glrlm SRLGLE	-0.194	0.169	-0.119	0.014	0.706	-0.156	-0.057	-0.057	0.082	0.123	-0.114	0.031	0.063	-0.029	0.035	0.066	-0.003	0.041	0.095	-0.049	-0.04	-0.047	0.007	0.001	-0.018	-0.031	0.091
CD50um lbp 2D glszm GLNUN	0.035	0.662	-0.096	0.03	0.409	-0.085	0.053	0.016	-0.004	-0.014	-0.106	-0.128	0.012	-0.008	-0.059	-0.003	-0.012	-0.013	0.063	-0.002	-0.088	-0.018	0.079	-0.016	-0.019	0.029	0.044
CD50um lbp 2D gldm LDHGLE	0.393	0.162	0.12	0.642	-0.156	0.065	0.036	0.06	0.017	-0.011	0.001	-0.1	-0.021	0.018	-0.083	0.012	-0.063	-0.08	0.042	-0.076	-0.012	0.002	0.025	-0.047	-0.068	-0.021	0.003

Pathomic feature name	P F1	P F2	P F3	P F4	P F5	P F6	P F7	P F8	P F9	P F10	P F11	P F12	P F13	P F14	P F15	P F16	P F17	P F18	P F19	P F20	P F21	P F22	P F23	P F24	P F25	P F26	P F27
CD50um lbp 2D gldm LDLGLE	-0.254	0.138	-0.132	-0.043	0.7	-0.188	-0.055	-0.061	0.034	0.104	-0.083	0.04	0.066	-0.066	0.018	0.037	0.007	0.058	0.047	0.02	-0.055	-0.032	-0.009	-0.046	-0.004	0.001	0.009
CD50um lbp 2D gldm SDE	-0.334	-0.046	-0.108	-0.683	0.011	-0.062	-0.024	-0.002	0.055	-0.057	0.036	0.095	0.06	0.036	0.146	0.043	0.095	0.078	0.012	0.034	0.013	-0.017	-0.011	0.088	0.078	-0.041	0.09
CD50um lbp 2D gldm SDHGLE	0.383	0.393	0.002	-0.174	-0.505	0.068	0.074	0.097	0.062	-0.212	0.022	-0.117	-0.018	0.032	-0.004	-0.013	-0.009	0.017	-0.039	0.004	-0.02	0.03	0.009	0.028	0.066	0.022	-0.035
CD50um lbp 2D ngtdm Complexity	0.058	0.458	-0.205	-0.331	-0.231	0.05	0.057	0.087	0.016	-0.138	-0.178	-0.268	-0.012	-0.054	-0.043	0.083	-0.03	-0.005	0.134	-0.07	-0.189	-0.129	0.06	0.071	0.066	-0.119	0.097
CD50um lbp 2D ngtdm Contrast	0.045	0.445	-0.287	-0.37	-0.08	0.036	-0.008	0.077	0.198	-0.091	-0.136	-0.206	0.088	-0.085	-0.004	0.144	-0.031	0.045	0.127	-0.1	-0.131	-0.115	0.023	0.088	0.102	-0.127	0.089
CD100um lbp 2D firstorder Uniformity	-0.732	0.138	-0.054	-0.028	0.032	-0.027	0.064	0.039	-0.094	-0.029	0.078	-0.016	-0.055	-0.021	0.122	0.053	-0.029	-0.106	0.125	0.058	-0.086	-0.005	0.108	0.096	-0.07	0.011	0.042
CD100um lbp 2D glcm Autocorrelation	0.647	0.374	-0.001	0.166	-0.146	0.084	0.079	0.007	0.057	-0.045	-0.005	-0.037	-0.059	0.023	0.056	0.037	0.012	0.138	0.04	0.015	-0.098	-0.075	0.071	-0.035	0.009	0.066	0.024
CD100um lbp 2D glcm ClusterShade	-0.551	-0.185	-0.019	-0.219	-0.006	0.034	-0.125	0.014	0.317	-0.005	-0.087	0.078	0.193	-0.03	-0.166	0.095	0.044	-0.052	0.068	0.009	0.09	0.045	-0.052	0.078	0.042	-0.241	-0.038
CD100um lbp 2D glcm ClusterTendency	0.587	0.139	-0.14	0.109	0.087	0.272	-0.039	0.06	0.12	0.018	-0.139	-0.099	0.029	-0.087	0	0.166	-0.008	0.102	-0.01	0.147	-0.08	-0.017	0.185	-0.046	0.082	0.07	-0.013
CD100um lbp 2D glcm Contrast	-0.176	0.372	-0.138	-0.266	-0.136	0.156	0.112	0.04	-0.486	-0.14	0.126	-0.091	-0.047	-0.071	0.129	-0.009	-0.005	-0.097	0.107	-0.024	0.027	0.007	0.131	0.04	0.001	-0.11	0.017
CD100um lbp 2D glcm Correlation	0.493	-0.163	0.007	0.267	0.144	0.056	-0.096	0.001	0.402	0.107	-0.179	-0.005	0.052	-0.003	-0.089	0.107	-0.011	0.131	-0.07	0.118	-0.074	-0.014	0.025	-0.06	0.047	0.108	-0.022
CD100um lbp 2D glcm DifferenceEntropy	0.689	0.182	0.022	-0.098	-0.061	0.006	0.075	-0.156	0.02	-0.075	0.022	0.096	-0.053	-0.019	-0.092	-0.006	0.025	-0.043	0.03	0.044	-0.034	-0.066	0.056	-0.045	-0.033	-0.264	0.035
CD100um lbp 2D glcm DifferenceVariance	-0.229	0.319	-0.096	-0.06	0.077	-0.054	0.034	0.139	-0.551	-0.142	0.19	-0.055	-0.05	-0.146	0.084	-0.007	-0.095	-0.143	0.102	-0.118	-0.013	0.063	0.044	0.107	0.024	0.034	-0.041
CD100um lbp 2D glcm JointEnergy	-0.75	0.066	-0.058	0.084	-0.138	0.064	-0.013	0.107	-0.175	-0.016	0.092	-0.005	0.009	-0.068	0.081	0.036	0.001	0.004	0.084	-0.057	0.018	0.022	0.04	0.08	0.02	-0.01	-0.02
CD100um lbp 2D glcm JointEntropy	0.789	0.024	0.094	0.009	0.082	-0.071	0.009	-0.067	0.12	0.007	-0.05	0.017	-0.016	0.047	-0.066	-0.02	-0.016	-0.005	-0.076	0.012	0.008	-0.008	-0.057	-0.054	-0.006	0.014	0.003
CD100um lbp 2D glcm Imc1	0.784	0.087	0.127	-0.018	0.035	-0.072	0.055	-0.048	-0.009	-0.014	-0.066	-0.002	-0.094	0.073	-0.038	0.006	-0.041	-0.072	0.009	0.041	-0.035	-0.006	-0.023	0.008	-0.042	0.018	0.025
CD100um lbp 2D glcm Id	0.118	-0.131	0.11	0.522	0.252	-0.31	-0.117	0.082	0.138	0.008	0.036	0.115	0.031	-0.052	-0.177	0.003	-0.108	0.003	0.009	-0.056	-0.097	0.043	-0.14	0.087	0.011	0.077	-0.084
CD100um lbp 2D glcm InverseVariance	0.187	-0.102	0.076	-0.181	0.259	-0.076	-0.031	0.163	-0.194	0.035	-0.192	-0.153	0.037	0.055	0.134	-0.015	0.102	0.004	-0.112	0.043	0.001	0.062	0.004	-0.019	-0.013	0.496	-0.011
CD100um lbp 2D glcm MaximumProbability	-0.485	0.031	-0.059	0.496	-0.045	-0.037	-0.034	0.087	-0.297	-0.02	0.135	0.05	-0.034	-0.112	0.093	0.054	-0.052	0.036	0.063	-0.112	-0.018	-0.012	-0.007	0.13	0.059	-0.007	-0.044
CD100um lbp 2D glcm SumEntropy	0.772	-0.107	-0.019	0.005	0.152	-0.015	0.029	-0.074	0.037	0.006	-0.076	-0.048	-0.05	0.041	-0.037	-0.006	-0.083	-0.009	-0.062	0.05	-0.099	-0.024	0.003	-0.07	0.002	0.037	0.024
CD100um lbp 2D glcm SumSquares	0.228	0.413	-0.211	-0.16	-0.062	0.313	0.072	0.074	-0.343	-0.11	0.019	-0.143	-0.021	-0.119	0.112	0.101	-0.01	-0.019	0.086	0.076	-0.029	-0.006	0.234	0.005	0.055	-0.049	0.006
CD100um lbp 2D glrlm GrayLevelVariance	0.523	0.412	-0.144	-0.049	0.067	0.068	0.104	0.017	-0.339	-0.105	0.043	-0.092	-0.077	-0.053	0.091	0.006	-0.074	-0.03	0.025	0.037	-0.107	-0.024	0.132	-0.005	0.02	0.03	-0.029
CD100um lbp 2D glrlm LRLGLE	-0.345	0.091	-0.095	0.011	0.595	-0.183	-0.062	0.017	-0.196	-0.055	0.033	0.043	0.062	-0.102	-0.063	-0.039	-0.173	-0.188	-0.044	0.02	0.025	0.068	0.008	0.062	0.028	-0.028	-0.048
CD100um lbp 2D glrlm LGLRE	-0.4	0.149	-0.11	-0.061	0.562	-0.099	-0.059	0.013	-0.221	-0.021	-0.112	0.036	0.057	-0.117	-0.04	-0.007	-0.137	-0.176	-0.002	0.06	0.066	0.07	0.046	0.052	0.037	-0.032	-0.02
CD100um lbp 2D glszm GLNUN	-0.759	0.106	-0.055	-0.087	0.066	-0.043	0.047	0.058	-0.101	-0.019	0.072	-0.026	-0.012	-0.03	0.112	0.002	-0.018	-0.118	0.059	0.053	-0.042	0.021	0.082	0.06	-0.066	0.038	0.007
CD100um lbp 2D glszm ZoneVariance	0.122	0.019	-0.003	0.65	-0.066	-0.139	-0.028	-0.026	0.003	-0.019	0.104	0.144	-0.132	0.053	-0.063	-0.046	0.05	0.187	-0.035	0.052	-0.002	0.005	-0.019	0.302	0.07	-0.039	0.005
CD100um lbp 2D gldm DependenceEntropy	0.747	0.065	0.112	0.197	0.017	-0.106	-0.026	0.006	0.096	-0.033	-0.007	0.04	-0.013	-0.005	-0.148	-0.027	-0.042	0.014	-0.025	-0.042	-0.024	0.024	-0.072	0	0	-0.009	-0.062
CD100um lbp 2D gldm LDHGLE	0.389	0.377	0.065	0.472	-0.218	0.041	0.02	0.047	-0.008	-0.035	-0.008	0.081	-0.081	-0.034	-0.034	0.109	-0.027	0.118	0.154	-0.015	-0.048	-0.032	0.049	0.112	0.04	-0.075	0.05
CD100um lbp 2D gldm SDHGLE	0.601	0.325	-0.037	-0.081	-0.24	0.07	0.153	0.002	-0.142	-0.094	0.068	-0.129	-0.098	0.063	0.107	-0.015	-0.002	0.08	0.003	0.01	-0.119	-0.068	0.079	-0.057	-0.008	0.089	-0.009

Pathomic feature name	P F1	P F2	P F3	P F4	P F5	P F6	P F7	P F8	P F9	P F10	P F11	P F12	P F13	P F14	P F15	P F16	P F17	P F18	P F19	P F20	P F21	P F22	P F23	P F24	P F25	P F26	P F27
CD100um lbp 2D ngtdm Contrast	-0.312	0.363	-0.082	-0.151	-0.201	0.136	0.079	0.058	-0.525	-0.075	0.002	-0.066	-0.125	-0.072	0.072	0.044	-0.006	-0.053	0.135	-0.028	-0.002	-0.011	0.081	0.047	0.046	-0.041	-0.071
CD150um wav LH firstorder Uniformity	-0.753	0.198	-0.156	-0.017	0.102	-0.034	0.008	-0.069	0.027	0.017	-0.033	0.032	-0.027	-0.007	0.107	-0.023	0.054	-0.034	-0.044	0.029	-0.013	-0.077	0.039	-0.068	-0.033	0.031	0.02
CD150um wav LH glcm DifferenceEntropy	0.742	-0.001	0.274	0.071	-0.131	0.033	-0.03	0.063	0.038	-0.041	0.049	-0.004	-0.001	-0.037	-0.018	0.051	-0.016	0.015	0.015	-0.05	0.087	0.057	-0.043	0.046	0.035	-0.002	-0.004
CD150um wav LH glcm DifferenceVariance	-0.015	-0.428	0.483	0.135	0.018	-0.018	-0.111	-0.088	-0.263	-0.025	-0.091	-0.215	-0.097	0.026	-0.008	0.02	-0.071	-0.063	-0.04	-0.057	0.091	-0.03	-0.002	0.074	0.016	0.105	0.161
CD150um wav LH glcm Idmn	0.619	0.385	0.229	0.006	-0.105	0.037	0.07	0.027	0.167	-0.022	-0.002	0	-0.039	0.048	-0.047	-0.015	0.087	0.016	0.068	0.077	0.092	-0.009	-0.028	-0.018	-0.024	-0.075	-0.085
CD150um wav LH glcm InverseVariance	0.044	0.74	-0.028	0.013	0.092	-0.06	0.078	-0.072	0.042	-0.067	0.11	0.03	-0.036	0.083	0.144	-0.012	0.059	-0.087	-0.074	0.073	0.048	-0.056	0.083	-0.063	-0.071	-0.018	-0.006
CD150um wav LH glrlm LRHGLE	0.189	-0.21	0.571	0.114	-0.09	-0.073	0.043	0.026	-0.127	0.126	-0.074	-0.208	-0.071	0.133	-0.079	-0.084	-0.072	0.017	-0.004	-0.027	0.211	-0.09	-0.026	0.018	-0.001	0.07	0.04
CD150um wav LH glszm SZNUN	-0.073	-0.702	-0.095	-0.059	-0.047	-0.113	-0.08	0.053	0.202	0.047	-0.039	-0.069	0.13	-0.05	-0.05	-0.028	-0.081	0.061	-0.09	0.069	-0.02	0.104	-0.073	-0.103	0.125	-0.039	0.05
CD150um wav LH gldm DependenceVariance	0.137	0.678	0.064	0.063	0.146	0.057	0.077	-0.065	-0.111	0.009	0.041	0.105	-0.147	0.047	0.054	-0.029	0.042	-0.103	-0.091	0.005	0.071	-0.059	-0.039	0.134	-0.088	0.066	-0.06
CD150um wav LH ngtdm Busyness	-0.707	0.046	-0.183	-0.034	0.094	-0.007	0.241	-0.05	-0.101	-0.016	0.013	0.112	-0.081	-0.043	0.056	-0.017	0.029	-0.042	-0.046	0.014	-0.15	0.011	0.008	-0.088	-0.017	0.053	0.012
CD150um wav HL firstorder Uniformity	-0.742	0.183	-0.187	-0.01	0.167	-0.034	-0.003	-0.063	-0.001	0.03	-0.073	0.029	0.037	-0.032	0.039	-0.003	0.019	0.036	-0.042	-0.002	0.021	-0.031	0.085	-0.021	-0.025	0.051	0.074
CD150um wav HL glcm Imc2	-0.06	-0.269	-0.086	0.024	-0.018	0.035	-0.743	-0.101	0.009	-0.028	-0.009	-0.039	0	-0.033	0.007	0	0.024	-0.009	0.041	0.014	-0.039	0.052	-0.067	-0.027	0.012	-0.016	-0.011
CD150um wav HL glszm SmallAreaEmphasis	-0.342	-0.57	0.162	-0.054	-0.132	0.045	-0.085	-0.001	0.027	-0.051	0.075	-0.046	0.022	-0.017	0.155	-0.101	-0.086	-0.083	-0.023	-0.098	0.103	0.011	-0.26	0.013	0.052	0.054	0.016
CD150um wav HL ngtdm Contrast	-0.62	-0.313	0.047	-0.049	-0.022	-0.103	0.038	-0.084	-0.134	-0.005	-0.077	0.001	-0.195	0.016	0.037	-0.069	0.166	-0.071	-0.016	0.1	-0.157	-0.091	0.089	-0.083	-0.096	0.035	-0.055
CD150um wav HH glcm ClusterProminence	-0.031	-0.452	0.406	0.115	0.164	0.021	-0.055	0.113	0.167	-0.136	0.054	0.044	0.081	0.206	-0.108	0.021	0.116	-0.231	0.009	0.015	-0.016	-0.123	-0.016	0.196	-0.107	0.012	0.087
CD150um wav HH glcm Correlation	0.51	-0.053	-0.019	0.07	0.143	-0.073	0.023	-0.024	0.238	0.079	0.163	-0.262	-0.014	0.189	0.019	-0.145	-0.06	-0.098	0.192	-0.071	0.017	0.143	-0.18	-0.042	-0.131	-0.059	-0.079
CD150um wav HH glcm InverseVariance	0.154	0.704	-0.082	0.025	0.283	0.013	0.049	-0.124	0.038	0.063	0.035	0.033	-0.029	0	-0.105	0.035	0.09	-0.024	-0.066	0.046	-0.012	0.02	0.062	-0.054	-0.09	-0.025	-0.021
CD150um wav HH glszm SZNUN	-0.229	-0.715	0.058	0.004	0.112	-0.066	-0.055	-0.026	-0.104	-0.022	0.136	0.017	-0.043	-0.047	0.067	-0.033	-0.002	-0.009	-0.007	-0.04	0.053	0.006	-0.01	-0.017	-0.03	-0.14	-0.138
CD150um wav HH ngtdm Busyness	-0.517	0.485	-0.093	0.042	0.23	-0.03	0.168	-0.027	0.106	-0.037	0.011	0.124	0.046	-0.052	0.046	-0.005	0.091	-0.011	-0.157	0.07	0.091	0.024	-0.012	-0.033	-0.022	0.024	0.046
CD150um wav HH ngtdm Strength	-0.184	-0.678	0.165	0.017	0.17	0.046	-0.14	0.086	0.166	0.001	-0.025	-0.096	0.067	0.144	-0.016	-0.015	0.019	-0.062	0.029	0.026	0.014	-0.09	0.07	0.094	-0.056	0.085	0.053
CD150um wav LL glcm Imc2	0.577	-0.1	-0.032	0.034	0.105	0.036	-0.394	-0.034	0.301	0.01	0.078	-0.045	0.179	0.009	-0.054	-0.045	-0.008	0.052	-0.097	0.017	0.044	-0.011	0.054	0.03	0	-0.077	0.083
CD150um wav LL glcm InverseVariance	-0.108	0.649	-0.04	-0.02	0.026	0.032	0.113	0.035	-0.345	0.00E+00	0.099	0.018	-0.186	0.011	0.05	0.065	-0.036	-0.111	0.136	0.05	-0.05	-0.045	-0.018	0.051	-0.056	0.014	0.026
CD150um wav LL glszm SmallAreaEmphasis	-0.352	-0.553	-0.037	-0.009	0.035	0.057	-0.122	0.019	0.015	0.069	-0.138	0.026	0.153	-0.028	0.019	0.002	0.068	0.126	-0.159	-0.244	0.061	0.077	-0.161	0.037	0.029	0.039	-0.018
CD150um wav LL ngtdm Coarseness	-0.461	0.109	-0.337	0.043	0.381	-0.039	-0.259	-0.1	-0.027	0.044	-0.011	0.092	0.181	0.026	-0.01	0.077	0.013	0.084	-0.092	-0.1	0.028	-0.156	0.045	-0.075	0.033	0.03	-0.008
CD150um lbp 2D firstorder Uniformity	-0.742	-0.101	-0.107	-0.032	0.233	0.016	-0.005	-0.071	0.023	-0.015	0.017	0.017	0.046	-0.006	-0.069	-0.049	-0.134	-0.043	-0.02	0.019	0.087	-0.003	-0.035	0.06	-0.011	-0.072	0.029
CD150um lbp 2D glcm Autocorrelation	0.656	0.339	0.107	0.133	-0.177	0.071	0.086	0.035	-0.044	-0.047	-0.006	0.018	-0.062	0.078	0.063	0.024	0.071	0.14	0.032	0.064	0.007	-0.058	0.1	0.021	-0.008	0.018	0.024
CD150um lbp 2D glcm ClusterProminence	0.645	-0.033	0.022	-0.025	0.021	0.119	-0.015	0.005	-0.036	-0.072	0.086	0.031	0.048	-0.081	0.065	0.069	0.232	-0.008	0.006	0.183	0.227	-0.037	0.242	0.013	-0.042	0.005	0.063
CD150um lbp 2D glcm ClusterShade	-0.081	-0.438	-0.087	-0.241	0.087	0.033	-0.13	0.034	0.138	-0.062	0.049	-0.047	0.183	-0.186	-0.131	-0.019	0.314	-0.213	-0.008	0.065	0.052	-0.015	0.13	-0.058	-0.102	-0.057	-0.044
CD150um lbp 2D glcm ClusterTendency	0.645	0.048	0.028	0.055	-0.018	0.169	-0.003	0.021	-0.118	-0.073	0.008	0.008	-0.003	-0.037	0.149	0.056	0.24	0.051	0.001	0.157	0.209	-0.048	0.22	0.022	-0.007	0.033	0.014
CD150um lbp 2D glcm Contrast	0.116	0.395	-0.186	-0.056	0.014	0.118	0.112	0.1	-0.117	-0.135	0.294	-0.08	-0.074	0.037	0.368	-0.028	-0.391	-0.009	0.035	-0.05	-0.028	-0.024	0.056	-0.07	-0.009	-0.006	0.012
CD150um lbp 2D glcm Correlation	0.539	-0.206	0.145	0.072	0.008	0.053	-0.068	-0.029	-0.016	0.019	-0.134	0.06	0.054	-0.046	-0.082	0.059	0.432	0.02	-0.022	0.155	0.174	-0.022	0.161	0.059	-0.024	0.04	0.026

Pathomic feature name	P F1	P F2	P F3	P F4	P F5	P F6	P F7	P F8	P F9	P F10	P F11	P F12	P F13	P F14	P F15	P F16	P F17	P F18	P F19	P F20	P F21	P F22	P F23	P F24	P F25	P F26	P F27
CD150um lbp 2D glcm DifferenceEntropy	0.714	0.21	-0.032	0.045	0.047	0.046	0.08	0.007	-0.018	-0.074	0.124	-0.073	-0.098	0.014	0.2	-0.046	-0.064	0.018	-0.06	0.01	-0.073	-0.017	0.076	-0.107	-0.02	0.047	-0.027
CD150um lbp 2D glcm DifferenceVariance	0.058	0.375	-0.198	0.073	0.138	-0.024	0.101	-0.023	-0.049	-0.071	0.084	-0.109	-0.122	-0.11	-0.069	0.003	-0.588	-0.068	0.014	0.057	0.054	0.08	0.023	0.006	0.005	-0.059	0.012
CD150um lbp 2D glcm JointEnergy	-0.794	-0.052	-0.087	0.004	-0.054	0.059	-0.035	0.008	0.018	0.022	0.026	0.038	0.068	-0.035	-0.069	-0.005	-0.061	0.026	0.033	-0.021	0.088	0.007	-0.014	0.05	0.03	-0.069	0.025
CD150um lbp 2D glcm Idm	-0.023	-0.079	0.033	0.185	0.131	-0.168	-0.065	-0.137	0.177	0.056	-0.276	0.043	0.045	-0.152	-0.627	0.042	0.002	0.009	-0.027	0.136	0.041	0.044	-0.026	0.058	0.038	-0.077	0.02
CD150um lbp 2D glcm InverseVariance	-0.587	-0.186	0.126	-0.181	-0.07	-0.083	-0.054	-0.048	0.123	0.04	-0.212	0.03	0.056	0.00E+00	-0.199	0.125	0.021	-0.045	0.001	0.061	0.139	0.025	-0.108	0.184	0.017	-0.077	0.114
CD150um lbp 2D glcm MaximumProbability	-0.606	-0.017	-0.162	0.22	-0.12	-0.011	-0.056	0.071	0.099	-0.035	0.055	0.125	0.147	-0.062	-0.192	0.006	-0.186	0.074	0	-0.022	0.032	-0.052	-0.03	0.117	0.104	-0.079	0.065
CD150um lbp 2D glcm SumEntropy	0.794	0.109	0.052	0.031	0.055	-0.008	0.029	-0.001	-0.036	-0.076	-0.043	-0.028	-0.035	0.03	0.09	0.001	0.055	-0.012	-0.034	0.026	0.004	-0.01	0.042	-0.009	-0.014	0.055	-0.046
CD150um lbp 2D glcm SumSquares	0.521	0.285	-0.099	0.002	-0.003	0.193	0.069	0.079	-0.156	-0.137	0.193	-0.045	-0.049	-0.002	0.338	0.021	-0.082	0.03	0.022	0.078	0.127	-0.049	0.189	-0.029	-0.01	0.02	0.017
CD150um lbp 2D glrlm RunVariance	0.247	0.159	-0.044	0.51	-0.002	-0.211	-0.021	-0.064	0.056	0.004	-0.012	0.146	-0.132	-0.005	-0.283	-0.07	0.03	0.201	-0.014	0.116	0.004	0.062	0.017	0.267	0.086	-0.026	-0.006
CD150um lbp 2D glrlm ShortRunEmphasis	-0.482	-0.125	0.002	-0.202	0.008	0.06	0.01	0.089	-0.055	-0.009	0.145	0.039	-0.007	0.202	0.514	0.012	0.004	0.013	0.016	-0.003	0.012	0.006	-0.103	0.11	0.007	-0.01	0
CD150um lbp 2D glszm ZoneEntropy	0.747	0.199	0.12	0.101	-0.158	-0.036	0.015	0.03	0.01	-0.02	-0.035	-0.011	-0.039	-0.039	-0.1	0.027	0.091	0.021	-0.012	0.014	-0.053	0.009	0.035	-0.062	0.012	0.022	-0.039
CD150um lbp 2D gldm GrayLevelVariance	0.621	0.343	-0.038	0.044	0.008	0.101	0.087	0.039	-0.127	-0.099	0.112	-0.018	-0.056	0.042	0.253	0.047	-0.07	0.069	0.005	0.08	0.063	-0.038	0.15	-0.03	-0.031	0.029	0.031
CD150um lbp 2D gldm LargeDependenceEmphasis	0.425	0.191	-0.006	0.374	0.041	-0.14	-0.017	-0.076	0.078	0.001	-0.128	0.042	-0.026	-0.142	-0.479	-0.02	-0.034	0.059	0.009	0.061	-0.028	0.037	0.06	-0.007	0.038	-0.03	-0.013
CD150um lbp 2D gldm LDHGLE	0.578	0.32	0.056	0.351	-0.197	0.052	0.072	0.03	-0.077	-0.029	0.042	0.02	-0.092	0.012	-0.091	-0.037	-0.017	0.086	0.082	0.054	0.011	-0.034	0.127	0.03	-0.03	0.015	0.06
CD150um lbp 2D gldm LDLGLE	-0.48	-0.041	-0.109	-0.034	0.523	-0.154	-0.052	-0.022	-0.178	0	-0.006	0.001	-0.022	-0.084	-0.012	-0.009	-0.093	-0.182	-0.084	0.039	0.105	0.077	-0.015	-0.048	0.017	0.077	-0.049
CD150um lbp 2D gldm SDHGLE	0.599	0.35	0.101	-0.009	-0.218	0.047	0.127	0.051	-0.078	-0.083	0.042	-0.011	-0.118	0.125	0.207	0.049	-0.01	0.136	0.031	0.038	-0.041	-0.031	0.076	0.016	-0.009	0.016	-0.013
CD150um lbp 2D gldm SDLGLE	-0.585	-0.058	-0.147	-0.053	0.415	-0.058	-0.026	0.011	-0.183	-0.04	0.084	0.014	0.031	-0.03	0.122	-0.053	-0.1	-0.183	-0.104	0.032	0.071	0.03	-0.015	-0.045	-0.033	0.056	-0.001
CD150um lbp 2D ngtdm Complexity	0.479	0.16	-0.198	-0.068	0.103	0.079	0.081	0.087	0.046	-0.113	0.412	-0.048	0.076	0.044	0.263	-0.009	-0.254	-0.032	0.005	0.043	0.022	-0.038	0.087	-0.063	-0.057	-0.066	-0.031
CD200um wav LH firstorder 90Percentile	-0.026	-0.677	0.28	0.081	0.085	0.027	-0.133	0.04	0.001	-0.048	-0.041	-0.051	0.009	0.005	-0.038	0.095	-0.012	-0.084	-0.059	-0.101	0.126	0.08	0.009	0.128	0.03	0.11	0.083
CD200um wav LH firstorder InterquartileRange	-0.172	-0.682	0.156	-0.003	0.108	0.021	-0.083	-0.015	0.055	0.027	-0.039	0.012	0.076	0.052	-0.083	0.017	-0.058	-0.042	0.002	-0.083	0.081	0.134	0.01	0.148	-0.047	0.006	0.238
CD200um wav LH firstorder Kurtosis	0.34	0.44	0.289	-0.04	-0.127	-0.051	0.272	0.125	-0.158	0.027	-0.027	-0.131	-0.134	0.054	-0.009	-0.115	0.021	0.066	0.001	0.067	0.026	-0.091	-0.033	-0.05	-0.004	-0.027	0.049
CD200um wav LH firstorder Maximum	0.385	-0.068	0.58	0.004	-0.134	0.031	0.106	0.048	-0.112	-0.046	-0.014	-0.104	-0.074	-0.09	-0.048	-0.049	0.022	0.058	0.018	-0.053	0.071	0.065	-0.091	-0.011	0.012	-0.014	0.104
CD200um wav LH firstorder Mean	-0.052	0.264	-0.044	0.07	0.094	-0.15	0.084	0.136	-0.622	0.063	0.075	0.257	-0.047	0.075	-0.036	0.026	-0.033	0.026	0.063	-0.018	0	0.023	-0.143	-0.047	0.019	0.003	-0.016
CD200um wav LH firstorder Median	-0.133	0.276	-0.029	0.103	0.033	-0.261	0.062	-0.025	-0.623	0.119	-0.051	0.073	-0.079	0.107	0.031	0.019	-0.143	0.042	0.091	0.021	0.018	0.016	-0.146	-0.036	0.08	0.027	-0.069
CD200um wav LH firstorder Range	0.355	-0.165	0.605	0.034	-0.144	-0.001	0.089	0.042	-0.123	0.034	-0.054	-0.162	-0.092	-0.003	-0.06	-0.073	-0.02	0.044	0.016	-0.03	0.152	0.016	-0.061	0.01	0.009	0.011	0.089
CD200um wav LH firstorder Uniformity	-0.666	0.326	-0.179	-0.007	0.174	-0.064	0.036	-0.092	0.006	-0.004	-0.02	0.04	-0.04	0.008	0.115	-0.028	0.06	-0.034	-0.059	0.055	-0.051	-0.106	0.043	-0.103	-0.044	0.037	0.003
CD200um wav LH glcm InverseVariance	0.021	0.698	-0.062	0.032	0.219	-0.072	0.083	-0.081	-0.09	-0.086	0.069	-0.024	-0.074	0.103	0.152	-0.053	-0.052	-0.142	-0.064	0.001	0.025	-0.062	0.05	-0.053	-0.057	-0.003	-0.011
CD200um wav LH glszm SZNUN	-0.242	-0.666	-0.053	-0.008	0.08	-0.093	-0.126	0.079	-0.033	0.019	-0.078	-0.029	0.102	-0.013	-0.051	0.012	-0.201	0.028	-0.022	-0.054	0.008	0.075	-0.15	-0.013	0.197	-0.046	0.05
CD200um wav LH gldm DependenceVariance	0.254	0.661	0.013	0.028	0.098	0.029	0.12	-0.088	0.018	0.036	0.11	0.094	-0.188	0.079	0.077	-0.033	0.128	0.015	-0.021	0.027	0.045	-0.125	0.039	0.115	-0.089	0.061	-0.027

Pathomic feature name	P F1	P F2	P F3	P F4	P F5	P F6	P F7	P F8	P F9	P F10	P F11	P F12	P F13	P F14	P F15	P F16	P F17	P F18	P F19	P F20	P F21	P F22	P F23	P F24	P F25	P F26	P F27
CD200um wav LH gldm LDLGLE	-0.729	0.072	-0.175	0.013	0.185	-0.039	-0.059	-0.081	0.051	-0.036	-0.033	0.013	0.02	-0.046	0.088	-0.021	-0.002	-0.027	-0.058	0.053	0.02	-0.03	0.065	-0.065	-0.056	0.058	0.047
CD200um wav LH ngtdm Busyness	-0.497	0.227	-0.211	-0.036	0.1	0.018	0.432	-0.054	-0.046	-0.059	0.052	0.201	-0.056	-0.02	0.032	0	0.049	-0.049	-0.031	0.046	-0.214	0.036	-0.025	-0.105	-0.022	0.034	0.003
CD200um wav LH ngtdm Complexity	0.287	-0.155	0.654	0.174	-0.069	-0.032	0.059	0.015	-0.041	-0.101	0.022	-0.107	0.006	-0.055	-0.018	0.044	0.007	0.012	-0.012	-0.076	0.099	0.043	-0.093	0.055	0.023	0.031	0.102
CD200um wav HL firstorder 10Percentile	0.094	0.624	-0.342	-0.054	0.035	0.004	0.079	0.045	-0.093	0.081	-0.025	0.116	0.081	0.014	-0.062	0.117	-0.181	0.126	0.073	-0.099	0.049	0.068	-0.004	0.099	0.025	0.048	0.125
CD200um wav HL firstorder 90Percentile	-0.127	-0.62	0.309	0.105	0.03	0.068	-0.1	0.036	0.204	-0.159	0.019	-0.001	-0.019	0.109	-0.002	-0.045	0.137	-0.12	-0.055	-0.001	-0.024	-0.064	-0.037	0.028	-0.049	-0.075	-0.113
CD200um wav HL firstorder Mean	-0.143	0.488	-0.153	0.06	-0.096	-0.064	0.056	0.159	0.112	0.032	0.144	0.349	0.172	0.046	-0.009	0.11	-0.082	0.054	0.122	-0.13	0.03	0.092	-0.05	0.144	0.024	-0.081	0.182
CD200um wav HL firstorder Median	-0.292	0.523	-0.164	0.096	-0.289	-0.097	0.015	0.009	-0.002	0.157	0.086	0.149	0.066	-0.069	0.104	0.062	-0.119	0.106	0.127	-0.108	0.152	0.035	-0.076	-0.054	0.092	-0.098	0.103
CD200um wav HL firstorder Minimum	-0.257	0.306	-0.563	-0.049	0.056	0.032	-0.016	-0.134	0.095	-0.073	-0.067	0.012	-0.003	-0.002	0.021	0.002	-0.109	-0.066	-0.023	-0.153	0.145	0.158	0.073	0.007	0.046	0.229	0.093
CD200um wav HL firstorder Range	0.313	-0.239	0.629	0.003	-0.089	-0.044	0.049	0.109	-0.026	0.037	0.064	-0.058	0.025	-0.004	-0.031	-0.047	0.094	0.041	0.028	0.041	-0.141	-0.005	-0.087	0	-0.01	-0.188	-0.059
CD200um wav HL firstorder Skewness	0.258	-0.081	0.171	-0.115	-0.17	-0.005	0.046	-0.005	0.117	-0.226	-0.135	-0.188	0.093	-0.039	-0.12	-0.067	-0.123	-0.065	-0.041	-0.082	0.007	0.522	-0.026	0.026	0.007	0.066	0.015
CD200um wav HL firstorder TotalEnergy	0.234	0.137	0.433	0.026	-0.094	0.045	0.556	0.092	-0.024	-0.099	-0.003	0.05	-0.01	-0.046	0.056	-0.055	0.023	0.041	0.033	-0.01	-0.062	0.095	-0.121	-0.04	0.043	-0.083	0.01
CD200um wav HL glcm ClusterProminence	-0.039	-0.399	0.491	0.057	0.151	-0.021	-0.067	0.033	0.101	-0.109	0.076	-0.022	0.074	0.126	-0.096	0.014	0.259	-0.174	-0.017	0.044	-0.116	-0.08	0.04	0.136	-0.098	-0.123	0.041
CD200um wav HL glcm Correlation	0.527	0.061	0.097	0.148	-0.012	0.12	0.014	0.034	0.36	0.1	0.089	-0.003	0.101	-0.057	-0.125	-0.132	-0.065	-0.104	0.008	0.068	0.286	0.067	0.073	-0.043	0.066	0.004	-0.007
CD200um wav HL glcm MaximumProbability	-0.776	-0.031	-0.126	-0.013	0.071	-0.068	-0.042	-0.048	-0.117	0.035	-0.142	0.007	-0.057	-0.047	-0.002	0.001	0.041	0.009	-0.02	0.002	-0.01	-0.028	0.053	-0.047	-0.006	0.051	-0.029
CD200um wav HL glcm SumEntropy	0.726	0.064	0.286	0.087	-0.16	0.041	-0.006	0.086	0.043	-0.064	0.048	0.005	-0.011	0.002	0.01	0.012	0.004	-0.013	0.011	-0.027	0.039	0.062	-0.077	0.015	0.054	-0.046	-0.047
CD200um wav HL ngtdm Busyness	-0.4	0.372	-0.127	0.05	0.42	-0.068	0.251	-0.097	0.06	-0.078	-0.061	0.097	0.017	-0.008	0.059	-0.062	0.043	-0.065	-0.183	-0.011	0.07	0.011	0.038	-0.07	-0.044	0.161	0.063
CD200um wav HL ngtdm Complexity	0.179	-0.239	0.652	0.12	0.017	0.014	0.052	0.078	0.052	-0.175	0.046	-0.003	0.043	0.024	-0.014	-0.018	0.164	-0.125	-0.028	-0.023	-0.098	-0.013	-0.095	0.095	-0.026	-0.115	0.024
CD200um wav HL ngtdm Strength	-0.225	-0.624	0.249	0.013	0.117	-0.003	-0.172	0.025	0.152	0.014	0.047	-0.136	0.064	0.066	-0.052	-0.03	0.154	-0.056	0.024	0.094	-0.058	-0.07	0.092	0.01	-0.049	-0.104	-0.084
CD200um wav HH firstorder 10Percentile	-0.104	0.645	-0.327	-0.15	-0.038	-0.055	0.078	-0.083	-0.216	0.067	0.029	0.015	-0.047	-0.127	-0.013	0.025	-0.016	0.07	0.01	-0.017	-0.047	0.038	0.018	-0.073	0.046	-0.075	-0.063
CD200um wav HH firstorder 90Percentile	-0.103	-0.704	0.301	0.113	0.064	0.006	-0.067	0.049	0.148	-0.09	0.02	-0.062	-0.062	0.04	0.034	-0.012	-0.058	-0.067	-0.014	0.014	-0.041	-0.014	-0.004	0.089	-0.027	0.031	0.007
CD200um wav HH firstorder InterquartileRange	-0.087	-0.628	0.3	0.04	0.002	-0.083	-0.066	0.092	0.203	-0.135	0.008	0.081	0.005	0.062	0.068	0.003	0.099	-0.137	-0.037	0.05	-0.063	-0.132	-0.053	0.091	-0.073	0.124	0.01
CD200um wav HH firstorder Kurtosis	0.481	0.346	0.242	-0.106	-0.052	0.053	0.119	0.12	-0.09	0.166	0.036	-0.146	-0.014	0.071	-0.079	-0.034	-0.006	0.038	0.256	-0.005	-0.052	-0.144	0.089	0.03	0.036	-0.001	0.02
CD200um wav HH firstorder Maximum	0.315	-0.37	0.538	0.073	-0.012	0.056	0.054	0.186	0.103	-0.068	0.024	-0.02	-0.006	0.109	-0.034	-0.025	0.038	-0.029	0.094	0.119	-0.026	-0.096	0.009	0.111	-0.027	0.037	-0.011
CD200um wav HH firstorder Skewness	0.16	0.074	-0.075	0.074	-0.059	0.044	-0.017	0.047	0.043	-0.045	-0.127	-0.155	-0.069	-0.072	-0.089	-0.034	0.024	0.025	-0.079	0.648	0.008	-0.023	0.022	0.013	0.02	0.015	-0.009
CD200um wav HH firstorder Uniformity	-0.639	0.435	-0.14	0.002	0.099	-0.009	-0.026	-0.037	0.133	0.013	0.024	0.062	0.082	-0.042	0.018	0.026	0.085	-0.019	-0.054	-0.026	0.092	-0.009	0.027	-0.043	-0.017	-0.024	0.02
CD200um wav HH glcm JointAverage	0.295	-0.304	0.61	-0.013	-0.014	0.04	0.091	0.189	0.086	-0.034	0.025	-0.025	0.039	0.107	0.001	-0.024	-0.009	-0.051	0.094	-0.143	-0.015	-0.011	-0.017	0.098	0.031	0.072	-0.019
CD200um wav HH gldm DependenceVariance	0.115	0.703	-0.051	0.016	-0.151	0.045	0.089	-0.02	0.124	-0.028	-0.046	0.04	0.012	0.117	-0.188	0.026	-0.011	0.024	-0.035	0.086	-0.101	0.004	-0.088	0.009	0.082	0.017	-0.012
CD200um wav HH ngtdm Busyness	-0.399	0.55	-0.054	0.05	0.243	-0.034	0.208	-0.016	0.117	-0.031	0.041	0.139	0.031	-0.051	0.066	-0.01	0.107	-0.032	-0.158	0.061	0.123	0.039	-0.009	-0.021	-0.033	0.026	0.056
CD200um wav HH ngtdm Complexity	0.169	-0.333	0.603	0.184	0.04	0.023	0.036	0.159	0.094	-0.197	0.039	0.082	0.057	0.093	-0.027	0.026	0.04	-0.103	-0.013	-0.015	0.011	-0.086	-0.058	0.125	-0.033	0.058	0.034
CD200um wav LL firstorder InterquartileRange	0.266	-0.279	0.491	0.073	-0.13	0.05	-0.09	0.043	0.174	0.203	-0.171	-0.09	0.028	-0.01	0.045	-0.226	0.007	-0.001	-0.215	0.011	-0.067	0.026	0.051	-0.038	0.084	0.012	0.027
CD200um wav LL firstorder Kurtosis	0.168	0.045	-0.049	0.081	0.16	-0.119	0.108	0	-0.115	0.128	0.195	0.064	-0.028	0.045	0.045	0.272	-0.016	0.04	0.626	-0.091	0.007	-0.008	-0.033	-0.015	-0.002	-0.06	-0.015

Pathomic feature name	P F1	P F2	P F3	P F4	P F5	P F6	P F7	P F8	P F9	P F10	P F11	P F12	P F13	P F14	P F15	P F16	P F17	P F18	P F19	P F20	P F21	P F22	P F23	P F24	P F25	P F26	P F27
CD200um wav LL firstorder Minimum	0.174	-0.013	0.359	-0.422	-0.131	0.021	-0.117	-0.02	-0.007	-0.06	-0.046	-0.447	-0.033	-0.014	-0.018	-0.021	-0.081	0.152	-0.086	-0.042	0.017	-0.059	0.051	-0.031	-0.158	0.066	-0.137
CD200um wav LL firstorder Range	0.435	-0.098	0.57	0.191	-0.163	-0.05	0.169	0.067	0.047	0.141	-0.035	-0.045	0.008	0.064	0.023	-0.074	-0.031	-0.023	0.1	-0.011	0.006	0.078	0.003	-0.037	0.067	-0.004	0.021
CD200um wav LL firstorder Skewness	-0.142	-0.075	-0.217	-0.175	0.289	-0.126	-0.051	0.001	0.009	0.587	0.015	0.181	0.071	-0.013	-0.085	-0.047	0.037	-0.073	0.113	-0.138	0.039	-0.06	-0.016	0.054	-0.046	-0.03	-0.076
CD200um wav LL glcm JointAverage	0.364	-0.102	0.592	0.207	-0.208	0.012	0.141	0.045	0.047	-0.172	-0.036	-0.112	0.006	0.031	0.087	-0.055	-0.032	-0.006	0.003	0.011	-0.066	0.044	0.026	-0.04	0.043	0.016	0.074
CD200um wav LL glcm ClusterShade	-0.036	0.026	-0.135	-0.655	0.133	-0.154	-0.006	0.039	0.048	0.304	-0.029	0.03	0.03	0.013	-0.057	-0.037	0.025	0.049	0.051	-0.015	0.019	0.154	0.007	0.089	-0.154	0.005	-0.089
CD200um wav LL glcm ClusterTendency	0.251	-0.148	0.473	0.439	-0.09	-0.094	-0.045	0.058	0.044	0.257	-0.061	-0.009	0.087	0.062	0.022	-0.091	0.007	-0.003	0.028	-0.01	0.015	0.151	0.026	-0.102	0.16	-0.004	-0.004
CD200um wav LL glcm Contrast	0.042	-0.541	0.428	0.178	0.062	0.048	-0.094	-0.057	0.259	0.027	-0.055	-0.231	-0.002	0.035	-0.006	-0.036	0.01	-0.096	0.003	0.024	0.09	0.073	0.035	0.023	0.025	-0.004	0.005
CD200um wav LL glcm Correlation	0.591	0.327	0.172	0.135	0.038	-0.122	0.092	0.008	-0.13	0.269	-0.016	0.159	-0.012	0.048	0.00E+00	-0.082	0.003	0.057	-0.022	0.035	-0.09	0.035	0.025	-0.022	0.033	0.034	0.047
CD200um wav LL glcm DifferenceVariance	0.176	-0.372	0.544	0.209	0.007	-0.001	-0.113	-0.019	0.183	0.012	-0.026	-0.292	0.02	0.009	0.008	-0.003	0.031	-0.056	0.039	0.013	0.091	0.097	0.038	0	0.081	-0.019	-0.048
CD200um wav LL glcm Imc2	0.044	-0.241	-0.048	0.049	0.034	-0.061	-0.701	-0.078	0.11	0.086	-0.018	-0.104	0.09	-0.038	-0.038	-0.077	0.005	0.017	-0.143	-0.061	0.061	-0.05	0.072	0.064	0.022	-0.048	0.008
CD200um wav LL glrlm ShortRunEmphasis	-0.372	-0.604	0.019	0.024	-0.009	0.031	-0.146	0.018	0.068	0.029	-0.168	0.026	0.121	-0.025	-0.022	-0.063	0.051	0.103	-0.132	-0.189	0.061	0.122	-0.074	-0.018	0.012	-0.001	-0.048
CD200um wav LL gldm LDLGLE	-0.607	0.199	-0.125	0.068	0.418	-0.043	-0.049	-0.057	0.108	-0.023	-0.037	0.036	0.046	-0.041	0.07	-0.025	0.045	-0.002	-0.16	0.015	0.065	-0.019	0.008	-0.055	-0.003	0.119	0.019
CD200um wav LL ngtdm Busyness	-0.781	0.066	-0.149	-0.018	0.134	-0.015	0.034	-0.027	-0.058	0.047	-0.027	0.055	-0.059	-0.053	0.023	0.004	0.035	-0.045	0.002	-0.024	0.026	-0.014	-0.046	0.002	-0.011	0.02	-0.038
CD200um wav LL ngtdm Complexity	0.321	-0.03	0.614	0.272	-0.13	-0.001	0.07	-0.015	0.03	0.017	-0.032	-0.106	-0.009	-0.097	0.046	-0.032	-0.004	-0.007	-0.048	-0.099	0.028	0.097	-0.075	-0.045	0.165	-0.029	-0.061
CD200um lbp 2D firstorder Kurtosis	-0.489	-0.131	-0.08	-0.039	0.146	0.033	-0.048	-0.117	0.448	0.068	-0.173	-0.038	0.174	0.044	-0.244	-0.035	-0.113	0.049	-0.001	0.045	0.058	0.055	-0.074	0.048	0.014	-0.13	-0.013
CD200um lbp 2D firstorder Skewness	-0.464	-0.284	-0.097	-0.096	0.205	0.053	-0.134	-0.054	0.405	0.064	-0.1	-0.03	0.239	-0.035	-0.105	-0.013	-0.008	-0.078	-0.04	-0.015	0.152	0.072	-0.028	0.048	-0.021	-0.107	-0.03
CD200um lbp 2D firstorder Uniformity	-0.68	-0.307	-0.131	-0.054	0.174	-0.056	-0.007	-0.07	-0.057	0.043	-0.023	-0.047	-0.033	-0.059	-0.157	-0.071	-0.062	-0.007	-0.006	0.062	-0.093	-0.02	-0.016	-0.047	0.018	-0.015	-0.072
CD200um lbp 2D glcm ClusterProminence	0.488	0	-0.005	0.02	-0.001	0.174	0.006	0.131	-0.262	-0.078	0.433	0.026	0.172	0.06	0.086	-0.021	0.138	0.029	-0.058	-0.01	0.161	-0.096	-0.036	-0.064	0.011	-0.018	-0.027
CD200um lbp 2D glcm ClusterTendency	0.553	0.037	0.024	0.049	-0.07	0.198	0.011	0.178	-0.218	-0.031	0.378	0.043	0.153	0.076	0.079	0.039	0.152	0.062	-0.032	0.013	0.159	-0.028	0.017	-0.038	0.005	0.012	0.033
CD200um lbp 2D glcm Contrast	0.479	0.151	-0.001	-0.038	-0.058	0.142	0.006	0.271	0.024	-0.1	0.493	0.066	-0.025	-0.111	0.113	0.121	-0.022	-0.031	0.098	-0.045	-0.057	-0.034	-0.007	0.101	0.014	0.027	0.035
CD200um lbp 2D glcm Correlation	0.112	-0.154	0.054	0.089	0.047	0.099	0.017	-0.074	-0.575	0.088	-0.126	-0.062	0.136	0.228	0.021	-0.111	0.171	0.03	-0.114	0.01	0.271	0.03	0.03	-0.086	-0.02	0.041	-0.032
CD200um lbp 2D glcm DifferenceEntropy	0.71	0.214	0.095	0.017	-0.082	0.039	-0.008	0.152	0.096	-0.059	0.212	0.044	0.023	-0.027	0.084	0.087	0.09	-0.031	0.048	-0.017	0.046	0.005	0.026	0.039	-0.01	0.007	-0.038
CD200um lbp 2D glcm DifferenceVariance	0.344	0.183	-0.001	-0.038	-0.045	0.045	-0.038	0.225	0.139	-0.122	0.52	0.072	0.109	-0.172	-0.012	0.083	-0.189	-0.053	0.037	-0.144	0.025	-0.004	-0.1	0.138	0.089	-0.014	0.088
CD200um lbp 2D glcm JointEnergy	-0.724	-0.228	-0.079	-0.023	0.074	-0.032	0.004	-0.036	-0.207	0.02	-0.059	-0.031	-0.094	-0.022	-0.112	-0.043	-0.05	0.012	0.004	0.02	-0.099	-0.025	-0.039	-0.01	0.036	0.014	-0.074
CD200um lbp 2D glcm Idm	-0.567	-0.04	-0.033	0.037	0.085	-0.23	-0.016	-0.169	-0.159	-0.001	-0.249	-0.023	0.007	-0.042	-0.235	-0.079	-0.238	0.011	-0.041	-0.066	-0.03	0.005	-0.16	0.012	0.124	-0.032	-0.029
CD200um lbp 2D glcm Idn	-0.488	-0.051	-0.028	0.034	0.096	-0.101	-0.05	-0.225	0.107	0.065	-0.364	-0.042	0.195	0.063	-0.258	-0.104	-0.162	0.063	-0.099	-0.045	0.124	0.05	-0.103	-0.003	0.082	-0.085	0.033
CD200um lbp 2D glcm InverseVariance	-0.526	-0.138	-0.038	-0.071	0.12	-0.178	0.011	-0.198	-0.095	0.074	-0.406	-0.072	-0.003	0.072	-0.064	-0.074	-0.228	-0.033	-0.007	-0.062	0.021	0.061	-0.096	0.032	-0.011	0.041	0.083
CD200um lbp 2D glcm MaximumProbability	-0.686	-0.203	-0.101	0.028	0.065	0.003	-0.031	0.011	-0.206	-0.011	-0.013	0.018	-0.028	-0.019	-0.188	-0.036	-0.035	0.112	0.02	0.046	-0.124	-0.043	-0.039	0.027	0.145	0.011	-0.069
CD200um lbp 2D glcm SumEntropy	0.747	0.221	0.076	0.045	-0.103	0.032	0.007	0.096	0.023	-0.038	0.119	0.026	0.054	0.016	0.089	0.056	0.04	-0.012	0.009	-0.046	0.104	0.038	-0.01	0.029	-0.015	0.031	0.03

Pathomic feature name	P F1	P F2	P F3	P F4	P F5	P F6	P F7	P F8	P F9	P F10	P F11	P F12	P F13	P F14	P F15	P F16	P F17	P F18	P F19	P F20	P F21	P F22	P F23	P F24	P F25	P F26	P F27
CD200um lbp 2D glcm SumSquares	0.549	0.104	0.011	0.002	-0.068	0.18	0.009	0.243	-0.095	-0.072	0.47	0.059	0.063	-0.025	0.104	0.088	0.064	0.014	0.04	-0.019	0.047	-0.034	0.004	0.038	0.011	0.021	0.037
CD200um lbp 2D glrlm LongRunEmphasis	0.018	0.209	0.067	0.263	-0.059	-0.257	-0.005	-0.117	-0.181	-0.188	0.12	0.046	-0.058	-0.066	-0.287	-0.079	-0.141	0.107	-0.008	0.019	-0.029	-0.021	-0.081	0.031	0.458	-0.025	-0.037
CD200um lbp 2D glszm ZoneEntropy	0.635	0.381	0.174	0.085	-0.232	-0.007	0.02	0.035	-0.045	-0.087	-0.02	0.065	-0.012	-0.021	-0.008	0.035	-0.006	0.029	-0.008	-0.077	0.043	-0.024	-0.037	0.043	0.044	-0.023	0.038
CD200um lbp 2D gldm GrayLevelVariance	0.535	0.187	0.05	0.026	-0.118	0.16	0.029	0.225	-0.129	-0.065	0.457	0.083	0.025	-0.011	0.101	0.078	0.055	0.025	0.062	-0.029	0.052	-0.048	0.004	0.039	0.015	0.009	0.041
CD200um lbp 2D gldm LDHGLE	0.448	0.33	0.092	0.251	-0.174	-0.007	0.077	0.094	-0.168	-0.07	0.4	0.024	-0.089	0.021	-0.006	-0.01	-0.021	0.108	0.075	0.012	-0.016	-0.045	-0.011	-0.002	0.159	-0.024	0.055
CD200um lbp 2D gldm SDHGLE	0.596	0.319	0.126	-0.014	-0.248	0.137	0.085	0.139	-0.099	-0.05	0.209	0.054	-0.034	0.068	0.074	0.057	0.058	0.071	0.051	-0.025	0.032	-0.025	0.06	0.034	-0.019	0.059	0.048
CD200um lbp 2D gldm SDLGLE	-0.51	-0.415	-0.104	-0.076	0.348	0.045	-0.04	0.127	-0.096	0.065	0.098	0.026	-0.031	-0.047	0.038	0.018	0.003	-0.116	0.043	0.129	-0.071	-0.007	-0.082	0.062	-0.05	0.055	0.005
CD200um lbp 2D ngtdm Busyness	0.189	0.231	0.202	-0.057	-0.096	0.02	0.708	0.115	-0.036	-0.018	0.013	0.071	-0.038	-0.008	0.029	-0.046	-0.033	0.039	0.04	0.015	-0.019	0.063	-0.045	-0.014	-0.002	-0.041	0.009
CD200um lbp 2D ngtdm Complexity	0.593	0.101	0.02	-0.044	-0.07	0.231	-0.02	0.261	-0.088	-0.109	0.274	0.068	-0.018	-0.11	0.1	0.127	0.093	-0.04	0.044	0.01	-0.017	-0.109	0.047	0.115	0.033	0.084	0.031
CD200um lbp 2D ngtdm Contrast	0.14	-0.026	-0.113	-0.014	0.015	0.029	0.057	0.172	-0.087	0	0.746	0.019	0.049	-0.043	0.09	0.006	-0.102	0.012	0.069	-0.062	-0.043	0.023	-0.034	-0.037	-0.003	-0.078	-0.042

Table S14 Summary of the significantly correlated radiomic-pathomic factors, with radiomic factors extracted from ADC. Abbreviations: ADC = Apparent Diffusion Coefficient; FDR = False Discovery Rate; BF = Bayes Factor; F = Factor.

Radiomic factor name	Pathomic factor name	ρ	FDR q-value	BF
ADC_F7	P_F8	3.81×10^{-3}	0.643	1.97×10^{4}
ADC_F10	P_F8	3.81×10^{-3}	0.637	1.46×10^{4}
ADC_F5	P_F3	4.46×10^{-3}	0.614	4.93×10^{3}
ADC_F2	P_F24	2.21×10^{-3}	0.613	4.65×10^{3}
ADC_F1	P_F22	1.37×10^{-2}	0.528	1.83×10^{2}

Table S15 Summary of the significantly correlated radiomic-pathomic factors, with radiomic factors extracted from T1C. Abbreviations: T1C = post-contrast T1; FDR = False Discovery Rate; BF = Bayes Factor; F = Factor.

Radiomic factor name	Pathomic factor name	ρ	FDR q-value	BF
T1C_F7	P_F9	2.47×10^{-2}	0.597	2.38×10^{3}
T1C_F13	P_F7	2.47×10^{-2}	0.586	1.53×10^{3}
T1C_F2	P_F7	4.10×10^{-2}	-0.545	3.23×10^{2}
